# Cox model inference for relative hazard and pure risk from stratified weight-calibrated case-cohort data

**DOI:** 10.1007/s10985-024-09621-2

**Published:** 2024-04-02

**Authors:** Lola Etievant, Mitchell H. Gail

**Affiliations:** Division of Cancer Epidemiology and Genetics, Biostatistics Branch, National Cancer Institute, 9609 Medical Center Drive, Rockville, MD 20850-9780 USA

**Keywords:** Case-cohort, Weight calibration, Stratification, Variance estimation, Two-phase sampling, Influence functions

## Abstract

The case-cohort design obtains complete covariate data only on cases and on a random sample (the subcohort) of the entire cohort. Subsequent publications described the use of stratification and weight calibration to increase efficiency of estimates of Cox model log-relative hazards, and there has been some work estimating pure risk. Yet there are few examples of these options in the medical literature, and we could not find programs currently online to analyze these various options. We therefore present a unified approach and R software to facilitate such analyses. We used influence functions adapted to the various design and analysis options together with variance calculations that take the two-phase sampling into account. This work clarifies when the widely used “robust” variance estimate of Barlow (Biometrics 50:1064–1072, 1994) is appropriate. The corresponding R software, CaseCohortCoxSurvival, facilitates analysis with and without stratification and/or weight calibration, for subcohort sampling with or without replacement. We also allow for phase-two data to be missing at random for stratified designs. We provide inference not only for log-relative hazards in the Cox model, but also for cumulative baseline hazards and covariate-specific pure risks. We hope these calculations and software will promote wider use of more efficient and principled design and analysis options for case-cohort studies.

**Supplementary Information:**

The online version contains supplementary material available at 10.1007/s10985-024-09621-2.

## Introduction

Prentice (1986) described the case-cohort design for time-to-response outcomes, in which one obtains covariate information on all cases (those with the event) and on a random subcohort (which may include some cases) from the entire study cohort. Two great advantages of this design are that hard-to-measure covariates need only be obtained for the cases and subcohort, which is much smaller than the entire study cohort, and the data from the subcohort can be used for several different types of time-to-response outcomes. There have been subsequent refinements and extensions of this design. Barlow (1994) proposed a widely used “robust” variance estimator for log-relative hazards (RH) based on the sum of squared influences. Borgan et al. (2000) showed that a stratified case-cohort design had increased efficiency, and Samuelsen et al. (2007) and Gray (2009) noted that the “robust” variance estimate overestimated variances of log-relative hazard estimates with stratification when sampling without replacement. Breslow et al. (2009a, 2009b) proposed survey weight calibration to improve efficiency of case-cohort estimates of relative hazard. Although much of this literature focused on estimation of log-relative hazards, some authors considered estimation of cumulative baseline hazard and covariate-specific “pure” risk of an event (Chapters 16 and 17 in Borgan et al. 2017; Breslow and Lumley 2013; Gray 2009).

Sharp et al. (2014) noted variability in the analysis and reporting of 32 case-cohort studies from 24 major medical and epidemiological journals. None of these analyses used weight calibration, some used an inappropriate “robust” variance estimate with stratified data, and various methods were used for missing covariate information. Our informal review of subsequent case-cohort publications also indicates that stratification, weight calibration, a principled approach to missing subcohort data, and analysis of pure risk are underutilized. This may be partly due to difficulty understanding the highly technical and varied methodologic literature and to lack of convenient software.

To facilitate wider use of improved design and analysis options for case-cohort data, we unify the various analytic options above by presenting empirical influence functions for log-relative hazards and pure risk under a Cox proportional hazards model. These influence functions are adapted to the various design and analytic options above, and variance calculations acknowledge the phase-one sampling of the cohort from a superpopulation and the phase-two sampling of the subcohort. We develop software so that users can conveniently analyze case-cohort data with or without stratification and with or without weight calibration and can handle stratified case-cohort data with missing phase-two data.

We introduce notation in Sect. 2 and inference for the stratified case-cohort design in Sect. 3, which includes the unstratified design as a special case with one stratum. We describe weight calibration in Sect. 4, and methods for missing phase-two data in Sect. 5. We discuss current software in Sect. 6. Sections 7 and 8 present simulations and a data illustration, where we investigate the comparative efficiencies associated with stratification and weight calibration for hazards and covariate-specific pure risk, and how the “robust” variance estimate performs, with or without calibration. Concluding remarks are in Sect. 9. Most technical derivations and details are in Web Appendices.

## Notation

We let $J$ be the number of strata in the whole cohort, $n_{ }^{\left( j \right)}$ be the number of subjects in stratum $j$, $j \in \left\{ {1, \ldots ,J} \right\}$. Then $n = \sum\nolimits_{j = 1}^{J} {n_{ }^{\left( j \right)} }$ is the number of subjects in the whole cohort. We allow for right censoring and left truncation. We let $T_{i,j}$ be the event time (or age if the analysis is on the age scale) for subject $i$ in stratum $j$, and $C_{i,j}$ be the censoring time for subject $i$ in stratum $j$, $i \in \left\{ {1, \ldots ,n_{ }^{\left( j \right)} } \right\}$, $j \in \left\{ {1, \ldots ,J} \right\}$. Using the time-on-study scale, the at-risk indicator for subject $i$ in stratum $j$ is $Y_{i,j} \left( t \right) = I\left( {\tilde{T}_{i,j} \ge t} \right)$, with $\tilde{T}_{i,j} = {\text{min}}(T_{ij} ,C_{ij}$). Using the age scale, $Y_{i,j} \left( t \right) = I\left( {\tilde{T}_{i,j} \ge t > E_{i,j} } \right)$, with $E_{i,j}$ the entry age for subject $i$ in stratum $j$. Let $\tau$ the maximum follow-up time or maximum age for analyses on the age scale. With $N_{i,j} \left( t \right) = I\left( {T_{i,j} \le t,T_{ij} \le C_{ij} } \right)$ indicating an observed event before or at time/age $t$ after study entry, ${\text{d}}N_{i,j} \left( t \right)$ indicates if individual $i$ in stratum $j$ fails (has the event) at time/age $t$. Finally, we let ${\varvec{X}}_{i,j}$ be a vector of $p$ baseline covariates for subject $i$ in stratum $j$; ${\varvec{X}}_{i,j}$ includes stratum indicators or stratum determinants.

We assume that failure follows the Cox proportional hazards model with hazard function $\lambda \left( t \right) = \lambda_{0} \left( t \right)\exp \left( {{\varvec{\beta}}^{\prime}{\varvec{X}}} \right)$, for covariates ${\varvec{X}}$, and where $\lambda_{0} \left( t \right)$ is a baseline hazard function, i.e., the hazard for an individual with ${\varvec{X}} = \varvec{0}$. We further assume that $\lambda_{0} \left( t \right)$ is homogeneous across strata and we let $\Lambda_{0} \left( t \right) = \mathop \int \limits_{0}^{t} \lambda_{0} \left( s \right){\text{d}}s$ denote the cumulative baseline hazard.

Estimation from complete cohort data is reviewed in Web Appendix A.1 with corresponding influence functions in Web Appendix A.2.

## Stratified case-cohort

### Estimation of relative hazard, cumulative baseline hazard and pure risk

We assume that a fixed number of individuals, $m_{ }^{\left( j \right)}$, is sampled from stratum $j$ (of size $n_{ }^{\left( j \right)}$) in the cohort, without replacement and independently of case status, $j \in \left\{ {1, \ldots ,J} \right\}$. Sampling is performed independently across strata. The subcohort includes all the sampled subjects from the $J$ strata. In addition, we sample all the cases in the cohort, some of whom may have been included in the subcohort. All of these individuals constitute the stratified case-cohort, that we also call the *phase-two sample*, because it is a subset of the cohort, which is regarded as a *phase-one sample* from a super-population. We let $\xi_{i,j}$ be the sampling indicator of individual $i$ in stratum $j$ and $$w_{i,j} = \left\{ {\begin{array}{*{20}l} {\frac{{n_{ }^{\left( j \right)} }}{{m_{ }^{\left( j \right)} }}} \hfill & {{\text{if }}i{\text{ is a non-case in stratum }}j} \hfill \\ 1 \hfill & {{\text{if }}i{\text{ is a case in stratum }}j{ }} \hfill \\ \end{array} } \right.$$ be his/her known design weight, $i \in \left\{ {1, \ldots ,n_{ }^{\left( j \right)} } \right\}$, $j \in \left\{ {1, \ldots ,J} \right\}$. We assume that some of the covariates in ${\varvec{X}}$ are only measured in the phase-two sample; we call these “phase-two covariates”. The stratum indicators are known for all members of the cohort and are not phase-two covariates. Because we sample all cases, $\xi_{i,j} w_{i,j} = 1$ for cases. Non-stratified case-cohort data correspond to the special case $J = 1$.

An estimate of the log-relative hazard ${\varvec{\beta}}$ is obtained by solving the estimating equation

1 $${\varvec{U}}\left( {\varvec{\beta}} \right) = \mathop \sum \limits_{j = 1}^{J} \mathop \sum \limits_{i = 1}^{{n_{ }^{\left( j \right)} }} \mathop \int \limits_{t} \left\{ {{\varvec{X}}_{i,j} - \frac{{{\varvec{S}}_{1} \left( {t;{\varvec{\beta}}} \right)}}{{S_{0} \left( {t;{\varvec{\beta}}} \right)}}} \right\}{\text{d}}N_{i,j} \left( t \right) = 0,$$

with

2 $$S_{0} \left( {t;{\varvec{\beta}}} \right) = \mathop \sum \limits_{j = 1}^{J} \mathop \sum \limits_{k = 1}^{{n_{ }^{\left( j \right)} }} w_{k,j} \xi_{k,j} Y_{k,j} \left( t \right)\exp \left( {\varvec{\beta}^{\prime}\varvec{X}_{k,j} } \right),$$

3 $${\varvec{S}}_{1} \left( {t;{\varvec{\beta}}} \right) = \mathop \sum \limits_{j = 1}^{J} \mathop \sum \limits_{k = 1}^{{n_{ }^{\left( j \right)} }} w_{k,j} \xi_{k,j} Y_{k,j} \left( t \right)\exp \left( {\varvec{\beta}^{\prime}\varvec{X}_{k,j} } \right){\varvec{X}}_{k,j} ,$$

and we also define

4 $${\varvec{S}}_{2} \left( {t;{\varvec{\beta}}} \right) = \mathop \sum \limits_{j = 1}^{J} \mathop \sum \limits_{k = 1}^{{n_{ }^{\left( j \right)} }} w_{k,j} \xi_{k,j} Y_{k,j} \left( t \right)\exp \left( {\varvec{\beta}^{\prime}\varvec{X}_{k,j} } \right){\varvec{X}}_{k,j} {\varvec{X}}_{k,j}^{\prime} .$$

Let $\widehat{\varvec{\beta }}$ denote this solution. We could write Eq. (1) as $\sum\nolimits_{j = 1}^{J} {\mathop \sum \nolimits_{i = 1}^{{n_{ }^{\left( j \right)} }} } \mathop \int_{t} \left\{ {{\varvec{X}}_{i,j} - \frac{{{\varvec{S}}_{1} \left( {t;{\varvec{\beta}}} \right)}}{{S_{0} \left( {t;{\varvec{\beta}}} \right)}}} \right\}\xi_{i,j} w_{i,j} {\text{d}}N_{i,j} \left( t \right) = 0$﻿, because $\xi_{i,j} w_{i,j} = 1$ for cases; this form would be useful if cases were subsampled (see Sect. 9). We then estimate the baseline hazard point mass at time $t$ non-parametrically (Breslow 1974) by

5 $${\text{d}}\widehat{\Lambda }_{0} \left( {t;\widehat{\varvec{\beta }}} \right) \equiv {\text{d}}\widehat{\Lambda }_{0} \left( t \right) = \frac{{\mathop \sum \nolimits_{j = 1}^{J} \mathop \sum \nolimits_{i = 1}^{{n_{ }^{\left( j \right)} }} {\text{d}}N_{i,j} \left( t \right)}}{{S_{0} \left( {t,\widehat{\varvec{\beta }}} \right)}},$$

the cumulative baseline hazard up to time $t$ by

6 $$\widehat{\Lambda }_{0} \left( {t;\widehat{\varvec{\beta }},\widehat{\lambda}_{0} } \right) \equiv \widehat{\Lambda }_{0} \left( t \right) = \mathop \int \limits_{0}^{t} {\text{d}}\widehat{\Lambda }_{0} \left( s \right),$$

and the pure covariate-specific risk for profile ${\varvec{x}}$ in the interval $(\tau_{1} ,\tau_{2} ]$ by

7 $$\widehat{\pi }\left( {\tau_{1} ,\tau_{2} ;{\varvec{x}},\widehat{\varvec{\beta }}, {\text{d}}\widehat{\Lambda }_{0} } \right) \equiv \widehat{\pi }\left( {\tau_{1} ,\tau_{2} ;{\varvec{x}}} \right) = 1 - \exp \left\{ { - \mathop \int \limits_{{\tau_{1} }}^{{\tau_{2} }} \exp \left( {\widehat{\varvec{\beta }}^{\prime}{\varvec{x}}} \right){\text{d}}\widehat{\Lambda }_{0} \left( s \right)} \right\}.$$

In Sects. 3.3 and 4.3 we show how to use influence functions to estimate the variance of $\widehat{\varvec{\beta }}$ and $\widehat{\Lambda }_{0} \left( t \right)$ for a fixed $t$.

### Influence functions

As described in Deville (1999), survey samplers often compute the variance of a statistic $\widehat{\varvec{\theta }}$ with expectation ${\varvec{\theta}}$ by using the linear approximation $\widehat{\varvec{\theta }} - {\varvec{\theta}} = \sum {{\varvec{\Delta}}}_{i} + {\varvec{R}}$, where the remainder ${\varvec{R}}$ is of smaller order than $\widehat{\varvec{\theta }}$ and the summation is over the sample units indexed by $i.$ The variance of $\widehat{\varvec{\theta }}$ can therefore be calculated as ${\text{var}}\left( {\sum {{\varvec{\Delta}}}_{i} } \right)$. The ${{\varvec{\Delta}}}_{i}$ are called Taylor deviates or influences (in other literature, e.g. Tsiatis (2006), the ${{\varvec{\Delta}}}_{i}$ divided by the sample size are called influences). Using the calculus for Taylor deviates described in Deville (1999) and Graubard and Fears (2005), we calculated the influences in this Section. The ${{\varvec{\Delta}}}_{i}$ are theoretical quantities that depend on unknown parameters, but substituting consistent estimates of these parameters to produce “empirical” influences still yields asymptotically consistent variance estimates (Deville 1999).

We let ${{\varvec{\Delta}}}_{i,j} \left( {\widehat{\varvec{\theta }}} \right)$ denote the empirical influence of subject $i$ in stratum $j$ on $\widehat{\varvec{\theta }}$ from the set $\left\{ {\widehat{\varvec{\beta }}, {\text{d}}\widehat{\Lambda }_{0} \left( t \right), \widehat{\Lambda }_{0}^{ } \left( t \right),{ }\widehat{\pi }\left( {\tau_{1} ,\tau_{2} ;{\varvec{x}}} \right)} \right\}$, $i \in \left\{ {1, \ldots ,n_{ }^{\left( j \right)} } \right\}, j \in \left\{ {1, \ldots ,J} \right\}$. From these influences, we can estimate the covariance matrix of $\widehat{\varvec{\theta }}$ as (Graubard and Fears 2005)

8 $${\text{var}}\left( {\widehat{\varvec{\theta }}} \right) \approx {\text{var}}\left\{ {\mathop \sum \limits_{j = 1}^{J} \mathop \sum \limits_{i = 1}^{{n_{ }^{\left( j \right)} }} {{\varvec{\Delta}}}_{i,j} \left( {\widehat{\varvec{\theta }}} \right)} \right\}.$$

Following Graubard and Fears (2005) and Section 4.6 in Pfeiffer and Gail (2017), we show in Web Appendix B.1 that ${{\varvec{\Delta}}}_{i,j} \left( {\widehat{\varvec{\theta }}} \right) = \xi_{i,j} w_{i,j} {\varvec{IF}}_{i,j}^{\left( 2 \right)} \left( {\widehat{\varvec{\theta }}} \right)$, where

9 $$\begin{aligned} {\varvec{IF}}_{i,j}^{\left( 2 \right)} \left( {\widehat{\varvec{\beta }}} \right) & = \left[ {\mathop \sum \limits_{l = 1}^{J} \mathop \sum \limits_{k = 1}^{{n_{ }^{\left( l \right)} }} \mathop \int \limits_{t} \left\{ {\frac{{ {\varvec{S}}_{2} \left( {t;\widehat{\varvec{\beta }}} \right)}}{{S_{0} \left( {t;\widehat{\varvec{\beta }}} \right)}} - \frac{{ {\varvec{S}}_{1} \left( {t;\widehat{\varvec{\beta }}} \right) {\varvec{S}}_{1} \left( {t;\widehat{\varvec{\beta }}} \right)^{\prime} }}{{S_{0} \left( {t;\widehat{\varvec{\beta }}} \right)^{2} }}} \right\}{\text{d}}N_{k,l} \left( t \right)} \right]^{ - 1} \left[ {\mathop {\int \limits_{t} \left\{ {{\varvec{X}}_{i,j} - \frac{{ {\varvec{S}}_{1} \left( {t;\widehat{\varvec{\beta }}} \right)}}{{ S_{0} \left( {t;\widehat{\varvec{\beta }}} \right)}}} \right\}} } \right. \\ & \quad \left. {\left\{ {{\text{d}}N_{i,j} \left( t \right) - \frac{{Y_{i,j} \left( t \right)\exp \left( {\widehat{\varvec{\beta }}^{\prime} {\varvec{X}}_{i,j} } \right) \mathop \sum \nolimits_{l = 1}^{J} \mathop \sum \nolimits_{k = 1}^{{n_{ }^{\left( l \right)} }} {\text{d}}N_{k,l} \left( t \right)}}{{S_{0} \left( {t;\widehat{\varvec{\beta }}} \right)}}} \right\}} \right], \\ \end{aligned}$$

10 $$\begin{aligned} IF_{i,j}^{\left( 2 \right)} \left\{ {{\text{d}}\widehat{\Lambda }_{0} \left( t \right)} \right\} & = \left\{ {S_{0} \left( {t;\widehat{\varvec{\beta }}} \right)} \right\}^{ - 1} \left\{ {{\text{d}}N_{i,j} \left( t \right) - {\text{d}}\widehat{\Lambda }_{0} \left( t \right)Y_{i,j} \left( t \right)\exp \left( {\widehat{\varvec{\beta }}^{\prime} {\varvec{X}}_{i,j} } \right)} \right\} \\ & \quad - \left\{ {S_{0} \left( {t;\widehat{\varvec{\beta }}} \right)} \right\}^{ - 1} {\text{d}}\widehat{\Lambda }_{0} \left( t \right){\varvec{S}}_{1} \left( {t;\widehat{\varvec{\beta }}} \right)^{\prime} {\varvec{IF}}_{i,j}^{\left( 2 \right)} \left( {\widehat{\varvec{\beta }}} \right), \\ \end{aligned}$$

11 $$IF_{i,j}^{\left( 2 \right)} \left\{ {\mathop \int \limits_{{\tau_{1} }}^{{\tau_{2} }} {\text{d}}\widehat{\Lambda }_{0} \left( t \right)} \right\} = \mathop \int \limits_{{\tau_{1} }}^{{\tau_{2} }} IF_{i,j}^{\left( 2 \right)} \left\{ {{\text{d}}\widehat{\Lambda }_{0} \left( t \right)} \right\},$$

and

12 $$\begin{aligned} IF_{i,j}^{\left( 2 \right)} \left\{ {\widehat{\pi }\left( {\tau_{1} ,\tau_{2} ;{\varvec{x}}} \right)} \right\} & = \left\{ {\frac{{\partial \widehat{\pi }\left( {\tau_{1} ,\tau_{2} ; {\varvec{x}}} \right)}}{{\partial {\varvec{\beta}}}}_{{|{\varvec{\beta}} = \widehat{\varvec{\beta }}}} } \right\}{\varvec{IF}}_{i,j}^{\left( 2 \right)} \left( {\widehat{\varvec{\beta }}} \right)\\ & \quad + \left[ {\frac{{\partial \widehat{\pi }\left( {\tau_{1} ,\tau_{2} ; {\varvec{x}}} \right)}}{{\partial \left\{ {\mathop \int \nolimits_{{\tau_{1} }}^{{\tau_{2} }} {\text{d}}\Lambda_{0} \left( t \right)} \right\}}}_{{|{\text{d}}\Lambda _{0} \left( t \right) = {\text{d}}{\widehat{\Lambda }}_{0}^{ } \left( t \right)}} } \right] IF_{i,j}^{\left( 2 \right)} \left\{ {\mathop \int \limits_{{\tau_{1} }}^{{\tau_{2} }} {\text{d}}\widehat{\Lambda }_{0} \left( t \right)} \right\}. \end{aligned}$$

Equations (9)–(12) depend on “phase-two covariates”. Hence, we use the superscript 2 in ${\varvec{IF}}_{i,j}^{\left( 2 \right)} \left( {\widehat{\varvec{\theta }}} \right)$.

### Variance decomposition and estimation from influence functions

The variance ${\text{var}}\left( {{\widehat{\varvec{\theta }}}} \right) \approx {\text{var}}\left\{ {\sum\nolimits_{{{{j}} = 1}}^{{{J}}} {\mathop \sum \nolimits_{{{i} = 1}}^{{{{n}}_{ }^{\left( {j} \right)} }} } {{\varvec{\Delta}}}_{{{i},{j}}} \left( {{\widehat{\varvec{\theta }}}} \right)} \right\}{ }$ can be decomposed as

13 $${\text{var}}\left[ {{\text{E}}\left\{ {\mathop \sum \limits_{j = 1}^{J} \mathop \sum \limits_{i = 1}^{{n_{ }^{\left( j \right)} }} {{\varvec{\Delta}}}_{i,j} \left( {\widehat{\varvec{\theta }}} \right)|{\varvec{C}}_{1} } \right\}} \right] + {\text{E}}\left[ {{\text{var}}\left\{ {\mathop \sum \limits_{j = 1}^{J} \mathop \sum \limits_{i = 1}^{{n_{ }^{\left( j \right)} }} {{\varvec{\Delta}}}_{i,j} \left( {\widehat{\varvec{\theta }}} \right)|{\varvec{C}}_{1} } \right\}} \right],$$

where ${\varvec{C}}_{1}$ denotes the information from the whole cohort. The first component accounts for sampling the cohort from the “superpopulation” (phase-one component of variance), whereas the second component accounts for sampling the subcohort from the cohort (phase-two component of variance).

We let $w_{i,k,j}$ and $\sigma_{i,k,j}$ denote ${\text{E}}\left( {\xi_{i,j} \xi_{k,j} |{\varvec{C}}_{1} } \right)^{ - 1}$ and ${\text{cov}}\left( {\xi_{i,j} ,\xi_{k,j} |{\varvec{C}}_{1} } \right)$, respectively, $i,k \in \left\{ {1, \ldots ,n_{ }^{\left( j \right)} } \right\}$, $j \in \left\{ {1, \ldots ,J} \right\}$; they are specified below. We know $w_{i,j} {\varvec{IF}}_{i,j}^{\left( 2 \right)} \left( {\widehat{\varvec{\theta }}} \right)$ is fixed conditional on ${\varvec{C}}_{1}$ and ${\text{E}}\left( {\xi_{i,j} w_{i,j} |{\varvec{C}}_{1} } \right) = 1$. Thus ${\text{var}}\left\{ {\sum\nolimits_{j = 1}^{J} {\mathop \sum \nolimits_{i = 1}^{{n_{ }^{\left( j \right)} }} } {{\varvec{\Delta}}}_{i,j} \left( {\widehat{\varvec{\theta }}} \right)} \right\} = {\text{var}}\left\{ {\sum\nolimits_{j = 1}^{J} {\mathop \sum \nolimits_{i = 1}^{{n_{ }^{\left( j \right)} }} } {\varvec{IF}}_{i,j}^{\left( 2 \right)} \left( {\widehat{\varvec{\theta }}} \right)} \right\} + {\text{E}}\left\{ {\sum\nolimits_{j = 1}^{J} {\sum\nolimits_{i = 1}^{{n_{ }^{\left( j \right)} }} {\mathop \sum \nolimits_{k = 1}^{{n_{ }^{\left( j \right)} }} } } \sigma_{i,k,j}^{ } w_{i,j} w_{k,j} {\varvec{IF}}_{i,j}^{\left( 2 \right)} \left( {\widehat{\varvec{\theta }}} \right){\varvec{IF}}_{k,j}^{\left( 2 \right)} \left( {\widehat{\varvec{\theta }}} \right)^{\prime} } \right\}$. Because ${\varvec{IF}}_{i,j}^{\left( 2 \right)} \left( {\widehat{\varvec{\theta }}} \right){\varvec{IF}}_{i,j}^{\left( 2 \right)} \left( {\widehat{\varvec{\theta }}} \right)^{\prime}$ and ${\varvec{IF}}_{i,j}^{\left( 2 \right)} \left( {\widehat{\varvec{\theta }}} \right){\varvec{IF}}_{k,j}^{\left( 2 \right)} \left( {\widehat{\varvec{\theta }}} \right)^{\prime}$ can only be computed if individuals $i$ and $k$ in stratum $j$ are in the phase-two sample, we weight the contributions from the individuals in the phase-two sample by the “marginal” and “joint” design weights, $w_{i,j}$ and $w_{i,k,j}$, to estimate ${\text{var}}\left( {\widehat{\varvec{\theta }}} \right)$ by

14 $$\frac{n}{n - 1}\mathop \sum \limits_{j = 1}^{J} \mathop \sum \limits_{i = 1}^{{n_{ }^{\left( j \right)} }} \xi_{i,j} w_{i,j} \varvec{ IF}_{i,j}^{\left( 2 \right)} \left( {\widehat{\varvec{\theta }}} \right){\varvec{IF}}_{i,j}^{\left( 2 \right)} \left( {\widehat{\varvec{\theta }}} \right)^{\prime} + \mathop \sum \limits_{j = 1}^{J} \mathop \sum \limits_{i = 1}^{{n_{ }^{\left( j \right)} }} \mathop \sum \limits_{k = 1}^{{n_{ }^{\left( j \right)} }} w_{i,k,j} \sigma_{i,k,j}^{ } w_{i,j} w_{k,j} \xi_{i,j} \xi_{k,j} {\varvec{IF}}_{i,j}^{\left( 2 \right)} \left( {\widehat{\varvec{\theta }}} \right){\varvec{IF}}_{k,j}^{\left( 2 \right)} \left( {\widehat{\varvec{\theta }}} \right)^{\prime} .$$

Following Barlow (1994), the “robust” variance estimate would be

15 $$\mathop \sum \limits_{j = 1}^{J} \mathop \sum \limits_{i = 1}^{{n_{ }^{\left( j \right)} }} {{\varvec{\Delta}}}_{i,j} \left( {\widehat{\varvec{\theta }}} \right){{\varvec{\Delta}}}_{i,j} \left( {\widehat{\varvec{\theta }}} \right)^{\prime} = \mathop \sum \limits_{j = 1}^{J} \mathop \sum \limits_{i = 1}^{{n_{ }^{\left( j \right)} }} \xi_{i,j} w_{i,j} \varvec{ }w_{i,j} \varvec{ IF}_{i,j}^{\left( 2 \right)} \left( {\widehat{\varvec{\theta }}} \right){\varvec{IF}}_{i,j}^{\left( 2 \right)} \left( {\widehat{\varvec{\theta }}} \right)^{\prime} .$$

With stratified data, Eq. (15) is often too large (see also Sect. 7 and Web Appendix D.2). Equation (15) minus Eq. (14) is

16 $$\frac{1}{n - 1}\mathop \sum \limits_{j = 1}^{J} \mathop \sum \limits_{i = 1}^{{n_{ }^{\left( j \right)} }} \xi_{i,j} w_{i,j} {\varvec{IF}}_{i,j}^{\left( 2 \right)} \left( {\widehat{\varvec{\theta }}} \right){\varvec{IF}}_{i,j}^{\left( 2 \right)} \left( {\widehat{\varvec{\theta }}} \right)^{\prime} + \mathop \sum \limits_{j = 1}^{J} \mathop \sum \limits_{i = 1}^{{n_{ }^{\left( j \right)} }} \mathop {\sum \limits_{k = 1}}_{k \ne i}^{{n_{ }^{\left( j \right)} }} w_{i,k,j} \sigma_{i,k,j}^{ } w_{i,j} w_{k,j} \xi_{i,j} \xi_{k,j} {\varvec{IF}}_{i,j}^{\left( 2 \right)} \left( {\widehat{\varvec{\theta }}} \right){\varvec{IF}}_{k,j}^{\left( 2 \right)} \left( {\widehat{\varvec{\theta }}} \right)^{\prime} .$$

Because we sample *without replacement* in each stratum, we have $w_{i,k,j} = \frac{{n_{ }^{\left( j \right)} \left( {n_{ }^{\left( j \right)} - 1} \right)}}{{m_{ }^{\left( j \right)} \left( {m_{ }^{\left( j \right)} - 1} \right)}}$ if individuals $i$ and $k$ in stratum $j$ are both non-cases, and $w_{i,k,j} = w_{i,j} \times w_{k,j}$ otherwise, $i,k \in \left\{ {1, \ldots ,n_{ }^{\left( j \right)} } \right\}$, $k \ne i$, $j \in \left\{ {1, \ldots ,J} \right\}$. Recall that $w_{i,i,j} = w_{i,j} = \frac{{n_{ }^{\left( j \right)} }}{{m_{ }^{\left( j \right)} }}$ if individual $i$ in stratum $j$ is a non-case, and $w_{i,j} = 1$ if individual $i$ in stratum $j$ is a case. Then $\sigma_{i,k,j} = \frac{{m_{ }^{\left( j \right)} }}{{n_{ }^{\left( j \right)} }}\frac{{m_{ }^{\left( j \right)} - 1}}{{n_{ }^{\left( j \right)} - 1}} - \left( {\frac{{m_{ }^{\left( j \right)} }}{{n_{ }^{\left( j \right)} }}} \right)^{2}$ if individuals $i$ and $k$ in stratum $j$ are both non-cases, and $\sigma_{i,k,j} = 0$ otherwise, $i,k \in \left\{ {1, \ldots ,n_{ }^{\left( j \right)} } \right\}$, $k \ne i$, $j \in \left\{ {1, \ldots ,J} \right\}$. Similarly, if individual $i$ in stratum $j$ is a non-case, then $\sigma_{i,i,j} \equiv \sigma_{i,j} = \frac{{m_{ }^{\left( j \right)} }}{{n_{ }^{\left( j \right)} }}\left( {1 - \frac{{m_{ }^{\left( j \right)} }}{{n_{ }^{\left( j \right)} }}} \right)$, and $\sigma_{i,j} = 0$ otherwise. As a result, only the sampled non-cases contribute to the phase-two component of the variance in Eq. (14). For sampling *with replacement* (i.e., Bernoulli sampling), individuals are sampled independently of each other. Then $w_{i,k,j} = w_{i,j} \times w_{k,j}$, and $\sigma_{i,k,j} = 0$ for any pair $\left( {i,k} \right)$ of distinct individuals in stratum $j$, $i,k \in \left\{ {1, \ldots ,n_{ }^{\left( j \right)} } \right\}$, $k \ne i$, $j \in \left\{ {1, \ldots ,J} \right\}$. In that case, the difference between the “robust” variance estimate in Eqs. (15) and (14) reduces to $\frac{1}{n - 1}\sum\nolimits_{j = 1}^{J} {\mathop \sum \nolimits_{i = 1}^{{n_{ }^{\left( j \right)} }} } \xi_{i,j} w_{i,j} {\varvec{IF}}_{i,j}^{\left( 2 \right)} \left( {\widehat{\varvec{\theta }}} \right){\varvec{IF}}_{i,j}^{\left( 2 \right)} \left( {\widehat{\varvec{\theta }}} \right)^{\prime}$, which is negligible compared to Eq. (14) in large cohorts.

The variance estimate in Eq. (14) is asymptotically equivalent to that of Lin (2000) for $\widehat{\varvec{\beta }}$ and $\widehat{\Lambda }_{0} \left( t \right)$ (Lin did not consider covariate-specific pure risks). The second component in Eq. (14) is precisely equal to the terms Lin (2000) used to estimate the phase-two component of the variance for $\widehat{\varvec{\beta }}$ and $\widehat{\Lambda }_{0} \left( t \right)$. To estimate the phase-one component of variance of $\widehat{\varvec{\beta }}$, Lin (2000) used the inverse of the observed information matrix for ${\varvec{U}}\left( {\varvec{\beta}} \right)$, whereas the weighted sum of ${\varvec{IF}}_{i,j}^{\left( 2 \right)} \left( {\widehat{\varvec{\beta }}} \right){\varvec{IF}}_{i,j}^{\left( 2 \right)} \left( {\widehat{\varvec{\beta }}} \right)^{\prime}$ in the first component in Eq. (14) is a “sandwich estimate” of this quantity that is consistent for it. In addition, relying on the influences to estimate the phase-one component of the variance allows for an easy extension to other designs and analytic options (e.g., using calibrated weights). The estimated phase-one component of variance of $\widehat{\Lambda }_{0} \left( t \right)$ on page 43 of Lin (2000) consists of two parts, as in Andersen and Gill (1982), that correspond to the two terms in $IF_{i,j}^{\left( 2 \right)} \left\{ {\mathop \int _{{\tau_{1} }}^{{\tau_{2} }} {\text{d}}\widehat{\Lambda }_{0} \left( t \right)} \right\}$ obtained from Eqs. (10) and (11). The conditional expectation of the second term is zero given the phase-one data, proving that the two components are uncorrelated. The weighted sum of the first term squared equals that in Lin (2000). The weighted sum of the cross-products of the second term is a sandwich estimate of the second quantity estimated in Lin (2000), who instead used the observed information matrix for ${\varvec{U}}\left( {\varvec{\beta}} \right)$ in the calulation. See also Web Appendix B.2 for comparison with the estimate of ${\text{var}}\left( {\widehat{\varvec{\beta }}} \right)$ by Samuelsen et al. (2007). We note that our influence function-based variance estimates performed well in simulations (see Sect. 7).

### Asymptotic normality

We assume that normed estimates of $\widehat{\varvec{\beta }}$, $\widehat{\Lambda }_{0}^{ } \left( t \right)$ and $\widehat{\pi }\left( {\tau_{1} ,\tau_{2} ;{\varvec{x}}} \right)$ are normally distributed for fixed $t$, $\tau_{1}$ and $\tau_{2}$. This assumption is supported by nominal coverage of confidence intervals in our simulations. From finite sampling theory, Borgan et al. (2000) and Lin (2000) argued that certain phase-two normed sums were asymptotically normally distributed conditional on the phase-one data with covariances that did not depend on the phase-one data, implying that $n^{\frac{1}{2}} \left( {\widehat{\varvec{\beta }} - {\varvec{\beta}}} \right)$ was asymptotically Normal unconditionally. Assuming an additional tightness condition, Lin (2000) proved that $n^{\frac{1}{2}} \left\{ {{\widehat{\Lambda }}_{0} \left( t \right) - {\Lambda }_{0} \left( t \right)} \right\}$ converged to a Gaussian process. Hence $n^{1/2} \left\{ {\widehat{\pi }\left( {\tau_{1} ,\tau_{2} ;{\varvec{x}}} \right) - \pi \left( {\tau_{1} ,\tau_{2} ;{\varvec{x}}} \right)} \right\}$ converges to normality for fixed $\tau_{1}$ and $\tau_{2}$. The tightness condition was not proved but thought to hold for stratified designs.

## Calibration of the design weights

### Calibration and choice of auxiliary variables

Breslow et al. (2009a, 2009b) advocated “weight calibration” to improve the efficiency of case-cohort studies. First, one identifies auxiliary variables that are highly correlated with the influences on $\widehat{\varvec{\theta }}$ and are known for the entire cohort. Then one perturbs the design weights to obtain calibrated weights that are close to the design weights but for which the observed sums of auxiliary variables in the phase-one sample equals the weighted sums in the phase-two sample with the calibrated weights. To obtain auxiliary variables, we follow Shin et al. (2020). First, we use weighted regression in the phase-two sample to estimate the expected value of phase-two covariates given phase-one data. These expectations are used to impute the phase-two covariates for all members of the cohort, including those with measured phase-two covariates. The phase-one data used for imputation may consist of covariates in ${\varvec{X}}$ and of phase-one proxies of the phase-two covariates that are measured on all cohort members. The auxiliary variables are (i) the influences for the log-relative hazard parameters estimated from the Cox model with imputed cohort data; and (ii) the products of follow-up time on the interval for which pure risk is to be estimated times the estimated relative hazard for the imputed cohort data, where the log-relative hazard parameters are estimated from the Cox model with case-cohort data and weights calibrated with (i). To standardize the weights, we also calibrate against (iii) a variable that is identically equal to 1. Calibration of the design weights against (i) alone was proposed by Breslow et al. (2009a, 2009b) to improve efficiency of case-cohort estimates of log-relative hazard. Shin et al. (2020) extended the work of Breslow et al. (2009a, 2009b) and proposed calibrating against (i) + (ii) + (iii) to improve efficiency of log-relative hazard and pure risk estimates under the nested case–control design. Additional details are in Web Appendix C.1; see also Breslow et al. (2009a) and Shin et al. (2020). Other auxiliary variables have been proposed for $\widehat{\Lambda }_{0} \left( t \right)$ (Breslow and Lumley 2013), but in unreported simulations, the proposal by Shin et al. performed better; see also Web Appendix C.3.

We let ${\varvec{A}}_{i,j}$ be the vector of $q$ auxiliary variables for individual $i$ in stratum $j$, with calibrated weights $w_{i,j}^{*} = w_{i,j} {\text{ exp}}\left( {\widehat{\varvec{\eta }}^{\prime}{\varvec{A}}_{i,j} } \right)$, $i \in \left\{ {1, \ldots ,n_{ }^{\left( j \right)} } \right\}$, $j \in \left\{ {1, \ldots ,J} \right\}$, that are obtained by solving $\sum\nolimits_{j = 1}^{J} {\mathop \sum \nolimits_{i = 1}^{{n^{\left( j \right)} }} } \left\{ {\xi_{i,j} w_{i,j} \exp \left( {\varvec{\eta}^{\prime}\varvec{A}_{i,j} } \right){\varvec{A}}_{i,j} - {\varvec{A}}_{i,j} } \right\} = 0$ for $\widehat{\varvec{\eta }}$. See Web Appendix C.1.

### Estimation of relative hazard, cumulative baseline hazard and pure risk using calibrated weights

An estimate of ${\varvec{\beta}}$ solves ${\varvec{U}}^{\varvec{*}} \left( {\varvec{\beta}} \right) = \mathop \sum\nolimits_{j = 1}^{J} \mathop \sum\nolimits_{i = 1}^{{n_{ }^{\left( j \right)} }} \mathop \int_{t} \xi_{i,j} w_{i,j}^{*} \left\{ {{\varvec{X}}_{i,j} - \frac{{{\varvec{S}}_{1}^{*} \left( {t;\widehat{\varvec{\eta }},{\varvec{\beta}}} \right)}}{{S_{0}^{*} \left( {t;\widehat{\varvec{\eta }},{\varvec{\beta}}} \right)}}} \right\}{\text{d}}N_{i,j} \left( t \right) = 0$, where $S_{0}^{*} \left( {t;\widehat{\varvec{\eta }},{\varvec{\beta}}} \right)$, ${\varvec{S}}_{1}^{*} \left( {t;\widehat{\varvec{\eta }},{\varvec{\beta}}} \right)$ and ${\varvec{S}}_{2}^{*} \left( {t;\widehat{\varvec{\eta }},{\varvec{\beta}}} \right)$ are obtained from Eqs. (2)–(4) with $w_{k,j}^{*}$ replacing $w_{k,j}$. Letting $\widehat{\varvec{\beta }}^{*} \left( {\widehat{\varvec{\eta }}} \right) \equiv \widehat{\varvec{\beta }}^{*}$, we estimate the baseline hazard point mass at time $t$, ${\text{d}}\widehat{\Lambda }_{0}^{*} \left( {t;\widehat{\varvec{\eta }},\widehat{\varvec{\beta }}^{*} } \right) \equiv {\text{d}}\widehat{\Lambda }_{0}^{*} \left( t \right)$, the cumulative baseline hazard up to time $t$, $\widehat{\Lambda }_{0}^{*} \left( {t;\widehat{\varvec{\eta }},\widehat{\varvec{\beta }}^{*} } \right) \equiv \widehat{\Lambda }_{0}^{*} \left( t \right)$, and the pure risk for profile ${\varvec{x}}$ in the interval $(\tau_{1} ,\tau_{2} ]$, $\widehat{\pi }^{*} \left( {\tau_{1} ,\tau_{2} ;{\varvec{x}},\widehat{\varvec{\eta }},\widehat{\varvec{\beta }}^{*} , {\text{d}}\widehat{\Lambda }_{0}^{*} } \right) \equiv \widehat{\pi }^{*} \left( {\tau_{1} ,\tau_{2} ;{\varvec{x}}} \right)$, from Eqs. (5)–(7) with $S_{0}^{*} \left( {t;\widehat{\varvec{\eta }},\widehat{\varvec{\beta }}^{*} } \right)$ and $\widehat{\varvec{\beta }}^{*}$ replacing $S_{0} \left( {t,\widehat{\varvec{\beta }}} \right)$ and $\widehat{\varvec{\beta }}.$ We do not calibrate the case weights in the numerator of the Breslow estimator because the event times are known for all cohort members (Breslow and Wellner 2007; Pugh et al. 1993; Shin et al. 2020).

### Variance estimation from influence functions

We let ${{\varvec{\Delta}}}_{i,j} \left( {\widehat{\varvec{\theta }}^{*} } \right)$ denote the influence of individual $i$ in stratum $j$ on one of the parameters $\widehat{\varvec{\theta }}^{*}$ from the set $\left\{ {\widehat{\varvec{\eta }}},{\widehat{\varvec{\beta }}}^{*}, {\text{d}}{\widehat{\Lambda }}_{0}^{*} \left( t \right),{\widehat{\Lambda }}_{0}^{*} \left( t \right), \widehat{\pi}^{*} \left(\tau_{1},\tau_{2} ;{\varvec{x}} \right) \right\}$, $i \in \left\{ {1, \ldots ,n_{ }^{\left( j \right)} } \right\}, \;j \in \left\{ {1, \ldots ,J} \right\}$, and use ${\text{var}}\left( {\widehat{\varvec{\theta }}^{*} } \right) \approx {\text{var}}\left\{ {\sum\nolimits_{j = 1}^{J} {\mathop \sum \nolimits_{i = 1}^{{n_{ }^{\left( j \right)} }} } {{\varvec{\Delta}}}_{i,j} \left( {\widehat{\varvec{\theta }}^{*} } \right)} \right\}.$ Following Shin et al. (2020) we can show that ${{\varvec{\Delta}}}_{i,j} \left( {\widehat{\varvec{\theta }}^{*} } \right) = {\varvec{IF}}_{i,j}^{\left( 1 \right)} \left( {\widehat{\varvec{\theta }}^{*} } \right) + \xi_{i,j} w_{i,j} {\varvec{IF}}_{i,j}^{\left( 2 \right)} \left( {\widehat{\varvec{\theta }}^{*} } \right)$. The superscript 1 indicates that ${\varvec{IF}}_{i,j}^{\left( 1 \right)} \left( {\widehat{\varvec{\theta }}^{*} } \right)$ depends only on variables measured on all cohort members. If individual $i$ in stratum $j$ is not in the phase-two sample, $\xi_{i,j} w_{i,j} {\varvec{IF}}_{i,j}^{\left( 2 \right)} \left( {\widehat{\varvec{\theta }}^{*} } \right)$ is zero, but such an individual has an influence on $\hat{\varvec{\eta }}$ and hence on $\hat{\varvec{\theta }}^{*}$ through ${\varvec{IF}}_{i,j}^{\left( 1 \right)} \left( {\widehat{\varvec{\theta }}^{*} } \right)$. Explicit forms of ${\varvec{IF}}_{i,j}^{\left( s \right)} \left( {\widehat{\varvec{\theta }}^{*} } \right)$, $s \in \left\{ {1,2} \right\}$, are in Appendix 1 and derived in Web Appendix C.2.

Because ${\varvec{IF}}_{i,j}^{\left( 1 \right)} \left( {\widehat{\varvec{\theta }}} \right)$ is fixed conditional on ${\varvec{C}}_{1}$, a decomposition similar to Eq. (13) yields

17 $${\text{var}}\left\{ {\mathop \sum \limits_{j = 1}^{J} \mathop \sum \limits_{i = 1}^{{n_{ }^{\left( j \right)} }} {{\varvec{\Delta}}}_{i,j} \left( {\widehat{\varvec{\theta }}^{*} } \right)} \right\} = {\text{ var}}\left\{ {\mathop \sum \limits_{j = 1}^{J} \mathop \sum \limits_{i = 1}^{{n_{ }^{\left( j \right)} }} {\varvec{IF}}_{i,j}^{\left( 1 \right)} \left( {\widehat{\varvec{\theta }}^{*} } \right) + {\varvec{IF}}_{i,j}^{\left( 2 \right)} \left( {\widehat{\varvec{\theta }}^{*} } \right)} \right\} + {\text{E}}\left\{ {\mathop \sum \limits_{j = 1}^{J} \mathop \sum \limits_{i = 1}^{{n_{ }^{\left( j \right)} }} \mathop \sum \limits_{k = 1}^{{n_{ }^{\left( j \right)} }} \sigma_{i,k,j}^{ } w_{i,j} w_{k,j} {\varvec{IF}}_{i,j}^{\left( 2 \right)} \left( {\widehat{\varvec{\theta }}^{*} } \right){\varvec{IF}}_{k,j}^{\left( 2 \right)} \left( {\widehat{\varvec{\theta }}^{*} } \right)^{\prime} } \right\},$$

which can be estimated by

18 $$\begin{aligned} & \frac{n}{n - 1}\mathop \sum \limits_{j = 1}^{J} \mathop \sum \limits_{i = 1}^{{n_{ }^{\left( j \right)} }} \left\{ {{\varvec{IF}}_{i,j}^{\left( 1 \right)} \left( {\widehat{\varvec{\theta }}^{*} } \right){\varvec{IF}}_{i,j}^{\left( 1 \right)} \left( {\widehat{\varvec{\theta }}^{*} } \right){\prime} + 2 \xi_{i,j} w_{i,j} \varvec{ IF}_{i,j}^{\left( 1 \right)} \left( {\widehat{\varvec{\theta }}^{*} } \right){\varvec{IF}}_{i,j}^{\left( 2 \right)} \left( {\widehat{\varvec{\theta }}^{*} } \right)^{\prime} } \right. \\ & \quad \left. { + \xi_{i,j} w_{i,j} \varvec{ IF}_{i,j}^{\left( 2 \right)} \left( {\widehat{\varvec{\theta }}^{*} } \right){\varvec{IF}}_{i,j}^{\left( 2 \right)} \left( {\widehat{\varvec{\theta }}^{*} } \right)^{\prime} } \right\} \\ & \quad + \mathop \sum \limits_{j = 1}^{J} \mathop \sum \limits_{i = 1}^{{n_{ }^{\left( j \right)} }} \mathop \sum \limits_{k = 1}^{{n_{ }^{\left( j \right)} }} w_{i,k,j} \sigma_{i,k,j}^{ } \xi_{i,j} \xi_{k,j} w_{i,j} w_{k,j} {\varvec{IF}}_{i,j}^{\left( 2 \right)} \left( {\widehat{\varvec{\theta }}^{*} } \right){\varvec{IF}}_{k,j}^{\left( 2 \right)} \left( {\widehat{\varvec{\theta }}^{*} } \right)^{\prime} . \\ \end{aligned}$$

Finally, the robust variance estimate (Barlow 1994) is

19 $$\begin{aligned} & \mathop \sum \limits_{j = 1}^{J} \mathop \sum \limits_{i = 1}^{{n_{ }^{\left( j \right)} }} {{\varvec{\Delta}}}_{i,j} \left( {\widehat{\varvec{\theta }}^{*} } \right){{\varvec{\Delta}}}_{i,j} \left( {\widehat{\varvec{\theta }}^{*} } \right)^{\prime} = \mathop \sum \limits_{j = 1}^{J} \mathop \sum \limits_{i = 1}^{{n_{ }^{\left( j \right)} }} \left\{ {{\varvec{IF}}_{i,j}^{\left( 1 \right)} \left( {\widehat{\varvec{\theta }}^{*} } \right){\varvec{IF}}_{i,j}^{\left( 1 \right)} \left( {\widehat{\varvec{\theta }}^{*} } \right)^{\prime} } \right. \\ & \quad \left. { + 2 \xi_{i,j} w_{i,j} \varvec{ IF}_{i,j}^{\left( 1 \right)} \left( {\widehat{\varvec{\theta }}^{*} } \right){\varvec{IF}}_{i,j}^{\left( 2 \right)} \left( {\widehat{\varvec{\theta }}^{*} } \right)^{\prime} + \xi_{i,j} w_{i,j} w_{i,j} \varvec{ IF}_{i,j}^{\left( 2 \right)} \left( {\widehat{\varvec{\theta }}^{*} } \right){\varvec{IF}}_{i,j}^{\left( 2 \right)} \left( {\widehat{\varvec{\theta }}^{*} } \right)^{\prime} } \right\}, \\ \end{aligned}$$

and the difference between Eqs. (18) and (19) is

20 $$\begin{aligned} & \frac{1}{n - 1}\mathop \sum \limits_{j = 1}^{J} \mathop \sum \limits_{i = 1}^{{n_{ }^{\left( j \right)} }} \left\{ {{\varvec{IF}}_{i,j}^{\left( 1 \right)} \left( {\widehat{\varvec{\theta }}^{*} } \right){\varvec{IF}}_{i,j}^{\left( 1 \right)} \left( {\widehat{\varvec{\theta }}^{*} } \right)^{\prime} + 2 \xi_{i,j} w_{i,j} \varvec{ IF}_{i,j}^{\left( 1 \right)} \left( {\widehat{\varvec{\theta }}^{*} } \right){\varvec{IF}}_{i,j}^{\left( 2 \right)} \left( {\widehat{\varvec{\theta }}^{*} } \right)^{\prime} } \right. \\ & \quad \left. { + \xi_{i,j} w_{i,j} \varvec{ IF}_{i,j}^{\left( 2 \right)} \left( {\widehat{\varvec{\theta }}^{*} } \right){\varvec{IF}}_{i,j}^{\left( 2 \right)} \left( {\widehat{\varvec{\theta }}^{*} } \right)^{\prime} } \right\} \\ & \quad + \mathop \sum \limits_{j = 1}^{J} \mathop \sum \limits_{i = 1}^{{n_{ }^{\left( j \right)} }} \mathop {\sum \limits_{k=1}}_{k \ne i}^{{n_{ }^{\left( j \right)} }} w_{i,k,j} \sigma_{i,k,j}^{ } w_{i,j} w_{k,j } \xi_{i,j} \xi_{k,j} \varvec{ IF}_{i,j}^{\left( 2 \right)} \left( {\widehat{\varvec{\theta }}^{*} } \right){\varvec{IF}}_{k,j}^{\left( 2 \right)} \left( {\widehat{\varvec{\theta }}^{*} } \right)^{\prime} . \\ \end{aligned}$$

For individuals $i$ and $k$ in stratum $j$ such that $\sigma_{i,k,j}^{ }$ and $\sigma_{i,j}^{ }$ are non-zero (i.e., non-cases), $\xi_{i,j} w_{i,j} \varvec{ IF}_{i,j}^{\left( 2 \right)} \left( {\widehat{\varvec{\theta }}^{*} } \right)$ and $\xi_{k,j} w_{k,j} {\varvec{IF}}_{k,j}^{\left( 2 \right)} \left( {\widehat{\varvec{\theta }}^{*} } \right)$ are weighted residuals from a weighted linear regression on the auxiliary variables; see Web Appendix C.3. With good auxiliary variables for calibration, one can expect the phase-two component of the variance and hence the total variance to be smaller, and the difference in Eq. (20) to be smaller than the difference in Eq. (16); see also Chapter 17 in Borgan et al. (2017).

See Web Appendix I for a summary of the steps for parameter and variance estimation with calibrated weights.

## Missing data

### Notation

Covariate information may be missing for individuals in phase-two. For example, stored blood samples from individuals in phase-two could have been previously used or lost. We assume such covariates are missing at random and we regard the set of individuals with complete covariate data as a *phase-three sample*. More precisely, let $V_{i,j}$ be the phase-three sampling indicator for subject $i$ in stratum $j$, $i \in \left\{ {1, \ldots ,n_{ }^{\left( j \right)} } \right\}$, $j \in \left\{ {1, \ldots ,J} \right\}$; we assume the Bernoulli indicators $V_{i,j}$ are mutually independent and independent of the phase-two indicators, $\xi_{i,j}$. Let $w_{i,j}^{\left( 3 \right)} \equiv \frac{1}{{\pi_{i,j}^{\left( 3 \right)} }}$ be the phase-three design weight, where $\pi_{i,j}^{\left( 3 \right)}$ is the phase-three design sampling probability. The overall sampling design weight of subject $i$ in stratum $j$ is $w_{i,j} = w_{i,j}^{\left( 2 \right)} \times w_{i,j}^{\left( 3 \right)}$.

The phase-three sampling probabilities may differ in $J^{\left( 3 \right)}$ exclusive and exhaustive subsets (phase-three strata) of the population that need not coincide with the $J$ phase-two strata. For example, cases may have a different probability of missingness from non-cases. Nonetheless, we index the members of the cohort as in Sect. 3. Web Appendix F describes analysis when the phase-three sampling probabilities are known. However, the $\pi_{i,j}^{\left( 3 \right)}$ are usually unknown and need to be estimated (Sect. 5.2).

### Weight estimation

When the $\pi_{i,j}^{\left( 3 \right)}$ are unknown, $w_{i,j}^{\left( 3 \right)}$ can be estimated as follows, $i \in \left\{ {1, \ldots ,n_{ }^{\left( j \right)} } \right\}$, $j \in \left\{ {1, \ldots ,J} \right\}$. The $V_{i,j}$ are known for all members of the phase-two sample, and let ${\varvec{B}}_{i,j}$ be a $J^{\left( 3 \right)} \times 1$ vector of indicator variables that take value 1 if subject $i$ in (phase-two) stratum $j$ is in the corresponding phase-three stratum, and 0 otherwise. Let $\exp \left( {\widetilde{\varvec{\gamma }}} \right)$ be the vector of $J^{\left( 3 \right)}$ estimated phase-three sampling weights that are obtained by solving the estimating equation $\sum\nolimits_{j = 1}^{J} {\mathop \sum \nolimits_{i = 1}^{{n_{ }^{\left( j \right)} }} } \xi_{i,j} {\varvec{B}}_{i,j} - \exp \left( {\varvec{\gamma}^{\prime}\varvec{B}_{i,j} } \right) \xi_{i,j} V_{i,j} {\varvec{B}}_{i,j} = 0$. For example, if phase-three sampling is stratified on case status, we use weights $$\tilde{w}_{i,j}^{\left( 3 \right)} = \frac{{\mathop \sum \nolimits_{l = 1}^{J} \mathop \sum \nolimits_{{\begin{subarray}{*{20}l} {k = 1,} \hfill \\ {non\, case} \hfill \\ \end{subarray} }}^{{n_{ }^{\left( j \right)} }} \xi_{k,l} }}{{\mathop \sum \nolimits_{l = 1}^{J} \mathop \sum \nolimits_{{\begin{subarray}{*{20}l} {k = 1,} \hfill \\ {non \,case} \hfill \\ \end{subarray} }}^{{n_{ }^{\left( j \right)} }} \xi_{k,l} V_{k,l} }}$$﻿ if subject $i$ in stratum $j$ is a non-case, and $$\tilde{w}_{i,j}^{\left( 3 \right)} = \frac{{\mathop \sum \nolimits_{l = 1}^{J} \mathop \sum \nolimits_{{\begin{subarray}{*{20}l} {k = 1,} \\ {case} \\ \end{subarray} }}^{{n_{ }^{\left( j \right)} }} \xi_{k,l} }}{{\mathop \sum \nolimits_{l = 1}^{J} \mathop \sum \nolimits_{{\begin{subarray}{*{20}l} {k = 1,} \\ {case} \\ \end{subarray} }}^{{n_{ }^{\left( j \right)} }} \xi_{k,l} V_{k,l} }}$$﻿ if subject $i$ in stratum $j$ is a case. Finally, we estimate ${\text{var}}\left( {V_{i,j} } \right) \equiv \sigma_{i,j}^{\left( 3 \right)}$ by $\widetilde{\sigma }_{i,j}^{\left( 3 \right)} = \frac{1}{{\widetilde{w}_{i,j}^{\left( 3 \right)} }}\left( {1 - \frac{1}{{\widetilde{w}_{i,j}^{\left( 3 \right)} }}} \right)$.

### Estimation of relative hazard, cumulative baseline hazard and pure risk

We obtain the log-relative hazard estimate $$\widetilde{\varvec{\beta }}\left( {\widetilde{\varvec{\gamma }}} \right) \equiv \widetilde{\varvec{\beta }}$$ from solving for $${\varvec{\beta}}$$ in the estimating equation $$\sum\nolimits_{j = 1}^{J} {\mathop \sum \nolimits_{i = 1}^{{n_{ }^{\left( j \right)} }} } \mathop \int_{t} V_{i,j} \widetilde{w}_{i,j}^{\left( 3 \right)} \left\{ {{\varvec{X}}_{i,j} - \frac{{\widetilde{\varvec{S}}_{1} \left( {t;\widetilde{\varvec{\gamma }},{\varvec{\beta}}} \right)}}{{\widetilde{S}_{0} \left( {t;\widetilde{\varvec{\gamma }},{\varvec{\beta}}} \right)}}} \right\}{\text{d}}N_{i,j} \left( t \right) = 0$$. We let $$\widetilde{w}_{k,j} = w_{i,j}^{\left( 2 \right)} \times \widetilde{w}_{i,j}^{\left( 3 \right)}$$ and compute $$\widetilde{S}_{0} \left( {t;\widetilde{\varvec{\gamma }},{\varvec{\beta}}} \right)$$, $$\widetilde{\varvec{S}}_{1} \left( {t;\widetilde{\varvec{\gamma }},{\varvec{\beta}}} \right)$$ and $$\widetilde{\varvec{S}}_{2} \left( {t;\widetilde{\varvec{\gamma }},{\varvec{\beta}}} \right)$$ from Eqs. (2)–(4) by substituting $$\xi_{k,j} V_{k,j}$$ for $$\xi_{k,j}$$ and $$\widetilde{w}_{k,j}$$ for $$w_{k,j.}$$ We estimate the baseline hazard point mass at time $$t$$, $${\text{d}}\widetilde{\Lambda }_{0} \left( {t;\widetilde{\varvec{\gamma }},\widetilde{\varvec{\beta }}} \right) \equiv {\text{d}}\widetilde{\Lambda }_{0} \left( t \right)$$, the cumulative baseline hazard up to time $$t$$, $$\widetilde{\Lambda }_{0} \left( {t;\widetilde{\varvec{\gamma }},\widetilde{\varvec{\beta }}} \right) \equiv \widetilde{\Lambda }_{0} \left( t \right)$$, and the pure risk for profile $${\varvec{x}}$$ in the interval $$(\tau_{1} ,\tau_{2} ]$$, $$\widetilde{\pi }\left( {\tau_{1} ,\tau_{2} ;{\varvec{x}},\widetilde{\varvec{\gamma }},\widetilde{\varvec{\beta }}, 
{\text{d}}{\widetilde{\Lambda }}_{0} } \right) \equiv \widetilde{\pi }\left( {\tau_{1} ,\tau_{2} ;{\varvec{x}}} \right)$$, from Eqs. (5)–(7) with $$\widetilde{S}_{0} \left( {t;\widetilde{\varvec{\gamma }},{\widetilde{\varvec{\beta}}}} \right)$$ and $$\widetilde{\varvec{\beta }}$$ replacing $$S_{0} \left( {t,\widehat{\varvec{\beta }}} \right)$$ and $$\widehat{\varvec{\beta }}.$$

If a case with missing phase-two data occurs at a time $t$ when no other member of the phase-three sample is at risk, the Breslow estimate of cumulative baseline hazard is undefined. One option is to restrict the risk projection interval by increasing $\tau_{1}$ or decreasing $\tau_{2}$ to avoid such times. If there are only a small number of such times, we recommend ignoring them in all calculations.

### Influence functions

Let ${{\varvec{\Delta}}}_{i,j} \left( {\widetilde{\varvec{\theta }}} \right)$ denote the influence of subject $i$ in stratum $j$ on one of the parameters $\widetilde{\varvec{\theta }}$ from the set $\left\{ {\widetilde{\varvec{\gamma }},\widetilde{\varvec{\beta }}, {\text{d}}\widetilde{\Lambda }_{0} \left( t \right), \widetilde{\Lambda }_{0} \left( t \right),{ }\widetilde{\pi }\left( {\tau_{1} ,\tau_{2} ;{\varvec{x}}} \right)} \right\}$, $i \in \left\{ {1, \ldots ,n_{ }^{\left( j \right)} } \right\}$, $j \in \left\{ {1, \ldots ,J} \right\}$. We can show that ${{\varvec{\Delta}}}_{i,j} \left( {\widetilde{\varvec{\theta }}} \right) = \xi_{i,j} \varvec{ IF}_{i,j}^{\left( 2 \right)} \left( {\widetilde{\varvec{\theta }}} \right) + \xi_{i,j} V_{i,j} \exp \left( {\widetilde{\varvec{\gamma }}^{\prime} {\varvec{B}}_{i,j} } \right){\varvec{IF}}_{i,j}^{\left( 3 \right)} \left( {\widetilde{\varvec{\theta }}} \right)$. Explicit forms of ${\varvec{IF}}_{i,j}^{\left( s \right)} \left( {\widetilde{\varvec{\theta }}} \right)$, $s \in \left\{ {2,3} \right\}$, are given in Appendix 2 and are derived in Web Appendix E.1. The superscript 3 emphasizes that ${\varvec{IF}}_{i,j}^{\left( 3 \right)} \left( {\widetilde{\varvec{\theta }}} \right)$ involves variables that are measured only on individuals in the phase-three sample. Thus $\xi_{i,j} V_{i,j} {\varvec{IF}}_{i,j}^{\left( 3 \right)} \left( {\widetilde{\varvec{\theta }}} \right)$ is zero if individual $i$ in stratum $j$ is not in the phase-three sample. However, such an individual affects $\widetilde{\varvec{\theta }}$ through her/his influence on $\widetilde{\varvec{\gamma }}$ via $\xi_{i,j} {\varvec{IF}}_{i,j}^{\left( 2 \right)} \left( {\widetilde{\varvec{\theta }}} \right)$, as he/she is used to estimate the phase-three sampling weights.

### Variance decomposition and estimation from influence functions

From ${\text{var}}\left( {\widetilde{\varvec{\theta }}} \right) \approx {\text{var}}\left\{ {\mathop \sum\nolimits_{j = 1}^{J} \mathop \sum\nolimits_{i = 1}^{{n_{ }^{\left( j \right)} }} {{\varvec{\Delta}}}_{i,j} \left( {\widetilde{\varvec{\theta }}} \right)} \right\}$, $\widetilde{\varvec{\theta }} \in \left\{ {\widetilde{\varvec{\gamma }},\widetilde{\varvec{\beta }}, {\text{d}}\widetilde{\Lambda }_{0} \left( t \right), \widetilde{\Lambda }_{0} \left( t \right),{ }\widetilde{\pi }\left( {\tau_{1} ,\tau_{2} ;{\varvec{x}}} \right)} \right\}$, the variance ${\text{var}}\left( {\widetilde{\varvec{\theta }}} \right)$ can be decomposed as

21 $$\begin{aligned} & {\text{var}}\left( {{\text{E}}\left[ {{\text{E}}\left\{ {\mathop \sum \limits_{j = 1}^{J} \mathop \sum \limits_{i = 1}^{{n_{ }^{\left( j \right)} }} {{\varvec{\Delta}}}_{i,j} \left( {\widetilde{\varvec{\theta }}} \right)|C_{1} ,C_{2} } \right\}|C_{1} } \right]} \right) \\ & \quad + {\text{E}}\left( {{\text{var}}\left[ {{\text{E}}\left\{ {\mathop \sum \limits_{j = 1}^{J} \mathop \sum \limits_{i = 1}^{{n_{ }^{\left( j \right)} }} {{\varvec{\Delta}}}_{i,j} \left( {\widetilde{\varvec{\theta }}} \right)|C_{1} ,C_{2} } \right\}|C_{1} } \right]} \right) \\ & \quad + {\text{E}}\left( {{\text{E}}\left[ {{\text{var}}\left\{ {\mathop \sum \limits_{j = 1}^{J} \mathop \sum \limits_{i = 1}^{{n_{ }^{\left( j \right)} }} {{\varvec{\Delta}}}_{i,j} \left( {\widetilde{\varvec{\theta }}} \right)|C_{1} ,C_{2} } \right\}|C_{1} } \right]} \right), \\ \end{aligned}$$

where $C_{1}$ denotes the information from the whole cohort, and $C_{2}$ denotes the information from the phase-two sample. The three terms correspond respectively to sampling from the “superpopulation”, sampling the subcohort from the cohort, and sampling the phase-three sample from the phase-two sample.

We estimate ${\text{var}}\left( {\widetilde{\varvec{\theta }}} \right)$ by

22 $$\begin{aligned} \frac{n}{n - 1}\mathop \sum \limits_{j = 1}^{J} \mathop \sum \limits_{i = 1}^{{n_{ }^{\left( j \right)} }} \frac{1}{{w_{i,j}^{\left( 2 \right)} }}\left\{ {\xi_{i,j } {\varvec{IF}}_{i,j}^{\left( 2 \right)} \left( {\widetilde{\varvec{\theta }}} \right){\varvec{IF}}_{i,j}^{\left( 2 \right)} \left( {\widetilde{\varvec{\theta }}} \right)^{\prime} + 2 \xi_{i,j } V_{i,j} \widetilde{w}_{i,j}^{\left( 3 \right)} \varvec{ IF}_{i,j}^{\left( 2 \right)} \left( {\widetilde{\varvec{\theta }}} \right){\varvec{IF}}_{i,j}^{\left( 3 \right)} \left( {\widetilde{\varvec{\theta }}} \right)^{\prime} } \right. \\ & \quad \left. { + \xi_{i,j } V_{i,j} \widetilde{w}_{i,j}^{\left( 3 \right)} {\varvec{IF}}_{i,j}^{\left( 3 \right)} \left( {\widetilde{\varvec{\theta }}} \right){\varvec{IF}}_{i,j}^{\left( 3 \right)} \left( {\widetilde{\varvec{\theta }}} \right)^{\prime} } \right\} + \mathop \sum \limits_{j = 1}^{J} \mathop \sum \limits_{i = 1}^{{n_{ }^{\left( j \right)} }} \sigma_{i,j}^{\left( 2 \right)} w_{i,j}^{\left( 2 \right)} \left\{ {\xi_{i,j } {\varvec{IF}}_{i,j}^{\left( 2 \right)} \left( {\widetilde{\varvec{\theta }}} \right){\varvec{IF}}_{i,j}^{\left( 2 \right)} \left( {\widetilde{\varvec{\theta }}} \right)^{\prime} } \right. \\ & \quad \left. { + 2 \xi_{i,j } V_{i,j} \widetilde{w}_{i,j}^{\left( 3 \right)} {\varvec{IF}}_{i,j}^{\left( 2 \right)} \left( {\widetilde{\varvec{\theta }}} \right){\varvec{IF}}_{i,j}^{\left( 3 \right)} \left( {\widetilde{\varvec{\theta }}} \right)^{\prime} + \xi_{i,j } V_{i,j} \widetilde{w}_{i,j}^{\left( 3 \right)} {\varvec{IF}}_{i,j}^{\left( 3 \right)} \left( {\widetilde{\varvec{\theta }}} \right){\varvec{IF}}_{i,j}^{\left( 3 \right)} \left( {\widetilde{\varvec{\theta }}} \right)^{\prime} } \right\} \\ & \quad + \mathop \sum \limits_{j = 1}^{J} \mathop \sum \limits_{i = 1}^{{n_{ }^{\left( j \right)} }} \mathop {\sum \limits_{k = 1}}_{k \ne i}^{{n_{ }^{\left( j \right)} }} \sigma_{i,k,j}^{\left( 2 \right)} w_{i,k,j}^{\left( 2 \right)} \left\{ {\xi_{i,j } {\varvec{IF}}_{i,j}^{\left( 2 \right)} \left( {\widetilde{\varvec{\theta }}} \right) + \xi_{i,j } V_{i,j} \widetilde{w}_{i,j}^{\left( 3 \right)} {\varvec{IF}}_{i,j}^{\left( 3 \right)} \left( {\widetilde{\varvec{\theta }}} \right)} \right\}\left\{ {\xi_{k,j } {\varvec{IF}}_{k,j}^{\left( 2 \right)} \left( {\widetilde{\varvec{\theta }}} \right)} \right. \\ & \quad + \left. {\xi_{k,j } V_{k,j } \widetilde{w}_{k,j}^{\left( 3 \right)} {\varvec{IF}}_{i,j}^{\left( 3 \right)} \left( {\widetilde{\varvec{\theta }}} \right)} \right\}^{\prime} + \mathop \sum \limits_{j = 1}^{J} \mathop \sum \limits_{i = 1}^{{n_{ }^{\left( j \right)} }} \widetilde{\sigma }_{i,j}^{\left( 3 \right)} \widetilde{w}_{i,j}^{\left( 3 \right)} \xi_{i,j } V_{i,j} \widetilde{w}_{i,j}^{\left( 3 \right)} \widetilde{w}_{i,j}^{\left( 3 \right)} {\varvec{IF}}_{i,j}^{\left( 3 \right)} \left( {\widetilde{\varvec{\theta }}} \right)\varvec{ IF}_{i,j}^{\left( 3 \right)} \left( {\widetilde{\varvec{\theta }}} \right)^{\prime} . \\ \end{aligned}$$

See Web Appendix E.2 for details. Variability of the estimated phase-three weights is accounted for in a part of $\xi_{i,j } {\varvec{IF}}_{i,j}^{\left( 2 \right)} \left( {\tilde{\varvec{\theta }}} \right)$.

## Software: CaseCohortCoxSurvival on CRAN

“Dfbetas”, which approximate influences and are available from survival software, can be used to estimate the variance of $\widehat{\varvec{\beta }}$ from unstratified case-cohort data (Therneau and Li 1999), and similar code was given to estimate the variance of $\widehat{\varvec{\beta }}$ (which corresponds to Estimate II in Borgan et al. (2000)) for stratified case-cohort designs (Samuelsen et al. 2007). The cch function from the CRAN package survival (Therneau et al. 2023) deals with Estimate I and Estimate II of Borgan et al. (2000). The CRAN package cchs (Jones 2018, 2020) was created for Estimate III of Borgan et al. (2000), but we do not consider Estimate III. The twophase function from the CRAN package survey (Lumley 2021) estimates ${\varvec{\beta}}$ and its variance from a phase-two sample, and thus from unstratified or stratified case-cohort data. The previous papers did not discuss pure risk, however. SAS code was proposed for Estimate III for stratified case-cohort studies and pure risk (Langholz and Jiao 2007), but we have been unable to find online procedures at the site mentioned in the original article. The CRAN package NestedCohort (Mark and Katki 2006) was removed from the CRAN repository, but its formerly available version can be found at https://dceg.cancer.gov/tools/analysis/nested-cohort and on the CRAN archive. More general survey software can accommodate weight calibration in addition to stratification (Lumley 2021), but R code showing how to use these more general programs to estimate ${\varvec{\beta}}$, as referenced in Breslow et al. (2009a, 2009b), are no longer online. Thus, there is a need for convenient software to allow for stratification, weight calibration and missing phase-two data.

We have created the CaseCohortCoxSurvival CRAN package available at https://CRAN.R-project.org/package=CaseCohortCoxSurvival, to facilitate such analyses. We present a script in Table 4 to illustrate convenient analysis of mortality data from Golestan, Iran in Sect. 8. Extensive details on the features and arguments of the caseCohortCoxSurvival function will be provided elsewhere.

## Simulations

### Simulation designs

We compared how well the methods in Sects. 3.3 and 4.3 estimate the variance of the log-relative hazard and of pure risk estimates in simulated cohorts. We also evaluated the gain in precision from using calibrated weights rather than the design weights. We considered a range of scenarios, defined by the models described below and by parameter values in Web Tables 1 and 2 of Web Appendix D.1.

We simulated cohorts with $n \in \left\{ {5 \times 10^{3} ,10^{4} } \right\}$ and used time on study as the time scale. We simulated three covariates ${\varvec{X}} = \left( {X_{1} ,X_{2} ,X_{3} } \right)^{\prime}$: $X_{1} \sim {\mathcal{N}}\left( {0,1} \right)$, $X_{2}$ takes values in $\Omega_{{X_{2} }} = \left\{ {0,1,2} \right\}$ with respective probabilities $\left\{ {p_{{0|X_{1} }} ,p_{{1|X_{1} }} ,p_{{2|X_{1} }} } \right\}$, given in Web Table 1 in Web Appendix D.1, and $X_{3} \sim {\mathcal{N}}\left( {\alpha_{1} X_{1} + \alpha_{2}X_{2} ,1} \right)$, where ${\mathcal{N}}\left( {a,b} \right)$ denotes the Normal distribution with mean $a$ and variance $b$. We defined a categorical variable $W$ from $X_{2}$ and from a binary variable based on $X_{1}$: $W = 0 \times I\left( {X_{1} \ge 0,X_{2} = 0} \right) + 1 \times I\left( {X_{1} < 0,X_{2} < 2} \right) + 2 \times I\left( {X_{1} \ge 0,X_{2} > 0} \right) + 3 \times I\left( {X_{1} < 0,X_{2} = 2} \right)$, where *I*() is the indicator function. We simulated proxies of $X_{1}$ and $X_{3}$, $\widetilde{X}_{1}$ and $\widetilde{X}_{3}$, as $\widetilde{X}_{1} = X_{1} + \varepsilon_{1}$, $\widetilde{X}_{3} = X_{3} + \varepsilon_{3}$, with $\varepsilon_{1}$ and $\varepsilon_{3}$ independently distributed as Normal ${\mathcal{N}}\left( {0,0.75^{2} } \right)$, so that ${\text{corr}}\left( {\tilde{X}_{1} ,X_{1} } \right) = {\text{corr}}\left( {\tilde{X}_{3} ,X_{3} } \right) = 0.8$. We simulated failure time $T$ from a Cox proportional hazards model with hazard $\lambda \left( {t;{\varvec{X}}} \right) = \lambda_{0} \times \exp \left( {\beta_{1} X_{1} + \beta_{2} X_{2} + \beta_{3} X_{3} } \right)$, where the baseline hazard $\lambda_{0} = \frac{{p_{Y} }}{{{\text{E}}\left\{ {\exp \left( {\beta_{1} X_{1} + \beta_{2} X_{2} + \beta_{3} X_{3} } \right)} \right\} \times 10}}$, $p_{Y} \in \left\{ {0.02, 0.05, 0.1} \right\}$, is a constant calculated to have approximately 98%, 95% or 90% 10-year pure survival probability. Parameters $\alpha_{1}$, $\alpha_{2}$, $\beta_{1}$, $\beta_{2}$ and $\beta_{3}$ are in Web Table 2 in Web Appendix D.1. Cohort entry time, $E$, was uniform on the first 5 years, and we assumed the time to censoring by loss to follow-up, $C$, had an exponential distribution with hazard $\frac{10}{{ - \log \left( {0.98} \right)}}$, corresponding to a pure risk of loss to follow-up of 2% in 10 years. We assumed $T$, $E$ and $C$ were mutually independent. We let $\widetilde{T} = {\text{min}}\left( {T,10 - E,C} \right)$ be the observed time. The total follow-up time on time interval $(\tau_{1} ,\tau_{2} ]$ was thus ${\text{max}}\left\{ {0,{\text{min}}\left( {\tilde{T},\tau_{2} } \right) - \tau_{1} } \right\}$.

We assumed that $X_{2}$, $W$, $\widetilde{X}_{1}$, $\widetilde{X}_{3}$, $\widetilde{T}$ and the case status were known for everybody in the cohort, but $X_{1}$ and $X_{3}$ were available only for individuals in phase-two. We sampled from the four strata defined by $W$; thus stratum “0” is low risk, strata “1” and “2” are both medium risk, and stratum “3” is high risk. We sampled without replacement fixed numbers of individuals, $m^{\left( j \right)}$, independently across strata, $j \in \left\{ {0,1,2,3} \right\}$, and independently of the case status. The $m^{\left( j \right)}$ depended on the expected numbers of failures and of individuals in the strata via $m^{\left( j \right)} = \left\lfloor \frac{{\lambda_{0} \times 10 \times {\text{E}}\left\{ {\exp \left( {\beta_{1} X_{1} + \beta_{2} X_{2} + \beta_{3} X_{3} } \right)|W = j} \right\} }}{{1 - \lambda_{0} \times 10 \times {\text{E}}\left\{ {\exp \left( {\beta_{1} X_{1} + \beta_{2} X_{2} + \beta_{3} X_{3} } \right)|W = j} \right\} }} \times {\text{E}}\left( {n^{\left( j \right)} } \right) \times K + \frac{1}{2} \right\rfloor$, where $K \in \left\{ {2, 4} \right\}$ is the number of non-cases we wish to sample for each case, and $\lfloor$   $\rfloor$ is the floor function. The subcohort consisted of all the sampled subjects from the *J* strata. Then, we sample all the cases in the cohort (some may have been included in the subcohort); the phase-two (or case-cohort) sample consisted of the subcohort and all the cases. Design weights were computed as in Sect. 3.3. Calibration of the weights was performed against the auxiliary variables proposed in Sect. 4.1, with the values of covariates $X_{1}$ and $X_{3}$ in the full cohort imputed from weighted linear regressions of $X_{1}$ on $\widetilde{X}_{1}$ and $W$, and of $X_{3}$ on $\widetilde{X}_{1}$ and $\widetilde{X}_{3}$.

For each scenario, we simulated 5000 cohorts. We estimated the log-relative hazard ${\varvec{\beta}} = \left( {\beta_{1} ,\beta_{2} ,\beta_{3} } \right)^{\prime}$ and pure risks $\pi \left( {\tau_{1} ,\tau_{2} ; {\varvec{x}}} \right)$ in time interval $(\tau_{1} ,\tau_{2} ] = \left( {0,8} \right]$ and for covariate profiles ${\varvec{x}} \in \left\{ {\left( { - 1, 1, - 0.6} \right)^{\prime} ,\left( {1, - 1, 0.6} \right)^{\prime} ,\left( {1, 1, 0.6} \right)^{\prime} } \right\}$, using the following sampling designs and methods of analysis: the stratified case-cohort with design weights (SCC); the stratified case-cohort with calibrated weights (SCC.Calib); the unstratified case-cohort with design weights (USCC); and the unstratified case-cohort with calibrated weights (USCC.Calib). We then estimated their variance. For each simulated realization, we obtained the variance estimate $\widehat{V}$ for SCC from Eq. (14) and the robust variance estimate ($\widehat{V}_{{{\text{Robust}}}}$) from Eq. (15). For SCC.Calib, we used $\widehat{V}$ in Eq. (18) and $\widehat{V}_{{{\text{Robust}}}}$ in Eq. (19). For USCC and USCC.Calib, we used the variance estimates in Eqs. (14), (15), (18) and (19) with $J = 1$. Corresponding 95% confidence intervals (CIs) were computed assuming normality. As a point of reference, we also estimated these parameters using the data from the whole cohort (Cohort).

### Simulation results

**Fig. 1:**
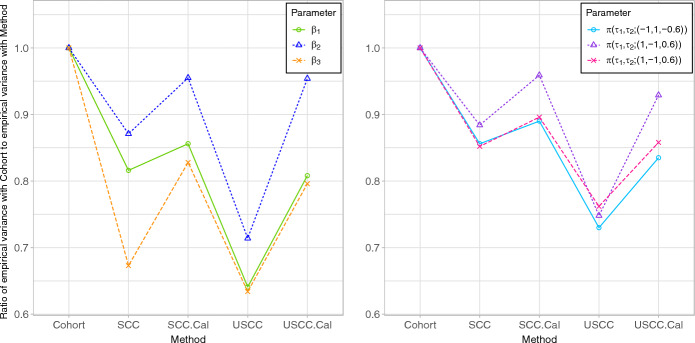
Ratio of empirical variance of log-relative hazard and pure risk estimates with the whole cohort to that when using different sampling designs and methods of analysis. The results are obtained from 5000 simulated cohorts with $n=$ 10,000, ${p}_{Y}=$ 0.02, $K=$ 2. The variance ratio is a measure of relative efficiency

The simulation results for the scenario with *n* = 10,000, $p_{Y} = 0.02$ and *K* = 2 are displayed in Fig. 1, Tables 1 and 2; see Web Table 3 to 20 in Web Appendix D.2 for other scenarios. The robust variance formula overestimated the variance (Table 1) and yielded supra-nominal confidence interval coverage (Table 2) for most log-relative hazards and pure risks with stratified designs, and for pure risk with unstratified designs. Weight calibration led to smaller variances (Table 1), as expected, because it led to a smaller phase-two component of the variance (see also Web Appendix D.4). In addition, robust variance estimates were approximately valid with calibrated weights, except for one pure risk (Tables 1 and 2). Because they properly accounted for the sampling features, the variance estimates in Eq. (14) for design weights and Eq. (18) for calibrated weights yielded proper coverage in all designs (Table 2), except for $\log \left\{ {\pi \left( {\tau_{1} ,\tau_{2} ; \varvec{x}} \right)} \right\}$ when $\varvec{x} = \left( { - 1, 1, - 0.6} \right)^{\prime}$, for which the full cohort analysis also had supra-nominal coverage. As shown in Fig. 1, stratification and/or weight calibration improved efficiency. Moreover, the unstratified case-cohort with weight calibration was nearly as efficient as the stratified case-cohort with weight calibration, and both were considerably more efficient than analyses with design weights. With design weights, stratification improved efficiency compared to the unstratified case-cohort design.

**Table 1:** Mean of estimated variances of log-relative hazard and pure risk estimates, from using different sampling designs, methods of analysis and variance estimation, over 5000 simulated cohorts with $n=$ 10,000, ${p}_{Y}=$ 0.02, $K=$ 2

Parameter	Cohort	SCC		SCC.Calib		USCC		USCC.Calib	
		${\widehat{V}}_{{\text{Robust}}}$	$\widehat{V}$	${\widehat{V}}_{{\text{Robust}}}$	$\widehat{V}$	${\widehat{V}}_{{\text{Robust}}}$	$\widehat{V}$	${\widehat{V}}_{{\text{Robust}}}$	$\widehat{V}$
$${\varvec{\beta}}$$									
${\beta }_{1}$	0.0069	0.0102	0.0087	0.0084	0.0082	0.0106	0.0106	0.0086	0.0086
	(0.007)	(0.0085)		(0.0081)		(0.0108)		(0.0087)	
${\beta }_{2}$	0.0097	0.0139	0.0114	0.0103	0.0102	0.014	0.014	0.0103	0.0103
	(0.01)	(0.0115)		(0.0105)		(0.014)		(0.0105)	
${\beta }_{3}$	0.0068	0.0102	0.0102	0.0084	0.0084	0.0107	0.0107	0.0087	0.0087
	(0.0069)	(0.0103)		(0.0083)		(0.0109)		(0.0087)	
$${\text{log}}\left\{\pi \left({\tau }_{1},{\tau }_{2};{\varvec{x}}\right)\right\}$$									
${\varvec{x}}=\left(-1, 1, -0.6\right)^{\prime}$	0.0122	0.0172	0.014	0.0142	0.0137	0.0181	0.0159	0.0145	0.0145
	(0.0119)	(0.0136)		(0.0133)		(0.0158)		(0.014)	
${\varvec{x}}=\left(1, -1, 0.6\right)^{\prime}$	0.062	0.0861	0.0697	0.0664	0.066	0.086	0.0837	0.0676	0.0676
	(0.0618)	(0.0688)		(0.0649)		(0.0823)		(0.0676)	
${\varvec{x}}=\left(1, 1, 0.6\right)^{\prime}$	0.0277	0.0379	0.0333	0.0318	0.0315	0.0386	0.0363	0.0327	0.0327
	(0.0274)	(0.0326)		(0.0308)		(0.0361)		(0.0323)	

The corresponding empirical variances are displayed between parentheses

**Table 2:** Coverage of 95% CIs of log-relative hazard and pure risk estimates, from using different sampling designs, methods of analysis and variance estimation, over 5,000 simulated cohorts with $n=$ 10,000, ${p}_{Y}=$ 0.02, $K=$ 2

Parameter	Cohort	SCC		SCC.Calib		USCC		USCC.Calib	
		${\widehat{V}}_{{\text{Robust}}}$	$\widehat{V}$	${\widehat{V}}_{{\text{Robust}}}$	$\widehat{V}$	${\widehat{V}}_{{\text{Robust}}}$	$\widehat{V}$	${\widehat{V}}_{{\text{Robust}}}$	$\widehat{V}$
$${\varvec{\beta}}$$									
${\beta }_{1}$	0.9476	0.9668*	0.9524	0.953	0.9506	0.947	0.947	0.9464	0.9464
${\beta }_{2}$	0.9486	0.9716*	0.9522	0.9498	0.9496	0.9554	0.9554	0.9466	0.9466
${\beta }_{3}$	0.9422*	0.9492	0.9494	0.9498	0.9498	0.9454	0.9454	0.9482	0.9482
$${\text{log}}\left\{\pi \left({\tau }_{1},{\tau }_{2};{\varvec{x}}\right)\right\}$$									
${\varvec{x}}=\left(-1, 1, -0.6\right)^{\prime}$	0.956*	0.973*	0.9568*	0.9604*	0.9554	0.9656*	0.9526	0.956*	0.956*
${\varvec{x}}=\left(1, -1, 0.6\right)^{\prime}$	0.952	0.9722*	0.9518	0.9542	0.9532	0.954	0.9522	0.95	0.95
${\varvec{x}}=\left(1, 1, 0.6\right)^{\prime}$	0.952	0.9666*	0.9542	0.9538	0.952	0.9574*	0.95	0.9502	0.9502

*Indicates coverage outside the expected interval [0.9440; 0.9560]

A few remarks follow. First, variables $X_{1}$ and $X_{3}$ were only measured in the phase-two sample. The strongest increase in efficiency from calibration was usually for $\widehat{\beta }_{2}$, because $X_{2}$ was measured in the entire cohort (see also Sect. 8). With weaker proxies, the efficiency gain from calibration would be more modest, and robust variance estimates may be too large (see Web Appendix D.6). Second, for log-relative hazards, the nominal coverage of the 95% CIs suggested that inference can be based on asymptotic normality, even with calibrated weights. We log-transformed the pure risks to improve coverage based on asymptotic Normal theory. Third, some authors used *post-stratified* weights instead of design weights, by having a separate stratum for cases and excluding cases from the strata with non-cases (Borgan et al. 2000; Samuelsen et al. 2007). This approach improved the precision of estimates with SCC and USCC negligibly (variance ratios of 1.01 or less), compared to using design weights (Web Appendix D.5). Finally, each stratum in the cohort and in the case-cohort had substantial numbers of subjects. Unreported simulation with very few subjects and cases in stratum $W = 0$ led to similar results.

Simulations concerning missing phase-two data showed that Eq. (22) in Sect. 5.5 and a simpler formula that ignores variability in the estimated weights (Web Appendix F.3) yielded nominal confidence interval coverage of log-relative hazards and pure risk (Web Appendix G), but in non-reported simulations with larger proportions missing, the simpler formula overestimated the variance. We therefore recommend using Eq. (22), as is computed in CaseCohortCoxSurvival available at https://CRAN.R-project.org/package=CaseCohortCoxSurvival.

## Data analysis

The Golestan Cohort included 49,819 individuals aged 36–81 and recruited in 2003–2009 (Pourshams et al. 2010). To reduce computation, we randomly sampled *n* = 30,000 individuals and analyzed this subset. We used the age-scale and assumed a Cox proportional hazards model predicting mortality from baseline variables: *X*_1_ = indicator of male gender, *X*_2_ = wealth score, *X*_3_ = indicator of former smoker (cigarettes, nass, or opium), *X*_4_ = indicator of current smoker, *X*_5_ = indicator of morbidity, $X_{6} = X_{1} X_{3}$ and $X_{7} = X_{1} X_{4} .$ We used “never smoker” as the reference category, and morbidity was a binary indicator with value 1 if the individuals had at least one of the following morbidities at baseline: cardiovascular disease, cerebrovascular accident, hypertension, diabetes, chronic obstructive pulmonary disease, tuberculosis, cancer. The wealth score had been computed from information such as house ownership and number and type of household appliances; see Islami et al. (2009). We also estimated the pure risk in interval $(\tau_{1} ,\tau_{2} ] = \left( {52,66} \right]$ and for covariate profiles ${\varvec{x}} \in \left\{ {\left( {0, - 0.4,0,1,\varvec{0}_{3} } \right)^{\prime} ,\left( {0,0.4,0,1,\varvec{0}_{3} } \right)^{\prime} ,\left( {\varvec{0}_{4} ,1,\varvec{0}_{2} } \right)^{\prime} ,\varvec{0}_{7}^{\prime} } \right\}$, where $\varvec{0}_{a}$ is the $a \times 1$ vector of zeros, and where for example $\left( {0, - 0.4,0,1,\varvec{0}_{3} } \right)^{\prime}$ corresponds to the profile of a currently smoking woman with a low wealth score, while $\left( {\varvec{0}_{4} ,1,\varvec{0}_{2} } \right)^{\prime}$ corresponds to a never-smoking woman with morbidity at baseline. We assumed that age, gender, smoking status, morbidity, residence (urban, rural), ethnicity (Turkmen, others), marital status (unmarried, married, widowed, divorced/separated, other), education (nil, less than 5th, 6th-8th, 9th-12th, College), socioeconomic status (low, low to medium, medium to high, high), death status and follow-up time were known for everybody in the cohort, but the wealth score was available only for individuals in phase-two. We sampled 33, 42, 192, 246, 57, 62, 313, 382, 82, 86, 391, 477, 565, 770, 1934 and 2949 individuals respectively in the 16 strata defined by gender (male, female), residence and four baseline age categories ([36,45), [45,50), [50,55) and [55,81]), so that we expected approximately one non-case per case in each stratum. We estimated the log-relative hazards and pure risks using SCC, SCC.Calib, USCC and USCC.Calib (see notation and methods of analysis in Sect. 5). We used gender, socioeconomic status, age at baseline, marital status, ethnicity, education and residence as proxies to impute the wealth score for the entire cohort and then calibrated the design weights. We also analyzed the whole cohort (*n* = 30,000).

Table 3 displays the variances of log-relative hazard and pure risk parameters; see Web Table 55 in Web Appendix H for parameter estimates. When using design weights, robust variance estimates were larger for the log-relative hazards of covariates $X_{1}$ and $X_{2}$, for all the pure risks in the stratified design, and for the pure risks with profiles ${\varvec{x}} \in \left\{ {\left( {0, - 0.4,0,1,\varvec{0}_{3} } \right)^{\prime} ,\left( {\varvec{0}_{4} ,1,\varvec{0}_{2} } \right)^{\prime} ,\varvec{0}_{7}^{\prime} } \right\}$ in the unstratified design. In the stratified design, $\widehat{V}_{{{\text{Robust}}}}$ agreed well with $\widehat{V}$ for 5 of the 7 log-relative hazard parameters, possibly because stratification was only based on $X_{1}$.

**Table 3:** Estimated variances of log-relative hazard and pure risk parameters from using different sampling designs, methods of analysis and variance estimation, in the Golestan Cohort ($n=$ 30,000)

Parameter	Cohort	SCC		SCC.Calib		USCC		USCC.Calib	
		${\widehat{V}}_{{\text{Robust}}}$	$\widehat{V}$	${\widehat{V}}_{{\text{Robust}}}$	$\widehat{V}$	${\widehat{V}}_{{\text{Robust}}}$	$\widehat{V}$	${\widehat{V}}_{{\text{Robust}}}$	$\widehat{V}$
$${\varvec{\beta}}$$									
${\beta }_{1}$	0.0013	0.0018	0.0016	0.0013	0.0013	0.0025	0.0025	0.0013	0.0013
${\beta }_{2}$	0.005	0.0075	0.0072	0.0055	0.0055	0.0104	0.0104	0.0061	0.0061
${\beta }_{3}$	0.0173	0.0289	0.0289	0.0178	0.0178	0.0394	0.0394	0.0173	0.0173
${\beta }_{4}$	0.0029	0.0057	0.0057	0.0029	0.0029	0.007	0.007	0.0031	0.0031
${\beta }_{5}$	0.0009	0.0013	0.0013	0.0009	0.0009	0.002	0.002	0.0009	0.0009
${\beta }_{6}$	0.0217	0.0342	0.0342	0.0219	0.0219	0.0497	0.0497	0.0217	0.0217
${\beta }_{7}$	0.0044	0.0076	0.0076	0.0044	0.0044	0.0103	0.0103	0.0045	0.0045
$$\pi \left({\tau }_{1},{\tau }_{2};{\varvec{x}}\right)$$									
${\varvec{x}}=\left(0,-\mathrm{0.4,0},1,\varvec{0}_{3}\right)^{\prime}$	0.00012	0.00023	0.00022	0.00012	0.00012	0.00031	0.00030	0.00013	0.00013
${\varvec{x}}=\left(\mathrm{0,0.4,0},1,\varvec{0}_{3}\right)^{\prime}$	5.10E−05	9.20E−05	8.90E−05	5.20E−05	5.10E−05	0.00010	0.00010	5.70E−05	5.70E−05
${\varvec{x}}=\left(\varvec{0}_{4},1,\varvec{0}_{2}\right)^{\prime}$	2.50E−05	4.10E−05	3.50E−05	2.50E−05	2.50E−05	4.50E−05	4.30E−05	2.70E−05	2.70E−05
${\varvec{x}}=\varvec{0}_{7}^{\prime}$	5.60E−06	8.50E−06	6.70E−06	5.80E−06	5.70E−06	8.90E−06	8.30E−06	5.60E−06	5.60E−06

${\beta }_{p}$ denotes the log-relative hazard parameter of covariate ${X}_{p}$, $p\in \{1,\dots ,7\}$

Weight calibration improved efficiency, and robust variance estimates were very close to $\widehat{V}$ for all parameters. Notably, calibration led to estimates with almost as much precision as with the full cohort, not only for covariates that were available on the whole cohort, but also for wealth score, for which there were good proxies.

Web Appendix I presents pseudo-code for all of the steps for estimation in Sects. 3 and 4. To illustrate how easily such analyses can be performed with the CaseCohortCoxSurvival CRAN package (available at https://CRAN.R-project.org/package=CaseCohortCoxSurvival), we present a script for SCC and SCC.Calib in Table 4.

**Table 4:**
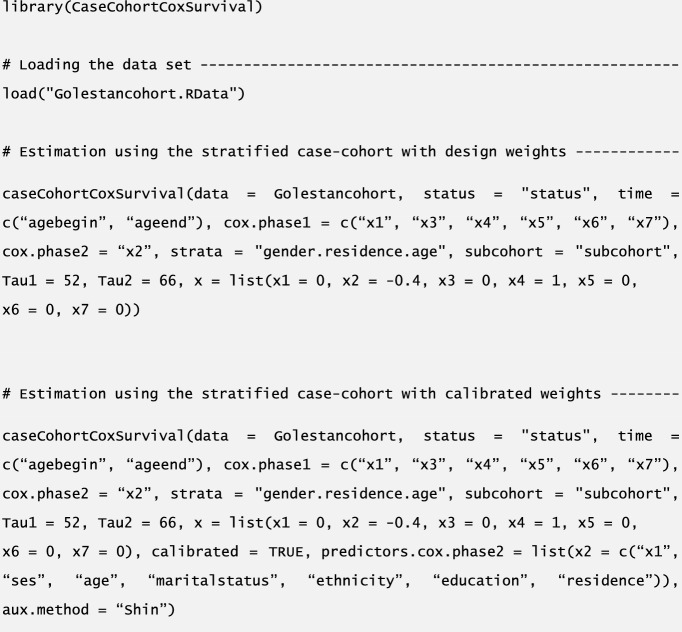
R script using the CaseCohortCoxSurvival R package to obtain variance estimates $\widehat{V}$ and ${\widehat{V}}_{{\text{Robust}}}$ with SCC and SCC.Calib, for the log-relative hazards and pure risk with profile ${\varvec{x}}=\left(0,-\mathrm{0.4,0},1,\varvec{0}_{3}\right)^{\prime}$ in Table 3

## Discussion

We presented a unified approach to analysis of case-cohort data that allows the practitioner to take advantage of various options and improvements in design and analysis since the landmark paper of Prentice (1986). We used influence functions adapted to the various design and analysis options together with variance calculations that take two-phase sampling into account. We developed corresponding software CaseCohortCoxSurvival, available at https://CRAN.R-project.org/package=CaseCohortCoxSurvival, that facilitates analysis with and without stratification and/or weight calibration, for subcohort sampling with or without replacement. We allow for phase-two data to be missing at random for stratified designs. We provide inference not only for log-relative hazards in the Cox model, but also for covariate-specific cumulative hazards and pure risks. We hope these calculations and software will promote wider and more principled design and analysis of case-cohort data, for which there is a need (Sharp et al. 2014). Detailed features and arguments of the CaseCohortCoxSurvival CRAN R package available at https://CRAN.R-project.org/package=CaseCohortCoxSurvival will be described elsewhere. Convenient software of the type we propose does not appear to be available online (Sect. 6).

We found that weight calibration improves efficiency with stratified or unstratified sampling of the subcohort, in line with previous findings for unstratified designs. We found theoretically and empirically that the robust variance estimate (Barlow 1994) is nearly unbiased if the covariances of the phase-two sampling indicators, $\sigma_{i,k,j}$, $i \ne k$, are zero, as when the subcohort members are sampled with replacement (Table 5). For sampling without replacement, these covariances are negative, which tends to bias the robust variance estimate upward. This has been noted for log-relative hazards in stratified designs (Gray 2009; Samuelsen et al. 2007), but we also found this bias for pure risk in unstratified designs. With weight calibration based on strong predictors of phase-two covariates, the robust variance had little bias (Table 5). Nonetheless, we recommend our influence-based approach with complete variance decomposition for theoretical and empirical reasons. In addition, and as previously recommended (Sharp et al. 2014), we stress the practical importance of describing the design fully in publications, including stratification details and whether or not the subcohort was sampled with replacement.

**Table 5:** Sampling designs and methods of analysis for which the robust variance estimator is approximately valid for relative hazard and pure risk estimates

Parameter	Sampling with replacement	Sampling without replacement			
		Unstratified sampling with design weights	Unstratified sampling with calibrated weights	Stratified sampling with design weights	Stratified sampling with calibrated weights*
*Approximate validity of robust variance estimate*					
Relative hazard	Yes	Yes	Yes	No	Yes*
Pure risk	Yes	No	Yes*	No	Yes*

We categorize the robust variance estimate as approximately valid if theoretical calculations indicate that the bias is negligible and/or if in simulations the means of the robust variance estimates were close to the means of the two-phase variance procedures we describe.

*Holds if the phase-one covariates are good predictors of covariates measured only in phase-two. If the proxies of phase-two covariates are too weak, the robust variance estimate may be inappropriate

In our simulation, the unstratified case-cohort with calibrated weights was nearly as efficient as the stratified case-cohort with calibrated weights. This is probably because information used to define the strata was also used for calibration. However, if strata depended for example only on time of events or censoring, but imputation of phase-two covariates depended on other phase-one covariates, a stratified calibrated approach might be more efficient than the corresponding unstratified calibrated one.

To obtain the subcohort, we sampled fixed numbers of individuals from the strata, independently of the case status. Thus, some of the cases may have been included in the subcohort. Alternatively, one might want to sample fixed numbers of non-cases. To do so, the strata could be redefined by excluding the cases, and an additional stratum containing all the cases, could be created. It is possible to analyze data from this slightly modified design CRAN R package (available at https://CRAN.R-project.org/package=CaseCohortCoxSurvival) by sampling all the cases from the case stratum, so that all cases are included in the case-cohort and have unit design weights.

We focused on the “standard” case-cohort design, where all the cases are sampled from the cohort. If the event of interest is not rare, one may want to only include a fraction of the cases in the case-cohort. The derivations presented in this paper can be extended to such a design, sometimes called the *generalized case-cohort* design (Kim et al. 2018; Xu et al. 2022). For example, a weighted version $\xi_{i,j} w_{i,j} {\text{d}}N_{i,j} \left( t \right)$ would be employed in Eqs. (1) and (5), with the cases having non-unit design weights. The cases would then contribute to the phase-two component of the variance.

The methods we presented used design weights. Borgan et al. (2000) and Samuelsen et al. (2007) recommended weights that are post-stratified into a case stratum and multiple non-case strata. In our simulations, there was less than 2% increase in efficiency from post-stratification. Using the influences we derived for design weights with post-stratified weights (with cases in one stratum and non-cases in the original strata) yielded confidence intervals with nominal coverage (results not shown). Thus, the influence functions we provide can be used for such post-stratification. Further efficiency gains might be obtained by post-stratifying on time intervals in which follow-up ends (Chen 2001; Ding et al. 2017; Samuelsen et al. 2007) or on other features (Section 16.4.5 in Borgan et al. 2017).

An alternative approach to sampling is to select the subcohort sample size in each stratum such that the expected number of non-cases is a multiple of the observed number of cases. In unreported simulations, using the influences we gave for design weights and substituting post-stratified weights yielded valid variance estimates and coverage of confidence intervals, unless the number of cases and non-cases in a stratum is small (e.g. fewer than 10 cases and 20 non-cases).

As discussed by Keogh et al. (2018), likelihood-based methods for missing data and imputation can increase efficiency of case-cohort analyses, but, unlike stratification and weight calibration, they yield biased risk model estimates if imputation models are misspecified. Indeed, a key advantage of weight calibration is that poor imputation models reduce the efficiency gains, but do not bias estimates of risk model parameters (Lumley et al. 2011). Weight calibrated estimators are in the class of augmented inverse-probability weighted estimators that are similarly robust (Lumley et al. 2011; Robins et al. 1994).

In Sect. 5.3, we suggested modifications for times when a case had missing covariate data and no other member of the phase-three sample was at risk when the case failed. An alternative would be to weight the numerator of the Breslow estimator and only use event times $t$ from cases with complete covariate data, namely ${\text{d}}{\widetilde{\Lambda }}_{0} \left( t \right) = \frac{{\mathop \sum \nolimits_{j = 1}^{J} \mathop \sum \nolimits_{i = 1}^{{n_{ }^{\left( j \right)} }} V_{i,j} \widetilde{w}_{i,j}^{\left( 3 \right)} {\text{d}}N_{i,j} \left( t \right)}}{{\widetilde{S}_{0} \left( {t;\widetilde{\varvec{\gamma }},\widetilde{\varvec{\beta }}} \right)}}$. Unreported simulations showed this led to biased estimates of pure risks, however.

This paper dealt with covariates measured at baseline. Although the influences for log-relative hazards apply equally to time-varying covariates, modifications are needed for pure risks, and computational challenges arise for large cohorts. Moreover, pure risk estimates are uninterpretable unless the time-varying covariates are “external” (Kalbfleisch and Prentice 2011). We have assumed a common baseline hazard across strata. A stratified Cox model with different baseline hazards in each stratum would require modifications of the influences given in this paper.

## Electronic supplementary material

Below is the link to the electronic supplementary material.

Supplementary file1 (DOCX 559 KB)

## Data Availability

R code and functions used for the simulations in Section 7 are available in the Supplementary Information of this article and on GitHub at https://github.com/Etievant/CaseCohort. The R package CaseCohortCoxSurvival integrating the various functions on GitHub is available on CRAN at https://CRAN.R-project.org/package=CaseCohortCoxSurvival. We are not authorized to release the clinical data used in Sect. 8.

## Appendix 1: Influences for stratified case-cohort with calibrated weights

$$\begin{aligned} {\varvec{IF}}_{i,j}^{\left( 1 \right)} \left( {\widehat{\varvec{\eta }}} \right) & = \left\{ {\mathop \sum \limits_{l = 1}^{J} \mathop \sum \limits_{k = 1}^{{n_{ }^{\left( j \right)} }} \xi_{k,l} w_{k,l} \exp \left( {\widehat{\varvec{\eta }}^{\prime} {\varvec{A}}_{k,l} } \right){\varvec{A}}_{k,l} {\varvec{A}}_{k,l}^{\prime} } \right\}^{ - 1} {\varvec{A}}_{i,j} , \\ {\varvec{IF}}_{i,j}^{\left( 2 \right)} \left( {\widehat{\varvec{\eta }}} \right) & = - \exp \left( {\widehat{\varvec{\eta }}^{\prime} {\varvec{A}}_{i,j} } \right)\left\{ {\mathop \sum \limits_{l = 1}^{J} \mathop \sum \limits_{k = 1}^{{n_{ }^{\left( j \right)} }} \xi_{k,l} w_{k,l} \exp \left( {\widehat{\varvec{\eta }}^{\prime} {\varvec{A}}_{k,l} } \right){\varvec{A}}_{k,l} {\varvec{A}}_{k,l}^{\prime} } \right\}^{ - 1} {\varvec{A}}_{i,j} , \\ {\varvec{IF}}_{i,j}^{\left( 1 \right)} \left( {\widehat{\varvec{\beta }}^{*} } \right) & = \left\{ {\mathop \sum \limits_{l = 1}^{J} \mathop \sum \limits_{k = 1}^{{n_{ }^{\left( j \right)} }} \xi_{k,l} w_{k,l} \exp \left( {\widehat{\varvec{\eta }}^{\prime} {\varvec{A}}_{k,l} } \right){\varvec{Z}}_{k,l} {\varvec{A}}_{k,l}^{\prime} } \right\}{\varvec{IF}}_{i,j}^{\left( 1 \right)} \left( {\widehat{\varvec{\eta }}} \right), \\ \end{aligned}$$

and $${{\varvec{I}}{\varvec{F}}}_{i,j}^{(2)}\left({\widehat{{\varvec{\beta}}}}^{*}\right)={\text{exp}}\left({\widehat{{\varvec{\eta}}}}^{\prime}{{\varvec{A}}}_{i,j}\right) {{\varvec{Z}}}_{i,j}+\left\{\mathop \sum \limits_{l = 1}^{J} \mathop \sum \limits_{k = 1}^{n_{ }^{\left( j \right)}} {\xi }_{k,l}{w}_{k,l}{\text{exp}}\left({\widehat{{\varvec{\eta}}}}^{\prime}{{\varvec{A}}}_{k,l}\right){{\varvec{Z}}}_{k,l}{{\varvec{A}}}_{k,l}^{\prime}\right\}{{\varvec{I}}{\varvec{F}}}_{i,j}^{(2)}\left(\widehat{{\varvec{\eta}}}\right)$$, with $${{\varvec{Z}}}_{i,j}={\left[\sum_{l=1}^{J}\sum_{k=1}^{{n}^{(l)}}{\int \limits_{t}}{\xi }_{k,l}{w}_{k,l}{\text{exp}}\left({\widehat{{\varvec{\eta}}}}^{\prime}{{\varvec{A}}}_{k,l}\right)\left\{\frac{ {{\varvec{S}}}_{2}^{*}\left(t;\widehat{{\varvec{\eta}}},{\widehat{{\varvec{\beta}}}}^{*}\right)}{{S}_{0}^{*}\left(t;\widehat{{\varvec{\eta}}},{\widehat{{\varvec{\beta}}}}^{*}\right)}-\frac{{{\varvec{S}}}_{1}^{*}\left(t;\widehat{{\varvec{\eta}}},{\widehat{{\varvec{\beta}}}}^{*}\right){{{\varvec{S}}}_{1}^{*}\left(t;\widehat{{\varvec{\eta}}},{\widehat{{\varvec{\beta}}}}^{*}\right)}^{\prime}}{{{S}_{0}^{*}\left(t;\widehat{{\varvec{\eta}}},{\widehat{{\varvec{\beta}}}}^{*}\right)}^{2}}\right\}{{\text{d}}N}_{k,l}\left(t\right)\right]}^{-1}\left[{\int \limits_{t}}\left\{{{\varvec{X}}}_{i,j}-\frac{{{\varvec{S}}}_{1}^{*}\left(t;\widehat{{\varvec{\eta}}},{\widehat{{\varvec{\beta}}}}^{*}\right)}{{S}_{0}^{*}\left(t;\widehat{{\varvec{\eta}}},{\widehat{{\varvec{\beta}}}}^{*}\right)}\right\}\left\{{{\text{d}}N}_{i,j}\left(t\right)-\frac{{Y}_{i,j}\left(t\right){\text{exp}}\left({{\widehat{{\varvec{\beta}}}}^{*}}{\prime}{{\varvec{X}}}_{i,j}\right)\sum_{l=1}^{J}\sum_{k=1}^{{n}^{(l)}}{\xi }_{k,l} {w}_{k,l}{\text{exp}}\left({\widehat{{\varvec{\eta}}}}^{\prime}{{\varvec{A}}}_{k,l}\right){{\text{d}}N}_{k,l}\left(t\right)}{{S}_{0}^{*}\left(t;\widehat{{\varvec{\eta}}},{\widehat{{\varvec{\beta}}}}^{*}\right)}\right\}\right]$$.

$$\begin{aligned} IF_{i,j}^{\left( 1 \right)} \left\{ {{\text{d}}{\widehat{\Lambda }}_{0}^{*} \left( t \right)} \right\} & = \left\{ {\mathop \sum \limits_{l = 1}^{J} \mathop \sum \limits_{k = 1}^{{n_{ }^{\left( l \right)} }} \xi_{k,l} w_{k,l} \exp \left( {\widehat{\varvec{\eta }}^{\prime} {\varvec{A}}_{k,l} } \right)H_{k,l} \left( t \right){\varvec{A}}_{k,l}^{\prime} } \right\}{\varvec{IF}}_{i,j}^{\left( 1 \right)} \left( {\widehat{\varvec{\eta }}} \right), \\ IF_{i,j}^{\left( 2 \right)} \left\{ {{\text{d}}{\widehat{\Lambda }}_{0}^{*} \left( t \right)} \right\} & = \left\{ {S_{0}^{*} \left( {t;\widehat{\varvec{\eta }},\widehat{\varvec{\beta }}^{*} } \right)} \right\}^{ - 1} \left[ {{\text{d}}N_{i,j} \left( t \right)} \right. \\ & \quad \left. { + \exp \left( {\widehat{\varvec{\eta }}^{\prime} {\varvec{A}}_{i,j} } \right)H_{i,j} \left( t \right) + \left\{ {\mathop \sum \limits_{l = 1}^{J} \mathop \sum \limits_{k = 1}^{{n_{ }^{\left( l \right)} }} \xi_{k,l} w_{k,l} \exp \left( {\widehat{\varvec{\eta }}^{\prime} {\varvec{A}}_{k,l} } \right)H_{k,l} \left( t \right){\varvec{A}}_{k,l}^{\prime} } \right\}{\varvec{IF}}_{i,j}^{\left( 2 \right)} \left( {\widehat{\varvec{\eta }}} \right)} \right], \\ \end{aligned}$$

with $${H}_{i,j}\left(t\right)=-{\left\{{S}_{0}^{*}\left(t;\widehat{{\varvec{\eta}}},{\widehat{{\varvec{\beta}}}}^{*}\right)\right\}}^{-1}{{\text{d}}\widehat{\Lambda }}_{0}^{*}\left(t\right)\left\{{{{\varvec{S}}}_{1}^{*}\left(t;\widehat{{\varvec{\eta}}},{\widehat{{\varvec{\beta}}}}^{*}\right)}^{\prime}{{\varvec{Z}}}_{i,j}+{K}_{i,j}(t)\right\}$$, and $${K}_{i,j}\left(t\right)={Y}_{i,j}\left(t\right){\text{exp}}\left({\widehat{{\varvec{\beta}}}}^{*{\prime}}{{\varvec{X}}}_{i,j}\right)$$.

Finally, for any $s\in \left\{\mathrm{1,2}\right\}$, ${IF}_{i,j}^{(s)}\left\{{\int }_{{\tau }_{1}}^{{\tau }_{2}}{{\text{d}}\widehat{\Lambda }}_{0}^{*}\left(t\right)\right\}={\int }_{{\tau }_{1}}^{{\tau }_{2}}{IF}_{i,j}^{\left(s\right)}\left\{{{\text{d}}\widehat{\Lambda }}_{0}^{*}\left(t\right)\right\}$, and ${IF}_{i,j}^{(s)}\left\{{\widehat{\pi }}^{*}\left({\tau }_{1},{\tau }_{2};{\varvec{x}}\right)\right\}=\left\{{\frac{\partial \widehat{\pi }\left({\tau }_{1},{\tau }_{2}; {\varvec{x}}\right)}{\partial{\varvec{\beta}}}}_{|{\varvec{\beta}}={\widehat{{\varvec{\beta}}}}^{*}}\right\}{{\varvec{I}}{\varvec{F}}}_{i,j}^{(s)}\left({\widehat{{\varvec{\beta}}}}^{*}\right)+\left[{\frac{\partial \widehat{\pi }\left({\tau }_{1},{\tau }_{2}; {\varvec{x}}\right)}{\partial \left\{{\int }_{{\tau }_{1}}^{{\tau }_{2}}{\mathrm{d\Lambda }}_{0}\left(t\right)\right\}}}_{|{\mathrm{d\Lambda }}_{0}(t)={{\text{d}}\widehat{\Lambda }}_{0}^{*}\left(t\right)}\right]{IF}_{i,j}^{(s)}\left\{{\int }_{{\tau }_{1}}^{{\tau }_{2}}{{\text{d}}\widehat{\Lambda }}_{0}^{*}\left(t\right)\right\}$.

## Appendix 2: Influences for stratified case-cohort with missing covariate information and estimated design sampling phase-three weights

$${{\varvec{I}}{\varvec{F}}}_{i,j}^{(2)}\left(\widetilde{{\varvec{\gamma}}}\right)={\left\{\sum_{l=1}^{J}\sum_{k=1}^{{n}^{\left(l\right)}}{\xi }_{k,l} {V}_{k,l}{\text{exp}}\left({\widetilde{{\varvec{\gamma}}}}^{\prime}{{\varvec{B}}}_{k,l}\right){{\varvec{B}}}_{k,l}{{\varvec{B}}}_{k,l}^{\prime}\right\}}^{-1}{{\varvec{B}}}_{i,j}$$, and $${{\varvec{I}}{\varvec{F}}}_{i,j}^{(3)}\left(\widetilde{{\varvec{\gamma}}}\right)=-\left\{\sum_{l=1}^{J}\sum_{k=1}^{{n}^{\left(l\right)}}{\xi }_{k,l}{V}_{k,l}{\text{exp}}\left({\widetilde{{\varvec{\gamma}}}}^{\prime}{{\varvec{B}}}_{k,l}\right){{\varvec{B}}}_{k,l}{{\varvec{B}}}_{k,l}^{\prime}\right\}{{\varvec{B}}}_{i,j}.$$ ﻿

Then ${{\varvec{I}}{\varvec{F}}}_{i,j}^{(2)}\left(\widetilde{{\varvec{\beta}}}\right)=\left\{\sum_{l=1}^{J}\sum_{k=1}^{{n}^{\left(l\right)}}{\xi }_{k,l}{V}_{k,l}{\text{exp}}\left({\widetilde{{\varvec{\gamma}}}}^{\prime}{{\varvec{B}}}_{k,l}\right){\widetilde{{\varvec{Z}}}}_{k,l}{{\varvec{B}}}_{k,l}^{\prime}\right\}{{\varvec{I}}{\varvec{F}}}_{i,j}^{(2)}\left(\widetilde{{\varvec{\gamma}}}\right)$, and ${{\varvec{I}}{\varvec{F}}}_{i,j}^{(3)}\left(\widetilde{{\varvec{\beta}}}\right)={\widetilde{{\varvec{Z}}}}_{i,j}+\left\{\sum_{l=1}^{J}\sum_{k=1}^{{n}^{\left(l\right)}}{\xi }_{k,l}{V}_{k,l}{\text{exp}}\left({\widetilde{{\varvec{\gamma}}}}^{\prime}{{\varvec{B}}}_{k,l}\right){\widetilde{{\varvec{Z}}}}_{k,l}{{\varvec{B}}}_{k,l}^{\prime}\right\}{{\varvec{I}}{\varvec{F}}}_{i,j}^{(3)}\left(\widetilde{{\varvec{\gamma}}}\right)$, with

$$\begin{aligned} \widetilde{\varvec{Z}}_{i,j} & = w_{i,j}^{\left( 2 \right)} \times \left[ {\mathop \sum \limits_{l = 1}^{J} \mathop \sum \limits_{k = 1}^{{n_{ }^{\left( l \right)} }} \mathop \int \limits_{t} \xi_{k,l} V_{k,l} w_{k,l}^{\left( 2 \right)} \exp \left( {\widetilde{\varvec{\gamma }}^{\prime} {\varvec{B}}_{k,l} } \right)\left\{ {\frac{{ \widetilde{\varvec{S}}_{2} \left( {t;\widetilde{\varvec{\gamma }},\widetilde{\varvec{\beta }}} \right)}}{{\widetilde{S}_{0} \left( {t;\widetilde{\varvec{\gamma }},\widetilde{\varvec{\beta }}} \right)}} - \frac{{\widetilde{\varvec{S}}_{1} \left( {t;\widetilde{\varvec{\gamma }},\widetilde{\varvec{\beta }}} \right)\widetilde{\varvec{S}}_{1} \left( {t;\widetilde{\varvec{\gamma }},\widetilde{\varvec{\beta }}} \right)^{\prime} }}{{\widetilde{S}_{0} \left( {t;\widetilde{\varvec{\gamma }},\widetilde{\varvec{\beta }}} \right)^{2} }}} \right\}{\text{d}}N_{k,l} \left( t \right)} \right]^{ - 1} \\ & \quad \times \left[ {\mathop \int \limits_{t} \left\{ {{\varvec{X}}_{i,j} - \frac{{\widetilde{\varvec{S}}_{1} \left( {t;\widetilde{\varvec{\gamma }},\widetilde{\varvec{\beta }}} \right)}}{{\widetilde{S}_{0} \left( {t;\widetilde{\varvec{\gamma }},\widetilde{\varvec{\beta }}} \right)}}} \right\}\left\{ {{\text{d}}N_{i,j} \left( t \right) - \frac{{Y_{i,j} \left( t \right)\exp \left( {\widetilde{\varvec{\beta }}^{\prime} {\varvec{X}}_{i,j} } \right)\mathop \sum \nolimits_{l = 1}^{J} \mathop \sum \nolimits_{k = 1}^{{n_{ }^{\left( l \right)} }} \xi_{k,l} V_{k,l} w_{k,l}^{\left( 2 \right)} \exp \left( {\widetilde{\varvec{\gamma }}^{\prime} {\varvec{B}}_{k,l} } \right){\text{d}}N_{k,l} \left( t \right)}}{{\widetilde{S}_{0} \left( {t;\widetilde{\varvec{\gamma }},\widetilde{\varvec{\beta }}} \right)}}} \right\}} \right]. \\ \end{aligned}$$

$$\begin{aligned} & IF_{i,j}^{\left( 2 \right)} \left\{ {{\text{d}}{\widetilde{\Lambda }}_{0} \left( t \right)} \right\} = \left\{ {\widetilde{S}_{0} \left( {t;\widetilde{\varvec{\gamma }},\widetilde{\varvec{\beta }}} \right)} \right\}^{ - 1} {\text{d}}N_{i,j} \left( t \right) \\ & \quad + \left\{ {\mathop \sum \limits_{l = 1}^{J} \mathop \sum \limits_{k = 1}^{{n_{ }^{\left( j \right)} }} \xi_{k,l} V_{k,l} \exp \left( {\widetilde{\varvec{\gamma }}^{\prime} {\varvec{B}}_{k,l} } \right)\widetilde{H}_{k,l} \left( t \right){\varvec{B}}_{k,l}^{\prime} } \right\}{\varvec{IF}}_{i,j}^{\left( 2 \right)} \left( {\widetilde{\varvec{\gamma }}} \right), \\ \end{aligned}$$

and ${IF}_{i,j}^{(3)}\left\{{{\text{d}}\widetilde{\Lambda }}_{0}\left(t\right)\right\}={\widetilde{H}}_{i,j}\left(t\right)+\left\{\sum_{l=1}^{J}\sum_{k=1}^{{n}^{\left(j\right)}}{\xi }_{k,l}{V}_{k,l}{\text{exp}}\left({\widetilde{{\varvec{\gamma}}}}^{\prime}{{\varvec{B}}}_{k,l}\right){\widetilde{H}}_{k,l}\left(t\right){{\varvec{B}}}_{k,l}^{\prime}\right\}{{\varvec{I}}{\varvec{F}}}_{i,j}^{(3)}\left(\widetilde{{\varvec{\gamma}}}\right)$, with ${\widetilde{H}}_{i,j}\left(t\right)=-{{\widetilde{S}}_{0}\left(t;\widetilde{{\varvec{\gamma}}},\widetilde{{\varvec{\beta}}}\right)}^{-1}{{\text{d}}\widetilde{\Lambda }}_{0}\left(t\right)\left\{ {\widetilde{{\varvec{S}}}}_{1}{\left(t;\widetilde{{\varvec{\gamma}}},\widetilde{{\varvec{\beta}}}\right)}^{\prime}{\widetilde{{\varvec{Z}}}}_{i,j}+{\widetilde{K}}_{i,j}\left(t\right)\right\}$, and ${\widetilde{K}}_{i,j}\left(t\right)={w}_{i,j}^{\left(2\right)} {Y}_{i,j}\left(t\right){\text{exp}}\left(\widetilde{{\varvec{\beta}}}^{\prime}{{\varvec{X}}}_{i,j}\right)$.

Finally, for any $s\in \left\{\mathrm{2,3}\right\}$, ${IF}_{i,j}^{(s)}\left\{{\int }_{{\tau }_{1}}^{{\tau }_{2}}{{\text{d}}\widetilde{\Lambda }}_{0}(t)\right\}={\int }_{{\tau }_{1}}^{{\tau }_{2}}{IF}_{i,j}^{\left(2\right)}\left\{{{\text{d}}\widetilde{\Lambda }}_{0}\left(t\right)\right\}$ and ${IF}_{i,j}^{(s)}\left\{{\widehat{\pi }}^{*}\left({\tau }_{1},{\tau }_{2};{\varvec{x}}\right)\right\}=\left\{{\frac{\partial \widetilde{\pi }\left({\tau }_{1},{\tau }_{2}; {\varvec{x}}\right)}{\partial{\varvec{\beta}}}}_{|{\varvec{\beta}}=\widetilde{{\varvec{\beta}}}}\right\}{{\varvec{I}}{\varvec{F}}}_{i,j}^{(s)}\left({\widetilde{{\varvec{\beta}}}}\right)+\left[{\frac{\partial \widetilde{\pi }\left({\tau }_{1},{\tau }_{2}; {\varvec{x}}\right)}{\partial \left\{{\int }_{{\tau }_{1}}^{{\tau }_{2}}{\mathrm{d\Lambda }}_{0}(t)\right\}}}_{|{\mathrm{d\Lambda }}_{0}(t)={{\text{d}}\widetilde{\Lambda }}_{0}(t)}\right]{IF}_{i,j}^{(s)}\left\{{\int }_{{\tau }_{1}}^{{\tau }_{2}}{{\text{d}}\widetilde{\Lambda }}_{0}(t)\right\}$.
