# Supplementary material for: Cox model inference for relative hazard and pure risk from stratified weight-calibrated case-cohort data
Source: Lifetime Data Anal. 2024 Apr 2;30(3):572–99. doi: 10.1007/s10985-024-09621-2 (PMC11420370; doi:10.1007/s10985-024-09621-2)
Supplement: Supplementary file 1 — Supplementary file1 (DOCX 559 KB) [file 10985_2024_9621_MOESM1_ESM.docx]

**Supplementary Information for Cox model inference for relative hazard and pure risk from stratified weight-calibrated case-cohort data**

Lola Etievant^1^, Mitchell H. Gail^1^

[lola.etievant@nih.gov](mailto:lola.etievant@nih.gov) [ORCID 0000-0001-7562-3550](https://orcid.org/0000-0001-7562-3550)

[gailm@mail.nih.gov](mailto:gailm@mail.nih.gov) [ORCID 0000-0002-3919-3263](https://orcid.org/0000-0002-3919-3263)

^1^ National Cancer Institute, Division of Cancer Epidemiology and Genetics, Biostatistics Branch, 9609 Medical Center Drive, Rockville, MD 20850-9780.

**Table of content**

[Web Appendix A. ESTIMATION WITH COMPLETE COVARIATE DATA FOR THE WHOLE COHORT ……………………………………………………………………………… 3](#_Toc152844938)

[A.1 Parameters estimation 3](#_Toc152844939)

[A.2 Influence functions 3](#_Toc152844940)

[A.3 Variance estimation from influence functions 4](#_Toc152844941)

[Web Appendix B. ESTIMATION USING DESIGN WEIGHTS FOR THE STRATIFIED CASE-COHORT DESIGN 5](#_Toc152844942)

[B.1 Derivation of the influence functions 5](#_Toc152844943)

[B.2 Comparison with the variance of Samuelsen et al. (2007) 7](#_Toc152844944)

[Web Appendix C. ESTIMATION USING CALIBRATED WEIGHTS FOR THE STRATIFIED CASE-COHORT DESIGN 9](#_Toc152844945)

[C.1 Estimating equation for calibration 9](#_Toc152844946)

[C.2 Derivation of the influence functions 10](#_Toc152844947)

[C.3 Comment on the choice of the auxiliary variables 15](#_Toc152844948)

[Web Appendix D. SIMULATIONS 17](#_Toc152844949)

[D.1 Fixed parameter values used for the simulations in Section 7 of the Main Document 17](#_Toc152844950)

[D.2 Simulations results for all investigated scenarios 17](#_Toc152844951)

[D.3 Comment on the robust variance estimation 18](#_Toc152844952)

[D.4 Estimation of the phase-two component of the variance 37](#_Toc152844953)

[D.5 Additional simulations, with design weights poststratified on the number of non-cases 37](#_Toc152844954)

[D.6 Additional simulations, with weights calibrated using weaker proxies 38](#_Toc152844955)

[Web Appendix E. PHASE-TWO MISSING DATA WHEN THE PHASE-THREE DESIGN SAMPLING PROBABILITIES ARE UNKNOWN 49](#_Toc152844956)

[E.1 Derivation of the influence functions 49](#_Toc152844957)

[E.2 Variance decomposition and estimation 53](#_Toc152844958)

[Web Appendix F. PHASE-TWO MISSING DATA WHEN THE PHASE-THREE DESIGN SAMPLING PROBABILITIES ARE KNOWN 58](#_Toc152844959)

[F.1 Parameters estimation 58](#_Toc152844960)

[F.2 Derivation of the influence functions 59](#_Toc152844961)

[F.3 Variance decomposition and estimation from influence functions 60](#_Toc152844962)

[Web Appendix G. SIMULATIONS WITH MISSING PHASE-TWO DATA 64](#_Toc152844963)

[G.1 Simulation design 64](#_Toc152844964)

[G.2 Simulation results 65](#_Toc152844965)

[Web Appendix H. DATA ANALYSIS 91](#_Toc152844966)

[H.1 Parameter estimation 91](#_Toc152844967)

[Web Appendix I. STEP BY STEP PSEUDO CODE TO OBTAIN $\boldsymbol{V}$ FOR SCC AND SCC.Calib IN TABLE 3 IN THE MAIN DOCUMENT 93](#_Toc152844968)

[DATA AVAILABILITY STATEMENT 95](#_Toc152844969)

[REFERENCES 95](#_Toc152844970)

# ESTIMATION WITH COMPLETE COVARIATE DATA FOR THE WHOLE COHORT

## Parameters estimation

Here we review methods for analyzing cohort data with complete covariates $\boldsymbol{X}$ measured on all cohort members. The estimate of the log-relative hazard, ${\hat{\boldsymbol{\beta}}}_{c}$, is obtained by maximizing the partial log-likelihood

$\sum_{j=1}^{J} \sum_{i=1}^{n^{(j)}} \int_{t} \left[ \log\left\{ Y_{i,j}\left( t \right)\exp\left( \boldsymbol{\beta}^{'}\boldsymbol{X}_{i,j} \right) \right\}-\log\left\{ \sum_{l=1}^{J} \sum_{k=1}^{n^{(l)}} Y_{k,l}\left( t \right)\exp\left( \boldsymbol{\beta}^{'}\boldsymbol{X}_{k,l} \right) \right\} \right]{dN}_{i,j}\left( t \right)$,

or equivalently by solving in $\boldsymbol{\beta}$ the estimating equation

$\boldsymbol{U}_{c}\left( \boldsymbol{\beta} \right)=\sum_{j=1}^{J} \sum_{i=1}^{n^{(j)}} \int_{t} \left\{ \boldsymbol{X}_{i,j} -\frac{\sum_{l=1}^{J} \sum_{k=1}^{n^{(l)}} Y_{k,l}\left( t \right)\exp\left( \boldsymbol{\beta}^{'}\boldsymbol{X}_{k,l} \right)\boldsymbol{X}_{k,l}}{\sum_{l=1}^{J} \sum_{k=1}^{n^{(l)}} Y_{k,l}\left( t \right)\exp\left( \boldsymbol{\beta}^{'}\boldsymbol{X}_{k,l} \right)} \right\}{dN}_{i,j}\left( t \right)=0$.

The baseline hazard point mass at time $t$ is estimated non-parametrically (Breslow, 1974) as ${d\hat{\Lambda}}_{0,c}\left( t;{\hat{\boldsymbol{\beta}}}_{c} \right)\equiv{d\hat{\Lambda}}_{0,c}\left( t \right)=\frac{\sum_{j=1}^{J} \sum_{i=1}^{n^{(j)}} {dN}_{i,j}\left( t \right)}{\sum_{j=1}^{J} \sum_{i=1}^{n^{(j)}} Y_{i,j}\left( t \right)\exp\left( {\hat{\boldsymbol{\beta}}}_{c}^{'}\boldsymbol{X}_{i,j} \right)}$, and the cumulative baseline hazard estimate up to time $t$ is estimated as $\hat{\Lambda}_{0,c}\left( t;{\hat{\boldsymbol{\beta}}}_{c},\hat{\lambda}_{0,c} \right)\equiv\hat{\Lambda}_{0,c}\left( t \right)=\int_{0}^{t} d\hat{\Lambda}_{0,c}\left( s \right)$.

Finally, the pure risk for covariate profile $\boldsymbol{x}$ in ${(\tau}_{1},\tau_{2}]$ (i.e., the probability of experiencing the event within the interval ${(\tau}_{1},\tau_{2}]$ when having profile $\boldsymbol{X}=\boldsymbol{x}$**)**, is estimated as $\hat{\pi}_{c}\left( \tau_{1},\tau_{2}; \boldsymbol{x},{\hat{\boldsymbol{\beta}}}_{c}, \hat{\lambda}_{0,c} \right)\equiv\hat{\pi}_{c}\left( \tau_{1},\tau_{2}; \boldsymbol{x} \right)=1-\exp\left\{ -\int_{\tau_{1}}^{\tau_{2}} \exp\left( {\hat{\boldsymbol{\beta}}}_{c}^{'}\boldsymbol{x} \right)d\hat{\Lambda}_{0,c}\left( t \right) \right\}$.

## Influence functions

Let $\boldsymbol{\Delta}_{i,j}\left( {\hat{\boldsymbol{\theta}}}_{c} \right)$ denote the influence of subject $i$ in stratum $j$ on ${\hat{\boldsymbol{\theta}}}_{c}\in\left\{ {\hat{\boldsymbol{\beta}}}_{c}, {d\hat{\Lambda}}_{0,c}\left( t \right), \hat{\Lambda}_{0,c}\left( t \right),\hat{\pi}_{c}\left( \tau_{1},\tau_{2};\boldsymbol{x} \right) \right\}$, $i\in\{1,\ldots,n^{(j)}\}$,$j\in\left\{ 1,\ldots,J \right\}$. From Reid & Crépeau (1985)

$\boldsymbol{\Delta}_{i,j}\left( {\hat{\boldsymbol{\beta}}}_{c} \right)=\left[ \sum_{l=1}^{J} \sum_{k=1}^{n^{(l)}} \int_{t} \left\{ \frac{\boldsymbol{S}_{2}^{c}\left( t;{\hat{\boldsymbol{\beta}}}_{c} \right)}{S_{0}^{c}\left( t;{\hat{\boldsymbol{\beta}}}_{c} \right)}-\frac{\boldsymbol{S}_{1}^{c}\left( t;{\hat{\boldsymbol{\beta}}}_{c} \right) {\boldsymbol{S}_{1}^{c}\left( t;{\hat{\boldsymbol{\beta}}}_{c} \right)}^{'}}{{S_{0}^{c}\left( t;{\hat{\boldsymbol{\beta}}}_{c} \right)}^{2}} \right\}{dN}_{k,l}\left( t \right) \right]^{-1}\times\left[ \int_{t} \left\{ \boldsymbol{X}_{i,j}-\frac{\boldsymbol{S}_{1}^{c}\left( t;{\hat{\boldsymbol{\beta}}}_{c} \right)}{S_{0}^{c}\left( t;{\hat{\boldsymbol{\beta}}}_{c} \right)} \right\}\times\left\{ {dN}_{i,j}\left( t \right)-Y_{i,j}\left( t \right)\exp\left( {{\hat{\boldsymbol{\beta}}}_{c}}^{'}\boldsymbol{X}_{i,j} \right)\frac{\sum_{l=1}^{J} \sum_{k=1}^{n^{(l)}} {dN}_{k,l}\left( t \right)}{S_{0}\left( t;{\hat{\boldsymbol{\beta}}}_{c} \right)} \right\} \right]$,

with $S_{0}^{c}\left( t;{\hat{\boldsymbol{\beta}}}_{c} \right)=\sum_{l=1}^{J} \sum_{k=1}^{n^{(l)}} Y_{k,l}\left( t \right)\exp\left( {{\hat{\boldsymbol{\beta}}}_{c}}^{'}\boldsymbol{X}_{k,l} \right)$, $\boldsymbol{S}_{1}^{c}\left( t;{\hat{\boldsymbol{\beta}}}_{c} \right)=\sum_{l=1}^{J} \sum_{k=1}^{n^{(l)}} Y_{k,l}\left( t \right)\exp\left( {{\hat{\boldsymbol{\beta}}}_{c}}^{'}\boldsymbol{X}_{k,l} \right)\boldsymbol{X}_{k,l}$,

and $\boldsymbol{S}_{2}^{c}\left( t;{\hat{\boldsymbol{\beta}}}_{c} \right)=\sum_{l=1}^{J} \sum_{k=1}^{n^{(l)}} Y_{k,l}\left( t \right)\exp\left( {{\hat{\boldsymbol{\beta}}}_{c}}^{'}\boldsymbol{X}_{k,l} \right)\boldsymbol{X}_{k,l} \boldsymbol{X}_{k,l}'$. Then, from Chapter 4 in (Pfeiffer & Gail, 2017),

$\Delta_{i,j}\left\{ {d\hat{\Lambda}}_{0,c}\left( t \right) \right\}=\left\{ S_{0}^{c}\left( t;{\hat{\boldsymbol{\beta}}}_{c} \right) \right\}^{-1}\left[ {dN}_{i,j}\left( t \right)-{d\hat{\Lambda}}_{0,c}\left( t \right) Y_{i,j}\left( t \right)\exp\left( {{\hat{\boldsymbol{\beta}}}_{c}}^{'}\boldsymbol{X}_{i,j} \right)-{d\hat{\Lambda}}_{0,c}\left( t \right)\boldsymbol{S}_{1}^{c}\left( t;{\hat{\boldsymbol{\beta}}}_{c} \right)'\boldsymbol{\Delta}_{i,j}\left( {\hat{\boldsymbol{\beta}}}_{c} \right) \right]$.

Note, $\Delta_{i,j}\left\{ {d\hat{\Lambda}}_{0,c}\left( t \right) \right\}$ is a linear combination of the increments ${dN}_{i,j}\left( t \right)$ and ${d\hat{\Lambda}}_{0,c}\left( t \right)$; thus $\Delta_{i,j}\left\{ \int_{\tau_{1}}^{\tau_{2}} {d\hat{\Lambda}}_{0,c}\left( t \right) \right\}=\int_{\tau_{1}}^{\tau_{2}} \Delta_{i,j}\left\{ {d\hat{\Lambda}}_{0,c}\left( t \right) \right\}$. Finally, following Graubard and Fears (2005),

$\Delta_{i,j}\left\{ \hat{\pi}_{c}\left( \tau_{1},\tau_{2};\boldsymbol{x} \right) \right\}=\left\{ {\frac{\partial\hat{\pi}_{c}\left( \tau_{1},\tau_{2};\boldsymbol{x} \right)}{\partial\boldsymbol{\beta}}}_{|\boldsymbol{\beta}={\hat{\boldsymbol{\beta}}}_{c}} \right\}\boldsymbol{\Delta}_{i,j}\left( {\hat{\boldsymbol{\beta}}}_{c} \right)+\left[ {\frac{\partial\hat{\pi}_{c}\left( \tau_{1},\tau_{2};\boldsymbol{x} \right)}{\partial\left\{ \int_{\tau_{1}}^{\tau_{2}} {d\Lambda}_{0}\left( t \right) \right\}}}_{|\Lambda_{0}(t)=\hat{\Lambda}_{0,c}\left( t \right)} \right]\Delta_{i,j}\left\{ \int_{\tau_{1}}^{\tau_{2}} {d\hat{\Lambda}}_{0,c}\left( t \right) \right\}$,

with ${\frac{\partial\hat{\pi}_{c}\left( \tau_{1},\tau_{2};\boldsymbol{x} \right)}{\partial\boldsymbol{\beta}}}_{|\boldsymbol{\beta}={\hat{\boldsymbol{\beta}}}_{c}}=\left\{ \int_{\tau_{1}}^{\tau_{2}} {d\hat{\Lambda}}_{0,c}\left( t \right)\exp\left( {\hat{\boldsymbol{\beta}}}_{c}'\boldsymbol{x} \right) \right\}\exp\left\{ -\int_{\tau_{1}}^{\tau_{2}} {d\hat{\Lambda}}_{0,c}\left( t \right)\exp\left( {\hat{\boldsymbol{\beta}}}_{c}'\boldsymbol{x} \right) \right\}\boldsymbol{x'}$,

$=\left\{ \int_{\tau_{1}}^{\tau_{2}} {d\hat{\Lambda}}_{0,c}\left( t \right)\exp\left( {\hat{\boldsymbol{\beta}}}_{c}'\boldsymbol{x} \right) \right\}\left\{ 1-\hat{\pi}_{c}\left( \tau_{1},\tau_{2};\boldsymbol{x} \right) \right\} \boldsymbol{x}'$,

And ${\frac{\partial\hat{\pi}_{c}\left( \tau_{1},\tau_{2};\boldsymbol{x} \right)}{\partial\left\{ \int_{\tau_{1}}^{\tau_{2}} {d\Lambda}_{0}\left( t \right) \right\}}}_{|\Lambda_{0}(t)=\hat{\Lambda}_{0,c}\left( t \right)}=\exp\left( {\hat{\boldsymbol{\beta}}}_{c}'\boldsymbol{x} \right)\exp\left\{ -\int_{\tau_{1}}^{\tau_{2}} {d\hat{\Lambda}}_{0,c}\left( t \right)\exp\left( {\hat{\boldsymbol{\beta}}}_{c}'\boldsymbol{x} \right) \right\}$,

$=\exp\left( {\hat{\boldsymbol{\beta}}}_{c}'\boldsymbol{x} \right)\left\{ 1-\hat{\pi}_{c}\left( \tau_{1},\tau_{2};\boldsymbol{x} \right) \right\}$.

## Variance estimation from influence functions

Reid and Crépeau (1985) estimated $\mathrm{var}\left( {\hat{\boldsymbol{\beta}}}_{c} \right)$ by $\sum_{j=1}^{J} \sum_{i=1}^{n^{\left( j \right)}} \boldsymbol{\Delta}_{i,j}\left( {\hat{\boldsymbol{\beta}}}_{c} \right){\boldsymbol{\Delta}_{i,j}\left( {\hat{\boldsymbol{\beta}}}_{c} \right)}^{'}$. Note the relation to the “robust variance” proposed by Barlow (1994) for the case-cohort design. Using the whole cohort, we could similarly estimate $\mathrm{var}\left( {\hat{\boldsymbol{\theta}}}_{c} \right)$ by $\sum_{j=1}^{J} \sum_{i=1}^{n^{\left( j \right)}} \boldsymbol{\Delta}_{i,j}\left( {\hat{\boldsymbol{\theta}}}_{c} \right){\boldsymbol{\Delta}_{i,j}\left( {\hat{\boldsymbol{\theta}}}_{c} \right)}^{'}$.

# ESTIMATION USING DESIGN WEIGHTS FOR THE STRATIFIED CASE-COHORT DESIGN

## Derivation of the influence functions

Let $\boldsymbol{\Delta}_{i,j}\left( \hat{\boldsymbol{\theta}} \right)$ denote the influence of subject $i$ in stratum $j$ on $\hat{\boldsymbol{\theta}}$, $i\in\{1,\ldots,n^{(j)}\}$,$j\in\left\{ 1,\ldots,J \right\}$, $\hat{\boldsymbol{\theta}}\in\left\{ \hat{\boldsymbol{\beta}}, {d\hat{\Lambda}}_{0}\left( t \right), \hat{\Lambda}_{0}\left( t \right), \hat{\pi}\left( \tau_{1},\tau_{2};\boldsymbol{x} \right) \right\}$. We can rewrite the estimating equation $\sum_{j=1}^{J} \sum_{i=1}^{n^{(j)}} \int_{t} \left\{ \boldsymbol{X}_{i,j} -\frac{\boldsymbol{S}_{1}\left( t;\hat{\boldsymbol{\beta}} \right)}{S_{0}\left( t;\hat{\boldsymbol{\beta}} \right)} \right\}{dN}_{i,j}\left( t \right)=0$ as

$\boldsymbol{G}_{1}-\int_{t} \frac{\boldsymbol{S}_{1}\left( t;\hat{\boldsymbol{\beta}} \right)}{S_{0}\left( t;\hat{\boldsymbol{\beta}} \right)}\left\{ \sum_{l=1}^{J} \sum_{k=1}^{n^{\left( l \right)}} {\xi_{k,l} w_{k,l} dN}_{k,l}\left( t \right) \right\}=0$,

with $\boldsymbol{G}_{1}=\sum_{j=1}^{J} \sum_{i=1}^{n^{(j)}} \int_{t} \xi_{i,j} w_{i,j}\boldsymbol{X}_{i,j}{dN}_{i,j}\left( t \right)$, because $\xi_{i,j} w_{i,j}=1$ for any subject $i$ in stratum $j$ such that $\int_{t} {dN}_{i,j}\left( t \right)=1$ (i.e., all the cases are included in the phase-two sample and have unit design sampling weights). Following Graubard and Fears (2005), we have

| $\boldsymbol{\Delta}_{i,j}\left\{ \boldsymbol{G}_{1} \right\}+\int_{t} \left[ -\frac{\Delta_{i,j}\left\{ \sum_{l=1}^{J} \sum_{k=1}^{n^{(l)}} {\xi_{k,l} w_{k,l} dN}_{k,l}\left( t \right) \right\}\times\boldsymbol{S}_{1}\left( t;\hat{\boldsymbol{\beta}} \right)}{S_{0}\left( t;\hat{\boldsymbol{\beta}} \right)}-\frac{\left\{ \sum_{l=1}^{J} \sum_{k=1}^{n^{(l)}} {\xi_{k,l} w_{k,l} dN}_{k,l}\left( t \right) \right\}\times\boldsymbol{\Delta}_{i,j}\left\{ \boldsymbol{S}_{1}\left( t;\hat{\boldsymbol{\beta}} \right) \right\}}{S_{0}\left( t;\hat{\boldsymbol{\beta}} \right)}+\frac{\left\{ \sum_{l=1}^{J} \sum_{k=1}^{n^{(l)}} {\xi_{k,l} w_{k,l} dN}_{k,l}\left( t \right) \right\}\times{\boldsymbol{S}_{1}\left( t;\hat{\boldsymbol{\beta}} \right)\times\Delta}_{i,j}\left\{ S_{0}\left( t;\hat{\boldsymbol{\beta}} \right) \right\}}{{S_{0}\left( t;\hat{\boldsymbol{\beta}} \right)}^{2}} \right]=0$, | (1) |
| --- | --- |

with

$\boldsymbol{\Delta}_{i,j}\left\{ \boldsymbol{G}_{1} \right\}=\int_{t} \xi_{i,j} w_{i,j}\boldsymbol{X}_{i,j}{dN}_{i,j}\left( t \right)$,

$\Delta_{i,j}\left\{ \sum_{l=1}^{J} \sum_{k=1}^{n^{(l)}} {\xi_{k,l} w_{k,l} dN}_{k,l}\left( t \right) \right\}={\xi_{i,j} w}_{i,j} {dN}_{i,j}\left( t \right)$,

$\boldsymbol{\Delta}_{i,j}\left\{ \boldsymbol{S}_{1}\left( t;\hat{\boldsymbol{\beta}} \right) \right\}=\xi_{i,j}w_{i,j} Y_{i,j}\left( t \right)\exp\left( {\hat{\boldsymbol{\beta}}}^{'}\boldsymbol{X}_{i,j} \right)\boldsymbol{X}_{i,j}+\left\{ {\frac{\partial\boldsymbol{S}_{1}\left( t,\boldsymbol{\beta} \right)}{\partial\boldsymbol{\beta}}}_{|\boldsymbol{\beta}=\tilde{\boldsymbol{\beta}}} \right\}\boldsymbol{\Delta}_{i,j}\left( \hat{\boldsymbol{\beta}} \right)$,

${\frac{\partial\boldsymbol{S}_{1}\left( t,\boldsymbol{\beta} \right)}{\partial\boldsymbol{\beta}}}_{|\boldsymbol{\beta}=\hat{\boldsymbol{\beta}}}=\sum_{l=1}^{J} \sum_{k=1}^{n^{(l)}} \xi_{k,l}w_{k,l} Y_{k,l}\left( t \right)\exp\left( {\hat{\boldsymbol{\beta}}}^{'}\boldsymbol{X}_{k,l} \right)\boldsymbol{X}_{k,l} \boldsymbol{X}_{k,l}'\equiv\boldsymbol{S}_{2}\left( t;\hat{\boldsymbol{\beta}} \right)$,

$\Delta_{i,j}\left\{ S_{0}\left( t;\hat{\boldsymbol{\beta}} \right) \right\}=\xi_{i,j}w_{i,j} Y_{i,j}\left( t \right)\exp\left( {\hat{\boldsymbol{\beta}}}^{'}\boldsymbol{X}_{i,j} \right)+\left\{ {\frac{\partial S_{0}\left( t;,\boldsymbol{\beta} \right)}{\partial\boldsymbol{\beta}}}_{|\boldsymbol{\beta}=\hat{\boldsymbol{\beta}}} \right\}\boldsymbol{\Delta}_{i,j}\left( \hat{\boldsymbol{\beta}} \right)$,

and ${\frac{\partial S_{0}\left( t;\boldsymbol{\beta} \right)}{\partial\boldsymbol{\beta}}}_{|\boldsymbol{\beta}=\hat{\boldsymbol{\beta}}}=\sum_{l=1}^{J} \sum_{k=1}^{n^{(l)}} \xi_{k,l}w_{k,l} Y_{k,l}\left( t \right)\exp\left( {\hat{\boldsymbol{\beta}}}^{'}\boldsymbol{X}_{k,l} \right)\boldsymbol{X}_{k,l}'=\boldsymbol{S}_{1}\left( t;\hat{\boldsymbol{\beta}} \right)'$.

Note, the term between square brackets in Equation (1) is a linear combination of ${dN}_{i,j}\left( t \right)$. Hence

$\boldsymbol{\Delta}_{i,j}\left( \hat{\boldsymbol{\beta}} \right)=\left[ \sum_{l=1}^{J} \sum_{k=1}^{n^{(l)}} \int_{t} \xi_{k,l} w_{k,l}\left\{ \frac{\boldsymbol{S}_{2}\left( t;\hat{\boldsymbol{\beta}} \right)}{S_{0}\left( t;\hat{\boldsymbol{\beta}} \right)}-\frac{\boldsymbol{S}_{1}\left( t;\hat{\boldsymbol{\beta}} \right){\boldsymbol{S}_{1}\left( t;\hat{\boldsymbol{\beta}} \right)}^{'}}{{S_{0}\left( t;\hat{\boldsymbol{\beta}} \right)}^{2}} \right\}{dN}_{k,l}\left( t \right) \right]^{-1}\times\left[ \int_{t} \left\{ \boldsymbol{X}_{i,j}-\frac{\boldsymbol{S}_{1}\left( t;\hat{\boldsymbol{\beta}} \right)}{S_{0}\left( t;\hat{\boldsymbol{\beta}} \right)} \right\}\times\left\{ {\xi_{i,j} w}_{i,j} {dN}_{i,j}\left( t \right)-\xi_{i,j}w_{i,j}Y_{i,j}\left( t \right)\exp\left( {\hat{\boldsymbol{\beta}}}^{'}\boldsymbol{X}_{i,j} \right)\frac{\sum_{l=1}^{J} \sum_{k=1}^{n^{(l)}} {\xi_{k,l} w_{k,l} dN}_{k,l}\left( t \right)}{S_{0}\left( t;\hat{\boldsymbol{\beta}} \right)} \right\} \right]$,

and we can write $\boldsymbol{\Delta}_{i,j}\left( \hat{\boldsymbol{\beta}} \right)=\xi_{i,j}w_{i,j} \boldsymbol{IF}_{i,j}^{(2)}\left( \hat{\boldsymbol{\beta}} \right)$, with

$\boldsymbol{IF}_{i,j}^{(2)}\left( \hat{\boldsymbol{\beta}} \right)=\left[ \sum_{l=1}^{J} \sum_{k=1}^{n^{(l)}} \int_{t} \xi_{k,l} w_{k,l}\left\{ \frac{\boldsymbol{S}_{2}\left( t;\hat{\boldsymbol{\beta}} \right)}{S_{0}\left( t;\hat{\boldsymbol{\beta}} \right)}-\frac{\boldsymbol{S}_{1}\left( t;\hat{\boldsymbol{\beta}} \right){\boldsymbol{S}_{1}\left( t;\hat{\boldsymbol{\beta}} \right)}^{'}}{{S_{0}\left( t;\hat{\boldsymbol{\beta}} \right)}^{2}} \right\}{dN}_{k,l}\left( t \right) \right]^{-1}\left[ \int_{t} \left\{ \boldsymbol{X}_{i,j}-\frac{\boldsymbol{S}_{1}\left( t;\hat{\boldsymbol{\beta}} \right)}{S_{0}\left( t;\hat{\boldsymbol{\beta}} \right)} \right\}\times\left\{ {dN}_{i,j}\left( t \right)-Y_{i,j}\left( t \right)\exp\left( {\hat{\boldsymbol{\beta}}}^{'}\boldsymbol{X}_{i,j} \right)\frac{\sum_{l=1}^{J} \sum_{k=1}^{n^{(l)}} {\xi_{k,l} w_{k,l} dN}_{k,l}\left( t \right)}{S_{0}\left( t;\hat{\boldsymbol{\beta}} \right)} \right\} \right]$.

Then we know $\sum_{j=1}^{J} \sum_{i=1}^{n^{(j)}} \left\{ {dN}_{i,j}\left( t \right) \right\}-{d\hat{\Lambda}}_{0}\left( t \right)\times S_{0}\left( t;\hat{\boldsymbol{\beta}} \right)=0$, as we estimate the baseline hazard non-parametrically by ${d\hat{\Lambda}}_{0}\left( t \right)=\frac{\sum_{j=1}^{J} \sum_{i=1}^{n^{(j)}} {dN}_{i,j}\left( t \right)}{S_{0}\left( t;\hat{\boldsymbol{\beta}} \right)}$. Thus, following similar arguments as for $\boldsymbol{\Delta}_{i,j}\left( \hat{\boldsymbol{\beta}} \right)$, we have ${dN}_{i,j}\left( t \right)-{d\hat{\Lambda}}_{0}\left( t \right) \Delta_{i,j}\left\{ S_{0}\left( t;\hat{\boldsymbol{\beta}} \right) \right\}-S_{0}\left( t;\hat{\boldsymbol{\beta}} \right) \Delta_{i,j}\left\{ {d\hat{\Lambda}}_{0}\left( t \right) \right\}=0$. In other words,

${dN}_{i,j}\left( t \right)-{{d\hat{\Lambda}}_{0}\left( t \right) \xi}_{i,j} w_{i,j}Y_{i,j}\left( t \right)\exp\left( {\hat{\boldsymbol{\beta}}}^{'}\boldsymbol{X}_{i,j} \right)-{d\hat{\Lambda}}_{0}\left( t \right)\boldsymbol{S}_{1}\left( t;\hat{\boldsymbol{\beta}} \right)'\boldsymbol{\Delta}_{i,j}\left( \hat{\boldsymbol{\beta}} \right)-S_{0}\left( t;\hat{\boldsymbol{\beta}} \right)\Delta_{i,j}\left\{ {d\hat{\Lambda}}_{0}\left( t \right) \right\}=0$,

and as a result $\Delta_{i,j}\left\{ {d\hat{\Lambda}}_{0}\left( t \right) \right\}=\left\{ S_{0}\left( t;\hat{\boldsymbol{\beta}} \right) \right\}^{-1}\left[ {dN}_{i,j}\left( t \right)-{d\hat{\Lambda}}_{0}\left( t \right) \xi_{i,j} w_{i,j} Y_{i,j}\left( t \right)\exp\left( {\hat{\boldsymbol{\beta}}}^{'}\boldsymbol{X}_{i,j} \right)-{d\hat{\Lambda}}_{0}\left( t \right)\boldsymbol{S}_{1}\left( t;\hat{\boldsymbol{\beta}} \right)'\boldsymbol{\Delta}_{i,j}\left( \hat{\boldsymbol{\beta}} \right) \right]$. We can thus write $\Delta_{i,j}\left\{ {d\hat{\Lambda}}_{0}\left( t \right) \right\}=\xi_{i,j}w_{i,j} {IF}_{i,j}^{(2)}\left\{ {d\hat{\Lambda}}_{0}\left( t \right) \right\}$, with ${{IF}_{i,j}^{(2)}\left\{ {d\hat{\Lambda}}_{0}\left( t \right) \right\}=\left\{ S_{0}\left( t;\hat{\boldsymbol{\beta}} \right) \right\}}^{-1}\left\{ {dN}_{i,j}\left( t \right)- {d\hat{\Lambda}}_{0}\left( t \right) Y_{i,j}\left( t \right)\exp\left( {\hat{\boldsymbol{\beta}}}^{'}\boldsymbol{X}_{i,j} \right)- {d\hat{\Lambda}}_{0}\left( t \right){\boldsymbol{S}_{1}\left( t;\hat{\boldsymbol{\beta}} \right)}^{'}\boldsymbol{IF}_{i,j}^{(2)}\left( \hat{\boldsymbol{\beta}} \right) \right\}$, as $\xi_{i,j}w_{i,j}=1$ if ${dN}_{i,j}\left( t \right)=1$.

Both ${IF}_{i,j}^{(2)}\left\{ {d\hat{\Lambda}}_{0}\left( t \right) \right\}$ and $\Delta_{i,j}\left\{ {d\hat{\Lambda}}_{0}\left( t \right) \right\}$ are linear combination of the increments ${dN}_{i,j}\left( t \right)$ and ${d\hat{\Lambda}}_{0}\left( t \right)$. Thus $\Delta_{i,j}\left\{ \int_{\tau_{1}}^{\tau_{2}} {d\hat{\Lambda}}_{0}\left( t \right) \right\}=\int_{\tau_{1}}^{\tau_{2}} \Delta_{i,j}\left\{ {d\hat{\Lambda}}_{0}\left( t \right) \right\}$, that we can rewrite $\xi_{i,j}w_{i,j} {IF}_{i,j}^{(2)}\left\{ \int_{\tau_{1}}^{\tau_{2}} {d\hat{\Lambda}}_{0}\left( t \right) \right\}$, with ${IF}_{i,j}^{(2)}\left\{ \int_{\tau_{1}}^{\tau_{2}} {d\hat{\Lambda}}_{0}\left( t \right) \right\}=\int_{\tau_{1}}^{\tau_{2}} {IF}_{i,j}^{(2)}\left\{ {d\hat{\Lambda}}_{0}\left( t \right) \right\}$. Finally,

$\Delta_{i,j}\left\{ \hat{\pi}\left( \tau_{1},\tau_{2}; \boldsymbol{x} \right) \right\}=\left\{ {\frac{\partial\hat{\pi}\left( \tau_{1},\tau_{2}; \boldsymbol{x} \right)}{\partial\boldsymbol{\beta}}}_{|\boldsymbol{\beta}=\hat{\boldsymbol{\beta}}} \right\}\boldsymbol{\Delta}_{i,j}\left( \hat{\boldsymbol{\beta}} \right)+\left[ {\frac{\partial\hat{\pi}\left( \tau_{1},\tau_{2};\boldsymbol{x} \right)}{\partial\left\{ \int_{\tau_{1}}^{\tau_{2}} {d\Lambda}_{0}\left( t \right) \right\}}}_{|d\Lambda_{0}(t)={d\hat{\Lambda}}_{0}\left( t \right)} \right]\Delta_{i,j}\left\{ \int_{\tau_{1}}^{\tau_{2}} {d\hat{\Lambda}}_{0}\left( t \right) \right\}$,

and we can rewrite $\Delta_{i,j}\left\{ \hat{\pi}\left( \tau_{1},\tau_{2}; \boldsymbol{x} \right) \right\}=\xi_{i,j}w_{i,j} {IF}_{i,j}^{(2)}\left\{ \hat{\pi}\left( \tau_{1},\tau_{2}; \boldsymbol{x} \right) \right\}$ with

${\frac{\partial\hat{\pi}\left( \tau_{1},\tau_{2}; \boldsymbol{x} \right)}{\partial\boldsymbol{\beta}}}_{|\boldsymbol{\beta}=\hat{\boldsymbol{\beta}}}=\left\{ \int_{\tau_{1}}^{\tau_{2}} {d\hat{\Lambda}}_{0}\left( t \right)\exp\left( \hat{\boldsymbol{\beta}}'\boldsymbol{x} \right) \right\}{\exp\left\{ -\int_{\tau_{1}}^{\tau_{2}} {d\hat{\Lambda}}_{0}\left( t \right)\exp\left( \hat{\boldsymbol{\beta}}'\boldsymbol{x} \right) \right\}\boldsymbol{x}}^{'}$,

$=\left\{ \int_{\tau_{1}}^{\tau_{2}} {d\hat{\Lambda}}_{0}\left( t \right)\exp\left( \hat{\boldsymbol{\beta}}'\boldsymbol{x} \right) \right\}\left\{ 1-\hat{\pi}\left( \tau_{1},\tau_{2};\boldsymbol{x} \right) \right\} \boldsymbol{x}'$, and

${\frac{\partial\hat{\pi}\left( \tau_{1},\tau_{2};\boldsymbol{x} \right)}{\partial\int_{\tau_{1}}^{\tau_{2}} {d\Lambda}_{0}\left( t \right)}}_{|{d\Lambda}_{0}(t)={d\hat{\Lambda}}_{0}\left( t \right)}=\exp\left( \hat{\boldsymbol{\beta}}'\boldsymbol{x} \right)\exp\left\{ -\int_{\tau_{1}}^{\tau_{2}} {d\hat{\Lambda}}_{0}\left( t \right)\exp\left( \hat{\boldsymbol{\beta}}'\boldsymbol{x} \right) \right\}$,

$=exp \left( \hat{\boldsymbol{\beta}}'\boldsymbol{x} \right)\left\{ 1-\hat{\pi}\left( \tau_{1},\tau_{2};\boldsymbol{x} \right) \right\}$.

Observe that for any $\hat{\boldsymbol{\theta}}\in\left\{ \hat{\boldsymbol{\beta}}, {d\hat{\Lambda}}_{0}\left( t \right), \hat{\Lambda}_{0}\left( t \right), \hat{\pi}\left( \tau_{1},\tau_{2};\boldsymbol{x} \right) \right\}$*,* if subject $i$ in stratum $j$ is not in the stratified case-cohort, then $\boldsymbol{\Delta}_{i,j}\left( \hat{\boldsymbol{\theta}} \right)$ is zero, $i\in\left\{ 1,\ldots,n^{(j)} \right\}$, $j\in\left\{ 1,\ldots,J \right\}$.

## Comparison with the variance of Samuelsen et al. (2007)

Although they do not mention influence functions, the variance estimate for $\hat{\boldsymbol{\beta}}$ from Equation (14) in Section 3.3 in the Main Document is very close to that of Samuelsen et al. (2007). There are two subtle differences. The first is that Samuelsen et al. (2007), following Borgan et al. (2000), redefined the strata by excluding the cases; we do not, and the non-cases stratum-specific design weights thus depend on the number of individuals sampled in each stratum, not the actual number of non-cases sampled in each stratum. Then, the phase-two component proposed by Samuelsen et al. (2007), with a slight modification of their notation so that it is closer to ours, is

$\sum_{j=1}^{J} \frac{\tilde{m}^{(j)}\left( 1-\frac{\tilde{m}^{(j)}}{\tilde{n}^{(j)}} \right)}{\tilde{m}^{(j)}-1}\sum_{i=1}^{\tilde{n}^{(j)}} \left( \boldsymbol{D}_{i,j}-{\bar{\boldsymbol{D}}}_{j} \right)\left( \boldsymbol{D}_{i,j}-{\bar{\boldsymbol{D}}}_{j} \right)^{'}$,

where $\tilde{n}^{(j)}$ is the size of stratum $j$ after having removed all of the cases, and $\tilde{m}^{(j)}$ is the number of non-cases sampled among the $m^{(j)}$ individuals sampled in stratum $j$. $\boldsymbol{D}_{i,j}$ is the analogue of our $\boldsymbol{\Delta}_{i,j}\left( \hat{\boldsymbol{\beta}} \right)$, and ${\bar{\boldsymbol{D}}}_{j}=\frac{1}{\tilde{m}^{(j)}}\sum_{i=1}^{\tilde{n}^{(j)}} \boldsymbol{D}_{i,j}$. This quantity equals

$$\sum_{j=1}^{J} \frac{\tilde{m}^{(j)}\left( 1-\frac{\tilde{m}^{(j)}}{\tilde{n}^{(j)}} \right)}{\tilde{m}^{(j)}-1}\left( \sum_{i=1}^{\tilde{n}^{(j)}} \boldsymbol{D}_{i,j}{\boldsymbol{D}_{i,j}}^{'}-\frac{1}{\tilde{m}^{(j)}}\sum_{i=1}^{\tilde{n}^{(j)}} \sum_{k=1}^{\tilde{n}^{(j)}} \boldsymbol{D}_{i,j}{\boldsymbol{D}_{k,j}}^{'} \right)$$

$=\sum_{j=1}^{J} \frac{\tilde{m}^{(j)}\left( 1-\frac{\tilde{m}^{(j)}}{\tilde{n}^{(j)}} \right)}{\tilde{m}^{(j)}-1}\left( \frac{\tilde{m}^{(j)}-1}{\tilde{m}^{(j)}}\sum_{i=1}^{\tilde{n}^{(j)}} \boldsymbol{D}_{i,j}{\boldsymbol{D}_{i,j}}^{'}-\frac{1}{\tilde{m}^{(j)}}\sum_{i=1}^{\tilde{n}^{(j)}} \sum_{\begin{aligned} k=1, \\ k\neq i \end{aligned}}^{\tilde{n}^{(j)}} \boldsymbol{D}_{i,j}{\boldsymbol{D}_{k,j}}^{'} \right)$,

$=\sum_{j=1}^{J} \left\{ \left( 1-\frac{\tilde{m}^{(j)}}{\tilde{n}^{(j)}} \right)\sum_{i=1}^{\tilde{n}^{(j)}} \boldsymbol{D}_{i,j}{\boldsymbol{D}_{i,j}}^{'}-\frac{1}{\tilde{m}^{(j)}-1}\left( 1-\frac{\tilde{m}^{(j)}}{\tilde{n}^{(j)}} \right)\sum_{i=1}^{\tilde{n}^{(j)}} \sum_{\begin{aligned} k=1, \\ k\neq i \end{aligned}}^{\tilde{n}^{(j)}} \boldsymbol{D}_{i,j}{\boldsymbol{D}_{k,j}}^{'} \right\}$.

Note that the sum in the phase-two component of the variance reduces to a sum only over the sampled non-cases, but $\left( 1-\frac{\tilde{m}^{(j)}}{\tilde{n}^{(j)}} \right)$ and $\frac{1}{\tilde{m}^{(j)}-1}\left( 1-\frac{\tilde{m}^{(j)}}{\tilde{n}^{(j)}} \right)$ would be $\left( 1-\frac{m^{(j)}}{n^{(j)}} \right)$ and $\frac{1}{m^{(j)}-1}\left( 1-\frac{m^{(j)}}{n^{(j)}} \right)$, respectively, in our Equation (14) in the Main Document.

Second, we use the influences to estimate the phase-one (superpopulation) component of the variance of $\hat{\boldsymbol{\beta}}$, whereas Samuelsen et al. (2007) use the inverse of the observed information matrix. Similarly, our phase-one component of the variance of $\hat{\boldsymbol{\beta}}$ and $\int_{\tau_{1}}^{\tau_{2}} {d\hat{\Lambda}}_{0}\left( t \right)$ differ from that proposed by Lin (2000); see Section 3.3 in the Main Document. Using the influences in this component allows to easily extend the derivation of the variance to other designs and analytic options (e.g., using calibrated weights).

# ESTIMATION USING CALIBRATED WEIGHTS FOR THE STRATIFIED CASE-COHORT DESIGN

## Estimating equation for calibration

Let $\boldsymbol{A}_{i,j}$ be the vector of $q$ auxiliary variables for individual $i$ in stratum $j$, $i\in\left\{ 1,\ldots,n^{(j)} \right\}$, $j\in\left\{ 1,\ldots,J \right\}$. Following Breslow et al. (2009a) and Shin et al. (2020), we obtained the auxiliary variables from (*i*) the influences for the log-relative hazard parameters from the Cox model with imputed cohort data; (*ii*) the products of total follow-up time (on the time interval for which the pure risk is to be estimated) and relative hazard for the imputed cohort data and with the log-relative hazard parameters estimated from the Cox model with stratified case-cohort data and weights calibrated with (*i*); and (*iii*) a variable that is identically equal to 1 for every individual in the cohort. The imputed cohort data is obtained from using phase-one data to impute covariate values (for covariates measured only in phase-two) for all members of the cohort. See Breslow et al. (2009a) and Shin et al. (2020) for details on the construction of the auxiliary variables.

We want to use the calibrated weights $\left( w_{i,j}^{*} \right)_{i\in\left\{ 1,\ldots,n^{(j)} \right\}, j\in\left\{ 1,\ldots,J \right\}}$ that are as close as possible to the design weights $\left( w_{i,j} \right)_{i\in\left\{ 1,\ldots,n^{(j)} \right\}, j\in\left\{ 1,\ldots,J \right\}}$, and such that we correctly estimate the totals of the auxiliary variables (i.e., such that $\sum_{j=1}^{J} \sum_{i=1}^{n^{(j)}} \xi_{i,j} w_{i,j}^{*}\boldsymbol{A}_{i,j}=\sum_{j=1}^{J} \sum_{i=1}^{n^{(j)}} \boldsymbol{A}_{i,j}$). In other words, we want to minimize $\sum_{j=1}^{J} \sum_{i=1}^{n^{(j)}} \xi_{i,j} \delta(w_{i,j}^{*},w_{i,j})$, for $\delta$ a given distance, under the constraint $\sum_{j=1}^{J} \sum_{i=1}^{n^{(j)}} {(\xi}_{i,j} w_{i,j}^{*}\boldsymbol{A}_{i,j}-\boldsymbol{A}_{i,j})=0$. To solve this constrained optimization problem, we consider $\boldsymbol{\eta}$, a vector of $q$ Lagrange multipliers, and look for a stationary point of the Lagrangian function $L:\left( w_{1,1}^{*},\ldots,w_{n^{(J)},J}^{*}, \eta\right)⟼\sum_{j=1}^{J} \sum_{i=1}^{n^{(J)}} {\{\xi}_{i,j} \delta(w_{i,j}^{*}, w_{i,j})+ \boldsymbol{\eta}{{'(\xi}_{i,j} w_{i,j}^{*}\boldsymbol{A}}_{i,j}-\boldsymbol{A}_{i,j})\}$*.* To do so, we solve the $n+1$ following equations, obtained from the gradient of $L$:

$$\left\{ \begin{aligned} \xi_{1,1}\left\{ \frac{\partial\delta(w_{1,1}^{*}, w_{1,1})}{\partial w_{1,1}^{*}}+\boldsymbol{\eta}'\boldsymbol{A}_{1,1} \right\}=0, \\ \vdots\\ \begin{matrix} \xi_{n^{(J)},J}\left\{ \frac{\partial\delta(w_{n^{(J)},J}^{*}, w_{n^{(J)},J})}{\partial w_{n^{(J)},J}^{*}}+\boldsymbol{\eta}'\boldsymbol{A}_{n^{(J)},J} \right\}=0, \\ \sum_{j=1}^{J} \sum_{i=1}^{n^{(j)}} {(\xi}_{i,j} {w_{i,j}}^{*}\boldsymbol{A}_{i,j}-\boldsymbol{A}_{i,j})=0. \end{matrix} \end{aligned} \right.$$

We use $\delta:\left( a,b \right)⟼a\log\left( \frac{a}{b} \right)+b-a$ as distance measure, which leads to the raking procedure (Breslow et al., 2009; Deville & Sarndal, 1992) and to solving the estimating equation

$\sum_{j=1}^{J} \sum_{i=1}^{n^{(j)}} \left\{ \xi_{i,j} w_{i,j}\exp\left( \boldsymbol{\eta}^{'}\boldsymbol{A}_{i,j} \right)\boldsymbol{A}_{i,j}-\boldsymbol{A}_{i,j} \right\}=0$.

From the estimate $\hat{\boldsymbol{\eta}}$**,** we then obtain the calibrated weights $w_{i,j}^{*}=w_{i,j}\exp(\hat{\boldsymbol{\eta}}'\boldsymbol{A}_{i,j})$, $i\in\left\{ 1,\ldots,n^{(j)} \right\}$, $j\in\left\{ 1,\ldots,J \right\}$.

## Derivation of the influence functions

Let $\boldsymbol{\Delta}_{i,j}\left( {\hat{\boldsymbol{\theta}}}^{*} \right)$ denote the influence of subject $i$ in stratum $j$ on ${\hat{\boldsymbol{\theta}}}^{*}$, $i\in\{1,\ldots,n^{(j)}\}$,$j\in\left\{ 1,\ldots,J \right\}$, ${\hat{\boldsymbol{\theta}}}^{*}\in\left\{ \hat{\boldsymbol{\eta}},{\hat{\boldsymbol{\beta}}}^{*}, d\hat{\Lambda}_{0}^{*}\left( t \right), \hat{\Lambda}_{0}^{*}\left( t \right), \hat{\pi}^{*}\left( \tau_{1},\tau_{2};\boldsymbol{x} \right) \right\}$. As $\sum_{j=1}^{J} \sum_{i=1}^{n^{(j)}} \left\{ \xi_{i,j} w_{i,j}\exp\left( {\hat{\boldsymbol{\eta}}}^{'}\boldsymbol{A}_{i,j} \right)\boldsymbol{A}_{i,j}-\boldsymbol{A}_{i,j} \right\}=0$, then, following Graubard and Fears (2005)

$\boldsymbol{A}_{i,j}-\xi_{i,j} w_{i,j}\exp\left( {\hat{\boldsymbol{\eta}}}^{'}\boldsymbol{A}_{i,j} \right)\boldsymbol{A}_{i,j}-$ $\left\{ \sum_{l=1}^{J} \sum_{k=1}^{n^{(l)}} \xi_{k,l} w_{k,l}\exp\left( {\hat{\boldsymbol{\eta}}}^{'}\boldsymbol{A}_{k,l} \right) \boldsymbol{A}_{k,l}\boldsymbol{A}_{k,l}' \right\}\boldsymbol{\Delta}_{i,j}\left( \hat{\boldsymbol{\eta}} \right)=0$,

and as a result

$\boldsymbol{\Delta}_{i,j}\left( \hat{\boldsymbol{\eta}} \right)=\left\{ \sum_{l=1}^{J} \sum_{k=1}^{n^{(l)}} \xi_{k,l} w_{k,l}\exp\left( {\hat{\boldsymbol{\eta}}}^{'}\boldsymbol{A}_{k,l} \right) \boldsymbol{A}_{k,l}\boldsymbol{A}_{k,l}' \right\}^{-1}\left\{ \boldsymbol{A}_{i,j}-\xi_{i,j} w_{i,j}\exp\left( {\hat{\boldsymbol{\eta}}}^{'}\boldsymbol{A}_{i,j} \right)\boldsymbol{A}_{i,j} \right\}$.

We can then write

$\boldsymbol{\Delta}_{i,j}\left( \hat{\boldsymbol{\eta}} \right)=\boldsymbol{IF}_{i,j}^{(1)}\left( \hat{\boldsymbol{\eta}} \right)+\xi_{i,j}w_{i,j} \boldsymbol{IF}_{i,j}^{(2)}\left( \hat{\boldsymbol{\eta}} \right)$,

with $\boldsymbol{IF}_{i,j}^{(1)}\left( \hat{\boldsymbol{\eta}} \right)=\left\{ \sum_{l=1}^{J} \sum_{k=1}^{n^{\left( j \right)}} \xi_{k,l} w_{k,l}\exp\left( {\hat{\boldsymbol{\eta}}}^{'}\boldsymbol{A}_{k,l} \right) \boldsymbol{A}_{k,l}\boldsymbol{A}_{k,l}' \right\}^{-1}\boldsymbol{A}_{i,j}$,

and $\boldsymbol{IF}_{i,j}^{(2)}\left( \hat{\boldsymbol{\eta}} \right)={-\exp\left( {\hat{\boldsymbol{\eta}}}^{'}\boldsymbol{A}_{i,j} \right)\left\{ \sum_{l=1}^{J} \sum_{k=1}^{n^{\left( j \right)}} \xi_{k,l} w_{k,l}\exp\left( {\hat{\boldsymbol{\eta}}}^{'}\boldsymbol{A}_{k,l} \right) \boldsymbol{A}_{k,l}\boldsymbol{A}_{k,l}' \right\}}^{-1}\boldsymbol{A}_{i,j}$.

Then we can write the estimating equation

$\sum_{j=1}^{J} \sum_{i=1}^{n^{\left( j \right)}} \int_{t} \xi_{i,j} w_{i,j}\exp\left( {\hat{\boldsymbol{\eta}}}^{'}\boldsymbol{A}_{i,j} \right)\left\{ \boldsymbol{X}_{i,j} -\frac{\boldsymbol{S}_{1}^{*}\left( t;\hat{\boldsymbol{\eta}},{\hat{\boldsymbol{\beta}}}^{*} \right)}{S_{0}^{*}\left( t;\hat{\boldsymbol{\eta}},{\hat{\boldsymbol{\beta}}}^{*} \right)} \right\}{dN}_{i,j}\left( t \right)=0$,

as

$\boldsymbol{G}_{1}^{\boldsymbol{*}}\left( \hat{\boldsymbol{\eta}} \right)-\int_{t} \frac{\boldsymbol{S}_{1}^{*}\left( t;\hat{\boldsymbol{\eta}},{\hat{\boldsymbol{\beta}}}^{*} \right)}{S_{0}^{*}\left( t;\hat{\boldsymbol{\eta}},{\hat{\boldsymbol{\beta}}}^{*} \right)}\left\{ \sum_{l=1}^{J} \sum_{k=1}^{n^{\left( l \right)}} {\xi_{k,l} w_{k,l}\exp\left( {\hat{\boldsymbol{\eta}}}^{'}\boldsymbol{A}_{k,l} \right) dN}_{k,l}\left( t \right) \right\}=0$,

with $\boldsymbol{G}_{1}^{\boldsymbol{*}}\left( \hat{\boldsymbol{\eta}} \right)=\sum_{j=1}^{J} \sum_{i=1}^{n^{(j)}} \int_{t} {\xi_{i,j} w_{i,j}\exp\left( {\hat{\boldsymbol{\eta}}}^{'}\boldsymbol{A}_{i,j} \right)dN}_{i,j}\left( t \right)\boldsymbol{X}_{i,j}$.

Following similar arguments as for $\boldsymbol{\Delta}_{i,j}\left( \hat{\boldsymbol{\eta}} \right)$, we have

| $\boldsymbol{\Delta}_{i,j}\left\{ \boldsymbol{G}_{1}^{\boldsymbol{*}}\left( \hat{\boldsymbol{\eta}} \right) \right\}+\int_{t} \left[ -\frac{\Delta_{i,j}\left\{ \sum_{l=1}^{J} \sum_{k=1}^{n^{\left( l \right)}} {\xi_{k,l} w_{k,l}\exp\left( {\hat{\boldsymbol{\eta}}}^{'}\boldsymbol{A}_{k,l} \right) dN}_{k,l}\left( t \right) \right\}\times\boldsymbol{S}_{1}^{*}\left( t;\hat{\boldsymbol{\eta}},{\hat{\boldsymbol{\beta}}}^{*} \right)}{S_{0}^{*}\left( t;\hat{\boldsymbol{\eta}},{\hat{\boldsymbol{\beta}}}^{*} \right)}-\frac{\left\{ \sum_{l=1}^{J} \sum_{k=1}^{n^{\left( l \right)}} {\xi_{k,l} w_{k,l}\exp\left( {\hat{\boldsymbol{\eta}}}^{'}\boldsymbol{A}_{k,l} \right) dN}_{k,l}\left( t \right) \right\}\times\boldsymbol{\Delta}_{i,j}\left\{ \boldsymbol{S}_{1}^{*}\left( t;\hat{\boldsymbol{\eta}},{\hat{\boldsymbol{\beta}}}^{*} \right) \right\}}{S_{0}^{*}\left( t;\hat{\boldsymbol{\eta}},{\hat{\boldsymbol{\beta}}}^{*} \right)}+\frac{\left\{ \sum_{l=1}^{J} \sum_{k=1}^{n^{\left( l \right)}} {\xi_{k,l} w_{k,l}\exp\left( {\hat{\boldsymbol{\eta}}}^{'}\boldsymbol{A}_{k,l} \right) dN}_{k,l}\left( t \right) \right\}\times{\boldsymbol{S}_{1}^{*}\left( t;\hat{\boldsymbol{\eta}},{\hat{\boldsymbol{\beta}}}^{*} \right)\times\Delta}_{i,j}\left\{ S_{0}^{*}\left( t;\hat{\boldsymbol{\eta}},{\hat{\boldsymbol{\beta}}}^{*} \right) \right\}}{{S_{0}^{*}\left( t;\hat{\boldsymbol{\eta}},{\hat{\boldsymbol{\beta}}}^{*} \right)}^{2}} \right]=0$, | (2) |
| --- | --- |

with

$\boldsymbol{\Delta}_{i,j}\left\{ \boldsymbol{G}_{1}^{\boldsymbol{*}}\left( \hat{\boldsymbol{\eta}} \right) \right\}=\int_{t} \xi_{i,j} w_{i,j}\exp\left( {\hat{\boldsymbol{\eta}}}^{'}\boldsymbol{A}_{i,j} \right){dN}_{i,j}\left( t \right) \boldsymbol{X}_{i,j}+\left\{ {\frac{\partial\boldsymbol{G}_{1}^{\boldsymbol{*}}\left( \boldsymbol{\eta} \right)}{\partial\boldsymbol{\eta}}}_{|\boldsymbol{\eta}=\hat{\boldsymbol{\eta}}} \right\}\boldsymbol{\Delta}_{i,j}\left( \hat{\boldsymbol{\eta}} \right)$,

$\Delta_{i,j}\left\{ \sum_{l=1}^{J} \sum_{k=1}^{n^{\left( l \right)}} {\xi_{k,l} w_{k,l}\exp\left( {\hat{\boldsymbol{\eta}}}^{'}\boldsymbol{A}_{k,l} \right) dN}_{k,l}\left( t \right) \right\}=\xi_{i,j} w_{i,j}\exp\left( {\hat{\boldsymbol{\eta}}}^{'}\boldsymbol{A}_{i,j} \right){dN}_{i,j}\left( t \right)+\left[ {\frac{\partial\left\{ \sum_{l=1}^{J} \sum_{k=1}^{n^{\left( l \right)}} {\xi_{k,l} w_{k,l}\exp\left( \boldsymbol{\eta}^{'}\boldsymbol{A}_{k,l} \right) dN}_{k,l}\left( t \right) \right\}}{\partial\boldsymbol{\eta}}}_{|\boldsymbol{\eta}=\hat{\boldsymbol{\eta}}} \right]\boldsymbol{\Delta}_{i,j}\left( \hat{\boldsymbol{\eta}} \right)$,

${\frac{\partial\boldsymbol{G}_{1}^{\boldsymbol{*}}\left( \boldsymbol{\eta} \right)}{\partial\boldsymbol{\eta}}}_{|\boldsymbol{\eta}=\hat{\boldsymbol{\eta}}}=\sum_{l=1}^{J} \sum_{k=1}^{n^{(l)}} \int_{t} \xi_{k,l}w_{k,l}\exp({\hat{\boldsymbol{\eta}}}^{'}\boldsymbol{A}_{k,l})\boldsymbol{X}_{k,l}\boldsymbol{A}_{k,l}'{dN}_{k,l}\left( t \right)$,

${\frac{\partial\left\{ \sum_{l=1}^{J} \sum_{k=1}^{n^{\left( l \right)}} {\xi_{k,l} w_{k,l}\exp\left( \boldsymbol{\eta}^{'}\boldsymbol{A}_{k,l} \right) dN}_{k,l}\left( t \right) \right\}}{\partial\boldsymbol{\eta}}}_{|\boldsymbol{\eta}=\hat{\boldsymbol{\eta}}}=\sum_{l=1}^{J} \sum_{k=1}^{n^{(l)}} \xi_{k,l}w_{k,l}\exp({\hat{\boldsymbol{\eta}}}^{'}\boldsymbol{A}_{k,l})\boldsymbol{A}_{k,l}'{dN}_{k,l}\left( t \right)$,

$\boldsymbol{\Delta}_{i,j}\left\{ \boldsymbol{S}_{1}^{*}\left( t;\hat{\boldsymbol{\eta}},{\hat{\boldsymbol{\beta}}}^{*} \right) \right\}=\xi_{i,j} w_{i,j}\exp\left( {\hat{\boldsymbol{\eta}}}^{'}\boldsymbol{A}_{i,j} \right)Y_{i,j}\left( t \right)\exp\left( {{\hat{\boldsymbol{\beta}}}^{*}}^{'}\boldsymbol{X}_{i,j} \right)\boldsymbol{X}_{i,j}+\left\{ {\frac{\partial\boldsymbol{S}_{1}^{*}\left( t;\hat{\boldsymbol{\eta}},\boldsymbol{\beta} \right)}{\partial\boldsymbol{\beta}}}_{|\boldsymbol{\beta}={\hat{\boldsymbol{\beta}}}^{*}} \right\}\boldsymbol{\Delta}_{i,j}\left( \tilde{\boldsymbol{\beta}} \right)+\left\{ {\frac{\partial\boldsymbol{S}_{1}^{*}\left( t;\boldsymbol{\eta},{\hat{\boldsymbol{\beta}}}^{*} \right)}{\partial\boldsymbol{\eta}}}_{|\boldsymbol{\eta}=\hat{\boldsymbol{\eta}}} \right\}\boldsymbol{\Delta}_{i,j}\left( \hat{\boldsymbol{\eta}} \right)$,

${\frac{\partial\boldsymbol{S}_{1}^{*}\left( t;\hat{\boldsymbol{\eta}},\boldsymbol{\beta} \right)}{\partial\boldsymbol{\beta}}}_{|\boldsymbol{\beta}={\hat{\boldsymbol{\beta}}}^{*}}=\sum_{l=1}^{J} \sum_{k=1}^{n^{(l)}} \xi_{k,l}w_{k,l}\exp({\hat{\boldsymbol{\eta}}}^{'}\boldsymbol{A}_{k,l})Y_{k,l}\left( t \right)\exp\left( {{\hat{\boldsymbol{\beta}}}^{*}}^{'}\boldsymbol{X}_{k,l} \right)\boldsymbol{X}_{k,l} \boldsymbol{X}_{k,l}'\equiv\boldsymbol{S}_{2}^{*}\left( t;\hat{\boldsymbol{\eta}},{\hat{\boldsymbol{\beta}}}^{*} \right)$,

${\frac{\partial\boldsymbol{S}_{1}^{*}\left( t;\boldsymbol{\eta},{\hat{\boldsymbol{\beta}}}^{*} \right)}{\partial\boldsymbol{\eta}}}_{|\boldsymbol{\eta}=\hat{\boldsymbol{\eta}}}=\sum_{l=1}^{J} \sum_{k=1}^{n^{(l)}} \xi_{k,l}w_{k,l}\exp({\hat{\boldsymbol{\eta}}}^{'}\boldsymbol{A}_{k,l})Y_{k,l}\left( t \right)\exp\left( {{\hat{\boldsymbol{\beta}}}^{*}}^{'}\boldsymbol{X}_{k,l} \right)\boldsymbol{X}_{k,l} \boldsymbol{A}_{k,l}'$,

$\Delta_{i,j}\left\{ S_{0}^{*}\left( t;\hat{\boldsymbol{\eta}},{\hat{\boldsymbol{\beta}}}^{*} \right) \right\}=\xi_{i,j} w_{i,j}\exp\left( {\hat{\boldsymbol{\eta}}}^{'}\boldsymbol{A}_{i,j} \right)Y_{i,j}\left( t \right)\exp\left( {{\hat{\boldsymbol{\beta}}}^{*}}^{'}\boldsymbol{X}_{i,j} \right)+\left\{ {\frac{\partial S_{0}^{*}\left( t;\hat{\boldsymbol{\eta}},\boldsymbol{\beta} \right)}{\partial\boldsymbol{\beta}}}_{|\boldsymbol{\beta}={\hat{\boldsymbol{\beta}}}^{*}} \right\}\boldsymbol{\Delta}_{i,j}\left( {\hat{\boldsymbol{\beta}}}^{*} \right)+\left\{ {\frac{\partial S_{0}^{*}\left( t;\boldsymbol{\eta},{\hat{\boldsymbol{\beta}}}^{*} \right)}{\partial\boldsymbol{\eta}}}_{|\boldsymbol{\eta}=\hat{\boldsymbol{\eta}}} \right\}\boldsymbol{\Delta}_{i,j}\left( \hat{\boldsymbol{\eta}} \right)$,

${\frac{\partial S_{0}^{*}\left( t;\hat{\boldsymbol{\eta}},\boldsymbol{\beta} \right)}{\partial\boldsymbol{\beta}}}_{|\boldsymbol{\beta}={\hat{\boldsymbol{\beta}}}^{*}}=\sum_{l=1}^{J} \sum_{k=1}^{n^{(l)}} \xi_{k,l}w_{k,l}\exp\left( {\hat{\boldsymbol{\eta}}}^{'}\boldsymbol{A}_{k,l} \right)Y_{k,l}\left( t \right)\exp\left( {{\hat{\boldsymbol{\beta}}}^{*}}^{'}\boldsymbol{X}_{k,l} \right)\boldsymbol{X}_{k,l}'=\boldsymbol{S}_{1}^{*}\left( t;\hat{\boldsymbol{\eta}},{\hat{\boldsymbol{\beta}}}^{*} \right)'$,

and ${\frac{\partial S_{0}^{*}\left( t;\boldsymbol{\eta},{\hat{\boldsymbol{\beta}}}^{*} \right)}{\partial\boldsymbol{\eta}}}_{|\boldsymbol{\eta}=\hat{\boldsymbol{\eta}}}=\sum_{l=1}^{J} \sum_{k=1}^{n^{(l)}} \xi_{k,l}w_{k,l}\exp({\hat{\boldsymbol{\eta}}}^{'}\boldsymbol{A}_{k,l})Y_{k,l}\left( t \right)\exp\left( {{\hat{\boldsymbol{\beta}}}^{*}}^{'}\boldsymbol{X}_{k,l} \right)\boldsymbol{A}_{k,l}'$.

Note, $\sum_{l=1}^{J} \sum_{k=1}^{n^{\left( l \right)}} {\xi_{k,l} w_{k,l}\exp\left( {\hat{\boldsymbol{\eta}}}^{'}\boldsymbol{A}_{k,l} \right) dN}_{k,l}\left( t \right)$, ${\frac{\partial\left\{ \sum_{l=1}^{J} \sum_{k=1}^{n^{\left( l \right)}} {\xi_{k,l} w_{k,l}\exp\left( \boldsymbol{\eta}^{'}\boldsymbol{A}_{k,l} \right) dN}_{k,l}\left( t \right) \right\}}{\partial\boldsymbol{\eta}}}_{|\boldsymbol{\eta}=\hat{\boldsymbol{\eta}}}$ and thus $\Delta_{i,j}\left\{ \sum_{l=1}^{J} \sum_{k=1}^{n^{\left( l \right)}} {\xi_{k,l} w_{k,l}\exp\left( {\hat{\boldsymbol{\eta}}}^{'}\boldsymbol{A}_{k,l} \right) dN}_{k,l}\left( t \right) \right\}$ and the component between square brackets in Equation (2) above are linear combinations of the increments ${dN}_{i,j}$. Thus

$\boldsymbol{\Delta}_{i,j}\left( {\hat{\boldsymbol{\beta}}}^{*} \right)=\left[ \sum_{l=1}^{J} \sum_{k=1}^{n^{\left( l \right)}} \int_{t} \xi_{k,l} w_{k,l}\exp\left( {\hat{\boldsymbol{\eta}}}^{'}\boldsymbol{A}_{k,l} \right)\left\{ \frac{\boldsymbol{S}_{2}^{*}\left( t;\hat{\boldsymbol{\eta}},{\hat{\boldsymbol{\beta}}}^{*} \right)}{S_{0}^{*}\left( t;\hat{\boldsymbol{\eta}},{\hat{\boldsymbol{\beta}}}^{*} \right)}-\frac{\boldsymbol{S}_{1}^{*}\left( t;\hat{\boldsymbol{\eta}},{\hat{\boldsymbol{\beta}}}^{*} \right){\boldsymbol{S}_{1}^{*}\left( t;\hat{\boldsymbol{\eta}},{\hat{\boldsymbol{\beta}}}^{*} \right)}^{'}}{{S_{0}^{*}\left( t;\hat{\boldsymbol{\eta}},{\hat{\boldsymbol{\beta}}}^{*} \right)}^{2}} \right\}{dN}_{k,l}\left( t \right) \right]^{-1}\times\left[ \int_{t} \left\{ \boldsymbol{X}_{i,j}-\frac{\boldsymbol{S}_{1}^{*}\left( t;\hat{\boldsymbol{\eta}},{\hat{\boldsymbol{\beta}}}^{*} \right)}{S_{0}^{*}\left( t;\hat{\boldsymbol{\eta}},{\hat{\boldsymbol{\beta}}}^{*} \right)} \right\}\times\left\{ \xi_{i,j} w_{i,j}\exp\left( {\hat{\boldsymbol{\eta}}}^{'}\boldsymbol{A}_{i,j} \right){dN}_{i,j}\left( t \right)-\xi_{i,j} w_{i,j}\exp\left( {\hat{\boldsymbol{\eta}}}^{'}\boldsymbol{A}_{i,j} \right)Y_{i,j}\left( t \right)\exp\left( {{\hat{\boldsymbol{\beta}}}^{*}}^{'}\boldsymbol{X}_{i,j} \right)\frac{\sum_{l=1}^{J} \sum_{k=1}^{n^{\left( l \right)}} {\xi_{k,l} w_{k,l}\exp\left( {\hat{\boldsymbol{\eta}}}^{'}\boldsymbol{A}_{k,l} \right) dN}_{k,l}\left( t \right)}{S_{0}^{*}\left( t;\hat{\boldsymbol{\eta}},{\hat{\boldsymbol{\beta}}}^{*} \right)} \right\} \right]+\left[ \sum_{l=1}^{J} \sum_{k=1}^{n^{\left( l \right)}} \int_{t} \xi_{k,l} w_{k,l}\exp\left( {\hat{\boldsymbol{\eta}}}^{'}\boldsymbol{A}_{k,l} \right)\left\{ \frac{\boldsymbol{S}_{2}^{*}\left( t;\hat{\boldsymbol{\eta}},{\hat{\boldsymbol{\beta}}}^{*} \right)}{S_{0}^{*}\left( t;\hat{\boldsymbol{\eta}},{\hat{\boldsymbol{\beta}}}^{*} \right)}-\frac{\boldsymbol{S}_{1}^{*}\left( t;\hat{\boldsymbol{\eta}},{\hat{\boldsymbol{\beta}}}^{*} \right){\boldsymbol{S}_{1}^{*}\left( t;\hat{\boldsymbol{\eta}},{\hat{\boldsymbol{\beta}}}^{*} \right)}^{'}}{{S_{0}^{*}\left( t;\hat{\boldsymbol{\eta}},{\hat{\boldsymbol{\beta}}}^{*} \right)}^{2}} \right\}{dN}_{k,l}\left( t \right) \right]^{-1}\times\left( \left\{ {\frac{\partial\boldsymbol{G}_{1}^{\boldsymbol{*}}\left( \boldsymbol{\eta} \right)}{\partial\boldsymbol{\eta}}}_{|\boldsymbol{\eta}=\hat{\boldsymbol{\eta}}} \right\}+-\int_{t} \frac{\boldsymbol{S}_{1}^{*}\left( t;\hat{\boldsymbol{\eta}},{\hat{\boldsymbol{\beta}}}^{*} \right)}{S_{0}^{*}\left( t;\hat{\boldsymbol{\eta}},{\hat{\boldsymbol{\beta}}}^{*} \right)}\times\left[ {\frac{\partial\left\{ \sum_{l=1}^{J} \sum_{k=1}^{n^{\left( l \right)}} {\xi_{k,l} w_{k,l}\exp\left( \boldsymbol{\eta}^{'}\boldsymbol{A}_{k,l} \right) dN}_{k,l}\left( t \right) \right\}}{\partial\boldsymbol{\eta}}}_{|\boldsymbol{\eta}=\hat{\boldsymbol{\eta}}} \right]-\int_{t} \frac{\left\{ \sum_{l=1}^{J} \sum_{k=1}^{n^{\left( l \right)}} {\xi_{k,l} w_{k,l}\exp\left( {\hat{\boldsymbol{\eta}}}^{'}\boldsymbol{A}_{k,l} \right) dN}_{k,l}\left( t \right) \right\}}{S_{0}^{*}\left( t;\hat{\boldsymbol{\eta}},{\hat{\boldsymbol{\beta}}}^{*} \right)}\times\left\{ {\frac{\partial\boldsymbol{S}_{1}^{*}\left( t;\boldsymbol{\eta},{\hat{\boldsymbol{\beta}}}^{*} \right)}{\partial\boldsymbol{\eta}}}_{|\boldsymbol{\eta}=\hat{\boldsymbol{\eta}}} \right\}+\int_{t} \frac{\left\{ \sum_{l=1}^{J} \sum_{k=1}^{n^{\left( l \right)}} {\xi_{k,l} w_{k,l}\exp\left( {\hat{\boldsymbol{\eta}}}^{'}\boldsymbol{A}_{k,l} \right) dN}_{k,l}\left( t \right) \right\} {\tilde{\boldsymbol{S}}}_{1}\left( t;\tilde{\boldsymbol{\gamma}},\tilde{\boldsymbol{\beta}} \right)}{{S_{0}^{*}\left( t;\hat{\boldsymbol{\eta}},{\hat{\boldsymbol{\beta}}}^{*} \right)}^{2}}\times\left\{ {\frac{\partial S_{0}^{*}\left( t;\boldsymbol{\eta},{\hat{\boldsymbol{\beta}}}^{*} \right)}{\partial\boldsymbol{\eta}}}_{|\boldsymbol{\eta}=\hat{\boldsymbol{\eta}}} \right\} \right)\boldsymbol{\Delta}_{i,j}\left( \hat{\boldsymbol{\eta}} \right)$,

that we can write as $\boldsymbol{\Delta}_{i,j}\left( {\hat{\boldsymbol{\beta}}}^{*} \right)=\boldsymbol{IF}_{i,j}^{(1)}\left( {\hat{\boldsymbol{\beta}}}^{*} \right)+\xi_{i,j}w_{i,j} \boldsymbol{IF}_{i,j}^{(2)}\left( {\hat{\boldsymbol{\beta}}}^{*} \right)$, with

$\boldsymbol{IF}_{i,j}^{(1)}\left( {\hat{\boldsymbol{\beta}}}^{*} \right)=$ $\left\{ \sum_{l=1}^{J} \sum_{k=1}^{n^{\left( j \right)}} \xi_{k,l} w_{k,l}\exp\left( {\hat{\boldsymbol{\eta}}}^{'}\boldsymbol{A}_{k,l} \right)\boldsymbol{Z}_{k,l}\boldsymbol{A}_{k,l}' \right\}\times\boldsymbol{IF}_{i,j}^{(1)}\left( \hat{\boldsymbol{\eta}} \right)$, and

$\boldsymbol{IF}_{i,j}^{(2)}\left( {\hat{\boldsymbol{\beta}}}^{*} \right)=\exp\left( {\hat{\boldsymbol{\eta}}}^{'}\boldsymbol{A}_{i,j} \right)\boldsymbol{Z}_{i,j}+\left\{ \sum_{l=1}^{J} \sum_{k=1}^{n^{\left( j \right)}} \xi_{k,l} w_{k,l}\exp\left( {\hat{\boldsymbol{\eta}}}^{'}\boldsymbol{A}_{k,l} \right)\boldsymbol{Z}_{k,l}\boldsymbol{A}_{k,l}' \right\}\times\boldsymbol{IF}_{i,j}^{(2)}\left( \hat{\boldsymbol{\eta}} \right)$,

Where

$\boldsymbol{Z}_{i,j}=\left[ \sum_{l=1}^{J} \sum_{k=1}^{n^{\left( l \right)}} \int_{t} \xi_{k,l} w_{k,l}\exp\left( {\hat{\boldsymbol{\eta}}}^{'}\boldsymbol{A}_{k,l} \right)\left\{ \frac{\boldsymbol{S}_{2}^{*}\left( t;\hat{\boldsymbol{\eta}},{\hat{\boldsymbol{\beta}}}^{*} \right)}{S_{0}^{*}\left( t;\hat{\boldsymbol{\eta}},{\hat{\boldsymbol{\beta}}}^{*} \right)}-\frac{\boldsymbol{S}_{1}^{*}\left( t;\hat{\boldsymbol{\eta}},{\hat{\boldsymbol{\beta}}}^{*} \right){\boldsymbol{S}_{1}^{*}\left( t;\hat{\boldsymbol{\eta}},{\hat{\boldsymbol{\beta}}}^{*} \right)}^{'}}{{S_{0}^{*}\left( t;\hat{\boldsymbol{\eta}},{\hat{\boldsymbol{\beta}}}^{*} \right)}^{2}} \right\}{dN}_{k,l}\left( t \right) \right]^{-1}\left[ \int_{t} \left\{ \boldsymbol{X}_{i,j}-\frac{\boldsymbol{S}_{1}^{*}\left( t;\hat{\boldsymbol{\eta}},{\hat{\boldsymbol{\beta}}}^{*} \right)}{S_{0}^{*}\left( t;\hat{\boldsymbol{\eta}},{\hat{\boldsymbol{\beta}}}^{*} \right)} \right\}\times\left\{ \xi_{i,j} w_{i,j}\exp\left( {\hat{\boldsymbol{\eta}}}^{'}\boldsymbol{A}_{i,j} \right){dN}_{i,j}\left( t \right)-\xi_{i,j} w_{i,j}\exp\left( {\hat{\boldsymbol{\eta}}}^{'}\boldsymbol{A}_{i,j} \right)Y_{i,j}\left( t \right)\exp\left( {{\hat{\boldsymbol{\beta}}}^{*}}^{'}\boldsymbol{X}_{i,j} \right)\frac{\sum_{l=1}^{J} \sum_{k=1}^{n^{\left( l \right)}} {\xi_{k,l} w_{k,l}\exp\left( {\hat{\boldsymbol{\eta}}}^{'}\boldsymbol{A}_{k,l} \right) dN}_{k,l}\left( t \right)}{S_{0}^{*}\left( t;\hat{\boldsymbol{\eta}},{\hat{\boldsymbol{\beta}}}^{*} \right)} \right\} \right]$.

The estimating equation $\sum_{j=1}^{J} \sum_{i=1}^{n^{(j)}} \left\{ {dN}_{i,j}\left( t \right) \right\}-{d\hat{\Lambda}}_{0}^{*}\left( t \right)\times S_{0}^{*}\left( t;\hat{\boldsymbol{\eta}},{\hat{\boldsymbol{\beta}}}^{*} \right)=0$ leads to the Breslow estimate ${d\hat{\Lambda}}_{0}^{*}\left( t \right)=\frac{\sum_{j=1}^{J} \sum_{i=1}^{n^{(j)}} {dN}_{i,j}\left( t \right)}{S_{0}^{*}\left( t;\hat{\boldsymbol{\eta}},{\hat{\boldsymbol{\beta}}}^{*} \right)}$. Thus

${dN}_{i,j}\left( t \right)-{d\hat{\Lambda}}_{0}^{*}\left( t \right) \Delta_{i,j}\left\{ S_{0}^{*}\left( t;\hat{\boldsymbol{\eta}},{\hat{\boldsymbol{\beta}}}^{*} \right) \right\}-S_{0}^{*}\left( t;\hat{\boldsymbol{\eta}},{\hat{\boldsymbol{\beta}}}^{*} \right) \Delta_{i,j}\left\{ {d\hat{\Lambda}}_{0}^{*}\left( t \right) \right\}=0$,

or, in other words,

${dN}_{i,j}\left( t \right)-{d\hat{\Lambda}}_{0}^{*}\left( t \right)\xi_{i,j} w_{i,j}\exp\left( {\hat{\boldsymbol{\eta}}}^{'}\boldsymbol{A}_{i,j} \right)Y_{i,j}\left( t \right)\exp\left( {{\hat{\boldsymbol{\beta}}}^{*}}^{'}\boldsymbol{X}_{i,j} \right)-{d\hat{\Lambda}}_{0}^{*}\left( t \right) \left\{ {\frac{\partial S_{0}^{*}\left( t;\hat{\boldsymbol{\eta}},\boldsymbol{\beta} \right)}{\partial\boldsymbol{\beta}}}_{|\boldsymbol{\beta}={\hat{\boldsymbol{\beta}}}^{*}} \right\}^{'}\boldsymbol{\Delta}_{i,j}\left( {\hat{\boldsymbol{\beta}}}^{*} \right)$ $-{d\hat{\Lambda}}_{0}^{*}\left( t \right)\left\{ {\frac{\partial S_{0}^{*}\left( t;\boldsymbol{\eta},{\hat{\boldsymbol{\beta}}}^{*} \right)}{\partial\boldsymbol{\eta}}}_{|\boldsymbol{\eta}=\hat{\boldsymbol{\eta}}} \right\}^{'}\boldsymbol{\Delta}_{i,j}\left( \hat{\boldsymbol{\eta}} \right)-S_{0}^{*}\left( t;\hat{\boldsymbol{\eta}},{\hat{\boldsymbol{\beta}}}^{*} \right) \Delta_{i,j}\left\{ {d\hat{\Lambda}}_{0}^{*}\left( t \right) \right\}=0$.

As a result

$\Delta_{i,j}\left\{ {d\hat{\Lambda}}_{0}^{*}\left( t \right) \right\}=\left\{ S_{0}^{*}\left( t;\hat{\boldsymbol{\eta}},{\hat{\boldsymbol{\beta}}}^{*} \right) \right\}^{-1}\left[ {dN}_{i,j}\left( t \right)-{d\hat{\Lambda}}_{0}^{*}\left( t \right) \xi_{i,j}w_{i,j}\exp\left( {\hat{\boldsymbol{\eta}}}^{'}\boldsymbol{A}_{i,j} \right)Y_{i,j}\left( t \right)\exp\left( {{\hat{\boldsymbol{\beta}}}^{*}}^{'}\boldsymbol{X}_{i,j} \right)-{d\hat{\Lambda}}_{0}^{*}\left( t \right) \boldsymbol{S}_{1}^{*}\left( t;\hat{\boldsymbol{\eta}},{\hat{\boldsymbol{\beta}}}^{*} \right)'\boldsymbol{\Delta}_{i,j}\left( {\hat{\boldsymbol{\beta}}}^{*} \right)-{d\hat{\Lambda}}_{0}^{*}\left( t \right)\left\{ \sum_{l=1}^{J} \sum_{k=1}^{n^{(l)}} \xi_{k,l}w_{k,l}\exp({\hat{\boldsymbol{\eta}}}^{'}\boldsymbol{A}_{k,l})Y_{k,l}\left( t \right)\exp\left( {{\hat{\boldsymbol{\beta}}}^{*}}^{'}\boldsymbol{X}_{k,l} \right)\boldsymbol{A}_{k,l} \right\}^{'}\boldsymbol{\Delta}_{i,j}\left( \hat{\boldsymbol{\eta}} \right) \right]$,

that we can rewrite ${\Delta_{i,j}\left\{ {d\hat{\Lambda}}_{0}^{*}\left( t \right) \right\}=IF}_{i,j}^{(1)}\left( {d\hat{\Lambda}}_{0}^{*}\left( t \right) \right)+\xi_{i,j}w_{i,j} {IF}_{i,j}^{(2)}\left( {d\hat{\Lambda}}_{0}^{*}\left( t \right) \right)$,

with ${IF}_{i,j}^{\left( 1 \right)}\left\{ {d\hat{\Lambda}}_{0}^{*}\left( t \right) \right\}=\left\{ \sum_{l=1}^{J} \sum_{k=1}^{n^{\left( l \right)}} \xi_{k,l} w_{k,l}\exp\left( {\hat{\boldsymbol{\eta}}}^{'}\boldsymbol{A}_{k,l} \right)H_{k,l}(t)\boldsymbol{A}_{k,l}' \right\}\times\boldsymbol{IF}_{i,j}^{(1)}\left( \hat{\boldsymbol{\eta}} \right)$,

${IF}_{i,j}^{(2)}\left\{ {d\hat{\Lambda}}_{0}^{*}\left( t \right) \right\}=\left\{ S_{0}^{*}\left( t;\hat{\boldsymbol{\eta}},{\hat{\boldsymbol{\beta}}}^{*} \right) \right\}^{-1}{dN}_{i,j}\left( t \right)+\exp\left( {\hat{\boldsymbol{\eta}}}^{'}\boldsymbol{A}_{i,j} \right) H_{i,j}\left( t \right)+\left\{ \sum_{l=1}^{J} \sum_{k=1}^{n^{\left( l \right)}} \xi_{k,l} w_{k,l}\exp\left( {\hat{\boldsymbol{\eta}}}^{'}\boldsymbol{A}_{k,l} \right)H_{k,l}(t)\boldsymbol{A}_{k,l}' \right\}\times\boldsymbol{IF}_{i,j}^{(2)}\left( \hat{\boldsymbol{\eta}} \right)$,

where $\xi_{i,j}w_{i,j}=1$ if ${dN}_{i,j}\left( t \right)=1$, and $H_{i,j}\left( t \right)=-\left\{ S_{0}^{*}\left( t;\hat{\boldsymbol{\eta}},{\hat{\boldsymbol{\beta}}}^{*} \right) \right\}^{-1}{d\hat{\Lambda}}_{0}^{*}\left( t \right)\left\{ {\boldsymbol{S}_{1}^{*}\left( t;\hat{\boldsymbol{\eta}},{\hat{\boldsymbol{\beta}}}^{*} \right)}^{'}\boldsymbol{Z}_{i,j}+K_{i,j}(t) \right\}$ and $K_{i,j}\left( t \right)=Y_{i,j}\left( t \right)\exp\left( {\hat{\boldsymbol{\beta}}}^{*'}\boldsymbol{X}_{i,j} \right)$.

Note, $H_{i,j}\left( t \right)$, and thus ${IF}_{i,j}^{\left( 1 \right)}\left\{ {d\hat{\Lambda}}_{0}^{*}\left( t \right) \right\}$ and ${IF}_{i,j}^{\left( 2 \right)}\left\{ {d\hat{\Lambda}}_{0}^{*}\left( t \right) \right\}$, are linear combinations of the increments ${dN}_{i,j}\left( t \right)$ and ${d\hat{\Lambda}}_{0}^{*}\left( t \right)$. Thus $\Delta_{i,j}\left\{ \int_{\tau_{1}}^{\tau_{2}} d\hat{\Lambda}_{0}^{*}\left( t \right) \right\}=\int_{\tau_{1}}^{\tau_{2}} \Delta_{i,j}\left\{ d\hat{\Lambda}_{0}^{*}\left( t \right) \right\}$, that we can rewrite as ${IF}_{i,j}^{(1)}\left\{ \int_{\tau_{1}}^{\tau_{2}} d\hat{\Lambda}_{0}^{*}\left( t \right) \right\}+\xi_{i,j}w_{i,j} {IF}_{i,j}^{(2)}\left\{ \int_{\tau_{1}}^{\tau_{2}} d\hat{\Lambda}_{0}^{*}\left( t \right) \right\}$, with ${IF}_{i,j}^{(1)}\left\{ \hat{\Lambda}_{0}^{*}\left( \tau_{2} \right) \right\}=\int_{\tau_{1}}^{\tau_{2}} {IF}_{i,j}^{(1)}\left\{ d\hat{\Lambda}_{0}^{*}\left( t \right) \right\}$, and ${IF}_{i,j}^{(2)}\left\{ \hat{\Lambda}_{0}^{*}\left( \tau_{2} \right) \right\}=\int_{\tau_{1}}^{\tau_{2}} {IF}_{i,j}^{(2)}\left\{ d\hat{\Lambda}_{0}^{*}\left( t \right) \right\}$.

Finally

$\Delta_{i,j}\left\{ \hat{\pi}^{*}\left( \tau_{1},\tau_{2};\boldsymbol{x} \right) \right\}=\left\{ {\frac{\partial\hat{\pi}^{*}\left( \tau_{1},\tau_{2}; \boldsymbol{x} \right)}{\partial\boldsymbol{\beta}}}_{|\boldsymbol{\beta}={\hat{\boldsymbol{\beta}}}^{*}} \right\}\boldsymbol{\Delta}_{i,j}\left( {\hat{\boldsymbol{\beta}}}^{*} \right)+\left[ {\frac{\partial\hat{\pi}^{*}\left( \tau_{1},\tau_{2};\boldsymbol{x} \right)}{\partial\left\{ \int_{\tau_{1}}^{\tau_{2}} {d\Lambda}_{0}\left( t \right) \right\}}}_{|{d\Lambda}_{0}(t)={d\hat{\Lambda}}_{0}^{*}\left( t \right)} \right]\Delta_{i,j}\left\{ \int_{\tau_{1}}^{\tau_{2}} {d\hat{\Lambda}}_{0}^{*}\left( t \right) \right\}$,

with ${\frac{\partial\hat{\pi}^{*}\left( \tau_{1},\tau_{2}; \boldsymbol{x} \right)}{\partial\boldsymbol{\beta}}}_{|\boldsymbol{\beta}={\hat{\boldsymbol{\beta}}}^{*}}=\left\{ \int_{\tau_{1}}^{\tau_{2}} {d\hat{\Lambda}}_{0}^{*}\left( t \right)\exp\left( {\hat{\boldsymbol{\beta}}}^{*}'\boldsymbol{x} \right) \right\}\left\{ 1-\hat{\pi}^{*}\left( \tau_{1},\tau_{2};\boldsymbol{x} \right) \right\} \boldsymbol{x}'$,

and ${\frac{\partial\hat{\pi}^{*}\left( \tau_{1},\tau_{2};\boldsymbol{x} \right)}{\partial\int_{\tau_{1}}^{\tau_{2}} {d\Lambda}_{0}\left( t \right)}}_{|{d\Lambda}_{0}(t)={d\hat{\Lambda}}_{0}^{*}\left( t \right)}=\exp\left( \hat{\boldsymbol{\beta}}'\boldsymbol{x} \right)\left\{ 1-\hat{\pi}^{*}\left( \tau_{1},\tau_{2};\boldsymbol{x} \right) \right\}$,

and we can rewrite $\Delta_{i,j}\left\{ \hat{\pi}^{*}\left( \tau_{1},\tau_{2}; \boldsymbol{x} \right) \right\}$ as ${{IF}_{i,j}^{(1)}\left\{ \hat{\pi}^{*}\left( \tau_{1},\tau_{2};\boldsymbol{x} \right) \right\}+\xi}_{i,j}w_{i,j} {IF}_{i,j}^{(2)}\left\{ \hat{\pi}^{*}\left( \tau_{1},\tau_{2};\boldsymbol{x} \right) \right\}$.

Overall, for any ${\hat{\boldsymbol{\theta}}}^{*}\in\left\{ \hat{\boldsymbol{\eta}},{\hat{\boldsymbol{\beta}}}^{*}, d\hat{\Lambda}_{0}^{*}\left( t \right), \hat{\Lambda}_{0}^{*}\left( t \right), \hat{\pi}^{*}\left( \tau_{1},\tau_{2};\boldsymbol{x} \right) \right\}$, $\xi_{i,j}w_{i,j} \boldsymbol{IF}_{i,j}^{(2)}\left( {\hat{\boldsymbol{\theta}}}^{*} \right)$ is zero if individual $i$ in stratum $j$ is not in the stratified case-cohort; such individual affects ${\hat{\boldsymbol{\theta}}}^{*}$ through her/his influence on $\hat{\boldsymbol{\eta}}$, as he/she is used to calibrate the design weights.

## Comment on the choice of the auxiliary variables

As mentioned in Section 4.3 in the Main Document, for any ${\hat{\boldsymbol{\theta}}}^{*}\in\left\{ \hat{\boldsymbol{\eta}},{\hat{\boldsymbol{\beta}}}^{*}, d\hat{\Lambda}_{0}^{*}\left( t \right), \hat{\Lambda}_{0}^{*}\left( t \right), \hat{\pi}^{*}\left( \tau_{1},\tau_{2};\boldsymbol{x} \right) \right\}$, we estimate $\mathrm{var}\left( {\hat{\boldsymbol{\theta}}}^{*} \right)$ by

$\frac{n}{n-1}\sum_{j=1}^{J} \sum_{i=1}^{n^{\left( j \right)}} \left\{ \boldsymbol{IF}_{i,j}^{(1)}\left( {\hat{\boldsymbol{\theta}}}^{*} \right){\boldsymbol{IF}_{i,j}^{(1)}\left( {\hat{\boldsymbol{\theta}}}^{*} \right)}^{'}+2 {\xi_{i,j}w_{i,j}\boldsymbol{IF}}_{i,j}^{(2)}\left( {\hat{\boldsymbol{\theta}}}^{*} \right){\boldsymbol{IF}_{i,j}^{(2)}\left( {\hat{\boldsymbol{\theta}}}^{*} \right)}^{'}+{\xi_{i,j}w_{i,j}\boldsymbol{IF}}_{i,j}^{(2)}\left( {\hat{\boldsymbol{\theta}}}^{*} \right){\boldsymbol{IF}_{i,j}^{(2)}\left( {\hat{\boldsymbol{\theta}}}^{*} \right)}^{'} \right\}+\sum_{j=1}^{J} \sum_{i=1}^{n^{\left( j \right)}} \sum_{k=1}^{n^{\left( j \right)}} w_{i,k,j} \sigma_{i,k,j}w_{i,j}w_{k,j}{\xi_{i,j} \xi_{k,j}\boldsymbol{IF}}_{i,j}^{(2)}\left( {\hat{\boldsymbol{\theta}}}^{*} \right){\boldsymbol{IF}_{k,j}^{(2)}\left( {\hat{\boldsymbol{\theta}}}^{*} \right)}^{'}$,

with $\sigma_{i,k,j}=\frac{m^{(j)}}{n^{(j)}}\frac{m^{(j)}-1}{n^{(j)}-1}-\left( \frac{m^{\left( j \right)}}{n^{\left( j \right)}} \right)^{2}$ if individuals $i$ and $k$ in stratum $j$, $k\neq i$, are both non-cases, and $\sigma_{i,k,j}=0$ otherwise, and with $\sigma_{i,i,j}=\frac{m^{(j)}}{n^{\left( j \right)}}\left( 1-\frac{m^{(j)}}{n^{\left( j \right)}} \right)$ if individual $i$ in stratum $j$ is a non-case and $\sigma_{i,i,j}=0$ otherwise, $i,k\in\{1,\ldots,n^{(j)}\}$,$j\in\left\{ 1,\ldots,J \right\}$.

Observe that $\boldsymbol{IF}_{i,j}^{(1)}\left( {\hat{\boldsymbol{\beta}}}^{*} \right)$ given in Web Appendix C.2 is the predicted value for the $i$-th individual in the $j$-th stratum from the weighted linear regression model of the $\xi_{i,j}\boldsymbol{Z}_{i,j}$ on the $\xi_{i,j}\boldsymbol{A}_{i,j}$, using the calibrated weights ${w_{i,j}}^{*}$, $i\in\left\{ 1,\ldots,n^{\left( j \right)} \right\}$,$j\in\left\{ 1,\ldots,J \right\}$. Then, $\xi_{i,j}w_{i,j} \boldsymbol{IF}_{i,j}^{(2)}\left( {\hat{\boldsymbol{\beta}}}^{*} \right)$ is the $\sum_{l=1}^{j-1} n^{\left( l \right)}+i$-th weighted residual from this weighted linear regression, and it is zero if individual $i$ in stratum $j$ is not in the stratified case-cohort (i.e., not a case or not a sampled non-case), $i\in\left\{ 1,\ldots,n^{\left( j \right)} \right\}$,$j\in\left\{ 1,\ldots,J \right\}$. Similarly, ${IF}_{i,j}^{(1)}\left\{ d\hat{\Lambda}_{0}^{*}\left( t \right) \right\}$ is the predicted value for the $i$-th individual in the $j$-th stratum from the weighted linear regression of the $\xi_{i,j}H_{i,j}\left( t \right)$ on the $\xi_{i,j}\boldsymbol{A}_{i,j}$, using weights ${w_{i,j}}^{*}$, and ${IF}_{i,j}^{(2)}\left\{ d\hat{\Lambda}_{0}^{*}\left( t \right) \right\}$ is zero if individual $i$ in stratum $j$ is not in the stratified case-cohort, $i\in\left\{ 1,\ldots,n^{\left( j \right)} \right\}$,$j\in\left\{ 1,\ldots,J \right\}$.

Thus, for any individuals $i$ and $k$ in stratum $j$ such that $\sigma_{i,k,j}$ and $\sigma_{i,i,j}$ are non-zero (i.e., non-cases), ${\xi_{i,j}w_{i,j}\boldsymbol{IF}}_{i,j}^{(2)}\left( {\hat{\boldsymbol{\theta}}}^{*} \right)$ and $\xi_{k,j}w_{k,j} \boldsymbol{IF}_{k,j}^{(2)}\left( {\hat{\boldsymbol{\theta}}}^{*} \right)$ are weighted residuals from a weighted linear regression. Then, one can expect the phase-two component of the variance of ${\hat{\boldsymbol{\theta}}}^{*}$ to be smaller, and subsequently the overall variance to be smaller, when these weighted residuals are close to zero. This should be case the case for ${\hat{\boldsymbol{\theta}}}^{*}\in\left\{ {\hat{\boldsymbol{\beta}}}^{*}, d\hat{\Lambda}_{0}^{*}\left( t \right), \hat{\Lambda}_{0}^{*}\left( t \right), \hat{\pi}^{*}\left( \tau_{1},\tau_{2};\boldsymbol{x} \right) \right\}$ when $\boldsymbol{A}$ contains the influences for the log-relative hazard and the total follow-up times on the pure risk time interval multiplied by the relative hazards (as detailed in Section 4.1 in the Main Document and in Web Appendix C.1). Indeed, observe that $\boldsymbol{Z}_{i,j}$ given in Web Appendix C.2 is the “direct” influence of individual $i$ in stratum $j$ on ${\hat{\boldsymbol{\beta}}}^{*}$ (i.e., not through his/her influence on the calibrated weights), $i\in\{1,\ldots,n^{(j)}\}$,$j\in\left\{ 1,\ldots,J \right\}$. Its form is similar to that of the influence when using design weights; see Web Appendix B.1. On the other hand, $\int_{\tau_{1}}^{\tau_{2}} K_{i,j}\left( t \right)dt$ corresponds to the total follow-up time in the interval $(\tau_{1},\tau_{2}]$ multiplied by the estimated relative hazard of individual $i$ in stratum $j$, $i\in\left\{ 1,\ldots,n^{\left( j \right)} \right\}$,$j\in\left\{ 1,\ldots,J \right\}$. Although this point was not mentioned by Shin et al. (2020), it supports their choice of auxiliary variables over that of Breslow and Lumley (2013).

# SIMULATIONS

## Fixed parameter values used for the simulations in Section 7 of the Main Document

|  | $X_{1}<-2$ | $-2\leq X_{1}<1$ | $1\leq X_{1}$ |
| --- | --- | --- | --- |
| $p_{0\vert X_{1}}$ | 0.7 | 0.45 | 0.4 |
| $p_{1\vert X_{1}}$ | 0.05 | 0.2 | 0.3 |
| $p_{2\vert X_{1}}$ | 0.25 | 0.35 | 0.3 |

**WEB TABLE 1-** Parameter values for $p_{0|X_{1}}=P\left( X_{2}=0|X_{1} \right)$, $p_{1|X_{1}}=P\left( X_{2}=1|X_{1} \right)$ and $p_{2|X_{1}}=P\left( X_{2}=2|X_{1} \right)$, used for the simulation of $X_{2}$

| $\alpha_{1}$ | $\alpha_{2}$ | $\beta_{1}$ | $\beta_{2}$ | $\beta_{3}$ |
| --- | --- | --- | --- | --- |
| 0.05 | -0.35 | -0.2 | 0.25 | -0.3 |

**WEB TABLE 2-** Parameters values used for the simulation of $X_{3}$ and T

## Simulations results for all investigated scenarios

Recall that estimation was performed using the stratified case-cohort with design weights (SCC); the stratified case-cohort with calibrated weights (SCC.Calib); the unstratified case-cohort with design weights (USCC); and the unstratified case-cohort with calibrated weights (USCC.Calib). Then, for SCC, we used the variance estimate with superpopulation and phase-two variance components ($\hat{V}$) given in Equation (14) in Section 3.3 in the Main Document; and the robust variance estimate ($\hat{V}_{\mathrm{Robust}}$) given in Equation (15) in Section 3.3 in the Main Document. For SCC.Calib, we use the variance estimate with superpopulation and phase-two variance components ($\hat{V}$) given in Equation (18) in Section 4.3 in the Main Document; and the robust variance estimate ($\hat{V}_{\mathrm{Robust}}$) given in Equation (19) in Section 4.3 in the Main Document. For USCC and USCC.Calib, we used the variance estimates obtained from the simplified versions of these equations when $J = 1$. We also estimated these parameters using the data from the whole cohort, to serve as a point of reference (Cohort).

Simulations results for all 12 scenarios (with $n\in\left\{ 5\times{10}^{3},{10}^{4} \right\}$, $p_{Y}\in\{0.02, 0.05, 0.1\}$ and $N\in\left\{ 2, 4 \right\}$) are displayed in **WEB TABLE 3** to **WEB TABLE 20**. More precisely, Web Tables **WEB TABLE 3** to **WEB TABLE 8** display the coverages of 95% CIs, **WEB TABLE 9** to **WEB TABLE 20** display the empirical variances, the mean of estimated variances and the ratios of empirical variances with that from using the whole cohort, for $\beta_{1}$, $\beta_{2}$, $\beta_{3}$ and $\log\left\{ \pi\left( \tau_{1},\tau_{2}; \boldsymbol{x} \right) \right\}$, ${(\tau}_{1},\tau_{2}]=(0,8]$ and $\boldsymbol{x}\in\left\{ \left( -1, 1, -0.6 \right)',\left( 1, -1, 0.6 \right)',\left( 1, 1, 0.6 \right)' \right\}$, respectively. Interpretation of the results and conclusions are identical to that given in Section 7.2 in the Main Document.

## Comment on the robust variance estimation

Barlow (1994) also mentioned stratified sampling of the subcohort, and he stated that his robust formula could be used for variance estimation, but he did not specify the formula. Similarly, other authors who mentioned the robust variance with stratified case-cohort data did not provide the formula, even when comparing variance estimates (Gray, 2009; Samuelsen et al., 2007). On the other hand, Jiao (2002) stated that the “naïve” robust variance estimate (i.e., the sum of squared influences) of the relative hazard, as proposed by Barlow (1994), is not valid when the sampling of the subcohort is stratified. She proposed an extension of the robust variance for the stratified case-cohort design. But because no difference was observed between the two robust variance estimates in our simulations (results not shown), we used Barlow’s initial robust variance formula. Jiao (2002) conjectured that Barlow (1994) could simply be referring the sum of the squared influences, and we believe that many practitioners use the sum of squared influences, as in Barlow (1994).

| Cohort | SCC | | SCC.Calib | | USCC | | | USCC.Calib | | $n$ | $K$ | $p_{Y}$ | $\beta_{1}$ |
| --- | --- | --- | --- | --- | --- | --- | --- | --- | --- | --- | --- | --- | --- |
|  | $\hat{V}_{\mathrm{Robust}}$ | $\hat{V}$ | $\hat{V}_{\mathrm{Robust}}$ | $\hat{V}$ | $\hat{V}_{\mathrm{Robust}}$ | $\hat{V}$ | $\hat{V}_{\mathrm{Robust}}$ | | $\hat{V}$ |  |  |  |  |
| 0.944 | 0.9682* | 0.9516 | 0.9508 | 0.949 | 0.9558 | 0.956* | 0.953 | | 0.953 | 5000 | 2 | 0.02 | -0.2 |
| 0.9512 | 0.961* | 0.9524 | 0.9524 | 0.9512 | 0.9512 | 0.9514 | 0.9464 | | 0.9464 | 5000 | 4 | 0.02 | -0.2 |
| 0.9476 | 0.9668* | 0.9524 | 0.953 | 0.9506 | 0.947 | 0.947 | 0.9452 | | 0.9454 | 10000 | 2 | 0.02 | -0.2 |
| 0.948 | 0.9596* | 0.9514 | 0.9514 | 0.9498 | 0.9502 | 0.9502 | 0.9504 | | 0.9504 | 10000 | 4 | 0.02 | -0.2 |
| 0.9476 | 0.968* | 0.9548 | 0.9562* | 0.9544 | 0.9528 | 0.953 | 0.9482 | | 0.9484 | 5000 | 2 | 0.05 | -0.2 |
| 0.954 | 0.9622* | 0.9546 | 0.952 | 0.9514 | 0.9528 | 0.953 | 0.9524 | | 0.9524 | 5000 | 4 | 0.05 | -0.2 |
| 0.9552 | 0.9654* | 0.953 | 0.9562* | 0.9544 | 0.9536 | 0.9536 | 0.954 | | 0.954 | 10000 | 2 | 0.05 | -0.2 |
| 0.9482 | 0.9566* | 0.9516 | 0.9516 | 0.9504 | 0.9468 | 0.9468 | 0.9484 | | 0.9484 | 10000 | 4 | 0.05 | -0.2 |
| 0.947 | 0.9598* | 0.9448 | 0.9496 | 0.9478 | 0.9522 | 0.9522 | 0.9482 | | 0.9482 | 5000 | 2 | 0.1 | -0.2 |
| 0.9544 | 0.9584* | 0.9538 | 0.9572* | 0.956* | 0.955 | 0.955 | 0.9574* | | 0.9574* | 5000 | 4 | 0.1 | -0.2 |
| 0.9488 | 0.9618* | 0.9506 | 0.9514 | 0.9502 | 0.9474 | 0.9476 | 0.9506 | | 0.9506 | 10000 | 2 | 0.1 | -0.2 |
| 0.9526 | 0.9552 | 0.9496 | 0.9524 | 0.951 | 0.9478 | 0.9478 | 0.9492 | | 0.9492 | 10000 | 4 | 0.1 | -0.2 |

**WEB TABLE 3-** Coverage of 95% CIs for log-relative hazard parameter $\beta_{1}$ for different sampling designs and methods of analysis and variance estimation in 5,000 simulated cohorts. * indicates coverage outside the expected interval [0.9440; 0.9560]

| Cohort | SCC | | | SCC.Calib | | USCC | | USCC.Calib | | $n$ | $K$ | $p_{Y}$ | $\beta_{2}$ |
| --- | --- | --- | --- | --- | --- | --- | --- | --- | --- | --- | --- | --- | --- |
|  | $\hat{V}_{\mathrm{Robust}}$ | | $\hat{V}$ | $\hat{V}_{\mathrm{Robust}}$ | $\hat{V}$ | $\hat{V}_{\mathrm{Robust}}$ | $\hat{V}$ | $\hat{V}_{\mathrm{Robust}}$ | $\hat{V}$ |  |  |  |  |
| 0.9532 | 0.9714* | 0.9568* | | 0.9518 | 0.9514 | 0.9536 | 0.9536 | 0.9514 | 0.9512 | 5000 | 2 | 0.02 | 0.25 |
| 0.9514 | 0.9666* | | 0.955 | 0.9522 | 0.9522 | 0.9564* | 0.9564* | 0.9512 | 0.9512 | 5000 | 4 | 0.02 | 0.25 |
| 0.9486 | 0.9716* | | 0.9522 | 0.9498 | 0.9496 | 0.9554 | 0.9554 | 0.9466 | 0.9466 | 10000 | 2 | 0.02 | 0.25 |
| 0.9488 | 0.9606* | | 0.9504 | 0.9482 | 0.9482 | 0.9488 | 0.9492 | 0.9504 | 0.9504 | 10000 | 4 | 0.02 | 0.25 |
| 0.9488 | 0.967* | | 0.9488 | 0.9498 | 0.9498 | 0.953 | 0.953 | 0.9518 | 0.9516 | 5000 | 2 | 0.05 | 0.25 |
| 0.9524 | 0.9608* | | 0.9502 | 0.951 | 0.951 | 0.9542 | 0.9542 | 0.9518 | 0.9518 | 5000 | 4 | 0.05 | 0.25 |
| 0.9506 | 0.9676* | | 0.95 | 0.9484 | 0.9482 | 0.9506 | 0.9506 | 0.9536 | 0.9538 | 10000 | 2 | 0.05 | 0.25 |
| 0.951 | 0.9562* | | 0.9488 | 0.9484 | 0.9484 | 0.9504 | 0.9504 | 0.9522 | 0.9522 | 10000 | 4 | 0.05 | 0.25 |
| 0.9512 | 0.9656* | | 0.95 | 0.9498 | 0.9496 | 0.947 | 0.947 | 0.953 | 0.9532 | 5000 | 2 | 0.1 | 0.25 |
| 0.9496 | 0.9592* | | 0.952 | 0.9498 | 0.9498 | 0.9504 | 0.9506 | 0.9504 | 0.9504 | 5000 | 4 | 0.1 | 0.25 |
| 0.9504 | 0.9636* | | 0.9504 | 0.9502 | 0.9502 | 0.9524 | 0.9524 | 0.9514 | 0.9512 | 10000 | 2 | 0.1 | 0.25 |
| 0.952 | 0.9588* | | 0.953 | 0.9526 | 0.9526 | 0.9496 | 0.9496 | 0.9514 | 0.9516 | 10000 | 4 | 0.1 | 0.25 |

**WEB TABLE 4-** Coverage of 95% CIs for log-relative hazard parameter $\beta_{2}$ for different sampling designs and methods of analysis and variance estimation in 5,000 simulated cohorts. * indicates coverage outside the expected interval [0.9440; 0.9560]

| Cohort | SCC | | SCC.Calib | | USCC | | USCC.Calib | | $n$ | $K$ | $p_{Y}$ | $\beta_{3}$ |
| --- | --- | --- | --- | --- | --- | --- | --- | --- | --- | --- | --- | --- |
|  | $\hat{V}_{\mathrm{Robust}}$ | $\hat{V}$ | $\hat{V}_{\mathrm{Robust}}$ | $\hat{V}$ | $\hat{V}_{\mathrm{Robust}}$ | $\hat{V}$ | $\hat{V}_{\mathrm{Robust}}$ | $\hat{V}$ |  |  |  |  |
| 0.9442 | 0.9546 | 0.9548 | 0.9512 | 0.9504 | 0.9482 | 0.9486 | 0.9474 | 0.9474 | 5000 | 2 | 0.02 | -0.3 |
| 0.9464 | 0.9478 | 0.9478 | 0.949 | 0.9488 | 0.9566* | 0.9566* | 0.9526 | 0.9528 | 5000 | 4 | 0.02 | -0.3 |
| 0.9422* | 0.9492 | 0.9494 | 0.9498 | 0.9498 | 0.9454 | 0.9454 | 0.9482 | 0.9482 | 10000 | 2 | 0.02 | -0.3 |
| 0.9506 | 0.9516 | 0.9516 | 0.9518 | 0.9516 | 0.9524 | 0.9524 | 0.9496 | 0.9496 | 10000 | 4 | 0.02 | -0.3 |
| 0.9484 | 0.9462 | 0.9466 | 0.9454 | 0.9452 | 0.9522 | 0.9522 | 0.951 | 0.951 | 5000 | 2 | 0.05 | -0.3 |
| 0.9464 | 0.95 | 0.95 | 0.9474 | 0.9474 | 0.948 | 0.948 | 0.9458 | 0.9458 | 5000 | 4 | 0.05 | -0.3 |
| 0.9504 | 0.9466 | 0.9468 | 0.9484 | 0.9484 | 0.9456 | 0.9458 | 0.9508 | 0.9508 | 10000 | 2 | 0.05 | -0.3 |
| 0.9498 | 0.9522 | 0.9524 | 0.9536 | 0.9536 | 0.9512 | 0.9512 | 0.9516 | 0.9516 | 10000 | 4 | 0.05 | -0.3 |
| 0.9494 | 0.951 | 0.951 | 0.9498 | 0.9498 | 0.949 | 0.949 | 0.9532 | 0.9532 | 5000 | 2 | 0.1 | -0.3 |
| 0.9508 | 0.9498 | 0.9498 | 0.951 | 0.951 | 0.952 | 0.952 | 0.9502 | 0.9504 | 5000 | 4 | 0.1 | -0.3 |
| 0.9532 | 0.9518 | 0.9518 | 0.9528 | 0.9528 | 0.9516 | 0.9516 | 0.9534 | 0.9534 | 10000 | 2 | 0.1 | -0.3 |
| 0.9482 | 0.9536 | 0.9536 | 0.9524 | 0.9524 | 0.949 | 0.949 | 0.9514 | 0.9514 | 10000 | 4 | 0.1 | -0.3 |

**WEB TABLE 5-** Coverage of 95% CIs for log-relative hazard parameter $\beta_{3}$ for different sampling designs and methods of analysis and variance estimation in 5,000 simulated cohorts. * indicates coverage outside the expected interval [0.9440; 0.9560]

| Cohort | SCC | | SCC.Calib | | USCC | | USCC.Calib | | $n$ | $K$ | $p_{Y}$ | $\log\left\{ \pi\left( \tau_{1},\tau_{2};\boldsymbol{x} \right) \right\}$ |
| --- | --- | --- | --- | --- | --- | --- | --- | --- | --- | --- | --- | --- |
|  | $\hat{V}_{\mathrm{Robust}}$ | $\hat{V}$ | $\hat{V}_{\mathrm{Robust}}$ | $\hat{V}$ | $\hat{V}_{\mathrm{Robust}}$ | $\hat{V}$ | $\hat{V}_{\mathrm{Robust}}$ | $\hat{V}$ |  |  |  |  |
| 0.9466 | 0.9722* | 0.95 | 0.9562* | 0.9524 | 0.9634* | 0.9494 | 0.952 | 0.952 | 5000 | 2 | 0.02 | -3.948 |
| 0.9484 | 0.9602* | 0.9492 | 0.9522 | 0.951 | 0.9558 | 0.9456 | 0.9494 | 0.9496 | 5000 | 4 | 0.02 | -3.948 |
| 0.956* | 0.973* | 0.9568* | 0.9604* | 0.9554 | 0.9656* | 0.9526 | 0.956 | 0.956 | 10000 | 2 | 0.02 | -3.948 |
| 0.951 | 0.9656* | 0.9538 | 0.9538 | 0.9522 | 0.9602* | 0.9526 | 0.951 | 0.951 | 10000 | 4 | 0.02 | -3.948 |
| 0.9488 | 0.9694* | 0.9494 | 0.9536 | 0.949 | 0.958* | 0.948 | 0.9502 | 0.9502 | 5000 | 2 | 0.05 | -3.046 |
| 0.9546 | 0.9634* | 0.955 | 0.956* | 0.9546 | 0.9596* | 0.9538 | 0.9534 | 0.9534 | 5000 | 4 | 0.05 | -3.046 |
| 0.9556 | 0.971* | 0.9556 | 0.9602* | 0.9554 | 0.9656* | 0.9554 | 0.954 | 0.954 | 10000 | 2 | 0.05 | -3.046 |
| 0.9534 | 0.9604* | 0.9518 | 0.9542 | 0.9528 | 0.962* | 0.9538 | 0.9532 | 0.9532 | 10000 | 4 | 0.05 | -3.046 |
| 0.9506 | 0.9658* | 0.951 | 0.9538 | 0.9512 | 0.9622* | 0.954 | 0.9528 | 0.9528 | 5000 | 2 | 0.1 | -2.377 |
| 0.9496 | 0.9566* | 0.9512 | 0.95 | 0.949 | 0.9544 | 0.9514 | 0.951 | 0.951 | 5000 | 4 | 0.1 | -2.377 |
| 0.9484 | 0.9634* | 0.9476 | 0.9532 | 0.9498 | 0.957* | 0.9484 | 0.9446 | 0.9446 | 10000 | 2 | 0.1 | -2.377 |
| 0.9538 | 0.9586* | 0.9524 | 0.9548 | 0.9544 | 0.9594* | 0.9562* | 0.9558 | 0.9558 | 10000 | 4 | 0.1 | -2.377 |

**WEB TABLE 6-** Coverage of 95% CIs for log-pure risk $\log\left\{ \pi\left( \tau_{1},\tau_{2}; \boldsymbol{x} \right) \right\}$, with ${(\tau}_{1},\tau_{2}]=(0,8]$ and $\boldsymbol{x}=\left( -1, 1, -0.6 \right)'$, for different sampling designs and methods of analysis and variance estimation in 5,000 simulated cohorts. * indicates coverage outside the expected interval [0.9440; 0.9560]

| Cohort | SCC | | SCC.Calib | | USCC | | USCC.Calib | | $n$ | $K$ | $p_{Y}$ | $\log\left\{ \pi\left( \tau_{1},\tau_{2};\boldsymbol{x} \right) \right\}$ |
| --- | --- | --- | --- | --- | --- | --- | --- | --- | --- | --- | --- | --- |
|  | $\hat{V}_{\mathrm{Robust}}$ | $\hat{V}$ | $\hat{V}_{\mathrm{Robust}}$ | $\hat{V}$ | $\hat{V}_{\mathrm{Robust}}$ | $\hat{V}$ | $\hat{V}_{\mathrm{Robust}}$ | $\hat{V}$ |  |  |  |  |
| 0.9476 | 0.9754* | 0.9504 | 0.9526 | 0.9526 | 0.9552 | 0.9526 | 0.9518 | 0.9518 | 5000 | 2 | 0.02 | -5.201 |
| 0.946 | 0.9642* | 0.948 | 0.9502 | 0.9498 | 0.953 | 0.9514 | 0.9444 | 0.9444 | 5000 | 4 | 0.02 | -5.201 |
| 0.952 | 0.9722* | 0.9518 | 0.9542 | 0.9532 | 0.954 | 0.9522 | 0.95 | 0.95 | 10000 | 2 | 0.02 | -5.201 |
| 0.946 | 0.9582* | 0.9482 | 0.947 | 0.947 | 0.9528 | 0.9508 | 0.9462 | 0.9462 | 10000 | 4 | 0.02 | -5.201 |
| 0.9456 | 0.968* | 0.9462 | 0.9478 | 0.9478 | 0.9512 | 0.9486 | 0.948 | 0.948 | 5000 | 2 | 0.05 | -4.288 |
| 0.9488 | 0.962* | 0.9508 | 0.9492 | 0.9492 | 0.9516 | 0.95 | 0.946 | 0.946 | 5000 | 4 | 0.05 | -4.288 |
| 0.9508 | 0.966* | 0.949 | 0.9514 | 0.9506 | 0.9558 | 0.9522 | 0.9494 | 0.9494 | 10000 | 2 | 0.05 | -4.288 |
| 0.9526 | 0.9576* | 0.9508 | 0.9536 | 0.9532 | 0.9526 | 0.9512 | 0.9536 | 0.9536 | 10000 | 4 | 0.05 | -4.288 |
| 0.9442 | 0.9666* | 0.9492 | 0.9488 | 0.9484 | 0.9518 | 0.9494 | 0.9486 | 0.9486 | 5000 | 2 | 0.1 | -3.602 |
| 0.9548 | 0.9616* | 0.9546 | 0.9524 | 0.9524 | 0.954 | 0.9534 | 0.9542 | 0.9542 | 5000 | 4 | 0.1 | -3.602 |
| 0.9526 | 0.9698* | 0.9568* | 0.9526 | 0.9522 | 0.9544 | 0.9522 | 0.9488 | 0.9488 | 10000 | 2 | 0.1 | -3.602 |
| 0.9514 | 0.959* | 0.9518 | 0.9532 | 0.9532 | 0.9524 | 0.9514 | 0.9534 | 0.9534 | 10000 | 4 | 0.1 | -3.602 |

**WEB TABLE 7-** Coverage of 95% CIs for log-pure risk $\log\left\{ \pi\left( \tau_{1},\tau_{2}; \boldsymbol{x} \right) \right\}$, with ${(\tau}_{1},\tau_{2}]=(0,8]$ and $\boldsymbol{x}=\left( 1, -1, 0.6 \right)'$, for different sampling designs and methods of analysis and variance estimation in 5,000 simulated cohorts. * indicates coverage outside the expected interval [0.9440; 0.9560]

| Cohort | SCC | | SCC.Calib | | USCC | | USCC.Calib | | $n$ | $K$ | $p_{Y}$ | $\log\left\{ \pi\left( \tau_{1},\tau_{2};\boldsymbol{x} \right) \right\}$ |
| --- | --- | --- | --- | --- | --- | --- | --- | --- | --- | --- | --- | --- |
|  | $\hat{V}_{\mathrm{Robust}}$ | $\hat{V}$ | $\hat{V}_{\mathrm{Robust}}$ | $\hat{V}$ | $\hat{V}_{\mathrm{Robust}}$ | $\hat{V}$ | $\hat{V}_{\mathrm{Robust}}$ | $\hat{V}$ |  |  |  |  |
| 0.955 | 0.9712* | 0.9582* | 0.9572* | 0.956* | 0.9658* | 0.9596* | 0.9592* | 0.9592* | 5000 | 2 | 0.02 | -4.702 |
| 0.9468 | 0.9578* | 0.9494 | 0.9496 | 0.949 | 0.9534 | 0.95 | 0.9494 | 0.9494 | 5000 | 4 | 0.02 | -4.702 |
| 0.952 | 0.9666* | 0.9542 | 0.9538 | 0.952 | 0.9574* | 0.95 | 0.9502 | 0.9502 | 10000 | 2 | 0.02 | -4.702 |
| 0.9492 | 0.9584* | 0.9504 | 0.9526 | 0.9518 | 0.956* | 0.9522 | 0.948 | 0.948 | 10000 | 4 | 0.02 | -4.702 |
| 0.9488 | 0.962* | 0.9488 | 0.9488 | 0.9478 | 0.9572* | 0.949 | 0.9504 | 0.9504 | 5000 | 2 | 0.05 | -3.793 |
| 0.95 | 0.9602* | 0.953 | 0.9508 | 0.9498 | 0.9526 | 0.9502 | 0.9514 | 0.9516 | 5000 | 4 | 0.05 | -3.793 |
| 0.9462 | 0.9606* | 0.9468 | 0.9504 | 0.9494 | 0.953 | 0.9444 | 0.9446 | 0.9446 | 10000 | 2 | 0.05 | -3.793 |
| 0.9496 | 0.9562* | 0.9494 | 0.9536 | 0.9532 | 0.9518 | 0.95 | 0.9526 | 0.9526 | 10000 | 4 | 0.05 | -3.793 |
| 0.9452 | 0.9608* | 0.9486 | 0.9482 | 0.9478 | 0.9514 | 0.9466 | 0.9446 | 0.9446 | 5000 | 2 | 0.1 | -3.111 |
| 0.9516 | 0.957* | 0.9522 | 0.9524 | 0.9518 | 0.9544 | 0.9506 | 0.9514 | 0.9514 | 5000 | 4 | 0.1 | -3.111 |
| 0.9502 | 0.9606* | 0.9512 | 0.9494 | 0.9492 | 0.9522 | 0.9466 | 0.9478 | 0.9478 | 10000 | 2 | 0.1 | -3.111 |
| 0.9528 | 0.956* | 0.9512 | 0.9516 | 0.9514 | 0.9526 | 0.9508 | 0.953 | 0.953 | 10000 | 4 | 0.1 | -3.111 |

**WEB TABLE 8-** Coverage of 95% CIs for log-pure risk $\log\left\{ \pi\left( \tau_{1},\tau_{2}; \boldsymbol{x} \right) \right\}$, with ${(\tau}_{1},\tau_{2}]=(0,8]$ and $\boldsymbol{x}=\left( 1, 1, 0.6 \right)'$, for different sampling designs and methods of analysis and variance estimation in 5,000 simulated cohorts. * indicates coverage outside the expected interval [0.9440; 0.9560]

| Cohort | | | SCC | | | SCC.Calib | | | Ratio of empirical variance with the whole cohort to empirical variance with | | $n$ | $K$ | $p_{Y}$ | $\beta_{1}$ |
| --- | --- | --- | --- | --- | --- | --- | --- | --- | --- | --- | --- | --- | --- | --- |
| Empir var | $\hat{V}_{\mathrm{Robust}}$ | Empir var | | $\hat{V}_{\mathrm{Robust}}$ | $\hat{V}$ | Empir var | $\hat{V}_{\mathrm{Robust}}$ | $\hat{V}$ | SCC | SCC.Calib |  |  |  |  |
| 0.0138 | 0.0138 | 0.0181 | | 0.0212 | 0.0182 | 0.0168 | 0.0175 | 0.0171 | 0.763 | 0.8228 | 5000 | 2 | 0.02 | -0.2 |
| 0.0138 | 0.0139 | 0.0159 | | 0.0174 | 0.016 | 0.0151 | 0.0156 | 0.0155 | 0.8678 | 0.9094 | 5000 | 4 | 0.02 | -0.2 |
| 0.007 | 0.0069 | 0.0085 | | 0.0102 | 0.0087 | 0.0081 | 0.0084 | 0.0082 | 0.8161 | 0.8557 | 10000 | 2 | 0.02 | -0.2 |
| 0.0069 | 0.0069 | 0.0076 | | 0.0085 | 0.0078 | 0.0074 | 0.0076 | 0.0075 | 0.9055 | 0.9309 | 10000 | 4 | 0.02 | -0.2 |
| 0.0056 | 0.0056 | 0.0068 | | 0.0079 | 0.0069 | 0.0064 | 0.0066 | 0.0065 | 0.8327 | 0.8852 | 5000 | 2 | 0.05 | -0.2 |
| 0.0055 | 0.0056 | 0.006 | | 0.0066 | 0.0061 | 0.0059 | 0.006 | 0.006 | 0.917 | 0.9405 | 5000 | 4 | 0.05 | -0.2 |
| 0.0027 | 0.0028 | 0.0033 | | 0.0039 | 0.0034 | 0.0031 | 0.0033 | 0.0032 | 0.8367 | 0.8844 | 10000 | 2 | 0.05 | -0.2 |
| 0.0028 | 0.0028 | 0.003 | | 0.0033 | 0.0031 | 0.0029 | 0.003 | 0.003 | 0.9275 | 0.9551 | 10000 | 4 | 0.05 | -0.2 |
| 0.003 | 0.0029 | 0.0034 | | 0.0037 | 0.0033 | 0.0032 | 0.0032 | 0.0032 | 0.8631 | 0.909 | 5000 | 2 | 0.1 | -0.2 |
| 0.0028 | 0.0029 | 0.003 | | 0.0032 | 0.003 | 0.0029 | 0.003 | 0.003 | 0.9535 | 0.9764 | 5000 | 4 | 0.1 | -0.2 |
| 0.0014 | 0.0014 | 0.0016 | | 0.0019 | 0.0017 | 0.0016 | 0.0016 | 0.0016 | 0.8596 | 0.9055 | 10000 | 2 | 0.1 | -0.2 |
| 0.0014 | 0.0014 | 0.0015 | | 0.0016 | 0.0015 | 0.0015 | 0.0015 | 0.0015 | 0.9603 | 0.9744 | 10000 | 4 | 0.1 | -0.2 |

**WEB TABLE 9-** Empirical variance, mean of estimated variances and ratio of empirical variance (to that with the whole cohort) of log-relative hazard parameter $\beta_{1}$ for different sampling designs and methods of analysis and variance estimation in 5,000 simulated cohorts

| Cohort | | USCC | | | USCC.Calib | | | Ratio of empirical variance with the whole cohort to empirical variance with | | $n$ | $K$ | $p_{Y}$ | $\beta_{1}$ |
| --- | --- | --- | --- | --- | --- | --- | --- | --- | --- | --- | --- | --- | --- |
| Empir var | $\hat{V}_{\mathrm{Robust}}$ | Empir var | $\hat{V}_{\mathrm{Robust}}$ | $\hat{V}$ | Empir var | $\hat{V}_{\mathrm{Robust}}$ | $\hat{V}$ | USCC | USCC.Calib |  |  |  |  |
| 0.0138 | 0.0138 | 0.0223 | 0.0222 | 0.0223 | 0.0175 | 0.0182 | 0.0182 | 0.62 | 0.7889 | 5000 | 2 | 0.02 | -0.2 |
| 0.0138 | 0.0139 | 0.0177 | 0.0177 | 0.0178 | 0.0158 | 0.0158 | 0.0158 | 0.7769 | 0.873 | 5000 | 4 | 0.02 | -0.2 |
| 0.007 | 0.0069 | 0.0108 | 0.0106 | 0.0106 | 0.0086 | 0.0086 | 0.0086 | 0.6408 | 0.8084 | 10000 | 2 | 0.02 | -0.2 |
| 0.0069 | 0.0069 | 0.0086 | 0.0086 | 0.0087 | 0.0076 | 0.0077 | 0.0077 | 0.8017 | 0.9053 | 10000 | 4 | 0.02 | -0.2 |
| 0.0056 | 0.0056 | 0.0082 | 0.0082 | 0.0082 | 0.0067 | 0.0068 | 0.0068 | 0.6897 | 0.8485 | 5000 | 2 | 0.05 | -0.2 |
| 0.0055 | 0.0056 | 0.0066 | 0.0067 | 0.0067 | 0.006 | 0.0061 | 0.0061 | 0.8331 | 0.9212 | 5000 | 4 | 0.05 | -0.2 |
| 0.0027 | 0.0028 | 0.004 | 0.004 | 0.004 | 0.0032 | 0.0033 | 0.0033 | 0.6895 | 0.8431 | 10000 | 2 | 0.05 | -0.2 |
| 0.0028 | 0.0028 | 0.0033 | 0.0033 | 0.0033 | 0.003 | 0.003 | 0.003 | 0.8424 | 0.9415 | 10000 | 4 | 0.05 | -0.2 |
| 0.003 | 0.0029 | 0.0039 | 0.0039 | 0.0039 | 0.0033 | 0.0033 | 0.0033 | 0.7548 | 0.8896 | 5000 | 2 | 0.1 | -0.2 |
| 0.0028 | 0.0029 | 0.0032 | 0.0032 | 0.0032 | 0.003 | 0.003 | 0.003 | 0.8931 | 0.9581 | 5000 | 4 | 0.1 | -0.2 |
| 0.0014 | 0.0014 | 0.0019 | 0.0019 | 0.0019 | 0.0016 | 0.0016 | 0.0016 | 0.7398 | 0.8728 | 10000 | 2 | 0.1 | -0.2 |
| 0.0014 | 0.0014 | 0.0016 | 0.0016 | 0.0016 | 0.0015 | 0.0015 | 0.0015 | 0.8908 | 0.9462 | 10000 | 4 | 0.1 | -0.2 |

**WEB TABLE 10-** Empirical variance, mean of estimated variances and ratio of empirical variance (to that with the whole cohort) of log-relative hazard parameter $\beta_{1}$ for different sampling designs and methods of analysis and variance estimation in 5,000 simulated cohorts

| Cohort | | SCC | | | | SCC.Calib | | | Ratio of empirical variance with the whole cohort to empirical variance with | | $n$ | $K$ | $p_{Y}$ | $\beta_{2}$ |
| --- | --- | --- | --- | --- | --- | --- | --- | --- | --- | --- | --- | --- | --- | --- |
| Empir var | $\hat{V}_{\mathrm{Robust}}$ | Empir var | $\hat{V}_{\mathrm{Robust}}$ | $\hat{V}$ | Empir var | | $\hat{V}_{\mathrm{Robust}}$ | $\hat{V}$ | SCC | SCC.Calib |  |  |  |  |
| 0.0197 | 0.0197 | 0.0235 | 0.0286 | 0.0237 | 0.0209 | | 0.0212 | 0.0212 | 0.8415 | 0.9425 | 5000 | 2 | 0.02 | 0.25 |
| 0.0195 | 0.0198 | 0.0213 | 0.024 | 0.0217 | 0.02 | | 0.0205 | 0.0205 | 0.9165 | 0.9745 | 5000 | 4 | 0.02 | 0.25 |
| 0.01 | 0.0097 | 0.0115 | 0.0139 | 0.0114 | 0.0105 | | 0.0103 | 0.0102 | 0.871 | 0.955 | 10000 | 2 | 0.02 | 0.25 |
| 0.0099 | 0.0097 | 0.0108 | 0.0117 | 0.0106 | 0.0102 | | 0.01 | 0.01 | 0.9173 | 0.9781 | 10000 | 4 | 0.02 | 0.25 |
| 0.008 | 0.0079 | 0.0092 | 0.0108 | 0.0091 | 0.0084 | | 0.0082 | 0.0082 | 0.868 | 0.9572 | 5000 | 2 | 0.05 | 0.25 |
| 0.008 | 0.0079 | 0.0085 | 0.0092 | 0.0084 | 0.0081 | | 0.008 | 0.008 | 0.9404 | 0.9872 | 5000 | 4 | 0.05 | 0.25 |
| 0.0039 | 0.0039 | 0.0045 | 0.0054 | 0.0045 | 0.004 | | 0.0041 | 0.0041 | 0.863 | 0.9685 | 10000 | 2 | 0.05 | 0.25 |
| 0.0038 | 0.0039 | 0.0042 | 0.0046 | 0.0042 | 0.0039 | | 0.004 | 0.004 | 0.9263 | 0.9815 | 10000 | 4 | 0.05 | 0.25 |
| 0.004 | 0.004 | 0.0045 | 0.0052 | 0.0045 | 0.0041 | | 0.0041 | 0.0041 | 0.8895 | 0.9734 | 5000 | 2 | 0.1 | 0.25 |
| 0.0041 | 0.004 | 0.0042 | 0.0044 | 0.0042 | 0.0041 | | 0.0041 | 0.0041 | 0.9647 | 0.9883 | 5000 | 4 | 0.1 | 0.25 |
| 0.002 | 0.002 | 0.0022 | 0.0026 | 0.0022 | 0.002 | | 0.0021 | 0.0021 | 0.8994 | 0.9737 | 10000 | 2 | 0.1 | 0.25 |
| 0.002 | 0.002 | 0.0021 | 0.0022 | 0.0021 | 0.002 | | 0.002 | 0.002 | 0.9564 | 0.9877 | 10000 | 4 | 0.1 | 0.25 |

**WEB TABLE 11-** Empirical variance, mean of estimated variances and ratio of empirical variance (to that with the whole cohort) of log-relative hazard parameter $\beta_{2}$ for different sampling designs and methods of analysis and variance estimation in 5,000 simulated cohorts

| Cohort | | | USCC | | | USCC.Calib | | | Ratio of empirical variance with the whole cohort to empirical variance with | | $n$ | $K$ | $p_{Y}$ | $\beta_{2}$ |
| --- | --- | --- | --- | --- | --- | --- | --- | --- | --- | --- | --- | --- | --- | --- |
| Empir var | $\hat{V}_{\mathrm{Robust}}$ | Empir var | | $\hat{V}_{\mathrm{Robust}}$ | $\hat{V}$ | Empir var | $\hat{V}_{\mathrm{Robust}}$ | $\hat{V}$ | USCC | USCC.Calib |  |  |  |  |
| 0.0197 | 0.0197 | 0.0287 | | 0.029 | 0.0291 | 0.0209 | 0.0213 | 0.0213 | 0.6885 | 0.9436 | 5000 | 2 | 0.02 | 0.25 |
| 0.0195 | 0.0198 | 0.0239 | | 0.0243 | 0.0243 | 0.0203 | 0.0206 | 0.0206 | 0.816 | 0.9624 | 5000 | 4 | 0.02 | 0.25 |
| 0.01 | 0.0097 | 0.014 | | 0.014 | 0.014 | 0.0105 | 0.0103 | 0.0103 | 0.7143 | 0.9539 | 10000 | 2 | 0.02 | 0.25 |
| 0.0099 | 0.0097 | 0.012 | | 0.0118 | 0.0118 | 0.0102 | 0.01 | 0.01 | 0.8279 | 0.9737 | 10000 | 4 | 0.02 | 0.25 |
| 0.008 | 0.0079 | 0.0108 | | 0.0109 | 0.0109 | 0.0084 | 0.0082 | 0.0082 | 0.7415 | 0.957 | 5000 | 2 | 0.05 | 0.25 |
| 0.008 | 0.0079 | 0.0093 | | 0.0092 | 0.0092 | 0.0081 | 0.008 | 0.008 | 0.8621 | 0.9802 | 5000 | 4 | 0.05 | 0.25 |
| 0.0039 | 0.0039 | 0.0054 | | 0.0054 | 0.0054 | 0.004 | 0.0041 | 0.0041 | 0.725 | 0.9648 | 10000 | 2 | 0.05 | 0.25 |
| 0.0038 | 0.0039 | 0.0044 | | 0.0046 | 0.0046 | 0.0039 | 0.004 | 0.004 | 0.8763 | 0.9881 | 10000 | 4 | 0.05 | 0.25 |
| 0.004 | 0.004 | 0.0052 | | 0.0052 | 0.0052 | 0.0041 | 0.0041 | 0.0041 | 0.7727 | 0.9804 | 5000 | 2 | 0.1 | 0.25 |
| 0.0041 | 0.004 | 0.0045 | | 0.0044 | 0.0044 | 0.0041 | 0.0041 | 0.0041 | 0.9184 | 0.9941 | 5000 | 4 | 0.1 | 0.25 |
| 0.002 | 0.002 | 0.0025 | | 0.0026 | 0.0026 | 0.002 | 0.002 | 0.002 | 0.7814 | 0.9843 | 10000 | 2 | 0.1 | 0.25 |
| 0.002 | 0.002 | 0.0022 | | 0.0022 | 0.0022 | 0.002 | 0.002 | 0.002 | 0.8997 | 0.9936 | 10000 | 4 | 0.1 | 0.25 |

**WEB TABLE 12-** Empirical variance, mean of estimated variances and ratio of empirical variance (to that with the whole cohort) of log-relative hazard parameter $\beta_{2}$ for different sampling designs and methods of analysis and variance estimation in 5,000 simulated cohorts

| Cohort | | SCC | | | | SCC.Calib | | | Ratio of empirical variance with the whole cohort to empirical variance with | | $n$ | $K$ | $p_{Y}$ | $\beta_{3}$ |
| --- | --- | --- | --- | --- | --- | --- | --- | --- | --- | --- | --- | --- | --- | --- |
| Empir var | $\hat{V}_{\mathrm{Robust}}$ | Empir var | $\hat{V}_{\mathrm{Robust}}$ | $\hat{V}$ | Empir var | | $\hat{V}_{\mathrm{Robust}}$ | $\hat{V}$ | SCC | SCC.Calib |  |  |  |  |
| 0.014 | 0.0136 | 0.0217 | 0.0214 | 0.0215 | 0.0177 | | 0.0178 | 0.0178 | 0.6443 | 0.7884 | 5000 | 2 | 0.02 | -0.3 |
| 0.0135 | 0.0136 | 0.0176 | 0.0173 | 0.0173 | 0.0154 | | 0.0156 | 0.0156 | 0.7652 | 0.8785 | 5000 | 4 | 0.02 | -0.3 |
| 0.0069 | 0.0068 | 0.0103 | 0.0102 | 0.0102 | 0.0083 | | 0.0084 | 0.0084 | 0.6734 | 0.8281 | 10000 | 2 | 0.02 | -0.3 |
| 0.0067 | 0.0068 | 0.0085 | 0.0084 | 0.0084 | 0.0075 | | 0.0075 | 0.0075 | 0.7921 | 0.9001 | 10000 | 4 | 0.02 | -0.3 |
| 0.0056 | 0.0055 | 0.0081 | 0.0079 | 0.0079 | 0.0067 | | 0.0066 | 0.0066 | 0.6875 | 0.8305 | 5000 | 2 | 0.05 | -0.3 |
| 0.0057 | 0.0055 | 0.0066 | 0.0066 | 0.0066 | 0.0061 | | 0.006 | 0.006 | 0.8593 | 0.9324 | 5000 | 4 | 0.05 | -0.3 |
| 0.0028 | 0.0028 | 0.004 | 0.0039 | 0.0039 | 0.0033 | | 0.0033 | 0.0033 | 0.696 | 0.8458 | 10000 | 2 | 0.05 | -0.3 |
| 0.0027 | 0.0028 | 0.0032 | 0.0032 | 0.0032 | 0.0029 | | 0.003 | 0.003 | 0.8468 | 0.9312 | 10000 | 4 | 0.05 | -0.3 |
| 0.0029 | 0.0028 | 0.0037 | 0.0037 | 0.0037 | 0.0032 | | 0.0032 | 0.0032 | 0.7675 | 0.892 | 5000 | 2 | 0.1 | -0.3 |
| 0.0028 | 0.0028 | 0.0031 | 0.0031 | 0.0031 | 0.0029 | | 0.003 | 0.003 | 0.9157 | 0.962 | 5000 | 4 | 0.1 | -0.3 |
| 0.0014 | 0.0014 | 0.0018 | 0.0018 | 0.0018 | 0.0016 | | 0.0016 | 0.0016 | 0.7728 | 0.8856 | 10000 | 2 | 0.1 | -0.3 |
| 0.0014 | 0.0014 | 0.0016 | 0.0016 | 0.0016 | 0.0015 | | 0.0015 | 0.0015 | 0.9 | 0.9557 | 10000 | 4 | 0.1 | -0.3 |

**WEB TABLE 13-** Empirical variance, mean of estimated variances and ratio of empirical variance (to that with the whole cohort) of log-relative hazard parameter $\beta_{3}$ for different sampling designs and methods of analysis and variance estimation in 5,000 simulated cohorts

| Cohort | | | USCC | | | USCC.Calib | | | Ratio of empirical variance with the whole cohort to empirical variance with | | $n$ | $K$ | $p_{Y}$ | $\beta_{3}$ |
| --- | --- | --- | --- | --- | --- | --- | --- | --- | --- | --- | --- | --- | --- | --- |
| Empir var | $\hat{V}_{\mathrm{Robust}}$ | Empir var | | $\hat{V}_{\mathrm{Robust}}$ | $\hat{V}$ | Empir var | $\hat{V}_{\mathrm{Robust}}$ | $\hat{V}$ | USCC | USCC.Calib |  |  |  |  |
| 0.014 | 0.0136 | 0.0223 | | 0.0223 | 0.0223 | 0.0181 | 0.0182 | 0.0182 | 0.6254 | 0.7728 | 5000 | 2 | 0.02 | -0.3 |
| 0.0135 | 0.0136 | 0.0175 | | 0.0178 | 0.0178 | 0.0153 | 0.0158 | 0.0158 | 0.7707 | 0.8822 | 5000 | 4 | 0.02 | -0.3 |
| 0.0069 | 0.0068 | 0.0109 | | 0.0107 | 0.0107 | 0.0087 | 0.0087 | 0.0087 | 0.6335 | 0.7962 | 10000 | 2 | 0.02 | -0.3 |
| 0.0067 | 0.0068 | 0.0085 | | 0.0086 | 0.0086 | 0.0076 | 0.0076 | 0.0076 | 0.7916 | 0.8898 | 10000 | 4 | 0.02 | -0.3 |
| 0.0056 | 0.0055 | 0.0082 | | 0.0082 | 0.0082 | 0.0068 | 0.0067 | 0.0067 | 0.6781 | 0.8221 | 5000 | 2 | 0.05 | -0.3 |
| 0.0057 | 0.0055 | 0.0069 | | 0.0067 | 0.0067 | 0.0062 | 0.0061 | 0.0061 | 0.8276 | 0.9175 | 5000 | 4 | 0.05 | -0.3 |
| 0.0028 | 0.0028 | 0.0041 | | 0.004 | 0.004 | 0.0033 | 0.0033 | 0.0033 | 0.6862 | 0.8376 | 10000 | 2 | 0.05 | -0.3 |
| 0.0027 | 0.0028 | 0.0032 | | 0.0033 | 0.0033 | 0.0029 | 0.003 | 0.003 | 0.8443 | 0.9282 | 10000 | 4 | 0.05 | -0.3 |
| 0.0029 | 0.0028 | 0.0039 | | 0.0038 | 0.0038 | 0.0032 | 0.0033 | 0.0033 | 0.7349 | 0.885 | 5000 | 2 | 0.1 | -0.3 |
| 0.0028 | 0.0028 | 0.0032 | | 0.0032 | 0.0032 | 0.003 | 0.003 | 0.003 | 0.8901 | 0.9392 | 5000 | 4 | 0.1 | -0.3 |
| 0.0014 | 0.0014 | 0.0019 | | 0.0019 | 0.0019 | 0.0016 | 0.0016 | 0.0016 | 0.7393 | 0.8715 | 10000 | 2 | 0.1 | -0.3 |
| 0.0014 | 0.0014 | 0.0016 | | 0.0016 | 0.0016 | 0.0015 | 0.0015 | 0.0015 | 0.8925 | 0.9488 | 10000 | 4 | 0.1 | -0.3 |

**WEB TABLE 14-** Empirical variance, mean of estimated variances and ratio of empirical variance (to that with the whole cohort) of log-relative hazard parameter $\beta_{3}$ for different sampling designs and methods of analysis and variance estimation in 5,000 simulated cohorts

| Cohort | | SCC | | | | SCC.Calib | | | Ratio of empirical variance with the whole cohort to empirical variance with | | $n$ | $K$ | $p_{Y}$ | | $\log\left\{ \pi\left( \tau_{1},\tau_{2};\boldsymbol{x} \right) \right\}$ |
| --- | --- | --- | --- | --- | --- | --- | --- | --- | --- | --- | --- | --- | --- | --- | --- |
| Empir var | $\hat{V}_{\mathrm{Robust}}$ | Empir var | $\hat{V}_{\mathrm{Robust}}$ | $\hat{V}$ | Empir var | | $\hat{V}_{\mathrm{Robust}}$ | $\hat{V}$ | SCC | SCC.Calib |  |  |  |  |  |
| 0.0254 | 0.0248 | 0.0295 | 0.0354 | 0.029 | 0.0284 | | 0.0295 | 0.0284 | 0.8606 | 0.8936 | 5000 | 2 | 0.02 | -3.948 | |
| 0.0255 | 0.0248 | 0.0275 | 0.0298 | 0.0268 | 0.0269 | | 0.0271 | 0.0265 | 0.9255 | 0.9473 | 5000 | 4 | 0.02 | -3.948 | |
| 0.0119 | 0.0122 | 0.0136 | 0.0172 | 0.014 | 0.0133 | | 0.0142 | 0.0137 | 0.87 | 0.8947 | 10000 | 2 | 0.02 | -3.948 | |
| 0.0119 | 0.0122 | 0.0127 | 0.0145 | 0.013 | 0.0125 | | 0.0131 | 0.0129 | 0.9384 | 0.9538 | 10000 | 4 | 0.02 | -3.948 | |
| 0.0097 | 0.0096 | 0.0108 | 0.013 | 0.0109 | 0.0105 | | 0.0109 | 0.0106 | 0.8983 | 0.9288 | 5000 | 2 | 0.05 | -3.046 | |
| 0.0094 | 0.0096 | 0.0099 | 0.0111 | 0.0102 | 0.0098 | | 0.0102 | 0.01 | 0.952 | 0.9644 | 5000 | 4 | 0.05 | -3.046 | |
| 0.0046 | 0.0048 | 0.0052 | 0.0064 | 0.0054 | 0.005 | | 0.0054 | 0.0052 | 0.8931 | 0.9213 | 10000 | 2 | 0.05 | -3.046 | |
| 0.0047 | 0.0048 | 0.0049 | 0.0055 | 0.005 | 0.0048 | | 0.005 | 0.005 | 0.9441 | 0.9608 | 10000 | 4 | 0.05 | -3.046 | |
| 0.0046 | 0.0047 | 0.0052 | 0.0059 | 0.0052 | 0.005 | | 0.0051 | 0.005 | 0.9003 | 0.9268 | 5000 | 2 | 0.1 | -2.377 | |
| 0.0047 | 0.0047 | 0.0048 | 0.0051 | 0.0048 | 0.0048 | | 0.0048 | 0.0048 | 0.9741 | 0.9816 | 5000 | 4 | 0.1 | -2.377 | |
| 0.0023 | 0.0023 | 0.0026 | 0.0029 | 0.0026 | 0.0025 | | 0.0026 | 0.0025 | 0.9076 | 0.9399 | 10000 | 2 | 0.1 | -2.377 | |
| 0.0023 | 0.0023 | 0.0023 | 0.0025 | 0.0024 | 0.0023 | | 0.0024 | 0.0024 | 0.9735 | 0.984 | 10000 | 4 | 0.1 | -2.377 | |

**WEB TABLE 15-** Empirical variance, mean of estimated variances and ratio of empirical variance (to that with the whole cohort) of log-pure risk $\log\left\{ \pi\left( \tau_{1},\tau_{2}; \boldsymbol{x} \right) \right\}$, with ${(\tau}_{1},\tau_{2}]=(0,8]$ and $\boldsymbol{x}=\left( -1, 1, -0.6 \right)'$, for different sampling designs and methods of analysis and variance estimation in 5,000 simulated cohorts

| Cohort | | USCC | | | | USCC.Calib | | | Ratio of empirical variance with the whole cohort to empirical variance with | | $n$ | $K$ | $p_{Y}$ | | $\log\left\{ \pi\left( \tau_{1},\tau_{2};\boldsymbol{x} \right) \right\}$ |
| --- | --- | --- | --- | --- | --- | --- | --- | --- | --- | --- | --- | --- | --- | --- | --- |
| Empir var | $\hat{V}_{\mathrm{Robust}}$ | Empir var | $\hat{V}_{\mathrm{Robust}}$ | $\hat{V}$ | Empir var | | $\hat{V}_{\mathrm{Robust}}$ | $\hat{V}$ | USCC | USCC.Calib |  |  |  |  |  |
| 0.0254 | 0.0248 | 0.0332 | 0.0374 | 0.0329 | 0.0299 | | 0.0305 | 0.0305 | 0.764 | 0.8502 | 5000 | 2 | 0.02 | -3.948 | |
| 0.0255 | 0.0248 | 0.0293 | 0.0307 | 0.0286 | 0.0278 | | 0.0275 | 0.0275 | 0.8689 | 0.9167 | 5000 | 4 | 0.02 | -3.948 | |
| 0.0119 | 0.0122 | 0.0158 | 0.0181 | 0.0159 | 0.014 | | 0.0145 | 0.0145 | 0.7522 | 0.8488 | 10000 | 2 | 0.02 | -3.948 | |
| 0.0119 | 0.0122 | 0.0137 | 0.015 | 0.0139 | 0.0129 | | 0.0133 | 0.0133 | 0.8689 | 0.9233 | 10000 | 4 | 0.02 | -3.948 | |
| 0.0097 | 0.0096 | 0.0124 | 0.0137 | 0.0122 | 0.0111 | | 0.0112 | 0.0112 | 0.7826 | 0.8729 | 5000 | 2 | 0.05 | -3.046 | |
| 0.0094 | 0.0096 | 0.0105 | 0.0114 | 0.0108 | 0.0101 | | 0.0103 | 0.0103 | 0.899 | 0.9377 | 5000 | 4 | 0.05 | -3.046 | |
| 0.0046 | 0.0048 | 0.0058 | 0.0068 | 0.006 | 0.0053 | | 0.0055 | 0.0055 | 0.796 | 0.8763 | 10000 | 2 | 0.05 | -3.046 | |
| 0.0047 | 0.0048 | 0.0052 | 0.0057 | 0.0053 | 0.0049 | | 0.0051 | 0.0051 | 0.9008 | 0.9527 | 10000 | 4 | 0.05 | -3.046 | |
| 0.0046 | 0.0047 | 0.0056 | 0.0062 | 0.0057 | 0.0051 | | 0.0052 | 0.0052 | 0.8338 | 0.9108 | 5000 | 2 | 0.1 | -2.377 | |
| 0.0047 | 0.0047 | 0.005 | 0.0052 | 0.005 | 0.0049 | | 0.0049 | 0.0049 | 0.9289 | 0.9625 | 5000 | 4 | 0.1 | -2.377 | |
| 0.0023 | 0.0023 | 0.0028 | 0.0031 | 0.0028 | 0.0026 | | 0.0026 | 0.0026 | 0.8249 | 0.8978 | 10000 | 2 | 0.1 | -2.377 | |
| 0.0023 | 0.0023 | 0.0024 | 0.0026 | 0.0025 | 0.0024 | | 0.0024 | 0.0024 | 0.9366 | 0.9703 | 10000 | 4 | 0.1 | -2.377 | |

**WEB TABLE 16-** Empirical variance, mean of estimated variances and ratio of empirical variance (to that with the whole cohort) of log-pure risk $\log\left\{ \pi\left( \tau_{1},\tau_{2}; \boldsymbol{x} \right) \right\}$, with ${(\tau}_{1},\tau_{2}]=(0,8]$ and $\boldsymbol{x}=\left( -1, 1, -0.6 \right)'$, for different sampling designs and methods of analysis and variance estimation in 5,000 simulated cohorts

| Cohort | | | SCC | | | SCC.Calib | | | Ratio of empirical variance with the whole cohort to empirical variance with | | $n$ | $K$ | $p_{Y}$ | | $\log\left\{ \pi\left( \tau_{1},\tau_{2};\boldsymbol{x} \right) \right\}$ |
| --- | --- | --- | --- | --- | --- | --- | --- | --- | --- | --- | --- | --- | --- | --- | --- |
| Empir var | $\hat{V}_{\mathrm{Robust}}$ | Empir var | | $\hat{V}_{\mathrm{Robust}}$ | $\hat{V}$ | Empir var | $\hat{V}_{\mathrm{Robust}}$ | $\hat{V}$ | SCC | SCC.Calib |  |  |  |  |  |
| 0.1248 | 0.1249 | 0.1423 | | 0.1773 | 0.1441 | 0.1337 | 0.1367 | 0.1358 | 0.8772 | 0.9335 | 5000 | 2 | 0.02 | -5.201 | |
| 0.1294 | 0.1258 | 0.1382 | | 0.1507 | 0.1349 | 0.133 | 0.1314 | 0.131 | 0.9363 | 0.9728 | 5000 | 4 | 0.02 | -5.201 | |
| 0.0618 | 0.062 | 0.0688 | | 0.0861 | 0.0697 | 0.0649 | 0.0664 | 0.066 | 0.8983 | 0.9525 | 10000 | 2 | 0.02 | -5.201 | |
| 0.062 | 0.0618 | 0.0657 | | 0.0733 | 0.0654 | 0.0634 | 0.0638 | 0.0636 | 0.9438 | 0.9781 | 10000 | 4 | 0.02 | -5.201 | |
| 0.0514 | 0.0496 | 0.0568 | | 0.0666 | 0.0549 | 0.0535 | 0.0523 | 0.0521 | 0.9047 | 0.9606 | 5000 | 2 | 0.05 | -4.288 | |
| 0.0499 | 0.0496 | 0.052 | | 0.0571 | 0.0518 | 0.0507 | 0.0507 | 0.0506 | 0.9599 | 0.9838 | 5000 | 4 | 0.05 | -4.288 | |
| 0.0241 | 0.0247 | 0.0266 | | 0.0329 | 0.0271 | 0.0252 | 0.0258 | 0.0257 | 0.9064 | 0.9573 | 10000 | 2 | 0.05 | -4.288 | |
| 0.0245 | 0.0247 | 0.0255 | | 0.0284 | 0.0257 | 0.0248 | 0.0252 | 0.0252 | 0.9636 | 0.9905 | 10000 | 4 | 0.05 | -4.288 | |
| 0.025 | 0.0248 | 0.027 | | 0.0316 | 0.0268 | 0.0257 | 0.0257 | 0.0256 | 0.9256 | 0.9727 | 5000 | 2 | 0.1 | -3.602 | |
| 0.0243 | 0.0248 | 0.0248 | | 0.0273 | 0.0255 | 0.0245 | 0.0251 | 0.0251 | 0.9789 | 0.9892 | 5000 | 4 | 0.1 | -3.602 | |
| 0.0125 | 0.0124 | 0.0133 | | 0.0157 | 0.0133 | 0.0128 | 0.0128 | 0.0127 | 0.9411 | 0.9794 | 10000 | 2 | 0.1 | -3.602 | |
| 0.0122 | 0.0124 | 0.0125 | | 0.0136 | 0.0127 | 0.0123 | 0.0125 | 0.0125 | 0.9762 | 0.99 | 10000 | 4 | 0.1 | -3.602 | |

**WEB TABLE 17-** Empirical variance, mean of estimated variances and ratio of empirical variance (to that with the whole cohort) of log-pure risk $\log\left\{ \pi\left( \tau_{1},\tau_{2}; \boldsymbol{x} \right) \right\}$, with ${(\tau}_{1},\tau_{2}]=(0,8]$ and $\boldsymbol{x}=\left( 1, -1, 0.6 \right)'$, for different sampling designs and methods of analysis and variance estimation in 5,000 simulated cohorts

| Cohort | | USCC | | | | USCC.Calib | | | Ratio of empirical variance with the whole cohort to empirical variance with | | $n$ | $K$ | $p_{Y}$ | | $\log\left\{ \pi\left( \tau_{1},\tau_{2};\boldsymbol{x} \right) \right\}$ |
| --- | --- | --- | --- | --- | --- | --- | --- | --- | --- | --- | --- | --- | --- | --- | --- |
| Empir var | $\hat{V}_{\mathrm{Robust}}$ | Empir var | $\hat{V}_{\mathrm{Robust}}$ | $\hat{V}$ | Empir var | | $\hat{V}_{\mathrm{Robust}}$ | $\hat{V}$ | USCC | USCC.Calib |  |  |  |  |  |
| 0.1248 | 0.1249 | 0.1388 | 0.1788 | 0.1744 | 0.1375 | | 0.1411 | 0.1410 | 0.7233 | 0.9073 | 5000 | 2 | 0.02 | -5.201 | |
| 0.1294 | 0.1258 | 0.1343 | 0.1511 | 0.1489 | 0.1356 | | 0.1328 | 0.1328 | 0.85 | 0.9542 | 5000 | 4 | 0.02 | -5.201 | |
| 0.0618 | 0.062 | 0.0662 | 0.086 | 0.0837 | 0.0676 | | 0.0676 | 0.0676 | 0.7518 | 0.9152 | 10000 | 2 | 0.02 | -5.201 | |
| 0.062 | 0.0618 | 0.0646 | 0.0731 | 0.072 | 0.0643 | | 0.0644 | 0.0644 | 0.8604 | 0.9647 | 10000 | 4 | 0.02 | -5.201 | |
| 0.0514 | 0.0496 | 0.0551 | 0.0661 | 0.0645 | 0.0546 | | 0.0532 | 0.0532 | 0.795 | 0.9411 | 5000 | 2 | 0.05 | -4.288 | |
| 0.0499 | 0.0496 | 0.0513 | 0.0569 | 0.0562 | 0.0515 | | 0.0512 | 0.0512 | 0.8882 | 0.9699 | 5000 | 4 | 0.05 | -4.288 | |
| 0.0241 | 0.0247 | 0.026 | 0.0325 | 0.0317 | 0.0257 | | 0.0261 | 0.0261 | 0.7713 | 0.9385 | 10000 | 2 | 0.05 | -4.288 | |
| 0.0245 | 0.0247 | 0.0252 | 0.0282 | 0.0278 | 0.025 | | 0.0254 | 0.0254 | 0.8908 | 0.9804 | 10000 | 4 | 0.05 | -4.288 | |
| 0.025 | 0.0248 | 0.0261 | 0.0311 | 0.0304 | 0.0261 | | 0.026 | 0.026 | 0.8188 | 0.9543 | 5000 | 2 | 0.1 | -3.602 | |
| 0.0243 | 0.0248 | 0.0247 | 0.0271 | 0.0268 | 0.0246 | | 0.0252 | 0.0252 | 0.9362 | 0.9862 | 5000 | 4 | 0.1 | -3.602 | |
| 0.0125 | 0.0124 | 0.013 | 0.0154 | 0.0151 | 0.013 | | 0.0129 | 0.0129 | 0.8257 | 0.9591 | 10000 | 2 | 0.1 | -3.602 | |
| 0.0122 | 0.0124 | 0.0124 | 0.0134 | 0.0133 | 0.0124 | | 0.0125 | 0.0125 | 0.92 | 0.9842 | 10000 | 4 | 0.1 | -3.602 | |

**WEB TABLE 18-** Empirical variance, mean of estimated variances and ratio of empirical variance (to that with the whole cohort) of log-pure risk $\log\left\{ \pi\left( \tau_{1},\tau_{2}; \boldsymbol{x} \right) \right\}$, with ${(\tau}_{1},\tau_{2}]=(0,8]$ and $\boldsymbol{x}=\left( 1, -1, 0.6 \right)'$, for different sampling designs and methods of analysis and variance estimation in 5,000 simulated cohorts

| Cohort | | SCC | | | | SCC.Calib | | | Ratio of empirical variance with the whole cohort to empirical variance with | | $n$ | $K$ | $p_{Y}$ | | $\log\left\{ \pi\left( \tau_{1},\tau_{2};\boldsymbol{x} \right) \right\}$ |
| --- | --- | --- | --- | --- | --- | --- | --- | --- | --- | --- | --- | --- | --- | --- | --- |
| Empir var | $\hat{V}_{\mathrm{Robust}}$ | Empir var | $\hat{V}_{\mathrm{Robust}}$ | $\hat{V}$ | Empir var | | $\hat{V}_{\mathrm{Robust}}$ | $\hat{V}$ | SCC | SCC.Calib |  |  |  |  |  |
| 0.0537 | 0.0557 | 0.0668 | 0.0789 | 0.0698 | 0.0624 | | 0.0668 | 0.0662 | 0.8032 | 0.8605 | 5000 | 2 | 0.02 | -4.702 | |
| 0.058 | 0.0559 | 0.0648 | 0.067 | 0.0626 | 0.062 | | 0.0611 | 0.0608 | 0.8962 | 0.9355 | 5000 | 4 | 0.02 | -4.702 | |
| 0.0274 | 0.0277 | 0.0326 | 0.0379 | 0.0333 | 0.0308 | | 0.0318 | 0.0315 | 0.8421 | 0.8899 | 10000 | 2 | 0.02 | -4.702 | |
| 0.0277 | 0.0277 | 0.0303 | 0.0325 | 0.0303 | 0.0292 | | 0.0296 | 0.0295 | 0.914 | 0.9499 | 10000 | 4 | 0.02 | -4.702 | |
| 0.0226 | 0.0221 | 0.0265 | 0.0291 | 0.0259 | 0.0251 | | 0.0248 | 0.0246 | 0.8506 | 0.8988 | 5000 | 2 | 0.05 | -3.793 | |
| 0.0218 | 0.0221 | 0.0233 | 0.0251 | 0.0237 | 0.0227 | | 0.0232 | 0.0231 | 0.935 | 0.9607 | 5000 | 4 | 0.05 | -3.793 | |
| 0.0111 | 0.011 | 0.0128 | 0.0143 | 0.0127 | 0.0121 | | 0.0122 | 0.0121 | 0.8618 | 0.9162 | 10000 | 2 | 0.05 | -3.793 | |
| 0.0111 | 0.011 | 0.0118 | 0.0125 | 0.0117 | 0.0114 | | 0.0115 | 0.0115 | 0.9405 | 0.9752 | 10000 | 4 | 0.05 | -3.793 | |
| 0.0114 | 0.011 | 0.0126 | 0.0136 | 0.0123 | 0.0121 | | 0.0119 | 0.0118 | 0.9051 | 0.9416 | 5000 | 2 | 0.1 | -3.111 | |
| 0.0109 | 0.011 | 0.0113 | 0.0119 | 0.0114 | 0.0112 | | 0.0113 | 0.0113 | 0.9625 | 0.9763 | 5000 | 4 | 0.1 | -3.111 | |
| 0.0055 | 0.0055 | 0.0061 | 0.0068 | 0.0061 | 0.0058 | | 0.0059 | 0.0059 | 0.9004 | 0.9415 | 10000 | 2 | 0.1 | -3.111 | |
| 0.0054 | 0.0055 | 0.0056 | 0.0059 | 0.0057 | 0.0055 | | 0.0056 | 0.0056 | 0.963 | 0.9828 | 10000 | 4 | 0.1 | -3.111 | |

**WEB TABLE 19-** Empirical variance, mean of estimated variances and ratio of empirical variance (to that with the whole cohort) of log-pure risk $\log\left\{ \pi\left( \tau_{1},\tau_{2}; \boldsymbol{x} \right) \right\}$, with ${(\tau}_{1},\tau_{2}]=(0,8]$ and $\boldsymbol{x}=\left( 1, 1, 0.6 \right)'$, for different sampling designs and methods of analysis and variance estimation in 5,000 simulated cohorts

| Cohort | | USCC | | | USCC.Calib | | | Ratio of empirical variance with the whole cohort to empirical variance with | | $n$ | $K$ | $p_{Y}$ | | $\log\left\{ \pi\left( \tau_{1},\tau_{2};\boldsymbol{x} \right) \right\}$ |
| --- | --- | --- | --- | --- | --- | --- | --- | --- | --- | --- | --- | --- | --- | --- |
| Empir var | $\hat{V}_{\mathrm{Robust}}$ | Empir var | $\hat{V}_{\mathrm{Robust}}$ | $\hat{V}$ | Empir var | $\hat{V}_{\mathrm{Robust}}$ | $\hat{V}$ | USCC | USCC.Calib |  |  |  |  |  |
| 0.0537 | 0.0557 | 0.0721 | 0.0806 | 0.0761 | 0.064 | 0.0687 | 0.0687 | 0.7448 | 0.838 | 5000 | 2 | 0.02 | -4.702 | |
| 0.058 | 0.0559 | 0.0678 | 0.0679 | 0.0657 | 0.0633 | 0.062 | 0.062 | 0.8557 | 0.9169 | 5000 | 4 | 0.02 | -4.702 | |
| 0.0274 | 0.0277 | 0.0361 | 0.0386 | 0.0363 | 0.0323 | 0.0327 | 0.0327 | 0.7589 | 0.8482 | 10000 | 2 | 0.02 | -4.702 | |
| 0.0277 | 0.0277 | 0.0315 | 0.0328 | 0.0317 | 0.0299 | 0.03 | 0.03 | 0.8793 | 0.9265 | 10000 | 4 | 0.02 | -4.702 | |
| 0.0226 | 0.0221 | 0.028 | 0.0294 | 0.0278 | 0.0253 | 0.0252 | 0.0252 | 0.8069 | 0.8921 | 5000 | 2 | 0.05 | -3.793 | |
| 0.0218 | 0.0221 | 0.0246 | 0.0253 | 0.0246 | 0.0232 | 0.0235 | 0.0235 | 0.8868 | 0.9409 | 5000 | 4 | 0.05 | -3.793 | |
| 0.0111 | 0.011 | 0.014 | 0.0145 | 0.0136 | 0.0126 | 0.0124 | 0.0124 | 0.7903 | 0.8793 | 10000 | 2 | 0.05 | -3.793 | |
| 0.0111 | 0.011 | 0.0123 | 0.0125 | 0.0122 | 0.0116 | 0.0116 | 0.0116 | 0.9017 | 0.9556 | 10000 | 4 | 0.05 | -3.793 | |
| 0.0114 | 0.011 | 0.0135 | 0.0137 | 0.013 | 0.0123 | 0.0121 | 0.0121 | 0.8434 | 0.9281 | 5000 | 2 | 0.1 | -3.111 | |
| 0.0109 | 0.011 | 0.0116 | 0.0119 | 0.0117 | 0.0114 | 0.0114 | 0.0114 | 0.9405 | 0.959 | 5000 | 4 | 0.1 | -3.111 | |
| 0.0055 | 0.0055 | 0.0066 | 0.0068 | 0.0065 | 0.006 | 0.006 | 0.006 | 0.8318 | 0.9191 | 10000 | 2 | 0.1 | -3.111 | |
| 0.0054 | 0.0055 | 0.0058 | 0.0059 | 0.0058 | 0.0057 | 0.0056 | 0.0056 | 0.9306 | 0.9581 | 10000 | 4 | 0.1 | -3.111 | |

**WEB TABLE 20-** Empirical variance, mean of estimated variances and ratio of empirical variance (to that with the whole cohort) of log-pure risk $\log\left\{ \pi\left( \tau_{1},\tau_{2}; \boldsymbol{x} \right) \right\}$, with ${(\tau}_{1},\tau_{2}]=(0,8]$ and $\boldsymbol{x}=\left( 1, 1, 0.6 \right)'$, for different sampling designs and methods of analysis and variance estimation in 5,000 simulated cohorts

## Estimation of the phase-two component of the variance

$\hat{V}$ corresponds to the sum of the estimated phase-one component and phase-two component of the variance, as in Equation (14) in Section 3.3 in the Main Document for SCC and USCC, and as in Equation (18) in Section 4.3 in the Main Document for SCC.Calib and USCC.Calib (with simplified versions of these equations when $J = 1$ for USCC and USCC.Calib).

An empirical estimate of the phase-two component of the variance can be obtained by averaging over the 5,000 replications the mean squared differences between the parameter estimate (from SCC, USCC, SCC.Calib or USCC.Calib) and that obtained with the entire cohort (Breslow et al., 2009; Lin, 2000).

**WEB TABLE 21** to **WEB TABLE 26** display the empirical phase-two component of the variance and the mean of estimated phase-two components of the variance for $\beta_{1}$, $\beta_{2}$, $\beta_{3}$ and $\log\left\{ \pi\left( \tau_{1},\tau_{2}; \boldsymbol{x} \right) \right\}$ for ${(\tau}_{1},\tau_{2}]=(0,8]$ and $\boldsymbol{x}\in\left\{ \left( -1, 1, -0.6 \right)',\left( 1, -1, 0.6 \right)',\left( 1, 1, 0.6 \right)' \right\}$, respectively. The estimated and empirical quantities agree well, and thus suggest an appropriate estimation of the phase-two component of the variance with Equation (14) and Equation (18) in Section 3.3 and Section 4.3 in the Main Document (for design weights or calibrated weights, respectively). And as expected, the phase-two component of the variance is smaller with calibrated weights than with design weights.

## Additional simulations, with design weights poststratified on the number of non-cases

In the simulation in Section 7 in the Main Document and in Web Appendix D.2, the results for SCC and USCC were obtained from using the design weights. In the literature, several authors computed the design weights after having redefined the strata by excluding the cases (Borgan et al., 2000; Samuelsen et al., 2007); this amounts to poststratification of the weights on the number of non-cases. In this Web Appendix, we performed the same simulations as before, except that in addition to estimation with SCC and USCC, estimation was also performed using the stratified case-cohort with post-stratified weights (SCC.Poststrat); and the unstratified case-cohort with post-stratified weights (USCC.Poststrat).

The ratio of the empirical variance with SCC to the empirical variance with SCC.Poststrat and the ratio of the empirical variance with USCC to the empirical variance with USCC.Poststrat, are displayed for $\beta_{1}$, $\beta_{2}$ and $\beta_{3}$ (**WEB TABLE 27)** and for $\pi\left( \tau_{1},\tau_{2}; \boldsymbol{x} \right)$, with ${(\tau}_{1},\tau_{2}]=(0,8]$ and $\boldsymbol{x}\in\left\{ \left( -1, 1, -0.6 \right)',\left( 1, -1, 0.6 \right)',\left( 1, 1, 0.6 \right)' \right\}$ (**WEB TABLE 28**). These ratios measure the efficiency gain from using the post-stratified weights instead of the design weights, with the stratified case-cohort and with the unstratified case-cohort designs, respectively. The ratios are all close to one, indicating almost no improvement with poststratification of the weights on the number of non-cases.

## Additional simulations, with weights calibrated using weaker proxies

In the simulations in Section 7 in the Main Document and in Web Appendix D.2, the results for SCC.Calib were obtained by using proxies $\tilde{\boldsymbol{X}}=\left( \tilde{X}_{1},\tilde{X}_{3} \right)'$, with $\tilde{X}_{1}=X_{1}+\varepsilon_{1}$, $\varepsilon_{1}\mathcal{\sim N}(0,{0.75}^{2})$, and thus such that $\mathrm{corr}\left( \tilde{X}_{1},X_{1} \right)=0.8$. In this Section, we performed the same simulations, except that we used $\varepsilon_{1}\mathcal{\sim N}(0,{1.2}^{2})$, so that $\mathrm{corr}\left( \tilde{X}_{1},X_{1} \right)\approx0.64$.

**WEB TABLE 29** and **WEB TABLE 30** display the estimation results with SCC.Calib for the log-relative hazard $\beta_{1}$ and the pure risk with profile $\boldsymbol{x=}\left( -1, 1, -0.6 \right)'$, respectively. Because of the weaker proxy for $X_{1}$, the robust variance formula overestimated the variance and yielded supra-nominal confidence interval coverage in many scenarios. In addition, the efficiency gain is more modest than with the stronger proxy; see empirical variances in **WEB TABLE 9** and **WEB TABLE 15** in Web Appendix D.2.

| SCC | | SCC.Calib | | USCC | | USCC.Calib | | $n$ | $K$ | $p_{Y}$ | $\beta_{1}$ |
| --- | --- | --- | --- | --- | --- | --- | --- | --- | --- | --- | --- |
| Empir phase-two var | Phase-two comp in $\hat{V}$ | Empir phase-two var | Phase-two comp in $\hat{V}$ | Empir phase-two var | Phase-two comp in $\hat{V}$ | Empir phase-two var | Phase-two comp in $\hat{V}$ |  |  |  |  |
| 0.00335 | 0.00319 | 0.00226 | 0.00228 | 0.00724 | 0.00689 | 0.00313 | 0.00304 | 5000 | 2 | 0.02 | -0.2 |
| 0.00159 | 0.0015 | 0.00108 | 0.00107 | 0.00342 | 0.00325 | 0.00145 | 0.00141 | 5000 | 4 | 0.02 | -0.2 |
| 0.0016 | 0.00148 | 0.00108 | 0.00104 | 0.00348 | 0.00331 | 0.00138 | 0.00138 | 10000 | 2 | 0.02 | -0.2 |
| 0.00072 | 0.00071 | 0.00050 | 0.00049 | 0.00161 | 0.00157 | 0.00067 | 0.00065 | 10000 | 4 | 0.02 | -0.2 |
| 0.00108 | 0.00105 | 0.00072 | 0.00072 | 0.00246 | 0.00234 | 0.00098 | 0.00096 | 5000 | 2 | 0.05 | -0.2 |
| 0.00044 | 0.00045 | 0.00031 | 0.00031 | 0.00101 | 0.00103 | 0.00042 | 0.00042 | 5000 | 4 | 0.05 | -0.2 |
| 0.00051 | 0.00051 | 0.00036 | 0.00035 | 0.00116 | 0.00115 | 0.00046 | 0.00046 | 10000 | 2 | 0.05 | -0.2 |
| 0.00022 | 0.00022 | 0.00015 | 0.00015 | 0.00050 | 0.00051 | 0.00021 | 0.00020 | 10000 | 4 | 0.05 | -0.2 |
| 0.00042 | 0.00042 | 0.00027 | 0.00027 | 0.00095 | 0.00093 | 0.00038 | 0.00038 | 5000 | 2 | 0.1 | -0.2 |
| 0.00014 | 0.00014 | 9.00E-05 | 9.00E-05 | 0.00034 | 0.00033 | 0.00014 | 0.00013 | 5000 | 4 | 0.1 | -0.2 |
| 0.00022 | 0.00021 | 0.00013 | 0.00013 | 0.00046 | 0.00046 | 0.00019 | 0.00019 | 10000 | 2 | 0.1 | -0.2 |
| 7.00E-05 | 7.00E-05 | 4.00E-05 | 4.00E-05 | 0.00017 | 0.00017 | 7.00E-05 | 7.00E-05 | 10000 | 4 | 0.1 | -0.2 |

**WEB TABLE 21-** Empirical phase-two component of the variance and mean of estimated phase-two components of the variance of log-relative hazard parameter $\beta_{1}$ for different sampling designs and methods of analysis and variance estimation in 5,000 simulated cohorts

| SCC | | SCC.Calib | | USCC | | USCC.Calib | | $n$ | $K$ | $p_{Y}$ | $\beta_{2}$ |
| --- | --- | --- | --- | --- | --- | --- | --- | --- | --- | --- | --- |
| Empir phase-two var | Phase-two comp in $\hat{V}$ | Empir phase-two var | Phase-two comp in $\hat{V}$ | Empir phase-two var | Phase-two comp in $\hat{V}$ | Empir phase-two var | Phase-two comp in $\hat{V}$ |  |  |  |  |
| 0.00321 | 0.00314 | 0.00085 | 0.00084 | 0.00851 | 0.00838 | 0.00082 | 0.00085 | 5000 | 2 | 0.02 | 0.25 |
| 0.00153 | 0.00148 | 0.00039 | 0.00039 | 0.00399 | 0.00395 | 0.00037 | 0.00038 | 5000 | 4 | 0.02 | 0.25 |
| 0.00152 | 0.00146 | 0.00034 | 0.00035 | 0.00396 | 0.00405 | 0.00034 | 0.00034 | 10000 | 2 | 0.02 | 0.25 |
| 0.00074 | 7.00E-04 | 0.00017 | 0.00017 | 0.00198 | 0.00192 | 0.00016 | 0.00015 | 10000 | 4 | 0.02 | 0.25 |
| 0.00113 | 0.00109 | 0.00026 | 0.00025 | 0.00287 | 0.00288 | 0.00022 | 0.00023 | 5000 | 2 | 0.05 | 0.25 |
| 0.00047 | 0.00048 | 0.00011 | 0.00011 | 0.00125 | 0.00126 | 0.00010 | 0.00010 | 5000 | 4 | 0.05 | 0.25 |
| 0.00054 | 0.00053 | 0.00012 | 0.00012 | 0.00142 | 0.00142 | 0.00010 | 0.00010 | 10000 | 2 | 0.05 | 0.25 |
| 0.00025 | 0.00024 | 5.00E-05 | 5.00E-05 | 6.00E-04 | 0.00063 | 4.00E-05 | 4.00E-05 | 10000 | 4 | 0.05 | 0.25 |
| 0.00047 | 0.00047 | 0.00010 | 0.00010 | 0.00115 | 0.00116 | 8.00E-05 | 8.00E-05 | 5000 | 2 | 0.1 | 0.25 |
| 0.00017 | 0.00017 | 4.00E-05 | 4.00E-05 | 0.00041 | 0.00041 | 3.00E-05 | 3.00E-05 | 5000 | 4 | 0.1 | 0.25 |
| 0.00024 | 0.00023 | 5.00E-05 | 5.00E-05 | 0.00058 | 0.00058 | 4.00E-05 | 4.00E-05 | 10000 | 2 | 0.1 | 0.25 |
| 8.00E-05 | 8.00E-05 | 2.00E-05 | 2.00E-05 | 2.00E-04 | 0.00021 | 1.00E-05 | 1.00E-05 | 10000 | 4 | 0.1 | 0.25 |

**WEB TABLE 22-** Empirical phase-two component of the variance and mean of estimated phase-two components of the variance of log-relative hazard parameter $\beta_{2}$ for different sampling designs and methods of analysis and variance estimation in 5,000 simulated cohorts

| SCC | | SCC.Calib | | USCC | | USCC.Calib | | $n$ | $K$ | $p_{Y}$ | $\beta_{3}$ |
| --- | --- | --- | --- | --- | --- | --- | --- | --- | --- | --- | --- |
| Empir phase-two var | Phase-two comp in $\hat{V}$ | Empir phase-two var | Phase-two comp in $\hat{V}$ | Empir phase-two var | Phase-two comp in $\hat{V}$ | Empir phase-two var | Phase-two comp in $\hat{V}$ |  |  |  |  |
| 0.00689 | 0.0065 | 0.00303 | 0.00289 | 0.00757 | 0.0072 | 0.00336 | 0.00326 | 5000 | 2 | 0.02 | -0.3 |
| 0.00317 | 0.00306 | 0.00138 | 0.00135 | 0.00363 | 0.00341 | 0.00159 | 0.00152 | 5000 | 4 | 0.02 | -0.3 |
| 0.00322 | 0.00310 | 0.00128 | 0.00132 | 0.00359 | 0.00347 | 0.00152 | 0.00149 | 10000 | 2 | 0.02 | -0.3 |
| 0.00157 | 0.00148 | 0.00065 | 0.00063 | 0.00174 | 0.00165 | 0.00071 | 0.00070 | 10000 | 4 | 0.02 | -0.3 |
| 0.0023 | 0.00218 | 0.00097 | 0.00092 | 0.00248 | 0.00244 | 0.00104 | 0.00103 | 5000 | 2 | 0.05 | -0.3 |
| 0.00092 | 0.00094 | 0.00039 | 0.00040 | 0.00112 | 0.00108 | 0.00048 | 0.00045 | 5000 | 4 | 0.05 | -0.3 |
| 0.00116 | 0.00108 | 0.00046 | 0.00045 | 0.00119 | 0.00121 | 0.00049 | 0.00050 | 10000 | 2 | 0.05 | -0.3 |
| 0.00046 | 0.00047 | 0.00019 | 0.00019 | 0.00053 | 0.00053 | 0.00022 | 0.00022 | 10000 | 4 | 0.05 | -0.3 |
| 0.00086 | 0.00085 | 0.00035 | 0.00035 | 0.00104 | 0.00097 | 0.00043 | 0.00041 | 5000 | 2 | 0.1 | -0.3 |
| 0.00028 | 0.00029 | 0.00012 | 0.00012 | 0.00035 | 0.00035 | 0.00015 | 0.00014 | 5000 | 4 | 0.1 | -0.3 |
| 0.00043 | 0.00042 | 0.00018 | 0.00017 | 0.00049 | 0.00048 | 0.00020 | 0.00020 | 10000 | 2 | 0.1 | -0.3 |
| 0.00014 | 0.00014 | 6.00E-05 | 6.00E-05 | 0.00017 | 0.00017 | 7.00E-05 | 7.00E-05 | 10000 | 4 | 0.1 | -0.3 |

**WEB TABLE 23-** Empirical phase-two component of the variance and mean of estimated phase-two components of the variance of log-relative hazard parameter $\beta_{3}$ for different sampling designs and methods of analysis and variance estimation in 5,000 simulated cohorts

| SCC | | SCC.Calib | | USCC | | USCC.Calib | | $n$ | $K$ | $p_{Y}$ | $\log\left\{ \pi\left( \tau_{1},\tau_{2}; \boldsymbol{x} \right) \right\}$ |
| --- | --- | --- | --- | --- | --- | --- | --- | --- | --- | --- | --- |
| Empir phase-two var | Phase-two comp in $\hat{V}$ | Empir phase-two var | Phase-two comp in $\hat{V}$ | Empir phase-two var | Phase-two comp in $\hat{V}$ | Empir phase-two var | Phase-two comp in $\hat{V}$ |  |  |  |  |
| 0.00351 | 0.00333 | 0.00259 | 0.00259 | 0.00744 | 0.00709 | 0.00425 | 0.00418 | 5000 | 2 | 0.02 | -3.948 |
| 0.00166 | 0.00155 | 0.00126 | 0.00121 | 0.00356 | 0.00332 | 0.00197 | 0.00193 | 5000 | 4 | 0.02 | -3.948 |
| 0.00171 | 0.00157 | 0.00123 | 0.00118 | 0.00356 | 0.00341 | 0.00194 | 0.00192 | 10000 | 2 | 0.02 | -3.948 |
| 0.00075 | 0.00074 | 0.00056 | 0.00056 | 0.00167 | 0.00161 | 0.00095 | 0.00090 | 10000 | 4 | 0.02 | -3.948 |
| 0.00115 | 0.00113 | 0.00081 | 0.00081 | 0.00253 | 0.0024 | 0.00131 | 0.0013 | 5000 | 2 | 0.05 | -3.046 |
| 0.00047 | 0.00048 | 0.00033 | 0.00035 | 0.00104 | 0.00105 | 0.00058 | 0.00057 | 5000 | 4 | 0.05 | -3.046 |
| 0.00055 | 0.00056 | 0.00040 | 0.00039 | 0.00118 | 0.00118 | 0.00063 | 0.00063 | 10000 | 2 | 0.05 | -3.046 |
| 0.00024 | 0.00024 | 0.00017 | 0.00017 | 0.00052 | 0.00052 | 0.00028 | 0.00028 | 10000 | 4 | 0.05 | -3.046 |
| 0.00046 | 0.00045 | 0.00030 | 0.00030 | 0.00098 | 0.00095 | 0.00049 | 0.00049 | 5000 | 2 | 0.1 | -2.377 |
| 0.00015 | 0.00014 | 0.00010 | 0.00010 | 0.00035 | 0.00034 | 0.00017 | 0.00017 | 5000 | 4 | 0.1 | -2.377 |
| 0.00023 | 0.00022 | 0.00015 | 0.00015 | 0.00047 | 0.00047 | 0.00024 | 0.00024 | 10000 | 2 | 0.1 | -2.377 |
| 7.00E-05 | 7.00E-05 | 5.00E-05 | 5.00E-05 | 0.00017 | 0.00017 | 9.00E-05 | 9.00E-05 | 10000 | 4 | 0.1 | -2.377 |

**WEB TABLE 24-** Empirical phase-two component of the variance and mean of estimated phase-two components of the variance of log-pure risk $\log\left\{ \pi\left( \tau_{1},\tau_{2}; \boldsymbol{x} \right) \right\}$, with ${(\tau}_{1},\tau_{2}]=(0,8]$ and $\boldsymbol{x}=\left( -1, 1, -0.6 \right)'$, for different sampling designs and methods of analysis and variance estimation in 5,000 simulated cohorts

| SCC | | SCC.Calib | | USCC | | USCC.Calib | | $n$ | $K$ | $p_{Y}$ | $\log\left\{ \pi\left( \tau_{1},\tau_{2}; \boldsymbol{x} \right) \right\}$ |
| --- | --- | --- | --- | --- | --- | --- | --- | --- | --- | --- | --- |
| Empir phase-two var | Phase-two comp in $\hat{V}$ | Empir phase-two var | Phase-two comp in $\hat{V}$ | Empir phase-two var | Phase-two comp in $\hat{V}$ | Empir phase-two var | Phase-two comp in $\hat{V}$ |  |  |  |  |
| 0.01449 | 0.01409 | 0.00653 | 0.00678 | 0.04344 | 0.04106 | 0.00978 | 0.00949 | 5000 | 2 | 0.02 | -5.201 |
| 0.00717 | 0.00657 | 0.00311 | 0.00313 | 0.02038 | 0.01939 | 0.00447 | 0.00432 | 5000 | 4 | 0.02 | -5.201 |
| 0.00689 | 0.00647 | 0.00294 | 0.0029 | 0.0198 | 0.0197 | 0.00418 | 0.00405 | 10000 | 2 | 0.02 | -5.201 |
| 0.00316 | 0.00307 | 0.0014 | 0.00136 | 0.00987 | 0.00937 | 0.00192 | 0.0019 | 10000 | 4 | 0.02 | -5.201 |
| 0.00484 | 0.00464 | 0.00192 | 0.00192 | 0.01414 | 0.01378 | 0.00285 | 0.00271 | 5000 | 2 | 0.05 | -4.288 |
| 0.002 | 0.00199 | 0.00081 | 8.00E-04 | 0.00625 | 0.00607 | 0.0012 | 0.00119 | 5000 | 4 | 0.05 | -4.288 |
| 0.00224 | 0.00226 | 0.00094 | 9.00E-04 | 0.00686 | 0.0068 | 0.00126 | 0.00128 | 10000 | 2 | 0.05 | -4.288 |
| 0.00099 | 0.00097 | 0.00037 | 0.00038 | 0.00286 | 0.00299 | 0.00058 | 0.00056 | 10000 | 4 | 0.05 | -4.288 |
| 0.00192 | 0.00188 | 0.00070 | 0.00068 | 0.00541 | 0.00541 | 0.00101 | 0.00101 | 5000 | 2 | 0.1 | -3.602 |
| 0.00060 | 0.00061 | 0.00020 | 0.00020 | 0.00193 | 0.00193 | 0.00037 | 0.00036 | 5000 | 4 | 0.1 | -3.602 |
| 0.00094 | 0.00093 | 0.00031 | 0.00033 | 0.00271 | 0.00269 | 0.00049 | 0.00049 | 10000 | 2 | 0.1 | -3.602 |
| 0.00030 | 0.00030 | 0.00010 | 0.00010 | 0.00094 | 0.00096 | 0.00018 | 0.00017 | 10000 | 4 | 0.1 | -3.602 |

**WEB TABLE 25-** Empirical phase-two component of the variance and mean of estimated phase-two components of the variance of log-pure risk $\log\left\{ \pi\left( \tau_{1},\tau_{2}; \boldsymbol{x} \right) \right\}$, with ${(\tau}_{1},\tau_{2}]=(0,8]$ and $\boldsymbol{x}=\left( 1, -1, 0.6 \right)'$, for different sampling designs and methods of analysis and variance estimation in 5,000 simulated cohorts

| SCC | | SCC.Calib | | USCC | | USCC.Calib | | $n$ | $K$ | $p_{Y}$ | $\log\left\{ \pi\left( \tau_{1},\tau_{2}; \boldsymbol{x} \right) \right\}$ |
| --- | --- | --- | --- | --- | --- | --- | --- | --- | --- | --- | --- |
| Empir phase-two var | Phase-two comp in $\hat{V}$ | Empir phase-two var | Phase-two comp in $\hat{V}$ | Empir phase-two var | Phase-two comp in $\hat{V}$ | Empir phase-two var | Phase-two comp in $\hat{V}$ |  |  |  |  |
| 0.01111 | 0.01003 | 0.00703 | 0.0068 | 0.01593 | 0.01523 | 0.00894 | 0.00863 | 5000 | 2 | 0.02 | -4.702 |
| 0.00504 | 0.00471 | 0.00316 | 0.00317 | 0.00787 | 0.00727 | 0.00409 | 0.00402 | 5000 | 4 | 0.02 | -4.702 |
| 0.00516 | 0.00468 | 0.00301 | 0.00301 | 0.00769 | 0.00728 | 0.00395 | 0.00383 | 10000 | 2 | 0.02 | -4.702 |
| 0.00229 | 0.00222 | 0.00147 | 0.00142 | 0.00367 | 0.00347 | 0.00182 | 0.00181 | 10000 | 4 | 0.02 | -4.702 |
| 0.00338 | 0.00321 | 0.00208 | 0.00202 | 0.00525 | 0.00502 | 0.00265 | 0.00257 | 5000 | 2 | 0.05 | -3.793 |
| 0.00134 | 0.00138 | 0.00086 | 0.00086 | 0.00224 | 0.00222 | 0.00118 | 0.00113 | 5000 | 4 | 0.05 | -3.793 |
| 0.00168 | 0.00157 | 0.00099 | 0.00097 | 0.00249 | 0.00247 | 0.00121 | 0.00124 | 10000 | 2 | 0.05 | -3.793 |
| 0.00068 | 0.00067 | 0.00041 | 0.00041 | 0.00106 | 0.00109 | 0.00055 | 0.00054 | 10000 | 4 | 0.05 | -3.793 |
| 0.00121 | 0.00122 | 0.00074 | 0.00073 | 0.00207 | 0.00195 | 0.00101 | 0.00096 | 5000 | 2 | 0.1 | -3.111 |
| 0.00039 | 0.00039 | 0.00024 | 0.00024 | 0.00069 | 7.00E-04 | 0.00035 | 0.00034 | 5000 | 4 | 0.1 | -3.111 |
| 0.00061 | 0.00060 | 0.00035 | 0.00036 | 0.00099 | 0.00096 | 0.00047 | 0.00047 | 10000 | 2 | 0.1 | -3.111 |
| 0.00019 | 0.00019 | 0.00012 | 0.00012 | 0.00035 | 0.00034 | 0.00017 | 0.00017 | 10000 | 4 | 0.1 | -3.111 |

**WEB TABLE 26-** Empirical phase-two component of the variance and mean of estimated phase-two components of the variance of log-pure risk $\log\left\{ \pi\left( \tau_{1},\tau_{2}; \boldsymbol{x} \right) \right\}$, with ${(\tau}_{1},\tau_{2}]=(0,8]$ and $\boldsymbol{x}=\left( 1, 1, 0.6 \right)'$, for different sampling designs and methods of analysis and variance estimation in 5,000 simulated cohorts

| $\beta_{1}$ | | $\beta_{2}$ | | $\beta_{3}$ | | $n$ | $K$ | $p_{Y}$ |
| --- | --- | --- | --- | --- | --- | --- | --- | --- |
| SCC / SCC.Poststrat | USCC / USCC.Poststrat | SCC / SCC.Poststrat | USCC / USCC.Poststrat | SCC / SCC.Poststrat | USCC / USCC.Poststrat |  |  |  |
| 1.002 | 1 | 1.001 | 1 | 1 | 1 | 5000 | 2 | 0.02 |
| 1.003 | 1 | 1.003 | 1 | 1 | 1 | 5000 | 4 | 0.02 |
| 1.001 | 1 | 1.004 | 1 | 1 | 1 | 10000 | 2 | 0.02 |
| 1 | 1 | 1.002 | 1 | 1 | 1 | 10000 | 4 | 0.02 |
| 1.005 | 1 | 1.004 | 1 | 1 | 1 | 5000 | 2 | 0.05 |
| 1.001 | 1 | 1 | 1 | 1 | 1 | 5000 | 4 | 0.05 |
| 1.006 | 1 | 1.006 | 1 | 1 | 1 | 10000 | 2 | 0.05 |
| 1.004 | 1 | 1.004 | 1 | 1 | 1 | 10000 | 4 | 0.05 |
| 1.015 | 1 | 1.011 | 1 | 1 | 1 | 5000 | 2 | 0.1 |
| 1 | 1 | 1.006 | 1 | 1 | 1 | 5000 | 4 | 0.1 |
| 1.012 | 1 | 1.01 | 1 | 1 | 1 | 10000 | 2 | 0.1 |
| 1.004 | 1 | 1.003 | 1 | 1 | 1 | 10000 | 4 | 0.1 |

**WEB TABLE 27-** Ratio of the empirical variance with SCC to the empirical variance with SCC. Poststrat and ratio of the empirical variance with USCC to the empirical variance with USCC.Poststrat, for the log-relative hazard parameters $\beta_{1}$, $\beta_{2}$ and $\beta_{3}$, from 5,000 simulated cohorts. The ratio is a measure of efficiency gain from using the poststratified weights

| $\boldsymbol{x=}\left( -1, 1, -0.6 \right)'$ | | $\boldsymbol{x=}\left( 1, -1, 0.6 \right)'$ | | $\boldsymbol{x=}\left( 1, 1, 0.6 \right)'$ | | $n$ | $K$ | $p_{Y}$ |
| --- | --- | --- | --- | --- | --- | --- | --- | --- |
| SCC / SCC.Poststrat | USCC / USCC.Poststrat | SCC / SCC.Poststrat | USCC / USCC.Poststrat | SCC / SCC.Poststrat | USCC / USCC.Poststrat |  |  |  |
| 1.005 | 1.005 | 1.006 | 1.001 | 1.003 | 1 | 5000 | 2 | 0.02 |
| 1.001 | 1.002 | 1.005 | 0.999 | 1.002 | 1 | 5000 | 4 | 0.02 |
| 1.002 | 1.003 | 1.006 | 1.001 | 1 | 1.001 | 10000 | 2 | 0.02 |
| 1.002 | 1.003 | 1.002 | 1.001 | 1.001 | 1 | 10000 | 4 | 0.02 |
| 1.009 | 1.005 | 1.007 | 1.002 | 1.008 | 1.002 | 5000 | 2 | 0.05 |
| 1.008 | 1.005 | 1 | 1.001 | 1.002 | 1.001 | 5000 | 4 | 0.05 |
| 1.005 | 1.005 | 1.008 | 1.001 | 1.007 | 1.003 | 10000 | 2 | 0.05 |
| 1.004 | 1 | 1.005 | 1.001 | 1.005 | 1.002 | 10000 | 4 | 0.05 |
| 1.018 | 1.011 | 1.012 | 1.003 | 1.011 | 1.006 | 5000 | 2 | 0.1 |
| 1.005 | 1.001 | 1.006 | 1 | 1.002 | 1.002 | 5000 | 4 | 0.1 |
| 1.019 | 1.007 | 1.01 | 1.001 | 1.006 | 1.001 | 10000 | 2 | 0.1 |
| 1.002 | 1.003 | 1.004 | 1 | 1.004 | 1.002 | 10000 | 4 | 0.1 |

**WEB TABLE 28-** Ratio of the empirical variance with SCC to the empirical variance with SCC. Poststrat and ratio of the empirical variance with USCC to the empirical variance with USCC.Poststrat, for pure risks $\pi\left( \tau_{1},\tau_{2}; \boldsymbol{x} \right)$, with ${(\tau}_{1},\tau_{2}]=(0,8]$ and $\boldsymbol{x}\in\left\{ \left( -1, 1, -0.6 \right)',\left( 1, -1, 0.6 \right)',\left( 1, 1, 0.6 \right)' \right\}$, from 5,000 simulated cohorts. The ratio is a measure of efficiency gain from using the poststratified weights

| SCC.Calib | | | | | $n$ | $K$ | $p_{Y}$ | $\beta_{1}$ |
| --- | --- | --- | --- | --- | --- | --- | --- | --- |
| Empirical variance | Mean of estimated variance | | Coverage of 95% CIs | |  |  |  |  |
|  | $\hat{V}_{\mathrm{Robust}}$ | $\hat{V}$ | $\hat{V}_{\mathrm{Robust}}$ | $\hat{V}$ |  |  |  |  |
| 0.0181 | 0.019 | 0.0181 | 0.9582* | 0.9532 | 5000 | 2 | 0.02 | -0.2 |
| 0.0159 | 0.0164 | 0.016 | 0.9548 | 0.9518 | 5000 | 4 | 0.02 | -0.2 |
| 0.0085 | 0.009 | 0.0086 | 0.9562* | 0.9512 | 10000 | 2 | 0.02 | -0.2 |
| 0.0076 | 0.0079 | 0.0077 | 0.9552 | 0.9512 | 10000 | 4 | 0.02 | -0.2 |
| 0.0069 | 0.0071 | 0.0068 | 0.9532 | 0.9482 | 5000 | 2 | 0.05 | -0.2 |
| 0.0061 | 0.0063 | 0.0061 | 0.9512 | 0.9492 | 5000 | 4 | 0.05 | -0.2 |
| 0.0033 | 0.0035 | 0.0033 | 0.9576* | 0.952 | 10000 | 2 | 0.05 | -0.2 |
| 0.0031 | 0.0031 | 0.003 | 0.9512 | 0.9496 | 10000 | 4 | 0.05 | -0.2 |
| 0.0033 | 0.0034 | 0.0033 | 0.9592* | 0.9544 | 5000 | 2 | 0.1 | -0.2 |
| 0.0031 | 0.0031 | 0.003 | 0.9476 | 0.947 | 5000 | 4 | 0.1 | -0.2 |
| 0.0016 | 0.0017 | 0.0016 | 0.9554 | 0.952 | 10000 | 2 | 0.1 | -0.2 |
| 0.0015 | 0.0015 | 0.0015 | 0.9518 | 0.9506 | 10000 | 4 | 0.1 | -0.2 |

**WEB TABLE 29-** Estimation results with SCC.Calib (with weaker proxy of $X_{1}$) for the log-relative hazard $\beta_{1}$*,* from using different variance estimation methods in 5,000 simulated cohorts. * indicates coverage outside the expected interval [0.9440; 0.9560]

| SCC.Calib | | | | | $n$ | $K$ | $p_{Y}$ | $\log\left\{ \pi\left( \tau_{1},\tau_{2};\boldsymbol{x} \right) \right\}$ |
| --- | --- | --- | --- | --- | --- | --- | --- | --- |
| Empirical variance | Mean of estimated variance | | Coverage of 95% CIs | |  |  |  |  |
|  | $\hat{V}_{\mathrm{Robust}}$ | $\hat{V}$ | $\hat{V}_{\mathrm{Robust}}$ | $\hat{V}$ |  |  |  |  |
| 0.0292 | 0.0307 | 0.0291 | 0.957* | 0.9516 | 5000 | 2 | 0.02 | -3.948 |
| 0.0269 | 0.0277 | 0.0269 | 0.9526 | 0.9492 | 5000 | 4 | 0.02 | -3.948 |
| 0.014 | 0.0147 | 0.014 | 0.9588* | 0.9546 | 10000 | 2 | 0.02 | -3.948 |
| 0.0128 | 0.0134 | 0.013 | 0.957* | 0.953 | 10000 | 4 | 0.02 | -3.948 |
| 0.0106 | 0.0113 | 0.0108 | 0.9618* | 0.9554 | 5000 | 2 | 0.05 | -3.046 |
| 0.0105 | 0.0104 | 0.0102 | 0.946 | 0.9446 | 5000 | 4 | 0.05 | -3.046 |
| 0.0053 | 0.0056 | 0.0053 | 0.9544 | 0.9488 | 10000 | 2 | 0.05 | -3.046 |
| 0.005 | 0.0051 | 0.005 | 0.9538 | 0.9506 | 10000 | 4 | 0.05 | -3.046 |
| 0.005 | 0.0053 | 0.0051 | 0.953 | 0.9492 | 5000 | 2 | 0.1 | -2.377 |
| 0.005 | 0.0049 | 0.0048 | 0.9486 | 0.947 | 5000 | 4 | 0.1 | -2.377 |
| 0.0025 | 0.0026 | 0.0025 | 0.957* | 0.9542 | 10000 | 2 | 0.1 | -2.377 |
| 0.0025 | 0.0024 | 0.0024 | 0.9476 | 0.9462 | 10000 | 4 | 0.1 | -2.377 |

**WEB TABLE 30-** Estimation results with SCC.Calib (with weaker proxy of $X_{1}$) for the pure risk with profile $\boldsymbol{x=}\left( -1, 1, -0.6 \right)'$*,* from using different variance estimation methods in 5,000 simulated cohorts. * indicates coverage outside the expected interval [0.9440; 0.9560]

# PHASE-TWO MISSING DATA WHEN THE PHASE-THREE DESIGN SAMPLING PROBABILITIES ARE UNKNOWN

## Derivation of the influence functions

We let $\boldsymbol{\Delta}_{i,j}\left( \tilde{\boldsymbol{\theta}} \right)$ denote the influence of subject $i$ in stratum $j$ on $\tilde{\boldsymbol{\theta}}$, $i\in\{1,\ldots,n^{(j)}\}$,$j\in\left\{ 1,\ldots,J \right\}$, $\tilde{\boldsymbol{\theta}}\in\left\{ \tilde{\boldsymbol{\gamma}},\tilde{\boldsymbol{\beta}}, {d\tilde{\Lambda}}_{0}\left( t \right), \tilde{\Lambda}_{0}\left( t \right), \tilde{\pi}\left( \tau_{1},\tau_{2};\boldsymbol{x} \right) \right\}$.

As $\sum_{j=1}^{J} \sum_{i=1}^{n^{(j)}} \left\{ {\xi_{i,j}\boldsymbol{B}}_{i,j}-\exp\left( {\tilde{\boldsymbol{\gamma}}}^{'}\boldsymbol{B}_{i,j} \right) \xi_{i,j} V_{i,j}\boldsymbol{B}_{i,j} \right\}=0$, then, following Graubard and Fears (2005), we know that

${\xi_{i,j}\boldsymbol{B}}_{i,j}-\exp\left( {\tilde{\boldsymbol{\gamma}}}^{'}\boldsymbol{B}_{i,j} \right) \xi_{i,j} V_{i,j}\boldsymbol{B}_{i,j}-$ $\left\{ \sum_{l=1}^{J} \sum_{k=1}^{n^{(l)}} \xi_{k,l} V_{k,l}\exp\left( {\tilde{\boldsymbol{\gamma}}}^{'}\boldsymbol{B}_{k,l} \right)\boldsymbol{B}_{k,l}\boldsymbol{B}_{k,l}' \right\}\boldsymbol{\Delta}_{i,j}\left( \tilde{\boldsymbol{\gamma}} \right)=0$,

and as a result

$\boldsymbol{\Delta}_{i,j}\left( \tilde{\boldsymbol{\gamma}} \right)=\left\{ \sum_{l=1}^{J} \sum_{k=1}^{n^{\left( l \right)}} \xi_{k,l} V_{k,l}\exp\left( {\tilde{\boldsymbol{\gamma}}}^{'}\boldsymbol{B}_{k,l} \right)\boldsymbol{B}_{k,l}\boldsymbol{B}_{k,l}' \right\}^{-1}\left\{ {\xi_{i,j}\boldsymbol{B}}_{i,j}-\xi_{i,j} V_{i,j}\exp\left( {\tilde{\boldsymbol{\gamma}}}^{'}\boldsymbol{B}_{i,j} \right)\boldsymbol{B}_{i,j} \right\}$.

We can then write

$\boldsymbol{\Delta}_{i,j}\left( \tilde{\boldsymbol{\gamma}} \right)=\xi_{i,j} \boldsymbol{IF}_{i,j}^{(2)}\left( \tilde{\boldsymbol{\gamma}} \right)+\xi_{i,j} V_{i,j}\exp\left( {\tilde{\boldsymbol{\gamma}}}^{'}\boldsymbol{B}_{i,j} \right)\boldsymbol{IF}_{i,j}^{(3)}\left( \tilde{\boldsymbol{\gamma}} \right)$,

with $\boldsymbol{IF}_{i,j}^{(2)}\left( \tilde{\boldsymbol{\gamma}} \right)=\left\{ \sum_{l=1}^{J} \sum_{k=1}^{n^{\left( l \right)}} \xi_{k,l} V_{k,l}\exp\left( {\tilde{\boldsymbol{\gamma}}}^{'}\boldsymbol{B}_{k,l} \right)\boldsymbol{B}_{k,l}\boldsymbol{B}_{k,l}' \right\}^{-1}\boldsymbol{B}_{i,j}$,

and $\boldsymbol{IF}_{i,j}^{(3)}\left( \tilde{\boldsymbol{\gamma}} \right)=-\left\{ \sum_{l=1}^{J} \sum_{k=1}^{n^{\left( l \right)}} \xi_{k,l} V_{k,l}\exp\left( {\tilde{\boldsymbol{\gamma}}}^{'}\boldsymbol{B}_{k,l} \right)\boldsymbol{B}_{k,l}\boldsymbol{B}_{k,l}' \right\}^{-1}\boldsymbol{B}_{i,j}$.

Then, we know that $\int_{t} \sum_{j=1}^{J} \sum_{i=1}^{n^{(j)}} V_{i,j}\exp\left( {\tilde{\boldsymbol{\gamma}}}^{'}\boldsymbol{B}_{i,j} \right)\left\{ X_{i,j}-\frac{{\tilde{\boldsymbol{S}}}_{1}\left( t;\tilde{\boldsymbol{\gamma}},\tilde{\boldsymbol{\beta}} \right)}{\tilde{S}_{0}\left( t;\tilde{\boldsymbol{\gamma}},\tilde{\boldsymbol{\beta}} \right)} \right\}{dN}_{i,j}\left( t \right)=0$. We can rewrite this estimating equation as $\int_{t} \sum_{j=1}^{J} \sum_{i=1}^{n^{(j)}} {\xi_{i,j} V}_{i,j} w_{i,j}^{\left( 2 \right)}\exp\left( {\tilde{\boldsymbol{\gamma}}}^{'}\boldsymbol{B}_{i,j} \right)\left\{ X_{i,j}-\frac{{\tilde{\boldsymbol{S}}}_{1}\left( t;\tilde{\boldsymbol{\gamma}},\tilde{\boldsymbol{\beta}} \right)}{\tilde{S}_{0}\left( t;\tilde{\boldsymbol{\gamma}},\tilde{\boldsymbol{\beta}} \right)} \right\}{dN}_{i,j}\left( t \right)=0$, because $\xi_{i,j} w_{i,j}^{\left( 2 \right)}=1$ for any subject $i$ in stratum $j$ such that $\int_{t} {dN}_{i,j}\left( t \right)=1$ (i.e., all the cases are included in the phase-two sample and have unit phase-two sampling weights). As a result, and following the similar arguments as for $\boldsymbol{\Delta}_{i,j}\left( \tilde{\boldsymbol{\gamma}} \right)$, we know

| $\boldsymbol{\Delta}_{i,j}\left\{ {\tilde{\boldsymbol{G}}}_{1}\left( \tilde{\boldsymbol{\gamma}} \right) \right\}+\int_{t} \left[ -\frac{\Delta_{i,j}\left\{ \sum_{l=1}^{J} \sum_{k=1}^{n^{(l)}} {{\xi_{k,l} V}_{k,l} w_{k,l}^{\left( 2 \right)}\exp({\tilde{\boldsymbol{\gamma}}}^{'}\boldsymbol{B}_{k,l})dN}_{k,l}\left( t \right) \right\}\times{\tilde{\boldsymbol{S}}}_{1}\left( t;\tilde{\boldsymbol{\gamma}},\tilde{\boldsymbol{\beta}} \right)}{\tilde{S}_{0}\left( t;\tilde{\boldsymbol{\gamma}},\tilde{\boldsymbol{\beta}} \right)}-\frac{\left\{ \sum_{l=1}^{J} \sum_{k=1}^{n^{(l)}} {{\xi_{k,l} V}_{k,l} w_{k,l}^{\left( 2 \right)}\exp({\tilde{\boldsymbol{\gamma}}}^{'}\boldsymbol{B}_{k,l})dN}_{k,l}\left( t \right) \right\}\times\boldsymbol{\Delta}_{i,j}\left\{ {\tilde{\boldsymbol{S}}}_{1}\left( t;\tilde{\boldsymbol{\gamma}},\tilde{\boldsymbol{\beta}} \right) \right\}}{\tilde{S}_{0}\left( t;\tilde{\boldsymbol{\gamma}},\tilde{\boldsymbol{\beta}} \right)}+\frac{\left\{ \sum_{l=1}^{J} \sum_{k=1}^{n^{(l)}} {{\xi_{k,l} V}_{k,l} w_{k,l}^{\left( 2 \right)}\exp({\tilde{\boldsymbol{\gamma}}}^{'}\boldsymbol{B}_{k,l})dN}_{k,l}\left( t \right) \right\}\times{{\tilde{\boldsymbol{S}}}_{1}\left( t;\tilde{\boldsymbol{\gamma}},\tilde{\boldsymbol{\beta}} \right)\times\Delta}_{i,j}\left\{ \tilde{S}_{0}\left( t;\tilde{\boldsymbol{\gamma}},\tilde{\boldsymbol{\beta}} \right) \right\}}{\left\{ \tilde{S}_{0}\left( t;\tilde{\boldsymbol{\gamma}},\tilde{\boldsymbol{\beta}} \right) \right\}^{2}} \right]=0$, | (3) |
| --- | --- |

With ${\tilde{\boldsymbol{G}}}_{1}\left( \tilde{\boldsymbol{\gamma}} \right)=\int_{t} \sum_{l=1}^{J} \sum_{k=1}^{n^{(l)}} {\xi_{k,l} V}_{k,l} w_{k,l}^{\left( 2 \right)}\exp({\tilde{\boldsymbol{\gamma}}}^{'}\boldsymbol{B}_{k,l})\boldsymbol{X}_{k,l} {dN}_{k,l}\left( t \right)$,

$\boldsymbol{\Delta}_{i,j}\left\{ {\tilde{\boldsymbol{G}}}_{1}\left( t,\tilde{\boldsymbol{\gamma}} \right) \right\}={\xi_{i,j} V}_{i,j} w_{i,j}^{\left( 2 \right)}\exp({\tilde{\boldsymbol{\gamma}}}^{'}\boldsymbol{B}_{i,j}){dN}_{i,j}\left( t \right) \boldsymbol{X}_{i,j}+\left\{ {\frac{\partial{\tilde{\boldsymbol{G}}}_{1}\left( t,\boldsymbol{\gamma} \right)}{\partial\boldsymbol{\gamma}}}_{|\boldsymbol{\gamma}=\tilde{\boldsymbol{\gamma}}} \right\}\boldsymbol{\Delta}_{i,j}\left( \tilde{\boldsymbol{\gamma}} \right)$,

$\Delta_{i,j}\left\{ \sum_{l=1}^{J} \sum_{k=1}^{n^{(l)}} {{\xi_{k,l} V}_{k,l} w_{k,l}^{\left( 2 \right)}\exp({\tilde{\boldsymbol{\gamma}}}^{'}\boldsymbol{B}_{k,l})dN}_{k,l}\left( t \right) \right\}={\xi_{i,j} V}_{i,j} w_{i,j}^{\left( 2 \right)}\exp({\tilde{\boldsymbol{\gamma}}}^{'}\boldsymbol{B}_{i,j}){dN}_{i,j}\left( t \right)+\left[ {\frac{\partial\left\{ \sum_{l=1}^{J} \sum_{k=1}^{n^{(l)}} {{\xi_{k,l} V}_{k,l} w_{k,l}^{\left( 2 \right)}\exp(\boldsymbol{\gamma}^{'}\boldsymbol{B}_{k,l})dN}_{k,l}\left( t \right) \right\}}{\partial\boldsymbol{\gamma}}}_{|\boldsymbol{\gamma}=\tilde{\boldsymbol{\gamma}}} \right]\boldsymbol{\Delta}_{i,j}\left( \tilde{\boldsymbol{\gamma}} \right)$,

${\frac{\partial{\tilde{\boldsymbol{G}}}_{1}\left( t,\boldsymbol{\gamma} \right)}{\partial\boldsymbol{\gamma}}}_{|\boldsymbol{\gamma}=\tilde{\boldsymbol{\gamma}}}=\sum_{l=1}^{J} \sum_{k=1}^{n^{(l)}} {\xi_{k,l} V}_{k,l}w_{k,l}^{\left( 2 \right)}\exp({\tilde{\boldsymbol{\gamma}}}^{'}\boldsymbol{B}_{k,l}){dN}_{k,l}\left( t \right) \boldsymbol{X}_{k,l}\boldsymbol{B}_{k,l}'$,

${\frac{\partial\left\{ \sum_{l=1}^{J} \sum_{k=1}^{n^{(j)}} {{\xi_{k,l} V}_{k,l} w_{k,l}^{\left( 2 \right)}\exp(\boldsymbol{\gamma}^{'}\boldsymbol{B}_{k,l})dN}_{k,l}\left( t \right) \right\}}{\partial\boldsymbol{\gamma}}}_{|\boldsymbol{\gamma}=\tilde{\boldsymbol{\gamma}}}=\sum_{l=1}^{J} \sum_{k=1}^{n^{(l)}} \xi_{k,l}V_{k,l}w_{k,l}^{\left( 2 \right)}\exp({\tilde{\boldsymbol{\gamma}}}^{'}\boldsymbol{B}_{k,l}){dN}_{k,l}\left( t \right) \boldsymbol{B}_{k,l}'$,

$\boldsymbol{\Delta}_{i,j}\left\{ {\tilde{\boldsymbol{S}}}_{1}\left( t;\tilde{\boldsymbol{\gamma}},\tilde{\boldsymbol{\beta}} \right) \right\}=\xi_{i,j}V_{i,j} w_{i,j}^{\left( 2 \right)}\exp\left( {\tilde{\boldsymbol{\gamma}}}^{'}\boldsymbol{B}_{i,j} \right)Y_{i,j}\left( t \right)\exp\left( {\tilde{\boldsymbol{\beta}}}^{'}\boldsymbol{X}_{i,j} \right)\boldsymbol{X}_{i,j}+\left\{ {\frac{\partial{\tilde{\boldsymbol{S}}}_{1}\left( t;\tilde{\boldsymbol{\gamma}},\boldsymbol{\beta} \right)}{\partial\boldsymbol{\beta}}}_{|\boldsymbol{\beta}=\tilde{\boldsymbol{\beta}}} \right\}\boldsymbol{\Delta}_{i,j}\left( \tilde{\boldsymbol{\beta}} \right)+\left\{ {\frac{\partial{\tilde{\boldsymbol{S}}}_{1}\left( t;\boldsymbol{\gamma},\tilde{\boldsymbol{\beta}} \right)}{\partial\boldsymbol{\gamma}}}_{|\boldsymbol{\gamma}=\tilde{\boldsymbol{\gamma}}} \right\}\boldsymbol{\Delta}_{i,j}\left( \tilde{\boldsymbol{\gamma}} \right)$,

${\frac{\partial{\tilde{\boldsymbol{S}}}_{1}\left( t;\tilde{\boldsymbol{\gamma}},\boldsymbol{\beta} \right)}{\partial\boldsymbol{\beta}}}_{|\boldsymbol{\beta}=\tilde{\boldsymbol{\beta}}}=\sum_{l=1}^{J} \sum_{k=1}^{n^{(l)}} \xi_{k,l}V_{k,l} w_{k,l}^{\left( 2 \right)}\exp({\tilde{\boldsymbol{\gamma}}}^{'}\boldsymbol{B}_{k,l})Y_{k,l}\left( t \right)\exp\left( {\tilde{\boldsymbol{\beta}}}^{'}\boldsymbol{X}_{k,l} \right)\boldsymbol{X}_{k,l} \boldsymbol{X}_{k,l}'\equiv{\tilde{\boldsymbol{S}}}_{2}\left( t;\tilde{\boldsymbol{\gamma}},\tilde{\boldsymbol{\beta}} \right)$,

${\frac{\partial{\tilde{\boldsymbol{S}}}_{1}\left( t;\boldsymbol{\gamma},\tilde{\boldsymbol{\beta}} \right)}{\partial\boldsymbol{\gamma}}}_{|\boldsymbol{\gamma}=\tilde{\boldsymbol{\gamma}}}=\sum_{l=1}^{J} \sum_{k=1}^{n^{(l)}} \xi_{k,l}V_{k,l} w_{k,l}^{\left( 2 \right)}\exp({\tilde{\boldsymbol{\gamma}}}^{'}\boldsymbol{B}_{k,l})Y_{k,l}\left( t \right)\exp\left( {\tilde{\boldsymbol{\beta}}}^{'}\boldsymbol{X}_{k,l} \right)\boldsymbol{X}_{k,l} \boldsymbol{B}_{k,l}'$,

$\Delta_{i,j}\left\{ \tilde{S}_{0}\left( t;\tilde{\boldsymbol{\gamma}},\tilde{\boldsymbol{\beta}} \right) \right\}=\xi_{i,j}V_{i,j}\exp({\tilde{\boldsymbol{\gamma}}}^{'}\boldsymbol{B}_{i,j})w_{i,j}^{\left( 2 \right)}Y_{i,j}\left( t \right)\exp\left( {\tilde{\boldsymbol{\beta}}}^{'}\boldsymbol{X}_{i,j} \right)+\left\{ {\frac{\partial\tilde{S}_{0}\left( t;\tilde{\boldsymbol{\gamma}},\boldsymbol{\beta} \right)}{\partial\boldsymbol{\beta}}}_{|\boldsymbol{\beta}=\tilde{\boldsymbol{\beta}}} \right\}\boldsymbol{\Delta}_{i,j}\left( \tilde{\boldsymbol{\beta}} \right)+\left\{ {\frac{\partial\tilde{S}_{0}\left( t;\boldsymbol{\gamma},\tilde{\boldsymbol{\beta}} \right)}{\partial\boldsymbol{\gamma}}}_{|\boldsymbol{\gamma}=\tilde{\boldsymbol{\gamma}}} \right\}\boldsymbol{\Delta}_{i,j}\left( \tilde{\boldsymbol{\gamma}} \right)$,

${\frac{\partial\tilde{S}_{0}\left( t;\tilde{\boldsymbol{\gamma}},\boldsymbol{\beta} \right)}{\partial\boldsymbol{\beta}}}_{|\boldsymbol{\beta}=\tilde{\boldsymbol{\beta}}}=\sum_{l=1}^{J} \sum_{k=1}^{n^{(l)}} \xi_{k,l}V_{k,l}\exp({\tilde{\boldsymbol{\gamma}}}^{'}\boldsymbol{B}_{k,l})w_{k,l}^{\left( 2 \right)} Y_{k,l}\left( t \right)\exp\left( {\tilde{\boldsymbol{\beta}}}^{'}\boldsymbol{X}_{k,l} \right)\boldsymbol{X}_{k,l}'={\tilde{\boldsymbol{S}}}_{1}\left( t;\tilde{\boldsymbol{\gamma}},\tilde{\boldsymbol{\beta}} \right)'$,

and ${\frac{\partial\tilde{S}_{0}\left( t;\boldsymbol{\gamma},\tilde{\boldsymbol{\beta}} \right)}{\partial\boldsymbol{\gamma}}}_{|\boldsymbol{\gamma}=\tilde{\boldsymbol{\gamma}}}=\sum_{l=1}^{J} \sum_{k=1}^{n^{(l)}} \xi_{k,l}V_{k,l}\exp({\tilde{\boldsymbol{\gamma}}}^{'}\boldsymbol{B}_{k,l})w_{k,l}^{\left( 2 \right)} Y_{k,l}\left( t \right)\exp\left( {\tilde{\boldsymbol{\beta}}}^{'}\boldsymbol{X}_{k,l} \right)\boldsymbol{B}_{k,l}'$.

Note, $\sum_{l=1}^{J} \sum_{k=1}^{n^{(l)}} {{\xi_{k,l} V}_{k,l} w_{k,l}^{\left( 2 \right)}\exp({\tilde{\boldsymbol{\gamma}}}^{'}\boldsymbol{B}_{k,l})dN}_{k,l}\left( t \right)$, ${\frac{\partial\left\{ \sum_{l=1}^{J} \sum_{k=1}^{n^{(j)}} {{\xi_{k,l} V}_{k,l} w_{k,l}^{\left( 2 \right)}\exp(\boldsymbol{\gamma}^{'}\boldsymbol{B}_{k,l})dN}_{k,l}\left( t \right) \right\}}{\partial\boldsymbol{\gamma}}}_{|\boldsymbol{\gamma}=\tilde{\boldsymbol{\gamma}}}$ and thus $\Delta_{i,j}\left\{ \sum_{l=1}^{J} \sum_{k=1}^{n^{(j)}} {{\xi_{k,l} V}_{k,l} w_{k,l}^{\left( 2 \right)}\exp({\tilde{\boldsymbol{\gamma}}}^{'}\boldsymbol{B}_{k,l})dN}_{k,l}\left( t \right) \right\}$ and the term between brackets in Equation (3) are linear combinations of ${dN}_{i,j}\left( t \right)$. Hence

$\boldsymbol{\Delta}_{i,j}\left( \tilde{\boldsymbol{\beta}} \right)=\left[ \sum_{l=1}^{J} \sum_{k=1}^{n^{(l)}} \int_{t} {\xi_{k,l} V}_{k,l} w_{k,l}^{\left( 2 \right)}\exp({\tilde{\boldsymbol{\gamma}}}^{'}\boldsymbol{B}_{k,l})\left\{ \frac{{\tilde{\boldsymbol{S}}}_{2}\left( t;\tilde{\boldsymbol{\gamma}},\tilde{\boldsymbol{\beta}} \right)}{\tilde{S}_{0}\left( t;\tilde{\boldsymbol{\gamma}},\tilde{\boldsymbol{\beta}} \right)}-\frac{{\tilde{\boldsymbol{S}}}_{1}\left( t;\tilde{\boldsymbol{\gamma}},\tilde{\boldsymbol{\beta}} \right){{\tilde{\boldsymbol{S}}}_{1}\left( t;\tilde{\boldsymbol{\gamma}},\tilde{\boldsymbol{\beta}} \right)}^{'}}{{\tilde{S}_{0}\left( t;\tilde{\boldsymbol{\gamma}},\tilde{\boldsymbol{\beta}} \right)}^{2}} \right\}{dN}_{k,l}\left( t \right) \right]^{-1}\times\left[ \int_{t} \left\{ \boldsymbol{X}_{i,j}-\frac{{\tilde{\boldsymbol{S}}}_{1}\left( t;\tilde{\boldsymbol{\gamma}},\tilde{\boldsymbol{\beta}} \right)}{\tilde{S}_{0}\left( t;\tilde{\boldsymbol{\gamma}},\tilde{\boldsymbol{\beta}} \right)} \right\}\times\left\{ {\xi_{i,j} V}_{i,j} w_{i,j}^{\left( 2 \right)}\exp({\tilde{\boldsymbol{\gamma}}}^{'}\boldsymbol{B}_{i,j}){dN}_{i,j}\left( t \right)-\xi_{i,j}V_{i,j}w_{i,j}^{\left( 2 \right)}\exp({\tilde{\boldsymbol{\gamma}}}^{'}\boldsymbol{B}_{i,j})Y_{i,j}\left( t \right)\exp\left( {\tilde{\boldsymbol{\beta}}}^{'}\boldsymbol{X}_{i,j} \right)\times\frac{\sum_{l=1}^{J} \sum_{k=1}^{n^{(j)}} {{\xi_{k,l} V}_{k,l} w_{k,l}^{\left( 2 \right)}\exp({\tilde{\boldsymbol{\gamma}}}^{'}\boldsymbol{B}_{k,l})dN}_{k,l}\left( t \right)}{\tilde{S}_{0}\left( t;\tilde{\gamma},\tilde{\beta} \right)} \right\} \right]+\left[ \sum_{l=1}^{J} \sum_{k=1}^{n^{(l)}} \int_{t} {\xi_{k,l} V}_{k,l} w_{k,l}^{\left( 2 \right)}\exp({\tilde{\boldsymbol{\gamma}}}^{'}\boldsymbol{B}_{k,l})\left\{ \frac{{\tilde{\boldsymbol{S}}}_{2}\left( t;\tilde{\boldsymbol{\gamma}},\tilde{\boldsymbol{\beta}} \right)}{\tilde{S}_{0}\left( t;\tilde{\boldsymbol{\gamma}},\tilde{\boldsymbol{\beta}} \right)}-\frac{{\tilde{\boldsymbol{S}}}_{1}\left( t;\tilde{\boldsymbol{\gamma}},\tilde{\boldsymbol{\beta}} \right){{\tilde{\boldsymbol{S}}}_{1}\left( t;\tilde{\boldsymbol{\gamma}},\tilde{\boldsymbol{\beta}} \right)}^{'}}{{\tilde{S}_{0}\left( t;\tilde{\boldsymbol{\gamma}},\tilde{\boldsymbol{\beta}} \right)}^{2}} \right\}{dN}_{k,l}\left( t \right) \right]^{-1}\times\left( {\frac{\partial{\tilde{\boldsymbol{G}}}_{1}\left( t,\boldsymbol{\gamma} \right)}{\partial\boldsymbol{\gamma}}}_{|\boldsymbol{\gamma}=\tilde{\boldsymbol{\gamma}}}-\int_{t} \frac{{\tilde{\boldsymbol{S}}}_{1}\left( t;\tilde{\boldsymbol{\gamma}},\tilde{\boldsymbol{\beta}} \right)}{\tilde{S}_{0}\left( t;\tilde{\boldsymbol{\gamma}},\tilde{\boldsymbol{\beta}} \right)}\times\left[ {\frac{\partial\left\{ \sum_{l=1}^{J} \sum_{k=1}^{n^{(j)}} {{\xi_{k,l} V}_{k,l} w_{k,l}^{\left( 2 \right)}\exp(\boldsymbol{\gamma}^{'}\boldsymbol{B}_{k,l})dN}_{k,l}\left( t \right) \right\}}{\partial\boldsymbol{\gamma}}}_{|\boldsymbol{\gamma}=\tilde{\boldsymbol{\gamma}}} \right]-\int_{t} \frac{\sum_{l=1}^{J} \sum_{k=1}^{n^{(j)}} {{\xi_{k,l} V}_{k,l} w_{k,l}^{\left( 2 \right)}\exp(\boldsymbol{\gamma}^{'}\boldsymbol{B}_{k,l})dN}_{k,l}\left( t \right)}{\tilde{S}_{0}\left( t;\tilde{\boldsymbol{\gamma}},\tilde{\boldsymbol{\beta}} \right)}\times\left\{ {\frac{\partial{\tilde{\boldsymbol{S}}}_{1}\left( t;\tilde{\boldsymbol{\gamma}},\boldsymbol{\beta} \right)}{\partial\boldsymbol{\gamma}}}_{|\boldsymbol{\gamma}=\tilde{\boldsymbol{\gamma}}} \right\}+\int_{t} \frac{\left\{ \sum_{l=1}^{J} \sum_{k=1}^{n^{(j)}} {{\xi_{k,l} V}_{k,l} w_{k,l}^{\left( 2 \right)}\exp(\boldsymbol{\gamma}^{'}\boldsymbol{B}_{k,l})dN}_{k,l}\left( t \right) \right\} {\tilde{\boldsymbol{S}}}_{1}\left( t;\tilde{\boldsymbol{\gamma}},\tilde{\boldsymbol{\beta}} \right)}{{\tilde{S}_{0}\left( t;\tilde{\boldsymbol{\gamma}},\tilde{\boldsymbol{\beta}} \right)}^{2}}\times\left\{ {\frac{\partial\tilde{S}_{0}\left( t;\boldsymbol{\gamma},\tilde{\boldsymbol{\beta}} \right)}{\partial\boldsymbol{\gamma}}}_{|\boldsymbol{\gamma}=\tilde{\boldsymbol{\gamma}}} \right\} \right)\boldsymbol{\Delta}_{i,j}\left( \tilde{\boldsymbol{\gamma}} \right)$,

and we can write

$\boldsymbol{\Delta}_{i,j}\left( \tilde{\boldsymbol{\beta}} \right)=\xi_{i,j}\boldsymbol{IF}_{i,j}^{(2)}\left( \tilde{\boldsymbol{\beta}} \right)+\xi_{i,j} V_{i,j}\exp\left( {\tilde{\boldsymbol{\gamma}}}^{'}\boldsymbol{B}_{i,j} \right)\boldsymbol{IF}_{i,j}^{(3)}\left( \tilde{\boldsymbol{\beta}} \right)$,

with $\boldsymbol{IF}_{i,j}^{(2)}\left( \tilde{\boldsymbol{\beta}} \right)=\left\{ \sum_{l=1}^{J} \sum_{k=1}^{n^{\left( j \right)}} \xi_{k,l} V_{k,l}\exp\left( {\tilde{\boldsymbol{\gamma}}}^{'}\boldsymbol{B}_{k,l} \right){\tilde{\boldsymbol{Z}}}_{k,l}\boldsymbol{B}_{k,l}' \right\}\times\boldsymbol{IF}_{i,j}^{(2)}\left( \tilde{\boldsymbol{\gamma}} \right)$,

and $\boldsymbol{IF}_{i,j}^{(3)}\left( \tilde{\boldsymbol{\beta}} \right)={\tilde{\boldsymbol{Z}}}_{i,j}+\left\{ \sum_{l=1}^{J} \sum_{k=1}^{n^{\left( j \right)}} \xi_{k,l} V_{k,l}\exp\left( {\tilde{\boldsymbol{\gamma}}}^{'}\boldsymbol{B}_{k,l} \right){\tilde{\boldsymbol{Z}}}_{k,l}\boldsymbol{B}_{k,l}' \right\}\times\boldsymbol{IF}_{i,j}^{(3)}\left( \tilde{\boldsymbol{\gamma}} \right)$,

and where

${\tilde{\boldsymbol{Z}}}_{i,j}=w_{i,j}^{\left( 2 \right)}\times\left[ \sum_{l=1}^{J} \sum_{k=1}^{n^{(l)}} \int_{t} {\xi_{k,l} V}_{k,l} w_{k,l}^{\left( 2 \right)}\exp({\tilde{\boldsymbol{\gamma}}}^{'}\boldsymbol{B}_{k,l})\left\{ \frac{{\tilde{\boldsymbol{S}}}_{2}\left( t;\tilde{\boldsymbol{\gamma}},\tilde{\boldsymbol{\beta}} \right)}{\tilde{S}_{0}\left( t;\tilde{\boldsymbol{\gamma}},\tilde{\boldsymbol{\beta}} \right)}-\frac{{\tilde{\boldsymbol{S}}}_{1}\left( t;\tilde{\boldsymbol{\gamma}},\tilde{\boldsymbol{\beta}} \right){{\tilde{\boldsymbol{S}}}_{1}\left( t;\tilde{\boldsymbol{\gamma}},\tilde{\boldsymbol{\beta}} \right)}^{'}}{{\tilde{S}_{0}\left( t;\tilde{\boldsymbol{\gamma}},\tilde{\boldsymbol{\beta}} \right)}^{2}} \right\}{dN}_{k,l}\left( t \right) \right]^{-1}\times\left[ \int_{t} \left\{ \boldsymbol{X}_{i,j}-\frac{{\tilde{\boldsymbol{S}}}_{1}\left( t;\tilde{\boldsymbol{\gamma}},\tilde{\boldsymbol{\beta}} \right)}{\tilde{S}_{0}\left( t;\tilde{\boldsymbol{\gamma}},\tilde{\boldsymbol{\beta}} \right)} \right\}\times\left\{ {dN}_{i,j}\left( t \right)-Y_{i,j}\left( t \right)\exp\left( {\tilde{\boldsymbol{\beta}}}^{'}\boldsymbol{X}_{i,j} \right)\times\frac{\sum_{l=1}^{J} \sum_{k=1}^{n^{(j)}} {{\xi_{k,l} V}_{k,l} w_{k,l}^{\left( 2 \right)}\exp({\tilde{\boldsymbol{\gamma}}}^{'}\boldsymbol{B}_{k,l})dN}_{k,l}\left( t \right)}{\tilde{S}_{0}\left( t;\tilde{\boldsymbol{\gamma}},\tilde{\boldsymbol{\beta}} \right)} \right\} \right]$.

Then we know $\sum_{j=1}^{J} \sum_{i=1}^{n^{(j)}} \left\{ {dN}_{i,j}\left( t \right)-{d\tilde{\Lambda}}_{0}\left( t \right)\xi_{i,j}V_{i,j} w_{i,j}^{\left( 2 \right)}\exp({\tilde{\boldsymbol{\gamma}}}^{'}\boldsymbol{B}_{i,j})Y_{i,j}\left( t \right)\exp\left( {\tilde{\boldsymbol{\beta}}}^{'}\boldsymbol{X}_{i,j} \right) \right\}=0$, as we estimate the baseline hazard non-parametrically by ${d\tilde{\Lambda}}_{0}\left( t \right)=\frac{\sum_{j=1}^{J} \sum_{i=1}^{n^{(j)}} {dN}_{i,j}\left( t \right)}{\tilde{S}_{0}\left( t;\tilde{\boldsymbol{\gamma}},\tilde{\boldsymbol{\beta}} \right)}$, and thus

${dN}_{i,j}\left( t \right)-{{d\tilde{\Lambda}}_{0}\left( t \right) \xi}_{i,j}V_{i,j} w_{i,j}^{\left( 2 \right)}\exp({\tilde{\boldsymbol{\gamma}}}^{'}\boldsymbol{B}_{i,j})Y_{i,j}\left( t \right)\exp\left( {\tilde{\boldsymbol{\beta}}}^{'}\boldsymbol{X}_{i,j} \right)-{d\tilde{\Lambda}}_{0}\left( t \right) {\tilde{\boldsymbol{S}}}_{1}\left( t;\tilde{\boldsymbol{\gamma}},\tilde{\boldsymbol{\beta}} \right)'\boldsymbol{\Delta}_{i,j}\left( \tilde{\boldsymbol{\beta}} \right)$ $-{d\tilde{\Lambda}}_{0}\left( t \right)\left\{ \sum_{l=1}^{J} \sum_{k=1}^{n^{(j)}} \xi_{k,l}V_{k,l}w_{k,l}^{\left( 2 \right)}\exp({\tilde{\boldsymbol{\gamma}}}^{'}\boldsymbol{B}_{k,l})Y_{k,l}\left( t \right)\exp\left( \tilde{\boldsymbol{\beta}}'\boldsymbol{X}_{k,l} \right)\boldsymbol{B}_{k,l} \right\}^{'}\boldsymbol{\Delta}_{i,j}\left( \tilde{\boldsymbol{\gamma}} \right)$ $-\tilde{S}_{0}\left( t;\tilde{\boldsymbol{\gamma}},\tilde{\boldsymbol{\beta}} \right) \Delta_{i,j}\left\{ {d\tilde{\Lambda}}_{0}\left( t \right) \right\}=0$.

As a result,

$\Delta_{i,j}\left\{ {d\tilde{\Lambda}}_{0}\left( t \right) \right\}=\left\{ \tilde{S}_{0}\left( t;\tilde{\boldsymbol{\gamma}},\tilde{\boldsymbol{\beta}} \right) \right\}^{-1}\left[ {dN}_{i,j}\left( t \right)-{d\tilde{\Lambda}}_{0}\left( t \right)\xi_{i,j}V_{i,j} w_{i,j}^{\left( 2 \right)}\exp\left( {\tilde{\boldsymbol{\gamma}}}^{'}\boldsymbol{B}_{i,j} \right)Y_{i,j}\left( t \right)\exp\left( {\tilde{\boldsymbol{\beta}}}^{'}\boldsymbol{X}_{i,j} \right)-{d\tilde{\Lambda}}_{0}\left( t \right) {\tilde{\boldsymbol{S}}}_{1}\left( t;\tilde{\boldsymbol{\gamma}},\tilde{\boldsymbol{\beta}} \right)^{'}\boldsymbol{\Delta}_{i,j}\left( \tilde{\boldsymbol{\beta}} \right)-{d\tilde{\Lambda}}_{0}\left( t \right)\left\{ \sum_{l=1}^{J} \sum_{k=1}^{n^{(j)}} \xi_{k,l}V_{k,l} w_{k,l}^{\left( 2 \right)}\exp({\tilde{\boldsymbol{\gamma}}}^{'}\boldsymbol{B}_{k,l})Y_{k,l}\left( t \right)\exp\left( \tilde{\boldsymbol{\beta}}'\boldsymbol{X}_{k,l} \right)\boldsymbol{B}_{k,l} \right\}^{'}\boldsymbol{\Delta}_{i,j}\left( \tilde{\boldsymbol{\gamma}} \right) \right]$,

and we can write $\Delta_{i,j}\left\{ {d\tilde{\Lambda}}_{0}\left( t \right) \right\}=\xi_{i,j} {IF}_{i,j}^{(2)}\left\{ {d\tilde{\Lambda}}_{0}\left( t \right) \right\}+\xi_{i,j} V_{i,j}\exp\left( {\tilde{\boldsymbol{\gamma}}}^{'}\boldsymbol{B}_{i,j} \right){IF}_{i,j}^{(3)}\left\{ {d\tilde{\Lambda}}_{0}\left( t \right) \right\}$, with

${IF}_{i,j}^{\left( 2 \right)}\left\{ {d\tilde{\Lambda}}_{0}\left( t \right) \right\}=\left\{ \tilde{S}_{0}\left( t;\tilde{\boldsymbol{\gamma}},\tilde{\boldsymbol{\beta}} \right) \right\}^{-1}{dN}_{i,j}\left( t \right)+\left\{ \sum_{l=1}^{J} \sum_{k=1}^{n^{\left( j \right)}} \xi_{k,l} V_{k,l}\exp\left( {\tilde{\boldsymbol{\gamma}}}^{'}\boldsymbol{B}_{k,l} \right)\tilde{H}_{k,l}\left( t \right)\boldsymbol{B}_{k,l}' \right\}\times\boldsymbol{IF}_{i,j}^{(2)}\left( \tilde{\boldsymbol{\gamma}} \right)$,

and ${IF}_{i,j}^{(3)}\left\{ {d\tilde{\Lambda}}_{0}\left( t \right) \right\}=\tilde{H}_{i,j}\left( t \right)+\left\{ \sum_{l=1}^{J} \sum_{k=1}^{n^{\left( j \right)}} \xi_{k,l} V_{k,l}\exp\left( {\tilde{\boldsymbol{\gamma}}}^{'}\boldsymbol{B}_{k,l} \right)\tilde{H}_{k,l}\left( t \right)\boldsymbol{B}_{k,l}' \right\}\times\boldsymbol{IF}_{i,j}^{(3)}\left( \tilde{\boldsymbol{\gamma}} \right)$,

and where $\tilde{H}_{i,j}\left( t \right)=-{\tilde{S}_{0}\left( t;\tilde{\boldsymbol{\gamma}},\tilde{\boldsymbol{\beta}} \right)}^{-1}{d\tilde{\Lambda}}_{0}\left( t \right)\left\{ {\tilde{\boldsymbol{S}}}_{1}\left( t;\tilde{\boldsymbol{\gamma}},\tilde{\boldsymbol{\beta}} \right)^{'}{\tilde{\boldsymbol{Z}}}_{i,j}+ \tilde{K}_{i,j}(t) \right\}$,

and $\tilde{K}_{i,j}\left( t \right)=w_{i,j}^{\left( 2 \right)} Y_{i,j}\left( t \right)\exp\left( \tilde{\boldsymbol{\beta}}'\boldsymbol{X}_{i,j} \right)$.

Note, $\tilde{H}_{i,j}\left( t \right)$, and thus ${IF}_{i,j}^{\left( 2 \right)}\left\{ {d\tilde{\Lambda}}_{0}\left( t \right) \right\}$ and ${IF}_{i,j}^{(3)}\left\{ {d\tilde{\Lambda}}_{0}\left( t \right) \right\}$, are linear combinations of the increments ${dN}_{i,j}\left( t \right)$ and ${d\tilde{\Lambda}}_{0}\left( t \right)$. Thus $\Delta_{i,j}\left\{ \int_{\tau_{1}}^{\tau_{2}} {d\tilde{\Lambda}}_{0}\left( t \right) \right\}=\int_{\tau_{1}}^{\tau_{2}} \Delta_{i,j}\left\{ {d\tilde{\Lambda}}_{0}\left( t \right) \right\}$, that we can write

$\xi_{i,j} {IF}_{i,j}^{(2)}\left\{ \int_{\tau_{1}}^{\tau_{2}} {d\tilde{\Lambda}}_{0}\left( t \right) \right\}+\xi_{i,j} V_{i,j}\exp\left( {\tilde{\boldsymbol{\gamma}}}^{'}\boldsymbol{B}_{i,j} \right) {IF}_{i,j}^{(3)}\left\{ \int_{\tau_{1}}^{\tau_{2}} {d\tilde{\Lambda}}_{0}\left( t \right) \right\}$,

with ${IF}_{i,j}^{(2)}\left\{ \int_{\tau_{1}}^{\tau_{2}} {d\tilde{\Lambda}}_{0}\left( t \right) \right\}=\sum_{t=0}^{\tau_{2}} {IF}_{i,j}^{(2)}\left\{ {d\tilde{\Lambda}}_{0}\left( t \right) \right\}$,

and ${IF}_{i,j}^{(2,3)}\left\{ \int_{\tau_{1}}^{\tau_{2}} {d\tilde{\Lambda}}_{0}\left( t \right) \right\}=\sum_{t=0}^{\tau_{2}} {IF}_{i,j}^{(3)}\left\{ {d\tilde{\Lambda}}_{0}\left( t \right) \right\}$, and we finally have

$\Delta_{i,j}\left\{ \tilde{\pi}\left( \tau_{1},\tau_{2};\boldsymbol{x} \right) \right\}=\left\{ {\frac{\partial\tilde{\pi}\left( \tau_{1},\tau_{2}; \boldsymbol{x} \right)}{\partial\boldsymbol{\beta}}}_{|\boldsymbol{\beta}=\tilde{\boldsymbol{\beta}}} \right\}\boldsymbol{\Delta}_{i,j}\left( \tilde{\boldsymbol{\beta}} \right)+\left[ {\frac{\partial\tilde{\pi}\left( \tau_{1},\tau_{2}; \boldsymbol{x} \right)}{\partial\left\{ \int_{\tau_{1}}^{\tau_{2}} {d\Lambda}_{0}\left( t \right) \right\}}}_{|{d\Lambda}_{0}(t)={d\tilde{\Lambda}}_{0}\left( t \right)} \right]\Delta_{i,j}\left\{ \int_{\tau_{1}}^{\tau_{2}} {d\tilde{\Lambda}}_{0}\left( t \right) \right\}$,

that we can write $\xi_{i,j} {IF}_{i,j}^{(2)}\left\{ \tilde{\pi}\left( \tau_{1},\tau_{2};\boldsymbol{x} \right) \right\}+\xi_{i,j} V_{i,j}\exp\left( {\tilde{\boldsymbol{\gamma}}}^{'}\boldsymbol{B}_{i,j} \right){IF}_{i,j}^{(3)}\left\{ \tilde{\pi}\left( \tau_{1},\tau_{2};\boldsymbol{x} \right) \right\}$,

with ${\frac{\partial\tilde{\pi}\left( \tau_{1},\tau_{2}; \boldsymbol{x} \right)}{\partial\boldsymbol{\beta}}}_{|\boldsymbol{\beta}=\tilde{\boldsymbol{\beta}}}=\left\{ \int_{\tau_{1}}^{\tau_{2}} {d\tilde{\Lambda}}_{0}\left( t \right)\exp\left( \tilde{\boldsymbol{\beta}}'\boldsymbol{x} \right) \right\}\left\{ 1-\tilde{\pi}\left( \tau_{1},\tau_{2}; \boldsymbol{x} \right) \right\}\boldsymbol{x'}$,

and ${\frac{\partial\tilde{\pi}\left( \tau_{1},\tau_{2}; \boldsymbol{x} \right)}{\partial\left\{ \int_{\tau_{1}}^{\tau_{2}} {d\Lambda}_{0}\left( t \right) \right\}}}_{|{d\Lambda}_{0}(t)={d\tilde{\Lambda}}_{0}\left( t \right)}=\exp\left( \tilde{\boldsymbol{\beta}}'\boldsymbol{x} \right)\left\{ 1-\tilde{\pi}\left( \tau_{1},\tau_{2}; \boldsymbol{x} \right) \right\}$.

Observe that for any $\tilde{\boldsymbol{\theta}}\in\left\{ \tilde{\boldsymbol{\gamma}},\tilde{\boldsymbol{\beta}}, {d\tilde{\Lambda}}_{0}\left( t \right), \tilde{\Lambda}_{0}\left( t \right), \tilde{\pi}\left( \tau_{1},\tau_{2};\boldsymbol{x} \right) \right\}$*,* if subject $i$ in stratum $j$ is in the phase-two sample but not in the phase-three sample, then $\xi_{i,j} V_{i,j}\exp\left( {\tilde{\boldsymbol{\gamma}}}^{'}\boldsymbol{B}_{i,j} \right)\boldsymbol{IF}_{i,j}^{(3)}\left( \tilde{\boldsymbol{\theta}} \right)$ is zero. However, $\xi_{i,j}\boldsymbol{IF}_{i,j}^{(2)}\left( \tilde{\boldsymbol{\theta}} \right)$ is usually non-zero. In particular, such individual affects $\tilde{\boldsymbol{\theta}}$ through his/her influence on $\tilde{\boldsymbol{\gamma}}$, as he/she is used for the estimation of the phase-three sampling weights.

## Variance decomposition and estimation

As mentioned in Section 5.5. in the Main Document, for any $\tilde{\boldsymbol{\theta}}\in\left\{ \tilde{\boldsymbol{\gamma}},\tilde{\boldsymbol{\beta}}, {d\tilde{\Lambda}}_{0}\left( t \right), \tilde{\Lambda}_{0}\left( t \right), \tilde{\pi}\left( \tau_{1},\tau_{2};\boldsymbol{x} \right) \right\}$, using the law of total covariance and the law of total expectation, we can decompose $\mathrm{var}\left\{ \sum_{j=1}^{J} \sum_{i=1}^{n^{(j)}} \boldsymbol{\Delta}_{i,j}\left( \tilde{\boldsymbol{\theta}} \right) \right\}$ as

$\mathrm{var}\left( E\left[ E\left\{ \sum_{j=1}^{J} \sum_{i=1}^{n^{\left( j \right)}} \boldsymbol{\Delta}_{i,j}\left( \tilde{\boldsymbol{\theta}} \right)|C_{1},C_{2} \right\}|C_{1} \right] \right)+ E\left( \mathrm{var}\left[ E\left\{ \sum_{j=1}^{J} \sum_{i=1}^{n^{\left( j \right)}} \boldsymbol{\Delta}_{i,j}\left( \tilde{\boldsymbol{\theta}} \right)|C_{1},C_{2} \right\}|C_{1} \right] \right)+ E\left( E\left[ \mathrm{var}\left\{ \sum_{j=1}^{J} \sum_{i=1}^{n^{\left( j \right)}} \boldsymbol{\Delta}_{i,j}\left( \tilde{\boldsymbol{\theta}} \right)|C_{1},C_{2} \right\}|C_{1} \right] \right)$,

where $C_{1}$ denote the information from the whole cohort, and $C_{2}$ denote the information from the phase-two sample.

For any $j,l\in\left\{ 1,\ldots,J \right\}$, we know that $E\left( \xi_{i,j} | C_{1} \right)=\frac{1}{w_{i,j}^{\left( 2 \right)}}$, $E\left( \xi_{i,j}\xi_{k,j} | C_{1} \right)=\frac{1}{w_{i,k,j}^{\left( 2 \right)}}=\frac{m^{\left( j \right)}(m^{\left( j \right)}-1)}{n^{\left( j \right)}(n^{\left( j \right)}-1)}$ if individuals $i$ and $k$, $i\neq k$, are both non-cases in stratum $j$, and $E\left( \xi_{i,j}\xi_{k,j} | C_{1} \right)=\frac{1}{w_{i,j}^{\left( 2 \right)}}\times\frac{1}{w_{k,j}^{\left( 2 \right)}}$ if $i$ and/or $k$, $i\neq k$, is a case in stratum $j$*.* In addition, $E\left( \xi_{i,j}\xi_{k,l} | C_{1} \right)=\frac{1}{w_{i,j}^{\left( 2 \right)}}\times\frac{1}{w_{k,l}^{\left( 2 \right)}}$ if individuals $i$ and $k$ are in stratum $j$ and stratum $l$, respectively, with $j\neq l$. Thus, $\mathrm{cov}\left( \xi_{i,j}{,\xi}_{k,j} | C_{1} \right)=\sigma_{i,k,j}^{(2)}=\frac{1}{w_{i,k,j}^{\left( 2 \right)}}-\frac{1}{w_{i,j}^{\left( 2 \right)}}\times\frac{1}{w_{k,j}^{\left( 2 \right)}}$ if both $i$ and $k$, $i\neq k$, are non-cases in stratum $j$, $\mathrm{cov}\left( \xi_{i,j}{,\xi}_{k,j} | C_{1} \right)=0$ if $i$ and/or $k$ is a case in stratum $j$, $\mathrm{cov}\left( \xi_{i,j}{,\xi}_{k,l} | C_{1} \right)=0$ if $i$ and $k$ are in stratum $j$ and stratum $l$, respectively, with $j\neq l$, $\mathrm{var}\left( \xi_{i,j} | C_{1} \right)=\sigma_{i,i,j}^{(2)}\equiv\sigma_{i,j}^{(2)}=\frac{1}{w_{i,j}^{\left( 2 \right)}}\left( 1-\frac{1}{w_{i,j}^{\left( 2 \right)}} \right)$ if $i$ is a non-case in stratum $j$*,* and $\mathrm{var}\left( \xi_{i,j} | C_{1} \right)=0$ if $i$ is a case in stratum $j$*.* In addition, because the third phase of sampling is Bernoulli, $E\left( V_{i,j} | C_{1},C_{2} \right)=\frac{1}{w_{i,j}^{\left( 3 \right)}}$ and $E\left( V_{i,j}V_{k,j} | C_{1},C_{2} \right)=\frac{1}{w_{i,j}^{\left( 3 \right)}}\times\frac{1}{w_{k,j}^{\left( 3 \right)}}$ if $i\neq k$, thus $\mathrm{cov}\left( V_{i,j},V_{k,j} | C_{1},C_{2} \right)=\sigma_{i,k,j}^{(3)}=0$ if $i\neq k$ and $\mathrm{var}\left( V_{i,j} | C_{1},C_{2} \right)=\sigma_{i,i,j}^{(3)}\equiv\sigma_{i,j}^{(3)}=\frac{1}{w_{i,j}^{\left( 3 \right)}}\left( 1-\frac{1}{w_{i,j}^{\left( 3 \right)}} \right)$; and obviously $E\left( V_{i,j}V_{k,l} | C_{1},C_{2} \right)=\frac{1}{w_{i,j}^{\left( 3 \right)}}\times\frac{1}{w_{k,l}^{\left( 3 \right)}}$ and $\mathrm{cov}\left( V_{i,j},V_{k,l} | C_{1},C_{2} \right)=0$ in stratum $j$ and stratum $l$, $j\neq l$. For our notation, we will also use $w_{i,k,j}=w_{i,k,j}^{\left( 2 \right)}\times w_{i,j}^{\left( 3 \right)}\times w_{k,j}^{\left( 3 \right)}$, for $i\neq k$, because $E\left( \xi_{i,j}\xi_{k,j}V_{i,j} V_{k,j}|C_{1} \right)=E\left\{ E\left( \xi_{i,j}\xi_{k,j}V_{i,j} V_{k,j}|C_{1},C_{2} \right)|C_{1} \right\}$ $=E\left\{ \xi_{i,j}\xi_{k,j}E\left( V_{i,j}|C_{1},C_{2} \right)E\left( V_{k,j}|C_{1},C_{2} \right)|C_{1} \right\}=\frac{1}{w_{i,j}^{\left( 3 \right)}}\times\frac{1}{w_{k,j}^{\left( 3 \right)}}\times E\left( \xi_{i,j}\xi_{k,j} |C_{1} \right)=\frac{1}{w_{i,j}^{\left( 3 \right)}}\times\frac{1}{w_{k,j}^{\left( 3 \right)}}\times\frac{1}{w_{i,k,j}^{\left( 2 \right)}}$,

and we will also use $w_{i,k,j}^{\left( 3 \right)}=w_{i,j}^{\left( 3 \right)}\times w_{k,j}^{\left( 3 \right)}$, for $i\neq k$, $w_{i,j}=w_{i,j}^{\left( 2 \right)}\times w_{i,j}^{\left( 3 \right)}$, $\sigma_{i,j}=\frac{1}{w_{i,j}^{\left( 2 \right)}}\times\frac{1}{w_{i,j}^{\left( 3 \right)}}\left( 1-\frac{1}{w_{i,j}^{\left( 2 \right)}}\times\frac{1}{w_{i,j}^{\left( 3 \right)}} \right)$ and $\sigma_{i,k,j}=\frac{1}{w_{i,k,j}}-\frac{1}{w_{i,j}}\times\frac{1}{w_{k,j}}=\frac{1}{w_{i,j}^{\left( 3 \right)}}\times\frac{1}{w_{k,j}^{\left( 3 \right)}}\times\sigma_{i,k,j}^{(2)}$, for $i\neq k$.

We have shown in Web Appendix E.1 that for any $\tilde{\boldsymbol{\theta}}\in\left\{ \tilde{\boldsymbol{\gamma}},\tilde{\boldsymbol{\beta}}, {d\tilde{\Lambda}}_{0}\left( t \right), \tilde{\Lambda}_{0}\left( t \right), \tilde{\pi}\left( \tau_{1},\tau_{2};\boldsymbol{x} \right) \right\}$, $\boldsymbol{\Delta}_{i,j}\left( \tilde{\boldsymbol{\theta}} \right)=\xi_{i,j}\boldsymbol{IF}_{i,j}^{(2)}\left( \tilde{\boldsymbol{\theta}} \right)+\xi_{i,j} V_{i,j}\tilde{w}_{i,j}^{\left( 3 \right)}\boldsymbol{IF}_{i,j}^{(3)}\left( \tilde{\boldsymbol{\theta}} \right)$, with $\tilde{w}_{i,j}^{\left( 3 \right)}=\exp\left( {\tilde{\boldsymbol{\gamma}}}^{'}\boldsymbol{B}_{i,j} \right)$. We have

$E\left\{ \sum_{j=1}^{J} \sum_{i=1}^{n^{\left( j \right)}} \boldsymbol{\Delta}_{i,j}\left( \tilde{\boldsymbol{\theta}} \right)|C_{1},C_{2} \right\}=\sum_{j=1}^{J} \sum_{i=1}^{n^{\left( j \right)}} \left\{ \xi_{i,j}\boldsymbol{IF}_{i,j}^{(2)}\left( \tilde{\boldsymbol{\theta}} \right)+E\left( V_{i,j} |C_{1},C_{2} \right)\xi_{i,j} \tilde{w}_{i,j}^{\left( 3 \right)}\boldsymbol{IF}_{i,j}^{(3)}\left( \tilde{\boldsymbol{\theta}} \right) \right\}$,

$=\sum_{j=1}^{J} \sum_{i=1}^{n^{\left( j \right)}} \left\{ \xi_{i,j}\boldsymbol{IF}_{i,j}^{(2)}\left( \tilde{\boldsymbol{\theta}} \right)+\xi_{i,j}\frac{\tilde{w}_{i,j}^{\left( 3 \right)}}{w_{i,j}^{\left( 3 \right)}}\boldsymbol{IF}_{i,j}^{(3)}\left( \tilde{\boldsymbol{\theta}} \right) \right\}$,

and we have

$\mathrm{var}\left\{ \sum_{j=1}^{J} \sum_{i=1}^{n^{\left( j \right)}} \boldsymbol{\Delta}_{i,j}\left( \tilde{\boldsymbol{\theta}} \right)|C_{1},C_{2} \right\}=\sum_{j=1}^{J} \sum_{i=1}^{n^{\left( j \right)}} var(V_{i,j}|C_{1},C_{2})\left\{ \xi_{i,j}\tilde{w}_{i,j}^{\left( 3 \right)}\boldsymbol{IF}_{i,j}^{(3)}\left( \tilde{\boldsymbol{\theta}} \right) \right\}\left\{ \xi_{i,j} \tilde{w}_{i,j}^{\left( 3 \right)}\boldsymbol{IF}_{i,j}^{(3)}\left( \tilde{\boldsymbol{\theta}} \right) \right\}^{'}=\sum_{j=1}^{J} \sum_{i=1}^{n^{\left( j \right)}} \sigma_{i,j}^{(3)} \xi_{i,j}\tilde{w}_{i,j}^{\left( 3 \right)}\tilde{w}_{i,j}^{\left( 3 \right)}\boldsymbol{IF}_{i,j}^{(3)}\left( \tilde{\boldsymbol{\theta}} \right){\boldsymbol{IF}_{i,j}^{(3)}\left( \tilde{\boldsymbol{\theta}} \right)}^{\boldsymbol{'}}$,

as indeed $\xi_{i,j}\boldsymbol{IF}_{i,j}^{(2)}\left( \tilde{\boldsymbol{\theta}} \right)$ is fixed conditional on $C_{2}$. Then we have

$E\left[ E\left\{ \sum_{j=1}^{J} \sum_{i=1}^{n^{\left( j \right)}} \boldsymbol{\Delta}_{i,j}\left( \tilde{\boldsymbol{\theta}} \right)|C_{1},C_{2} \right\}|C_{1} \right]=\sum_{j=1}^{J} \sum_{i=1}^{n^{\left( j \right)}} E\left( \xi_{i,j} |C_{1} \right) \boldsymbol{IF}_{i,j}^{(2)}\left( \tilde{\boldsymbol{\theta}} \right)+E\left( \xi_{i,j}|C_{1} \right)\frac{\tilde{w}_{i,j}^{\left( 3 \right)}}{w_{i,j}^{\left( 3 \right)}}\boldsymbol{IF}_{i,j}^{(3)}\left( \tilde{\boldsymbol{\theta}} \right)=\sum_{j=1}^{J} \sum_{i=1}^{n^{\left( j \right)}} \frac{1}{w_{i,j}^{\left( 2 \right)}}\left\{ \boldsymbol{IF}_{i,j}^{(2)}\left( \tilde{\boldsymbol{\theta}} \right)+\frac{\tilde{w}_{i,j}^{\left( 3 \right)}}{w_{i,j}^{\left( 3 \right)}} \boldsymbol{IF}_{i,j}^{(3)}\left( \tilde{\boldsymbol{\theta}} \right) \right\}$,

and

$\mathrm{var}\left[ E\left\{ \sum_{j=1}^{J} \sum_{i=1}^{n^{\left( j \right)}} \boldsymbol{\Delta}_{i,j}\left( \tilde{\boldsymbol{\theta}} \right)|C_{1},C_{2} \right\}|C_{1} \right]=\mathrm{var}\left\{ \sum_{j=1}^{J} \sum_{i=1}^{n^{\left( j \right)}} \xi_{i,j}\boldsymbol{IF}_{i,j}^{(2)}\left( \tilde{\boldsymbol{\theta}} \right)+\xi_{i,j}\frac{\tilde{w}_{i,j}^{\left( 3 \right)}}{w_{i,j}^{\left( 3 \right)}}\boldsymbol{IF}_{i,j}^{(3)}\left( \tilde{\boldsymbol{\theta}} \right) \right\}$,

$=\sum_{j=1}^{J} \mathrm{var}\left[ \sum_{i=1}^{n^{\left( j \right)}} \xi_{i,j}\left\{ \boldsymbol{IF}_{i,j}^{(2)}\left( \tilde{\boldsymbol{\theta}} \right)+\frac{\tilde{w}_{i,j}^{\left( 3 \right)}}{w_{i,j}^{\left( 3 \right)}}\boldsymbol{IF}_{i,j}^{(3)}\left( \tilde{\boldsymbol{\theta}} \right) \right\} \right]$,

$=\sum_{j=1}^{J} \sum_{i=1}^{n^{\left( j \right)}} \sum_{k=1}^{n^{\left( j \right)}} \sigma_{i,k,j}^{(2)}\left\{ \boldsymbol{IF}_{i,j}^{(2)}\left( \tilde{\boldsymbol{\theta}} \right)+\frac{\tilde{w}_{i,j}^{\left( 3 \right)}}{w_{i,j}^{\left( 3 \right)}}\boldsymbol{IF}_{i,j}^{(3)}\left( \tilde{\boldsymbol{\theta}} \right) \right\}\left\{ \boldsymbol{IF}_{k,j}^{(2)}\left( \tilde{\boldsymbol{\theta}} \right)+\frac{\tilde{w}_{k,j}^{\left( 3 \right)}}{w_{k,j}^{\left( 3 \right)}}\boldsymbol{IF}_{k,j}^{(3)}\left( \tilde{\boldsymbol{\theta}} \right) \right\}^{'}$,

and

$E\left[ \mathrm{var}\left\{ \sum_{j=1}^{J} \sum_{i=1}^{n^{\left( j \right)}} \boldsymbol{\Delta}_{i,j}\left( \tilde{\boldsymbol{\theta}} \right)|C_{1},C_{2} \right\}|C_{1} \right]=\sum_{j=1}^{J} \sum_{i=1}^{n^{\left( j \right)}} E\left( \xi_{i,j}|C_{1} \right)\sigma_{i,j}^{(3)} \tilde{w}_{i,j}^{\left( 3 \right)}\tilde{w}_{i,j}^{\left( 3 \right)}\boldsymbol{IF}_{i,j}^{(3)}\left( \tilde{\boldsymbol{\theta}} \right){\boldsymbol{IF}_{i,j}^{(3)}\left( \tilde{\boldsymbol{\theta}} \right)}^{\boldsymbol{'}}$,

$=\sum_{j=1}^{J} \sum_{i=1}^{n^{\left( j \right)}} \frac{1}{w_{i,j}^{\left( 2 \right)}}\sigma_{i,j}^{(3)} \tilde{w}_{i,j}^{\left( 3 \right)}\tilde{w}_{i,j}^{\left( 3 \right)}\boldsymbol{IF}_{i,j}^{(3)}\left( \tilde{\boldsymbol{\theta}} \right){\boldsymbol{IF}_{i,j}^{(3)}\left( \tilde{\boldsymbol{\theta}} \right)}^{\boldsymbol{'}}$.

Finally,

$\mathrm{var}\left( E\left[ E\left\{ \sum_{j=1}^{J} \sum_{i=1}^{n^{\left( j \right)}} \boldsymbol{\Delta}_{i,j}\left( \tilde{\boldsymbol{\theta}} \right)|C_{1},C_{2} \right\}|C_{1} \right] \right)=\mathrm{var}\left[ \sum_{j=1}^{J} \sum_{i=1}^{n^{\left( j \right)}} \frac{1}{w_{i,j}^{\left( 2 \right)}}\left\{ \boldsymbol{IF}_{i,j}^{(2)}\left( \tilde{\boldsymbol{\theta}} \right)+\frac{\tilde{w}_{i,j}^{\left( 3 \right)}}{w_{i,j}^{\left( 3 \right)}} \boldsymbol{IF}_{i,j}^{(3)}\left( \tilde{\boldsymbol{\theta}} \right) \right\} \right]$,

which can be estimated by $\frac{n}{n-1}\sum_{j=1}^{J} \sum_{i=1}^{n^{\left( j \right)}} \left( \frac{1}{w_{i,j}^{\left( 2 \right)}} \right)^{2}\left\{ \xi_{i,j}w_{i,j}^{\left( 2 \right)}\boldsymbol{IF}_{i,j}^{(2)}\left( \tilde{\boldsymbol{\theta}} \right){\boldsymbol{IF}_{i,j}^{(2)}\left( \tilde{\boldsymbol{\theta}} \right)}^{\boldsymbol{'}}+2 \xi_{i,j}V_{i,j} \tilde{w}_{i,j}\boldsymbol{IF}_{i,j}^{(2)}\left( \tilde{\boldsymbol{\theta}} \right){\boldsymbol{IF}_{i,j}^{(3)}\left( \tilde{\boldsymbol{\theta}} \right)}^{\boldsymbol{'}}+\xi_{i,j}V_{i,j} \tilde{w}_{i,j}\boldsymbol{IF}_{i,j}^{(3)}\left( \tilde{\boldsymbol{\theta}} \right){\boldsymbol{IF}_{i,j}^{(3)}\left( \tilde{\boldsymbol{\theta}} \right)}^{\boldsymbol{'}} \right\}$,

that is by $\frac{n}{n-1}\sum_{j=1}^{J} \sum_{i=1}^{n^{\left( j \right)}} \frac{1}{w_{i,j}^{\left( 2 \right)}}\left\{ \xi_{i,j}\boldsymbol{IF}_{i,j}^{(2)}\left( \tilde{\boldsymbol{\theta}} \right){\boldsymbol{IF}_{i,j}^{(2)}\left( \tilde{\boldsymbol{\theta}} \right)}^{\boldsymbol{'}}+2 \xi_{i,j}V_{i,j} \tilde{w}_{i,j}^{(3)}\boldsymbol{IF}_{i,j}^{(2)}\left( \tilde{\boldsymbol{\theta}} \right){\boldsymbol{IF}_{i,j}^{(3)}\left( \tilde{\boldsymbol{\theta}} \right)}^{\boldsymbol{'}}+\xi_{i,j}V_{i,j} \tilde{w}_{i,j}^{(3)}\boldsymbol{IF}_{i,j}^{(3)}\left( \tilde{\boldsymbol{\theta}} \right){\boldsymbol{IF}_{i,j}^{(3)}\left( \tilde{\boldsymbol{\theta}} \right)}^{\boldsymbol{'}} \right\}$,

or again by $\frac{n}{n-1}\sum_{j=1}^{J} \sum_{i=1}^{n^{\left( j \right)}} \frac{1}{w_{i,j}^{\left( 2 \right)}}\left\{ \xi_{i,j}\boldsymbol{IF}_{i,j}^{(2)}\left( \tilde{\boldsymbol{\theta}} \right){\boldsymbol{IF}_{i,j}^{(2)}\left( \tilde{\boldsymbol{\theta}} \right)}^{\boldsymbol{'}}+2 \xi_{i,j}V_{i,j} \tilde{w}_{i,j}^{(3)}\boldsymbol{IF}_{i,j}^{(2)}\left( \tilde{\boldsymbol{\theta}} \right){\boldsymbol{IF}_{i,j}^{(3)}\left( \tilde{\boldsymbol{\theta}} \right)}^{\boldsymbol{'}}+\frac{1}{\tilde{w}_{i,j}^{(3)}}\xi_{i,j}V_{i,j}\tilde{w}_{i,j}^{(3)}\tilde{w}_{i,j}^{(3)}\boldsymbol{IF}_{i,j}^{(3)}\left( \tilde{\boldsymbol{\theta}} \right){\boldsymbol{IF}_{i,j}^{(3)}\left( \tilde{\boldsymbol{\theta}} \right)}^{\boldsymbol{'}} \right\}$.

Then $E\left( \mathrm{var}\left[ E\left\{ \sum_{j=1}^{J} \sum_{i=1}^{n^{\left( j \right)}} \boldsymbol{\Delta}_{i,j}\left( \tilde{\boldsymbol{\theta}} \right)|C_{1},C_{2} \right\}|C_{1} \right] \right)=E\left[ \sum_{j=1}^{J} \sum_{i=1}^{n^{\left( j \right)}} \sum_{k=1}^{n^{\left( j \right)}} \sigma_{i,k,j}^{(2)}\left\{ \boldsymbol{IF}_{i,j}^{(2)}\left( \tilde{\boldsymbol{\theta}} \right)+\frac{\tilde{w}_{i,j}^{\left( 3 \right)}}{w_{i,j}^{\left( 3 \right)}}\boldsymbol{IF}_{i,j}^{(3)}\left( \tilde{\boldsymbol{\theta}} \right) \right\}\left\{ \boldsymbol{IF}_{k,j}^{(2)}\left( \tilde{\boldsymbol{\theta}} \right)+\frac{\tilde{w}_{k,j}^{\left( 3 \right)}}{w_{k,j}^{\left( 3 \right)}}\boldsymbol{IF}_{k,j}^{(3)}\left( \tilde{\boldsymbol{\theta}} \right) \right\}^{'} \right]$,

that can be estimated by

$\sum_{j=1}^{J} \sum_{i=1}^{n^{\left( j \right)}} \sigma_{i,j}^{(2)}\left\{ \xi_{i,j}w_{i,j}^{\left( 2 \right)}\boldsymbol{IF}_{i,j}^{(2)}\left( \tilde{\boldsymbol{\theta}} \right){\boldsymbol{IF}_{i,j}^{(2)}\left( \tilde{\boldsymbol{\theta}} \right)}^{\boldsymbol{'}}+2 \xi_{i,j}V_{i,j} w_{i,j}^{\left( 2 \right)} \tilde{w}_{i,j}^{\left( 3 \right)} \boldsymbol{IF}_{i,j}^{(2)}\left( \tilde{\boldsymbol{\theta}} \right){\boldsymbol{IF}_{i,j}^{(3)}\left( \tilde{\boldsymbol{\theta}} \right)}^{'}+\xi_{i,j}V_{i,j}w_{i,j}^{\left( 2 \right)} \tilde{w}_{i,j}^{\left( 3 \right)} \boldsymbol{IF}_{i,j}^{(3)}\left( \tilde{\boldsymbol{\theta}} \right){\boldsymbol{IF}_{i,j}^{(3)}\left( \tilde{\boldsymbol{\theta}} \right)}^{'} \right\}+\sum_{j=1}^{J} \sum_{i=1}^{n^{\left( j \right)}} \sum_{\begin{aligned} k=1, \\ k\neq i \end{aligned}}^{n^{\left( j \right)}} \sigma_{i,k,j}^{(2)}\left\{ \xi_{i,j}\xi_{k,j}w_{i,k,j}^{\left( 2 \right)}\boldsymbol{IF}_{i,j}^{(2)}\left( \tilde{\boldsymbol{\theta}} \right){\boldsymbol{IF}_{k,j}^{(2)}\left( \tilde{\boldsymbol{\theta}} \right)}^{\boldsymbol{'}}+\xi_{i,j}\xi_{k,j}V_{k,j}w_{i,k,j}^{\left( 2 \right)} \tilde{w}_{k,j}^{\left( 3 \right)}\boldsymbol{IF}_{i,j}^{(2)}\left( \tilde{\boldsymbol{\theta}} \right){\boldsymbol{IF}_{k,j}^{(3)}\left( \tilde{\boldsymbol{\theta}} \right)}^{'}+\xi_{i,j}\xi_{k,j}V_{i,j} w_{i,k,j}^{\left( 2 \right)} \tilde{w}_{i,j}^{\left( 3 \right)}\boldsymbol{IF}_{i,j}^{(3)}\left( \tilde{\boldsymbol{\theta}} \right){\boldsymbol{IF}_{k,j}^{(2)}\left( \tilde{\boldsymbol{\theta}} \right)}^{'}+\xi_{i,j}\xi_{k,j}V_{i,j} V_{k,j}\tilde{w}_{i,k,j}\boldsymbol{IF}_{i,j}^{(3)}\left( \tilde{\boldsymbol{\theta}} \right){\boldsymbol{IF}_{k,j}^{(3)}\left( \tilde{\boldsymbol{\theta}} \right)}^{'} \right\}$,

that is by

$\sum_{j=1}^{J} \sum_{i=1}^{n^{\left( j \right)}} \sigma_{i,j}^{(2)}w_{i,j}^{\left( 2 \right)}\left\{ \xi_{i,j}\boldsymbol{IF}_{i,j}^{(2)}\left( \tilde{\boldsymbol{\theta}} \right){\boldsymbol{IF}_{i,j}^{(2)}\left( \tilde{\boldsymbol{\theta}} \right)}^{\boldsymbol{'}}+2 \xi_{i,j}V_{i,j} \tilde{w}_{i,j}^{\left( 3 \right)}\boldsymbol{IF}_{i,j}^{(2)}\left( \tilde{\boldsymbol{\theta}} \right){\boldsymbol{IF}_{i,j}^{(3)}\left( \tilde{\boldsymbol{\theta}} \right)}^{'}+\frac{1}{\tilde{w}_{i,j}^{\left( 3 \right)}}\xi_{i,j}V_{i,j} \tilde{w}_{i,j}^{\left( 3 \right)}\tilde{w}_{i,j}^{\left( 3 \right)}\boldsymbol{IF}_{i,j}^{(3)}\left( \tilde{\boldsymbol{\theta}} \right){\boldsymbol{IF}_{i,j}^{(3)}\left( \tilde{\boldsymbol{\theta}} \right)}^{'} \right\}+\sum_{j=1}^{J} \sum_{i=1}^{n^{\left( j \right)}} \sum_{\begin{aligned} k=1, \\ k\neq i \end{aligned}}^{n^{\left( j \right)}} \sigma_{i,k,j}^{(2)} w_{i,k,j}^{\left( 2 \right)}\left\{ \xi_{i,j}\boldsymbol{IF}_{i,j}^{(2)}\left( \tilde{\boldsymbol{\theta}} \right)+\xi_{i,j}V_{i,j} \tilde{w}_{i,j}^{\left( 3 \right)}\boldsymbol{IF}_{i,j}^{(3)}\left( \tilde{\boldsymbol{\theta}} \right) \right\}\left\{ \xi_{k,j}\boldsymbol{IF}_{k,j}^{(2)}\left( \tilde{\boldsymbol{\theta}} \right)+\xi_{k,j}V_{k,j}\tilde{w}_{k,j}^{\left( 3 \right)}\boldsymbol{IF}_{i,j}^{(3)}\left( \tilde{\boldsymbol{\theta}} \right) \right\}^{'}$,

as indeed $\tilde{w}_{i,k,j}=w_{i,k,j}^{\left( 2 \right)}\times\tilde{w}_{i,j}^{\left( 3 \right)}\times\tilde{w}_{k,j}^{\left( 3 \right)}$, and

$E\left( E\left[ \mathrm{var}\left\{ \sum_{j=1}^{J} \sum_{i=1}^{n^{\left( j \right)}} \boldsymbol{\Delta}_{i,j}\left( \tilde{\boldsymbol{\theta}} \right)|C_{1},C_{2} \right\}|C_{1} \right] \right)=E\left\{ \sum_{j=1}^{J} \sum_{i=1}^{n^{\left( j \right)}} \frac{1}{w_{i,j}^{\left( 2 \right)}}\sigma_{i,j}^{(3)}\tilde{w}_{i,j}^{\left( 3 \right)}\tilde{w}_{i,j}^{\left( 3 \right)}\boldsymbol{IF}_{i,j}^{(3)}\left( \tilde{\boldsymbol{\theta}} \right){\boldsymbol{IF}_{i,j}^{(3)}\left( \tilde{\boldsymbol{\theta}} \right)}^{\boldsymbol{'}} \right\}$, that can be estimated by $\sum_{j=1}^{J} \sum_{i=1}^{n^{\left( j \right)}} \tilde{\sigma}_{i,j}^{(3)}\tilde{w}_{i,j}^{\left( 3 \right)}\xi_{i,j}V_{i,j}\tilde{w}_{i,j}^{\left( 3 \right)}\tilde{w}_{i,j}^{\left( 3 \right)}\boldsymbol{IF}_{i,j}^{(3)}\left( \tilde{\boldsymbol{\theta}} \right){\boldsymbol{IF}_{i,j}^{(3)}\left( \tilde{\boldsymbol{\theta}} \right)}^{\boldsymbol{'}}$.

As a result, $\mathrm{var}\left( \tilde{\boldsymbol{\theta}} \right)$ can be estimated by

$\frac{n}{n-1}\sum_{j=1}^{J} \sum_{i=1}^{n^{\left( j \right)}} \frac{1}{w_{i,j}^{\left( 2 \right)}}\left\{ \xi_{i,j}\boldsymbol{IF}_{i,j}^{(2)}\left( \tilde{\boldsymbol{\theta}} \right){\boldsymbol{IF}_{i,j}^{(2)}\left( \tilde{\boldsymbol{\theta}} \right)}^{\boldsymbol{'}}+2 \xi_{i,j}V_{i,j} \tilde{w}_{i,j}^{(3)}\boldsymbol{IF}_{i,j}^{(2)}\left( \tilde{\boldsymbol{\theta}} \right){\boldsymbol{IF}_{i,j}^{(3)}\left( \tilde{\boldsymbol{\theta}} \right)}^{\boldsymbol{'}}+\frac{1}{\tilde{w}_{i,j}^{(3)}}\xi_{i,j}V_{i,j}\tilde{w}_{i,j}^{(3)}\tilde{w}_{i,j}^{(3)}\boldsymbol{IF}_{i,j}^{(3)}\left( \tilde{\boldsymbol{\theta}} \right){\boldsymbol{IF}_{i,j}^{(3)}\left( \tilde{\boldsymbol{\theta}} \right)}^{\boldsymbol{'}} \right\}+\sum_{j=1}^{J} \sum_{i=1}^{n^{\left( j \right)}} \sigma_{i,j}^{(2)}w_{i,j}^{\left( 2 \right)}\left\{ \xi_{i,j}\boldsymbol{IF}_{i,j}^{(2)}\left( \tilde{\boldsymbol{\theta}} \right){\boldsymbol{IF}_{i,j}^{(2)}\left( \tilde{\boldsymbol{\theta}} \right)}^{\boldsymbol{'}}+2 \xi_{i,j}V_{i,j} \tilde{w}_{i,j}^{\left( 3 \right)}\boldsymbol{IF}_{i,j}^{(2)}\left( \tilde{\boldsymbol{\theta}} \right){\boldsymbol{IF}_{i,j}^{(3)}\left( \tilde{\boldsymbol{\theta}} \right)}^{'}+\frac{1}{\tilde{w}_{i,j}^{\left( 3 \right)}}\xi_{i,j}V_{i,j} \tilde{w}_{i,j}^{\left( 3 \right)}\tilde{w}_{i,j}^{\left( 3 \right)}\boldsymbol{IF}_{i,j}^{(3)}\left( \tilde{\boldsymbol{\theta}} \right){\boldsymbol{IF}_{i,j}^{(3)}\left( \tilde{\boldsymbol{\theta}} \right)}^{'} \right\}+\sum_{j=1}^{J} \sum_{i=1}^{n^{\left( j \right)}} \sum_{\begin{aligned} k=1, \\ k\neq i \end{aligned}}^{n^{\left( j \right)}} \sigma_{i,k,j}^{(2)} w_{i,k,j}^{\left( 2 \right)}\left\{ \xi_{i,j}\boldsymbol{IF}_{i,j}^{(2)}\left( \tilde{\boldsymbol{\theta}} \right)+\xi_{i,j}V_{i,j} \tilde{w}_{i,j}^{\left( 3 \right)}\boldsymbol{IF}_{i,j}^{(3)}\left( \tilde{\boldsymbol{\theta}} \right) \right\}\left\{ \xi_{k,j}\boldsymbol{IF}_{k,j}^{(2)}\left( \tilde{\boldsymbol{\theta}} \right)+\xi_{k,j}V_{k,j}\tilde{w}_{k,j}^{\left( 3 \right)}\boldsymbol{IF}_{i,j}^{(3)}\left( \tilde{\boldsymbol{\theta}} \right) \right\}^{'}+\sum_{j=1}^{J} \sum_{i=1}^{n^{\left( j \right)}} \tilde{\sigma}_{i,j}^{(3)}\tilde{w}_{i,j}^{\left( 3 \right)}\xi_{i,j}V_{i,j}\tilde{w}_{i,j}^{\left( 3 \right)}\tilde{w}_{i,j}^{\left( 3 \right)}\boldsymbol{IF}_{i,j}^{(3)}\left( \tilde{\boldsymbol{\theta}} \right){\boldsymbol{IF}_{i,j}^{(3)}\left( \tilde{\boldsymbol{\theta}} \right)}^{\boldsymbol{'}}$.

Note that we can rewrite this quantity as

$\frac{n}{n-1}\sum_{j=1}^{J} \sum_{i=1}^{n^{\left( j \right)}} \frac{1}{w_{i,j}^{\left( 2 \right)}}\left\{ \xi_{i,j}\boldsymbol{IF}_{i,j}^{(2)}\left( \tilde{\boldsymbol{\theta}} \right){\boldsymbol{IF}_{i,j}^{(2)}\left( \tilde{\boldsymbol{\theta}} \right)}^{\boldsymbol{'}}+2 \xi_{i,j}V_{i,j} \tilde{w}_{i,j}^{\left( 3 \right)}\boldsymbol{IF}_{i,j}^{(2)}\left( \tilde{\boldsymbol{\theta}} \right){\boldsymbol{IF}_{i,j}^{(3)}\left( \tilde{\boldsymbol{\theta}} \right)}^{\boldsymbol{'}}+\frac{1}{\tilde{w}_{i,j}^{(3)}}\xi_{i,j}V_{i,j}\tilde{w}_{i,j}^{(3)}\tilde{w}_{i,j}^{(3)}\boldsymbol{IF}_{i,j}^{(3)}\left( \tilde{\boldsymbol{\theta}} \right){\boldsymbol{IF}_{i,j}^{(3)}\left( \tilde{\boldsymbol{\theta}} \right)}^{\boldsymbol{'}} \right\}+\sum_{j=1}^{J} \sum_{i=1}^{n^{\left( j \right)}} \sum_{k=1}^{n^{\left( j \right)}} \sigma_{i,k,j}^{(2)} w_{i,k,j}^{\left( 2 \right)}\left\{ \xi_{i,j}\boldsymbol{IF}_{i,j}^{(2)}\left( \tilde{\boldsymbol{\theta}} \right)+\xi_{i,j}V_{i,j} \tilde{w}_{i,j}^{\left( 3 \right)}\boldsymbol{IF}_{i,j}^{(3)}\left( \tilde{\boldsymbol{\theta}} \right) \right\}\left\{ \xi_{k,j}\boldsymbol{IF}_{k,j}^{(2)}\left( \tilde{\boldsymbol{\theta}} \right)+\xi_{k,j}V_{k,j}\tilde{w}_{k,j}^{\left( 3 \right)}\boldsymbol{IF}_{i,j}^{(3)}\left( \tilde{\boldsymbol{\theta}} \right) \right\}^{'}\boldsymbol{+}\sum_{j=1}^{J} \sum_{i=1}^{n^{\left( j \right)}} \frac{1}{w_{i,j}^{\left( 2 \right)}}\tilde{\sigma}_{i,j}^{(3)}\tilde{w}_{i,j}^{\left( 3 \right)}\left\{ \xi_{i,j}V_{i,j} \tilde{w}_{i,j}^{\left( 3 \right)}\tilde{w}_{i,j}^{\left( 3 \right)}\boldsymbol{IF}_{i,j}^{(3)}\left( \tilde{\boldsymbol{\theta}} \right){\boldsymbol{IF}_{i,j}^{(3)}\left( \tilde{\boldsymbol{\theta}} \right)}^{\boldsymbol{'}} \right\}$,

or also as

$\frac{n}{n-1}\sum_{j=1}^{J} \sum_{i=1}^{n^{\left( j \right)}} \frac{1}{w_{i,j}^{\left( 2 \right)}}\left\{ \xi_{i,j}\boldsymbol{IF}_{i,j}^{(2)}\left( \tilde{\boldsymbol{\theta}} \right){\boldsymbol{IF}_{i,j}^{(2)}\left( \tilde{\boldsymbol{\theta}} \right)}^{\boldsymbol{'}}+2 \xi_{i,j}V_{i,j} \tilde{w}_{i,j}^{\left( 3 \right)}\boldsymbol{IF}_{i,j}^{(2)}\left( \tilde{\boldsymbol{\theta}} \right){\boldsymbol{IF}_{i,j}^{(3)}\left( \tilde{\boldsymbol{\theta}} \right)}^{\boldsymbol{'}}+\frac{1}{\tilde{w}_{i,j}^{(3)}}\xi_{i,j}V_{i,j}\tilde{w}_{i,j}^{(3)}\tilde{w}_{i,j}^{(3)}\boldsymbol{IF}_{i,j}^{(3)}\left( \tilde{\boldsymbol{\theta}} \right){\boldsymbol{IF}_{i,j}^{(3)}\left( \tilde{\boldsymbol{\theta}} \right)}^{\boldsymbol{'}} \right\}+\sum_{j=1}^{J} \sum_{i=1}^{n^{\left( j \right)}} \sum_{k=1}^{n^{\left( j \right)}} \tilde{\sigma}_{i,k,j} \tilde{w}_{i,k,j}\left\{ \xi_{i,j}\boldsymbol{IF}_{i,j}^{(2)}\left( \tilde{\boldsymbol{\theta}} \right)+\xi_{i,j}V_{i,j} \tilde{w}_{i,j}^{\left( 3 \right)}\boldsymbol{IF}_{i,j}^{(3)}\left( \tilde{\boldsymbol{\theta}} \right) \right\}\left\{ \xi_{k,j}\boldsymbol{IF}_{k,j}^{(2)}\left( \tilde{\boldsymbol{\theta}} \right)+\xi_{k,j}V_{k,j}\tilde{w}_{k,j}^{\left( 3 \right)}\boldsymbol{IF}_{i,j}^{(3)}\left( \tilde{\boldsymbol{\theta}} \right) \right\}^{'}\boldsymbol{-}\sum_{j=1}^{J} \sum_{i=1}^{n^{\left( j \right)}} \frac{1}{w_{i,j}^{\left( 2 \right)}}\tilde{\sigma}_{i,j}^{(3)}\tilde{w}_{i,j}^{\left( 3 \right)}\left\{ \xi_{i,j}\boldsymbol{IF}_{i,j}^{(2)}\left( \tilde{\boldsymbol{\theta}} \right){\boldsymbol{IF}_{i,j}^{(2)}\left( \tilde{\boldsymbol{\theta}} \right)}^{\boldsymbol{'}}\boldsymbol{+}2 \xi_{i,j}V_{i,j} \tilde{w}_{i,j}^{\left( 3 \right)}\boldsymbol{IF}_{i,j}^{(2)}\left( \tilde{\boldsymbol{\theta}} \right){\boldsymbol{IF}_{i,j}^{(3)}\left( \tilde{\boldsymbol{\theta}} \right)}^{\boldsymbol{'}} \right\}$.

# PHASE-TWO MISSING DATA WHEN THE PHASE-THREE DESIGN SAMPLING PROBABILITIES ARE KNOWN

## Parameters estimation

Here, we assume that the phase-three design sampling probabilities are known; in that case, the phase-three design weights $w_{i,j}^{\left( 3 \right)}$ and variances $\sigma_{i,j}^{(3)}$ are known too, and do not need to be estimated, $i\in\left\{ 1,\ldots,n^{(j)} \right\}$, $j\in\left\{ 1,\ldots,J \right\}$. From the available data, we estimate the log-relative hazard by solving in $\boldsymbol{\beta}$ the estimating equation

$\sum_{j=1}^{J} \sum_{i=1}^{n^{(j)}} \int_{t} V_{i,j} w_{i,j}^{\left( 3 \right)}\left\{ \boldsymbol{X}_{i,j}-\frac{\boldsymbol{S}_{\boldsymbol{1}}\left( t;\boldsymbol{\beta} \right)}{S_{0}\left( t;\boldsymbol{\beta} \right)} \right\}{dN}_{i,j}\left( t \right)=0$,

with $S_{0}\left( t;\boldsymbol{\beta} \right)=\sum_{j=1}^{J} \sum_{k=1}^{n^{\left( j \right)}} \xi_{k,j}{V_{k,j} w}_{k,j} Y_{k,j}\left( t \right)\exp\left( \boldsymbol{\beta}^{'}\boldsymbol{X}_{k,j} \right)$ and $\boldsymbol{S}_{1}\left( t;\boldsymbol{\beta} \right)=\sum_{j=1}^{J} \sum_{k=1}^{n^{\left( j \right)}} \xi_{k,j}{V_{k,j} w}_{k,j} Y_{k,j}\left( t \right)\exp\left( \boldsymbol{\beta}^{'}X_{k,j} \right)\boldsymbol{X}_{k,j}$, and we estimate the baseline hazard point mass at time $t$ by ${d\hat{\Lambda}}_{0}\left( t; \hat{\boldsymbol{\beta}} \right)\equiv{d\hat{\Lambda}}_{0}\left( t \right)=\frac{\sum_{j=1}^{J} \sum_{i=1}^{n^{(j)}} {dN}_{i,j}\left( t \right)}{S_{0}\left( t;\hat{\boldsymbol{\beta}} \right)}$. We then estimate the cumulative baseline hazard up to time $t$ by $\hat{\Lambda}_{0}\left( t;\hat{\boldsymbol{\beta}} \right)\equiv\hat{\Lambda}_{0}\left( t \right)=\int_{0}^{t} d\hat{\Lambda}_{0}\left( s \right)$, and we finally estimate the pure risk as

$\hat{\pi}\left( \tau_{1},\tau_{2}; \boldsymbol{x},\hat{\boldsymbol{\beta}}, d\hat{\Lambda}_{0} \right)\equiv\hat{\pi}\left( \tau_{1},\tau_{2};\boldsymbol{x} \right)=1-\exp\left\{ -\int_{\tau_{1}}^{\tau_{2}} \exp\left( \hat{\boldsymbol{\beta}}'\boldsymbol{x} \right){d\hat{\Lambda}}_{0}\left( t \right) \right\}$.

We assume that failure time and case-status are known for all $n$ individuals in the cohort; then, and as in the Main Document, we leave the numerator of the modified non-parametric Breslow estimator unchanged. In addition, observe that the estimating equation given above could be rewritten as $\sum_{j=1}^{J} \sum_{i=1}^{n^{(j)}} \int_{t} \xi_{i,j} V_{i,j} w_{i,j}\left\{ X_{i,j}-\frac{\boldsymbol{S}_{1}\left( t;\boldsymbol{\beta} \right)}{S_{0}\left( t;\beta\right)} \right\}{dN}_{i,j}\left( t \right)=0$ and that ${d\hat{\Lambda}}_{0}\left( t \right)$ could be rewritten as $\frac{\sum_{j=1}^{J} \sum_{i=1}^{n^{(j)}} \xi_{i,j} w_{i,j}^{\left( 2 \right)}{dN}_{i,j}\left( t \right)}{S_{0}\left( t;\hat{\boldsymbol{\beta}} \right)}$, as indeed all the cases are initially included in the stratified case-cohort (i.e., included in the phase-two sample) and have unit phase-two sampling weights, and thus $\xi_{i,j} w_{i,j}^{\left( 2 \right)}=1$ for any subject $i$ in stratum $j$ such that $\int_{t} {dN}_{i,j}\left( t \right)=1$.

## Derivation of the influence functions

We let $\boldsymbol{\Delta}_{i,j}\left( \hat{\boldsymbol{\theta}} \right)$ denote the influence of subject $i$ in stratum $j$ on $\hat{\boldsymbol{\theta}}$, $i\in\{1,\ldots,n^{(j)}\}$,$j\in\left\{ 1,\ldots,J \right\}$, $\hat{\boldsymbol{\theta}}\in\left\{ \hat{\boldsymbol{\beta}},{d\hat{\Lambda}}_{0}\left( t \right), \hat{\Lambda}_{0}\left( t \right), \hat{\pi}\left( \tau_{1},\tau_{2};\boldsymbol{x} \right) \right\}$. Because the derivations are similar to that in Web Appendices B.1 and C.2 we do not give details. We have

$\boldsymbol{\Delta}_{i,j}\left( \hat{\boldsymbol{\beta}} \right)=\xi_{i,j}\boldsymbol{IF}_{i,j}^{(2)}\left( \hat{\boldsymbol{\beta}} \right)+\xi_{i,j}V_{i,j}w_{i,j} \boldsymbol{IF}_{i,j}^{(3)}\left( \hat{\boldsymbol{\beta}} \right)$,

with $\boldsymbol{IF}_{i,j}^{(2)}\left( \hat{\boldsymbol{\beta}} \right)=0$,

$\boldsymbol{IF}_{i,j}^{(3)}\left( \hat{\boldsymbol{\beta}} \right)=\left[ \sum_{l=1}^{J} \sum_{k=1}^{n^{(l)}} \int_{t} \xi_{k,l}V_{k,l}w_{k,l}\left\{ \frac{\boldsymbol{S}_{2}\left( t;\hat{\boldsymbol{\beta}} \right)}{S_{0}\left( t;\hat{\boldsymbol{\beta}} \right)}-\frac{\boldsymbol{S}_{1}\left( t;\hat{\boldsymbol{\beta}} \right){\boldsymbol{S}_{1}\left( t;\hat{\boldsymbol{\beta}} \right)}^{'}}{{S_{0}\left( t;\hat{\boldsymbol{\beta}} \right)}^{2}} \right\}{dN}_{k,l}\left( t \right) \right]^{-1}\times\left[ \int_{t} \left\{ \boldsymbol{X}_{i,j}-\frac{\boldsymbol{S}_{1}\left( t;\hat{\boldsymbol{\beta}} \right)}{S_{0}\left( t;\hat{\boldsymbol{\beta}} \right)} \right\}\left\{ {dN}_{i,j}\left( t \right)-Y_{i,j}\left( t \right)\exp\left( {\hat{\boldsymbol{\beta}}}^{'}\boldsymbol{X}_{i,j} \right)\frac{\sum_{l=1}^{J} \sum_{k=1}^{n^{(l)}} {\xi_{k,l}V_{k,l}w_{k,l} dN}_{k,l}\left( t \right)}{S_{0}\left( t;\hat{\boldsymbol{\beta}} \right)} \right\} \right]$ ,

and $\boldsymbol{S}_{2}\left( t;\hat{\boldsymbol{\beta}} \right)=\sum_{j=1}^{J} \sum_{k=1}^{n^{\left( j \right)}} \xi_{k,j}{V_{k,j} w}_{k,j} Y_{k,j}\left( t \right)\exp\left( {\hat{\boldsymbol{\beta}}}^{'}\boldsymbol{X}_{k,j} \right)\boldsymbol{X}_{k,j} \boldsymbol{X}_{k,j}'$.

Then $\Delta_{i,j}\left\{ {d\hat{\Lambda}}_{0}\left( t \right) \right\}=\xi_{i,j}{IF}_{i,j}^{(2)}\left\{ {d\hat{\Lambda}}_{0}\left( t \right) \right\}+\xi_{i,j}V_{i,j}w_{i,j} {IF}_{i,j}^{(3)}\left\{ {d\hat{\Lambda}}_{0}\left( t \right) \right\}$, with ${IF}_{i,j}^{(2)}\left\{ {d\hat{\Lambda}}_{0}\left( t \right) \right\}{=\left\{ S_{0}\left( t;\hat{\beta} \right) \right\}}^{-1}{dN}_{i,j}$ and ${IF}_{i,j}^{(3)}\left\{ {d\hat{\Lambda}}_{0}\left( t \right) \right\}{=-\left\{ S_{0}\left( t;\hat{\beta} \right) \right\}}^{-1}\left\{ {d\hat{\Lambda}}_{0}\left( t \right){\boldsymbol{S}_{1}\left( t;\hat{\boldsymbol{\beta}} \right)}^{'} \boldsymbol{IF}_{i,j}^{\left( 2,3 \right)}\left( \hat{\boldsymbol{\beta}} \right)+{d\hat{\Lambda}}_{0}\left( t \right) Y_{i,j}\left( t \right)\exp\left( {\hat{\boldsymbol{\beta}}}^{'}\boldsymbol{X}_{i,j} \right) \right\}$.

Note that ${IF}_{i,j}^{(2)}\left\{ {d\hat{\Lambda}}_{0}\left( t \right) \right\}$ and ${IF}_{i,j}^{(3)}\left\{ {d\hat{\Lambda}}_{0}\left( t \right) \right\}$are linear combinations of the increments ${dN}_{i,j}\left( t \right)$ and ${d\tilde{\Lambda}}_{0}\left( t \right)$. Hence $\Delta_{i,j}\left\{ \int_{\tau_{1}}^{\tau_{2}} {d\hat{\Lambda}}_{0}\left( t \right) \right\}=\xi_{i,j}{IF}_{i,j}^{(2)}\left\{ \int_{\tau_{1}}^{\tau_{2}} {d\hat{\Lambda}}_{0}\left( t \right) \right\}+\xi_{i,j}V_{i,j}w_{i,j} {IF}_{i,j}^{(3)}\left\{ \int_{\tau_{1}}^{\tau_{2}} {d\hat{\Lambda}}_{0}\left( t \right) \right\}$, with ${IF}_{i,j}^{(s)}\left\{ \int_{\tau_{1}}^{\tau_{2}} {d\hat{\Lambda}}_{0}\left( t \right) \right\}=\int_{\tau_{1}}^{\tau_{2}} {IF}_{i,j}^{(s)}\left\{ {d\hat{\Lambda}}_{0}\left( s \right) \right\}$, $s\in\{2,3\}$. Finally, $\Delta_{i,j}\left\{ \hat{\pi}\left( \tau_{1},\tau_{2}; x \right) \right\}=\xi_{i,j}{IF}_{i,j}^{(2)}\left\{ \hat{\pi}\left( \tau_{1},\tau_{2}; \boldsymbol{x} \right) \right\}+\xi_{i,j}V_{i,j}w_{i,j} {IF}_{i,j}^{(3)}\left\{ \hat{\pi}\left( \tau_{1},\tau_{2}; \boldsymbol{x} \right) \right\}$, with ${IF}_{i,j}^{(s)}\left\{ \hat{\pi}\left( \tau_{1},\tau_{2}; \boldsymbol{x} \right) \right\}=\left\{ {\frac{\partial\hat{\pi}\left( \tau_{1},\tau_{2}; \boldsymbol{x} \right)}{\partial\boldsymbol{\beta}}}_{|\boldsymbol{\beta}=\hat{\boldsymbol{\beta}}} \right\}\boldsymbol{IF}_{i,j}^{(s)}+\left[ {\frac{\partial\hat{\pi}\left( \tau_{1},\tau_{2}; \boldsymbol{x} \right)}{\partial\left\{ \int_{\tau_{1}}^{\tau_{2}} {d\Lambda}_{0}\left( t \right) \right\}}}_{|{d\Lambda}_{0}(t)={d\hat{\Lambda}}_{0}\left( t \right)} \right]\Delta_{i,j}\left\{ \int_{\tau_{1}}^{\tau_{2}} {d\hat{\Lambda}}_{0}\left( t \right) \right\}$, $s\in\left\{ 2,3 \right\}$, and with

${\frac{\partial\hat{\pi}\left( \tau_{1},\tau_{2}; \boldsymbol{x} \right)}{\partial\boldsymbol{\beta}}}_{|\boldsymbol{\beta}=\hat{\boldsymbol{\beta}}}=\left\{ \sum_{t=\tau_{1}}^{\tau_{2}} \hat{\lambda}_{0}\left( t \right)\exp\left( \hat{\boldsymbol{\beta}}'\boldsymbol{x} \right) \right\}\left\{ 1-\hat{\pi}\left( \tau_{1},\tau_{2};\boldsymbol{x} \right) \right\} \boldsymbol{x'}$,

${\frac{\partial\tilde{\pi}\left( \tau_{1},\tau_{2}; \boldsymbol{x} \right)}{\partial\Lambda_{0}(\tau_{1})}}_{|\Lambda_{0}(\tau_{1})=\tilde{\Lambda}_{0}\left( \tau_{1} \right)}=\exp\left( \tilde{\boldsymbol{\beta}}'\boldsymbol{x} \right)\left\{ 1-\tilde{\pi}\left( \tau_{1},\tau_{2}; \boldsymbol{x} \right) \right\}$,

and ${\frac{\partial\hat{\pi}\left( \tau_{1},\tau_{2}; \boldsymbol{x} \right)}{\partial\left\{ \int_{\tau_{1}}^{\tau_{2}} {d\Lambda}_{0}\left( t \right) \right\}}}_{|{d\Lambda}_{0}(t)={d\hat{\Lambda}}_{0}\left( t \right)}=-\exp\left( \hat{\boldsymbol{\beta}}'\boldsymbol{x} \right)\left\{ 1-\hat{\pi}\left( \tau_{1},\tau_{2};\boldsymbol{x} \right) \right\}$.

Here, $\boldsymbol{\Delta}_{i,j}\left( \hat{\boldsymbol{\beta}} \right)$ is zero if subject $i$ in stratum $j$ has not been sampled in both the second and third phase of sampling, $i\in\{1,\ldots,n^{(j)}\}$,$j\in\left\{ 1,\ldots,J \right\}$; thus, a case may have a zero influence on $\hat{\boldsymbol{\beta}}$. On the other hand, $\xi_{i,j}V_{i,j}w_{i,j} {IF}_{i,j}^{(3)}\left\{ {d\hat{\Lambda}}_{0}\left( t \right) \right\}$, $\xi_{i,j}V_{i,j}w_{i,j} {IF}_{i,j}^{(3)}\left\{ \int_{\tau_{1}}^{\tau_{2}} {d\hat{\Lambda}}_{0}\left( t \right) \right\}$ and $\xi_{i,j}V_{i,j}w_{i,j} {IF}_{i,j}^{(3)}\left\{ \hat{\pi}\left( \tau_{1},\tau_{2}; \boldsymbol{x} \right) \right\}$ are also zero if subject $i$ in stratum $j$ has not been sampled in both the second and third phase of sampling, but $\xi_{i,j} {IF}_{i,j}^{(2)}\left\{ {d\hat{\Lambda}}_{0}\left( t \right) \right\}$, $\xi_{i,j} {IF}_{i,j}^{(2)}\left\{ \int_{\tau_{1}}^{\tau_{2}} {d\hat{\Lambda}}_{0}\left( t \right) \right\}$ and $\xi_{i,j} {IF}_{i,j}^{(2)}\left\{ \hat{\pi}\left( \tau_{1},\tau_{2}; \boldsymbol{x} \right) \right\}$ may be non-zero. Indeed, because we choose not to reweight the numerator of the Breslow estimator (i.e., we choose to use the actual failure times of the observed cases), even if case $i$ in stratum $j$ has missing covariate data (i.e., has not been sampled in the third phase of sampling), he/she has a non-zero influence on ${d\hat{\Lambda}}_{0}\left( t \right)$, equal to $\left\{ S_{0}\left( t;\hat{\boldsymbol{\beta}} \right) \right\}^{-1}{dN}_{i,j}\left( t \right)$, at her/his time of failure $t$, and subsequently may have a non-zero influence on $\hat{\Lambda}_{0}\left( t \right)$ and $\hat{\pi}\left( \tau_{1},\tau_{2}; x \right)$.

## Variance decomposition and estimation from influence functions

For any $\hat{\boldsymbol{\theta}}\in\left\{ \hat{\boldsymbol{\beta}}, {d\hat{\Lambda}}_{0}\left( t \right), \hat{\Lambda}_{0}\left( t \right), \hat{\pi}\left( \tau_{1},\tau_{2};\boldsymbol{x} \right) \right\}$, the variance based on the influences is $\mathrm{var}\left\{ \sum_{j=1}^{J} \sum_{i=1}^{n^{\left( j \right)}} \boldsymbol{\Delta}_{i,j}\left( \hat{\boldsymbol{\theta}} \right) \right\}$. Using the law of total covariance and the law of total expectation, it can be decomposed as

$\mathrm{var}\left( E\left[ E\left\{ \sum_{j=1}^{J} \sum_{i=1}^{n^{\left( j \right)}} \boldsymbol{\Delta}_{i,j}\left( \hat{\boldsymbol{\theta}} \right)|C_{1},C_{2} \right\}|C_{1} \right] \right)+ E\left( \mathrm{var}\left[ E\left\{ \sum_{j=1}^{J} \sum_{i=1}^{n^{\left( j \right)}} \boldsymbol{\Delta}_{i,j}\left( \hat{\boldsymbol{\theta}} \right)|C_{1},C_{2} \right\}|C_{1} \right] \right)+ E\left( E\left[ \mathrm{var}\left\{ \sum_{j=1}^{J} \sum_{i=1}^{n^{\left( j \right)}} \boldsymbol{\Delta}_{i,j}\left( \hat{\boldsymbol{\theta}} \right)|C_{1},C_{2} \right\}|C_{1} \right] \right)$,

where $C_{1}$ denote the information from the whole cohort, and $C_{2}$ denote the information from the phase-two sample.

We have shown in Web Appendix F.2 that $\boldsymbol{\Delta}_{i,j}\left( \hat{\boldsymbol{\theta}} \right)=\xi_{i,j}\boldsymbol{IF}_{i,j}^{(2)}\left( \hat{\boldsymbol{\theta}} \right)+\xi_{i,j} V_{i,j} w_{i,j} \boldsymbol{IF}_{i,j}^{(3)}\left( \hat{\boldsymbol{\theta}} \right)$, with $\boldsymbol{IF}_{i,j}^{(2)}\left( \hat{\boldsymbol{\beta}} \right)=0$. Using similar arguments as in Web Appendix E.2, we have

$E\left\{ \sum_{j=1}^{J} \sum_{i=1}^{n^{\left( j \right)}} \boldsymbol{\Delta}_{i,j}\left( \hat{\boldsymbol{\theta}} \right)|C_{1},C_{2} \right\}=\sum_{j=1}^{J} \sum_{i=1}^{n^{\left( j \right)}} \left\{ \xi_{i,j}\boldsymbol{IF}_{i,j}^{(2)}\left( \hat{\boldsymbol{\theta}} \right)+E\left( V_{i,j} w_{i,j}^{\left( 3 \right)}|C_{1},C_{2} \right)\xi_{i,j} w_{i,j}^{\left( 2 \right)}\boldsymbol{IF}_{i,j}^{(3)}\left( \hat{\boldsymbol{\theta}} \right) \right\}$,

$=\sum_{j=1}^{J} \sum_{i=1}^{n^{\left( j \right)}} \xi_{i,j}\left\{ \boldsymbol{IF}_{i,j}^{(2)}\left( \hat{\boldsymbol{\theta}} \right)+w_{i,j}^{\left( 2 \right)}\boldsymbol{IF}_{i,j}^{(3)}\left( \hat{\boldsymbol{\theta}} \right) \right\}$,

and we have

$\mathrm{var}\left\{ \sum_{j=1}^{J} \sum_{i=1}^{n^{\left( j \right)}} \boldsymbol{\Delta}_{i,j}\left( \hat{\boldsymbol{\theta}} \right)|C_{1},C_{2} \right\}=\sum_{j=1}^{J} \sum_{i=1}^{n^{\left( j \right)}} var(V_{i,j}|C_{1},C_{2})\left\{ \xi_{i,j}w_{i,j}\boldsymbol{IF}_{i,j}^{(3)}\left( \hat{\boldsymbol{\theta}} \right) \right\}\left\{ \xi_{i,j} w_{i,j}\boldsymbol{IF}_{i,j}^{(3)}\left( \hat{\boldsymbol{\theta}} \right) \right\}^{'}=\sum_{j=1}^{J} \sum_{i=1}^{n^{\left( j \right)}} \sigma_{i,j}^{(3)} \xi_{i,j}w_{i,j}w_{i,j}\boldsymbol{IF}_{i,j}^{(3)}\left( \hat{\boldsymbol{\theta}} \right){\boldsymbol{IF}_{i,j}^{(3)}\left( \hat{\boldsymbol{\theta}} \right)}^{\boldsymbol{'}}$,

as indeed $\xi_{i,j}\boldsymbol{IF}_{i,j}^{(2)}\left( \hat{\boldsymbol{\theta}} \right)$ is fixed conditional on $C_{2}$.

Then we have

$E\left[ E\left\{ \sum_{j=1}^{J} \sum_{i=1}^{n^{\left( j \right)}} \boldsymbol{\Delta}_{i,j}\left( \hat{\boldsymbol{\theta}} \right)|C_{1},C_{2} \right\}|C_{1} \right]=\sum_{j=1}^{J} \sum_{i=1}^{n^{\left( j \right)}} E\left( \xi_{i,j} |C_{1} \right)\left\{ \boldsymbol{IF}_{i,j}^{(2)}\left( \hat{\boldsymbol{\theta}} \right)+w_{i,j}^{\left( 2 \right)}\boldsymbol{IF}_{i,j}^{(3)}\left( \hat{\boldsymbol{\theta}} \right) \right\}$,

$=\sum_{j=1}^{J} \sum_{i=1}^{n^{\left( j \right)}} \left\{ \frac{1}{w_{i,j}^{\left( 2 \right)}}\boldsymbol{IF}_{i,j}^{(2)}\left( \hat{\boldsymbol{\theta}} \right)+\boldsymbol{IF}_{i,j}^{(3)}\left( \hat{\boldsymbol{\theta}} \right) \right\}$,

and

$\mathrm{var}\left[ E\left\{ \sum_{j=1}^{J} \sum_{i=1}^{n^{\left( j \right)}} \boldsymbol{\Delta}_{i,j}\left( \hat{\boldsymbol{\theta}} \right)|C_{1},C_{2} \right\}|C_{1} \right]=\sum_{j=1}^{J} \mathrm{var}\left[ \sum_{i=1}^{n^{\left( j \right)}} \xi_{i,j}\left\{ \boldsymbol{IF}_{i,j}^{(2)}\left( \hat{\boldsymbol{\theta}} \right)+w_{i,j}^{\left( 2 \right)}\boldsymbol{IF}_{i,j}^{(3)}\left( \hat{\boldsymbol{\theta}} \right) \right\} \right]$,

$=\sum_{j=1}^{J} \sum_{i=1}^{n^{\left( j \right)}} \sum_{k=1}^{n^{\left( j \right)}} \sigma_{i,k,j}^{(2)}\left\{ \boldsymbol{IF}_{i,j}^{(2)}\left( \hat{\boldsymbol{\theta}} \right)+w_{i,j}^{\left( 2 \right)}\boldsymbol{IF}_{i,j}^{(3)}\left( \hat{\boldsymbol{\theta}} \right) \right\}\left\{ \boldsymbol{IF}_{k,j}^{(2)}\left( \hat{\boldsymbol{\theta}} \right)+w_{k,j}^{\left( 2 \right)}\boldsymbol{IF}_{k,j}^{(3)}\left( \hat{\boldsymbol{\theta}} \right) \right\}^{'}$,

$=\sum_{j=1}^{J} \sum_{i=1}^{n^{\left( j \right)}} \sum_{k=1}^{n^{\left( j \right)}} \sigma_{i,k,j}^{(2)} w_{i,j}^{\left( 2 \right)}w_{k,j}^{\left( 2 \right)}\boldsymbol{IF}_{i,j}^{(3)}\left( \hat{\boldsymbol{\theta}} \right){\boldsymbol{IF}_{k,j}^{(3)}\left( \hat{\boldsymbol{\theta}} \right)}^{'}$,

as indeed any pair of individuals $i$ and $k$ in stratum $j$, $j\in\left\{ 1,\ldots,J \right\}$, such that $\sigma_{i,k,j}^{(2)}\neq0$ is necessarily such that $\boldsymbol{IF}_{i,j}^{(2)}\left( \hat{\boldsymbol{\theta}} \right)=\boldsymbol{IF}_{k,j}^{(3)}\left( \hat{\boldsymbol{\theta}} \right)=0$, and

$E\left[ \mathrm{var}\left\{ \sum_{j=1}^{J} \sum_{i=1}^{n^{\left( j \right)}} \boldsymbol{\Delta}_{i,j}\left( \hat{\boldsymbol{\theta}} \right)|C_{1},C_{2} \right\}|C_{1} \right]=\sum_{j=1}^{J} \sum_{i=1}^{n^{\left( j \right)}} E\left( \xi_{i,j}|C_{1} \right)\sigma_{i,j}^{(3)} w_{i,j} w_{i,j}\boldsymbol{IF}_{i,j}^{(3)}\left( \hat{\boldsymbol{\theta}} \right){\boldsymbol{IF}_{i,j}^{(3)}\left( \hat{\boldsymbol{\theta}} \right)}^{\boldsymbol{'}}$,

$=\sum_{j=1}^{J} \sum_{i=1}^{n^{\left( j \right)}} \sigma_{i,j}^{(3)}w_{i,j}^{\left( 3 \right)}w_{i,j} \boldsymbol{IF}_{i,j}^{(3)}\left( \hat{\boldsymbol{\theta}} \right){\boldsymbol{IF}_{i,j}^{(3)}\left( \hat{\boldsymbol{\theta}} \right)}^{\boldsymbol{'}}$.

Finally,

$\mathrm{var}\left( E\left[ E\left\{ \sum_{j=1}^{J} \sum_{i=1}^{n^{\left( j \right)}} \boldsymbol{\Delta}_{i,j}\left( \hat{\boldsymbol{\theta}} \right)|C_{1},C_{2} \right\}|C_{1} \right] \right)=\mathrm{var}\left[ \sum_{j=1}^{J} \sum_{i=1}^{n^{\left( j \right)}} \frac{1}{w_{i,j}^{\left( 2 \right)}}\boldsymbol{IF}_{i,j}^{(2)}\left( \hat{\boldsymbol{\theta}} \right)+\boldsymbol{IF}_{i,j}^{(3)}\left( \hat{\boldsymbol{\theta}} \right) \right]$,

and can be estimated by

$\frac{n}{n-1}\sum_{j=1}^{J} \sum_{i=1}^{n^{\left( j \right)}} \left\{ \xi_{i,j}\frac{1}{w_{i,j}^{\left( 2 \right)}}\boldsymbol{IF}_{i,j}^{(2)}\left( \hat{\boldsymbol{\theta}} \right){\boldsymbol{IF}_{i,j}^{(2)}\left( \hat{\boldsymbol{\theta}} \right)}^{\boldsymbol{'}}+2 \xi_{i,j}V_{i,j} \frac{w_{i,j}}{w_{i,j}^{\left( 2 \right)}}\boldsymbol{IF}_{i,j}^{(2)}\left( \hat{\boldsymbol{\theta}} \right){\boldsymbol{IF}_{i,j}^{(3)}\left( \hat{\boldsymbol{\theta}} \right)}^{\boldsymbol{'}}+\xi_{i,j}V_{i,j} w_{i,j}\boldsymbol{IF}_{i,j}^{(3)}\left( \hat{\boldsymbol{\theta}} \right){\boldsymbol{IF}_{i,j}^{(3)}\left( \hat{\boldsymbol{\theta}} \right)}^{\boldsymbol{'}} \right\}$,

that is by

$\frac{n}{n-1}\sum_{j=1}^{J} \sum_{i=1}^{n^{\left( j \right)}} \left\{ \frac{1}{w_{i,j}^{\left( 2 \right)}}\xi_{i,j}\boldsymbol{IF}_{i,j}^{(2)}\left( \hat{\boldsymbol{\theta}} \right){\boldsymbol{IF}_{i,j}^{(2)}\left( \hat{\boldsymbol{\theta}} \right)}^{\boldsymbol{'}}+2\frac{1}{w_{i,j}^{\left( 2 \right)}} \xi_{i,j}V_{i,j} w_{i,j}\boldsymbol{IF}_{i,j}^{(2)}\left( \hat{\boldsymbol{\theta}} \right){\boldsymbol{IF}_{i,j}^{(3)}\left( \hat{\boldsymbol{\theta}} \right)}^{\boldsymbol{'}}+\frac{1}{w_{i,j}}\xi_{i,j}V_{i,j} w_{i,j}w_{i,j}\boldsymbol{IF}_{i,j}^{(3)}\left( \hat{\boldsymbol{\theta}} \right){\boldsymbol{IF}_{i,j}^{(3)}\left( \hat{\boldsymbol{\theta}} \right)}^{\boldsymbol{'}} \right\}$,

and

$E\left( \mathrm{var}\left[ E\left\{ \sum_{j=1}^{J} \sum_{i=1}^{n^{\left( j \right)}} \boldsymbol{\Delta}_{i,j}\left( \hat{\boldsymbol{\theta}} \right)|C_{1},C_{2} \right\}|C_{1} \right] \right)=E\left[ \sum_{j=1}^{J} \sum_{i=1}^{n^{\left( j \right)}} \sum_{k=1}^{n^{\left( j \right)}} \sigma_{i,k,j}^{(2)} w_{i,j}^{\left( 2 \right)}w_{k,j}^{\left( 2 \right)}\boldsymbol{IF}_{i,j}^{(3)}\left( \hat{\boldsymbol{\theta}} \right){\boldsymbol{IF}_{k,j}^{(3)}\left( \hat{\boldsymbol{\theta}} \right)}^{'} \right]$,

that can be estimated by

$\sum_{j=1}^{J} \sum_{i=1}^{n^{\left( j \right)}} \sum_{k=1}^{n^{\left( j \right)}} \sigma_{i,k,j}^{(2)} w_{i,k,j} \xi_{i,j}\xi_{k,j}V_{i,j} V_{k,j}w_{i,j}^{\left( 2 \right)}w_{k,j}^{\left( 2 \right)}\boldsymbol{IF}_{i,j}^{(3)}\left( \hat{\boldsymbol{\theta}} \right){\boldsymbol{IF}_{k,j}^{(3)}\left( \hat{\boldsymbol{\theta}} \right)}^{'}$,

that is by$\sum_{j=1}^{J} \sum_{i=1}^{n^{\left( j \right)}} \sigma_{i,j}^{(2)}\frac{w_{i,j}^{\left( 2 \right)}}{w_{i,j}^{\left( 3 \right)}}\xi_{i,j}V_{i,j} w_{i,j}w_{i,j}\boldsymbol{IF}_{i,j}^{(3)}\left( \hat{\boldsymbol{\theta}} \right){\boldsymbol{IF}_{i,j}^{(3)}\left( \hat{\boldsymbol{\theta}} \right)}^{'}+\sum_{j=1}^{J} \sum_{i=1}^{n^{\left( j \right)}} \sum_{\begin{aligned} k=1, \\ k\neq i \end{aligned}}^{n^{\left( j \right)}} \sigma_{i,k,j}^{(2)} w_{i,k,j}^{\left( 2 \right)} \xi_{i,j}V_{i,j} \xi_{k,j}V_{k,j} w_{i,j}w_{k,j}\boldsymbol{IF}_{i,j}^{(3)}\left( \hat{\boldsymbol{\theta}} \right){\boldsymbol{IF}_{i,j}^{(3)}\left( \hat{\boldsymbol{\theta}} \right)}^{'}$,

and $E\left( E\left[ \mathrm{var}\left\{ \sum_{j=1}^{J} \sum_{i=1}^{n^{\left( j \right)}} \boldsymbol{\Delta}_{i,j}\left( \hat{\boldsymbol{\theta}} \right)|C_{1},C_{2} \right\}|C_{1} \right] \right)=E\left\{ \sum_{j=1}^{J} \sum_{i=1}^{n^{\left( j \right)}} \sigma_{i,j}^{(3)}w_{i,j}^{\left( 3 \right)}w_{i,j} \boldsymbol{IF}_{i,j}^{(3)}\left( \hat{\boldsymbol{\theta}} \right){\boldsymbol{IF}_{i,j}^{(3)}\left( \hat{\boldsymbol{\theta}} \right)}^{\boldsymbol{'}} \right\}$, that can be estimated by $\sum_{j=1}^{J} \sum_{i=1}^{n^{\left( j \right)}} \xi_{i,j}V_{i,j} w_{i,j}\sigma_{i,j}^{(3)} w_{i,j}^{\left( 3 \right)}w_{i,j}\boldsymbol{IF}_{i,j}^{(3)}\left( \hat{\boldsymbol{\theta}} \right){\boldsymbol{IF}_{i,j}^{(3)}\left( \hat{\boldsymbol{\theta}} \right)}^{\boldsymbol{'}}$, that is by $\sum_{j=1}^{J} \sum_{i=1}^{n^{\left( j \right)}} \sigma_{i,j}^{(3)}w_{i,j}^{\left( 3 \right)}\xi_{i,j}V_{i,j} w_{i,j}w_{i,j}\boldsymbol{IF}_{i,j}^{(3)}\left( \hat{\boldsymbol{\theta}} \right){\boldsymbol{IF}_{i,j}^{(3)}\left( \hat{\boldsymbol{\theta}} \right)}^{\boldsymbol{'}}$.

As a result, $\mathrm{var}\left( \hat{\boldsymbol{\theta}} \right)$ can be estimated by

$\frac{n}{n-1}\sum_{j=1}^{J} \sum_{i=1}^{n^{\left( j \right)}} \left\{ \frac{1}{w_{i,j}^{\left( 2 \right)}}\xi_{i,j}\boldsymbol{IF}_{i,j}^{(2)}\left( \hat{\boldsymbol{\theta}} \right){\boldsymbol{IF}_{i,j}^{(2)}\left( \hat{\boldsymbol{\theta}} \right)}^{\boldsymbol{'}}+2\frac{1}{w_{i,j}^{\left( 2 \right)}} \xi_{i,j}V_{i,j} w_{i,j}\boldsymbol{IF}_{i,j}^{(2)}\left( \hat{\boldsymbol{\theta}} \right){\boldsymbol{IF}_{i,j}^{(3)}\left( \hat{\boldsymbol{\theta}} \right)}^{\boldsymbol{'}}+\frac{1}{w_{i,j}}\xi_{i,j}V_{i,j} w_{i,j}w_{i,j}\boldsymbol{IF}_{i,j}^{(3)}\left( \hat{\boldsymbol{\theta}} \right){\boldsymbol{IF}_{i,j}^{(3)}\left( \hat{\boldsymbol{\theta}} \right)}^{\boldsymbol{'}} \right\}+\sum_{j=1}^{J} \sum_{i=1}^{n^{\left( j \right)}} \sigma_{i,j}^{(2)}\frac{w_{i,j}^{\left( 2 \right)}}{w_{i,j}^{\left( 3 \right)}}\xi_{i,j}V_{i,j} w_{i,j}w_{i,j}\boldsymbol{IF}_{i,j}^{(3)}\left( \hat{\boldsymbol{\theta}} \right){\boldsymbol{IF}_{i,j}^{(3)}\left( \hat{\boldsymbol{\theta}} \right)}^{'}+\sum_{j=1}^{J} \sum_{i=1}^{n^{\left( j \right)}} \sum_{\begin{aligned} k=1, \\ k\neq i \end{aligned}}^{n^{\left( j \right)}} \sigma_{i,k,j}^{(2)} w_{i,k,j}^{\left( 2 \right)} \xi_{i,j}V_{i,j} \xi_{k,j}V_{k,j} w_{i,j}w_{k,j}\boldsymbol{IF}_{i,j}^{(3)}\left( \hat{\boldsymbol{\theta}} \right){\boldsymbol{IF}_{i,j}^{(3)}\left( \hat{\boldsymbol{\theta}} \right)}^{'}+\sum_{j=1}^{J} \sum_{i=1}^{n^{\left( j \right)}} \sigma_{i,j}^{(3)}w_{i,j}^{\left( 3 \right)}\xi_{i,j}V_{i,j} w_{i,j}w_{i,j}\boldsymbol{IF}_{i,j}^{(3)}\left( \hat{\boldsymbol{\theta}} \right){\boldsymbol{IF}_{i,j}^{(3)}\left( \hat{\boldsymbol{\theta}} \right)}^{\boldsymbol{'}}$,

that we can rewrite

| $\frac{n}{n-1}\sum_{j=1}^{J} \sum_{i=1}^{n^{\left( j \right)}} \left\{ \frac{1}{w_{i,j}^{\left( 2 \right)}}\xi_{i,j}\boldsymbol{IF}_{i,j}^{(2)}\left( \hat{\boldsymbol{\theta}} \right){\boldsymbol{IF}_{i,j}^{(2)}\left( \hat{\boldsymbol{\theta}} \right)}^{\boldsymbol{'}}+2\frac{1}{w_{i,j}^{\left( 2 \right)}} \xi_{i,j}V_{i,j} w_{i,j}\boldsymbol{IF}_{i,j}^{(2)}\left( \hat{\boldsymbol{\theta}} \right){\boldsymbol{IF}_{i,j}^{(3)}\left( \hat{\boldsymbol{\theta}} \right)}^{\boldsymbol{'}}+\frac{1}{w_{i,j}}\xi_{i,j}V_{i,j} w_{i,j}w_{i,j}\boldsymbol{IF}_{i,j}^{(3)}\left( \hat{\boldsymbol{\theta}} \right){\boldsymbol{IF}_{i,j}^{(3)}\left( \hat{\boldsymbol{\theta}} \right)}^{\boldsymbol{'}} \right\}+\sum_{j=1}^{J} \sum_{i=1}^{n^{\left( j \right)}} \sum_{k=1}^{n^{\left( j \right)}} \sigma_{i,k,j} w_{i,k,j} \xi_{i,j}V_{i,j} \xi_{k,j}V_{k,j} w_{i,j}w_{k,j}\boldsymbol{IF}_{i,j}^{(3)}\left( \hat{\boldsymbol{\theta}} \right){\boldsymbol{IF}_{i,j}^{(3)}\left( \hat{\boldsymbol{\theta}} \right)}^{'}$, | (4) |
| --- | --- |

as indeed $\sigma_{i,j}^{(2)}\frac{w_{i,j}^{\left( 2 \right)}}{w_{i,j}^{\left( 3 \right)}}+\sigma_{i,j}^{(3)}w_{i,j}^{\left( 3 \right)}=1-\frac{1}{w_{i,j}}=\sigma_{i,j} w_{i,j}$, and $\sigma_{i,k,j}^{\left( 2 \right)}w_{i,k,j}^{\left( 2 \right)}=\sigma_{i,k,j}^{\left( 2 \right)}\times\frac{1}{w_{i,j}^{\left( 3 \right)}}\times\frac{1}{w_{k,j}^{\left( 3 \right)}}\times w_{i,k,j}^{\left( 2 \right)}\times w_{i,j}^{\left( 3 \right)}\times w_{k,j}^{\left( 3 \right)}=\sigma_{i,k,j}w_{i,k,j}$, $i,k\in\{1,\ldots,n^{(j)}\}$, $i\neq k$,$j\in\left\{ 1,\ldots,J \right\}$.

Note, when we assume that the $w_{i}^{\left( 3 \right)}$are known, and because the third phase of sampling is Bernoulli, we could collapse the phase-two and phase-three into a single sampling phase. The variance estimate given above in Equation (4) and written with only two components, corresponds to the form we would obtain directly when the second and third phases of sampling are collapsed into a single phase of sampling.

# SIMULATIONS WITH MISSING PHASE-TWO DATA

## Simulation design

We simulated cohorts with $n\in\left\{ 5\times{10}^{3},{10}^{4} \right\}$ as in Section 7 in the Main Document. We sampled independently across the $J=4$ strata, defined by $W=0\times I\left( X_{1}\geq0,X_{2}=0 \right)+1\times I\left( X_{1}<0,X_{2}<2 \right)+2\times I\left( X_{1}\geq0,X_{2}>0 \right)+3\times I\left( X_{1}<0,X_{2}=2 \right)$, fixed numbers of individuals, $m^{(j)} =\left\lfloor\frac{\lambda_{0}\times10\times E\left\{ \exp(\beta_{1}X_{1}+\beta_{2}X_{2}+\beta_{3}X_{3})|W=j \right\}}{1- \lambda_{0}\times10\times E\left\{ \exp(\beta_{1}X_{1}+\beta_{2}X_{2}+\beta_{3}X_{3})|W=j \right\}}\times E\left( n^{(j)} \right)\times K+\frac{1}{2} \right\rfloor$, with $K\in\left\{ 2, 4 \right\}$, $j\in\{0,1,2,3\}$, and where $\left\lfloor\right\rfloor$ is the floor function. These individuals, in addition to the remaining cases, constituted the phase-two sample. In parallel, we sampled individuals from the $J^{(3)}=2$ strata defined by case status, with phase-three sampling probabilities $\boldsymbol{\pi}^{\left( 3 \right)}\in\left\{ \left( 0.9, 0.8 \right),\left( 0.98, 0.9 \right) \right\}$. In other words, we assumed that cases always had a higher probability of missing covariate information, for example due to stored blood samples being previously used. Finally, we regarded the individuals who were in both samples, i.e., individuals with complete covariate data, as the phase-three sample.

Phase-two sampling design weights were computed as in Section 3.3 in the Main Document, and phase-three sampling design weights were given by the inverse of the phase-three sampling probabilities. The overall sampling designs weights were then obtained from their product. However, in practice, the phase-three sampling design weights, $\frac{\boldsymbol{1}}{\boldsymbol{\pi}^{\left( 3 \right)}}$, are usually unknown. We thus estimated the phase-three sampling design weights as in Section 5.2 in the Main Document; the overall estimated sampling designs weights were then obtained from the product with the known phase-two sampling design weights.

For each scenario, we simulated 5,000 cohorts. We estimated the log-relative hazard $\boldsymbol{\beta=}\left( \beta_{1},\beta_{2},\beta_{3} \right)\boldsymbol{'}$ and pure risks $\pi\left( \tau_{1},\tau_{2}; \boldsymbol{x} \right)$ in time interval ${(\tau}_{1},\tau_{2}]=(0,8]$ and for covariate profiles $\boldsymbol{x}\in\left\{ \left( -1, 1, -0.6 \right)',\left( 1, -1, 0.6 \right)',\left( 1, 1, 0.6 \right)' \right\}$, using: the stratified case-cohort with estimated design weights (SCC.Est); the stratified case-cohort with true design weights (SCC.True); the unstratified case-cohort with estimated design weights (USCC.Est); and the unstratified case-cohort with true design weights (USCC.True). For each simulated realization, we then estimated their variances. For SCC.Est, we used: the variance estimate with superpopulation, phase-two and phase-three variance components ($\hat{V}$) from Equation (22) in Section 5.5 the Main Document or in Web Appendix E.2; and the robust variance estimate ($\hat{V}_{\mathrm{Robust}}$) computed as in Section 3.3 or Section 4.3 in the Main Document but with the influences given in Section 5.4 in the Main document or in Web Appendix E.1. For SCC.True, we used: the variance estimate with superpopulation, phase-two and phase-three variance components ($\hat{V}$) from Equation (4) in Web Appendix F.3; and $\hat{V}_{\mathrm{Robust}}$ computed as in Section 3.3 or Section 4.3 in the Main Document but with the influences given in Web Appendix F.2. For USCC.Est and USCC.True, we used similar variance estimates, with $J = 1$. An alternative was also to treat the estimated design weights as if they were the true known ones, and to estimate the variance as in Web Appendix F.3 (SCC.Naive and USCC.Naive). As a point of reference, we also estimated these parameters using the data from the whole cohort (Cohort).

## Simulation results

**WEB TABLE 31** to **WEB TABLE 42** display the coverages of 95% CIs, and **WEB TABLE 43** to **WEB TABLE 54** display the mean of estimated variances and empirical variances, for $\beta_{1}$, $\beta_{2}$, $\beta_{3}$ and $\log\left\{ \pi\left( \tau_{1},\tau_{2}; \boldsymbol{x} \right) \right\}$, ${(\tau}_{1},\tau_{2}]=(0,8]$ and $\boldsymbol{x}\in\left\{ \left( -1, 1, -0.6 \right)',\left( 1, -1, 0.6 \right)',\left( 1, 1, 0.6 \right)' \right\}$, respectively. For brevity, we left out results for $n=$ 10,000 and $p_{Y}=$ 0.1. Again, the robust variance overestimated the variance and yielded supra-nominal confidence interval coverage for log-relative hazards and pure risks with stratified designs in most of the scenarios, and for pure risk with unstratified designs in most of the scenarios. On the other hand, variance estimation properly accounting for the sampling features yielded proper coverage in most of the scenarios. Using the estimated design weights led to similar performance as using the true design weights. In particular, in the present simulation, treating the estimated weights as if they were the true known ones, and using the simpler variance formula given in Web Appendix F.3, led to similar performance as using the more complex influence-based variance given in Equation (22) in the Main Document. However, this was not the case in non-reported simulations with larger proportions missing. We therefore recommend accounting for the variability from estimation of the phase-three weights as in Equation (22) in the Main Document. Using estimated weights in SCC.Est and SCC.Naive (respectively USCC.Est and USCC.Naive) yielded negligible, if any, gain of efficiency compared to SCC.True (respectively USCC.True) in the present simulations.

| Cohort | SCC.True | | SCC.Est | | SCC.Naive | | $n$ | $K$ | $p_{Y}$ | $\boldsymbol{\pi}^{(3)}$ | $\beta_{1}$ |
| --- | --- | --- | --- | --- | --- | --- | --- | --- | --- | --- | --- |
|  | $\hat{V}_{\mathrm{Robust}}$ | $\hat{V}$ | $\hat{V}_{\mathrm{Robust}}$ | $\hat{V}$ | $\hat{V}_{\mathrm{Robust}}$ | $\hat{V}$ |  |  |  |  |  |
| 0.9504 | 0.969* | 0.9574* | 0.969* | 0.9574* | 0.969* | 0.9574* | 5000 | 2 | 0.02 | (0.9,0.8) | -0.2 |
| 0.9504 | 0.9694* | 0.9554 | 0.9694* | 0.9554 | 0.9694* | 0.9554 | 5000 | 2 | 0.02 | (0.98,0.9) | -0.2 |
| 0.943* | 0.9534 | 0.9474 | 0.9536 | 0.9474 | 0.9536 | 0.9474 | 5000 | 4 | 0.02 | (0.9,0.8) | -0.2 |
| 0.943* | 0.9568* | 0.948 | 0.957* | 0.948 | 0.957* | 0.948 | 5000 | 4 | 0.02 | (0.98,0.9) | -0.2 |
| 0.9478 | 0.9652* | 0.953 | 0.9652* | 0.953 | 0.9652* | 0.953 | 10000 | 2 | 0.02 | (0.9,0.8) | -0.2 |
| 0.9478 | 0.9664* | 0.9534 | 0.9662* | 0.9534 | 0.9662* | 0.9534 | 10000 | 2 | 0.02 | (0.98,0.9) | -0.2 |
| 0.9482 | 0.9542 | 0.9462 | 0.9542 | 0.9462 | 0.9542 | 0.9462 | 10000 | 4 | 0.02 | (0.9,0.8) | -0.2 |
| 0.9482 | 0.9572* | 0.9474 | 0.9572* | 0.9474 | 0.9572* | 0.9474 | 10000 | 4 | 0.02 | (0.98,0.9) | -0.2 |
| 0.9486 | 0.9638* | 0.9548 | 0.9638* | 0.9546 | 0.9638* | 0.9546 | 5000 | 2 | 0.05 | (0.9,0.8) | -0.2 |
| 0.9486 | 0.96* | 0.9472 | 0.96* | 0.947 | 0.96* | 0.947 | 5000 | 2 | 0.05 | (0.98,0.9) | -0.2 |
| 0.9462 | 0.9506 | 0.944 | 0.9506 | 0.9442 | 0.9506 | 0.9442 | 5000 | 4 | 0.05 | (0.9,0.8) | -0.2 |
| 0.9462 | 0.955 | 0.9474 | 0.9548 | 0.9476 | 0.9548 | 0.9476 | 5000 | 4 | 0.05 | (0.98,0.9) | -0.2 |
| 0.9486 | 0.9654* | 0.9536 | 0.9654* | 0.9536 | 0.9654* | 0.9536 | 10000 | 2 | 0.05 | (0.9,0.8) | -0.2 |
| 0.9486 | 0.966* | 0.9498 | 0.966* | 0.95 | 0.966* | 0.95 | 10000 | 2 | 0.05 | (0.98,0.9) | -0.2 |
| 0.948 | 0.9554 | 0.9496 | 0.9554 | 0.9496 | 0.9554 | 0.9496 | 10000 | 4 | 0.05 | (0.9,0.8) | -0.2 |
| 0.948 | 0.9594* | 0.9514 | 0.9596* | 0.9514 | 0.9596* | 0.9514 | 10000 | 4 | 0.05 | (0.98,0.9) | -0.2 |
| 0.949 | 0.9574* | 0.947 | 0.9574* | 0.9474 | 0.9574* | 0.9474 | 5000 | 2 | 0.1 | (0.9,0.8) | -0.2 |
| 0.949 | 0.9592* | 0.9484 | 0.9594* | 0.9484 | 0.9594* | 0.9484 | 5000 | 2 | 0.1 | (0.98,0.9) | -0.2 |
| 0.9494 | 0.952 | 0.9486 | 0.9518 | 0.9484 | 0.9518 | 0.9484 | 5000 | 4 | 0.1 | (0.9,0.8) | -0.2 |
| 0.9494 | 0.954 | 0.9494 | 0.9536 | 0.9494 | 0.9536 | 0.9494 | 5000 | 4 | 0.1 | (0.98,0.9) | -0.2 |

**WEB TABLE 31-** Coverage of 95% CIs for log-relative hazard parameter $\beta_{1}$ from stratified sampling using different methods of analysis and variance estimation, for various probabilities of missing covariate data, in 5,000 simulated cohorts. * indicates coverage outside the expected interval [0.9440; 0.9560]

| Cohort | USCC.True | | USCC.Est | | USCC.Naive | | $n$ | $K$ | $p_{Y}$ | $\boldsymbol{\pi}^{(3)}$ | $\beta_{1}$ |
| --- | --- | --- | --- | --- | --- | --- | --- | --- | --- | --- | --- |
|  | $\hat{V}_{\mathrm{Robust}}$ | $\hat{V}$ | $\hat{V}_{\mathrm{Robust}}$ | $\hat{V}$ | $\hat{V}_{\mathrm{Robust}}$ | $\hat{V}$ |  |  |  |  |  |
| 0.9504 | 0.9576* | 0.9576* | 0.9576* | 0.9578* | 0.9576* | 0.9578* | 5000 | 2 | 0.02 | (0.9,0.8) | -0.2 |
| 0.9504 | 0.9564* | 0.9566* | 0.956 | 0.9566* | 0.956 | 0.9566* | 5000 | 2 | 0.02 | (0.98,0.9) | -0.2 |
| 0.943* | 0.9514 | 0.9514 | 0.9514 | 0.9514 | 0.9514 | 0.9514 | 5000 | 4 | 0.02 | (0.9,0.8) | -0.2 |
| 0.943* | 0.9486 | 0.9486 | 0.9486 | 0.9486 | 0.9486 | 0.9486 | 5000 | 4 | 0.02 | (0.98,0.9) | -0.2 |
| 0.9478 | 0.9542 | 0.9542 | 0.9542 | 0.9542 | 0.9542 | 0.9542 | 10000 | 2 | 0.02 | (0.9,0.8) | -0.2 |
| 0.9478 | 0.9512 | 0.9512 | 0.9512 | 0.9512 | 0.9512 | 0.9512 | 10000 | 2 | 0.02 | (0.98,0.9) | -0.2 |
| 0.9482 | 0.9458 | 0.9458 | 0.9458 | 0.946 | 0.9458 | 0.946 | 10000 | 4 | 0.02 | (0.9,0.8) | -0.2 |
| 0.9482 | 0.948 | 0.948 | 0.948 | 0.948 | 0.948 | 0.948 | 10000 | 4 | 0.02 | (0.98,0.9) | -0.2 |
| 0.9486 | 0.9504 | 0.9504 | 0.9506 | 0.9508 | 0.9506 | 0.9508 | 5000 | 2 | 0.05 | (0.9,0.8) | -0.2 |
| 0.9486 | 0.9506 | 0.9506 | 0.9506 | 0.9506 | 0.9506 | 0.9506 | 5000 | 2 | 0.05 | (0.98,0.9) | -0.2 |
| 0.9462 | 0.9492 | 0.9492 | 0.9492 | 0.9492 | 0.9492 | 0.9492 | 5000 | 4 | 0.05 | (0.9,0.8) | -0.2 |
| 0.9462 | 0.9472 | 0.9472 | 0.9472 | 0.9472 | 0.9472 | 0.9472 | 5000 | 4 | 0.05 | (0.98,0.9) | -0.2 |
| 0.9486 | 0.9474 | 0.9476 | 0.9476 | 0.9476 | 0.9476 | 0.9476 | 10000 | 2 | 0.05 | (0.9,0.8) | -0.2 |
| 0.9486 | 0.9502 | 0.9504 | 0.9502 | 0.9504 | 0.9502 | 0.9504 | 10000 | 2 | 0.05 | (0.98,0.9) | -0.2 |
| 0.948 | 0.9514 | 0.9514 | 0.9512 | 0.9512 | 0.9512 | 0.9512 | 10000 | 4 | 0.05 | (0.9,0.8) | -0.2 |
| 0.948 | 0.95 | 0.95 | 0.95 | 0.95 | 0.95 | 0.95 | 10000 | 4 | 0.05 | (0.98,0.9) | -0.2 |
| 0.949 | 0.9506 | 0.9506 | 0.9508 | 0.9508 | 0.9508 | 0.9508 | 5000 | 2 | 0.1 | (0.9,0.8) | -0.2 |
| 0.949 | 0.95 | 0.95 | 0.9498 | 0.9498 | 0.9498 | 0.9498 | 5000 | 2 | 0.1 | (0.98,0.9) | -0.2 |
| 0.9494 | 0.9482 | 0.9482 | 0.9484 | 0.9484 | 0.9484 | 0.9484 | 5000 | 4 | 0.1 | (0.9,0.8) | -0.2 |
| 0.9494 | 0.9496 | 0.9496 | 0.9496 | 0.9496 | 0.9496 | 0.9496 | 5000 | 4 | 0.1 | (0.98,0.9) | -0.2 |

**WEB TABLE 32-** Coverage of 95% CIs for log-relative hazard parameter $\beta_{1}$ from unstratified sampling using different methods of analysis and variance estimation, for various probabilities of missing covariate data, in 5,000 simulated cohorts. * indicates coverage outside the expected interval [0.9440; 0.9560]

| Cohort | SCC.True | | SCC.Est | | SCC.Naive | | $n$ | $K$ | $p_{Y}$ | $\boldsymbol{\pi}^{(3)}$ | $\beta_{2}$ |
| --- | --- | --- | --- | --- | --- | --- | --- | --- | --- | --- | --- |
|  | $\hat{V}_{\mathrm{Robust}}$ | $\hat{V}$ | $\hat{V}_{\mathrm{Robust}}$ | $\hat{V}$ | $\hat{V}_{\mathrm{Robust}}$ | $\hat{V}$ |  |  |  |  |  |
| 0.9464 | 0.9632* | 0.9486 | 0.9634* | 0.9488 | 0.9634* | 0.9488 | 5000 | 2 | 0.02 | (0.9,0.8) | 0.25 |
| 0.9464 | 0.9662* | 0.947 | 0.9662* | 0.9468 | 0.9662* | 0.947 | 5000 | 2 | 0.02 | (0.98,0.9) | 0.25 |
| 0.9508 | 0.9614* | 0.9524 | 0.9614* | 0.9524 | 0.9614* | 0.9524 | 5000 | 4 | 0.02 | (0.9,0.8) | 0.25 |
| 0.9508 | 0.9614* | 0.95 | 0.9614* | 0.95 | 0.9614* | 0.95 | 5000 | 4 | 0.02 | (0.98,0.9) | 0.25 |
| 0.9474 | 0.9676* | 0.9502 | 0.9676* | 0.9502 | 0.9676* | 0.9502 | 10000 | 2 | 0.02 | (0.9,0.8) | 0.25 |
| 0.9474 | 0.9682* | 0.9512 | 0.9682* | 0.9512 | 0.9682* | 0.9512 | 10000 | 2 | 0.02 | (0.98,0.9) | 0.25 |
| 0.9488 | 0.9612* | 0.949 | 0.961* | 0.949 | 0.961* | 0.949 | 10000 | 4 | 0.02 | (0.9,0.8) | 0.25 |
| 0.9488 | 0.9634* | 0.951 | 0.9636* | 0.9512 | 0.9636* | 0.9512 | 10000 | 4 | 0.02 | (0.98,0.9) | 0.25 |
| 0.9508 | 0.9658* | 0.9542 | 0.9658* | 0.9546 | 0.9658* | 0.9546 | 5000 | 2 | 0.05 | (0.9,0.8) | 0.25 |
| 0.9508 | 0.9714* | 0.9574* | 0.9712* | 0.9574* | 0.9712* | 0.9574* | 5000 | 2 | 0.05 | (0.98,0.9) | 0.25 |
| 0.9438* | 0.9546 | 0.9466 | 0.9546 | 0.9464 | 0.9546 | 0.9464 | 5000 | 4 | 0.05 | (0.9,0.8) | 0.25 |
| 0.9438* | 0.9556 | 0.9474 | 0.9556 | 0.9478 | 0.9556 | 0.9478 | 5000 | 4 | 0.05 | (0.98,0.9) | 0.25 |
| 0.95 | 0.9594* | 0.9464 | 0.9594* | 0.9464 | 0.9594* | 0.9464 | 10000 | 2 | 0.05 | (0.9,0.8) | 0.25 |
| 0.95 | 0.9652* | 0.9506 | 0.965* | 0.9506 | 0.965* | 0.9506 | 10000 | 2 | 0.05 | (0.98,0.9) | 0.25 |
| 0.9458 | 0.959* | 0.9506 | 0.9588* | 0.9504 | 0.9588* | 0.9504 | 10000 | 4 | 0.05 | (0.9,0.8) | 0.25 |
| 0.9458 | 0.9584* | 0.9498 | 0.9582* | 0.9498 | 0.9582* | 0.9498 | 10000 | 4 | 0.05 | (0.98,0.9) | 0.25 |
| 0.9492 | 0.963* | 0.9492 | 0.9628* | 0.9488 | 0.9628* | 0.9488 | 5000 | 2 | 0.1 | (0.9,0.8) | 0.25 |
| 0.9492 | 0.9664* | 0.9514 | 0.9664* | 0.9514 | 0.9664* | 0.9514 | 5000 | 2 | 0.1 | (0.98,0.9) | 0.25 |
| 0.954 | 0.954 | 0.9494 | 0.9542 | 0.9496 | 0.9542 | 0.9496 | 5000 | 4 | 0.1 | (0.9,0.8) | 0.25 |
| 0.954 | 0.9552 | 0.9496 | 0.9554 | 0.9494 | 0.9556 | 0.9494 | 5000 | 4 | 0.1 | (0.98,0.9) | 0.25 |

**WEB TABLE 33-** Coverage of 95% CIs for log-relative hazard parameter $\beta_{2}$ from stratified sampling using different methods of analysis and variance estimation, for various probabilities of missing covariate data, in 5,000 simulated cohorts. * indicates coverage outside the expected interval [0.9440; 0.9560]

| Cohort | USCC.True | | USCC.Est | | USCC.Naive | | $n$ | $K$ | $p_{Y}$ | $\boldsymbol{\pi}^{(3)}$ | $\beta_{2}$ |
| --- | --- | --- | --- | --- | --- | --- | --- | --- | --- | --- | --- |
|  | $\hat{V}_{\mathrm{Robust}}$ | $\hat{V}$ | $\hat{V}_{\mathrm{Robust}}$ | $\hat{V}$ | $\hat{V}_{\mathrm{Robust}}$ | $\hat{V}$ |  |  |  |  |  |
| 0.9464 | 0.9484 | 0.9486 | 0.9484 | 0.9486 | 0.9484 | 0.9486 | 5000 | 2 | 0.02 | (0.9,0.8) | 0.25 |
| 0.9464 | 0.95 | 0.9506 | 0.95 | 0.9506 | 0.95 | 0.9506 | 5000 | 2 | 0.02 | (0.98,0.9) | 0.25 |
| 0.9508 | 0.9518 | 0.9518 | 0.9518 | 0.9518 | 0.9518 | 0.9518 | 5000 | 4 | 0.02 | (0.9,0.8) | 0.25 |
| 0.9508 | 0.9534 | 0.9534 | 0.9534 | 0.9534 | 0.9534 | 0.9534 | 5000 | 4 | 0.02 | (0.98,0.9) | 0.25 |
| 0.9474 | 0.9512 | 0.9512 | 0.9512 | 0.9512 | 0.9512 | 0.9512 | 10000 | 2 | 0.02 | (0.9,0.8) | 0.25 |
| 0.9474 | 0.9486 | 0.9486 | 0.9486 | 0.9488 | 0.9486 | 0.9488 | 10000 | 2 | 0.02 | (0.98,0.9) | 0.25 |
| 0.9488 | 0.951 | 0.951 | 0.9508 | 0.951 | 0.9508 | 0.951 | 10000 | 4 | 0.02 | (0.9,0.8) | 0.25 |
| 0.9488 | 0.9532 | 0.9532 | 0.953 | 0.9532 | 0.953 | 0.9532 | 10000 | 4 | 0.02 | (0.98,0.9) | 0.25 |
| 0.9508 | 0.9514 | 0.9516 | 0.9518 | 0.952 | 0.9518 | 0.952 | 5000 | 2 | 0.05 | (0.9,0.8) | 0.25 |
| 0.9508 | 0.9538 | 0.954 | 0.9538 | 0.9538 | 0.9538 | 0.9538 | 5000 | 2 | 0.05 | (0.98,0.9) | 0.25 |
| 0.9438* | 0.9472 | 0.9472 | 0.947 | 0.947 | 0.947 | 0.947 | 5000 | 4 | 0.05 | (0.9,0.8) | 0.25 |
| 0.9438* | 0.945 | 0.9452 | 0.9454 | 0.9454 | 0.9454 | 0.9454 | 5000 | 4 | 0.05 | (0.98,0.9) | 0.25 |
| 0.95 | 0.95 | 0.95 | 0.95 | 0.95 | 0.95 | 0.95 | 10000 | 2 | 0.05 | (0.9,0.8) | 0.25 |
| 0.95 | 0.9482 | 0.9482 | 0.9482 | 0.9482 | 0.9482 | 0.9482 | 10000 | 2 | 0.05 | (0.98,0.9) | 0.25 |
| 0.9458 | 0.954 | 0.954 | 0.9538 | 0.9538 | 0.9538 | 0.9538 | 10000 | 4 | 0.05 | (0.9,0.8) | 0.25 |
| 0.9458 | 0.9522 | 0.9522 | 0.952 | 0.952 | 0.952 | 0.952 | 10000 | 4 | 0.05 | (0.98,0.9) | 0.25 |
| 0.9492 | 0.9488 | 0.949 | 0.9488 | 0.9488 | 0.9488 | 0.9488 | 5000 | 2 | 0.1 | (0.9,0.8) | 0.25 |
| 0.9492 | 0.9504 | 0.9504 | 0.9504 | 0.9504 | 0.9504 | 0.9504 | 5000 | 2 | 0.1 | (0.98,0.9) | 0.25 |
| 0.954 | 0.9494 | 0.9494 | 0.9492 | 0.9492 | 0.9492 | 0.9492 | 5000 | 4 | 0.1 | (0.9,0.8) | 0.25 |
| 0.954 | 0.95 | 0.95 | 0.9498 | 0.9498 | 0.9498 | 0.9498 | 5000 | 4 | 0.1 | (0.98,0.9) | 0.25 |

**WEB TABLE 34-** Coverage of 95% CIs for log-relative hazard parameter $\beta_{2}$ from unstratified sampling using different methods of analysis and variance estimation, for various probabilities of missing covariate data, in 5,000 simulated cohorts. * indicates coverage outside the expected interval [0.9440; 0.9560]

| Cohort | SCC.True | | SCC.Est | | SCC.Naive | | $n$ | $K$ | $p_{Y}$ | $\boldsymbol{\pi}^{(3)}$ | $\beta_{3}$ |
| --- | --- | --- | --- | --- | --- | --- | --- | --- | --- | --- | --- |
|  | $\hat{V}_{\mathrm{Robust}}$ | $\hat{V}$ | $\hat{V}_{\mathrm{Robust}}$ | $\hat{V}$ | $\hat{V}_{\mathrm{Robust}}$ | $\hat{V}$ |  |  |  |  |  |
| 0.9456 | 0.9524 | 0.9526 | 0.9524 | 0.9526 | 0.9524 | 0.9526 | 5000 | 2 | 0.02 | (0.9,0.8) | -0.3 |
| 0.9456 | 0.9538 | 0.9548 | 0.9534 | 0.9546 | 0.9534 | 0.9546 | 5000 | 2 | 0.02 | (0.98,0.9) | -0.3 |
| 0.9412* | 0.9492 | 0.9494 | 0.9492 | 0.9496 | 0.9492 | 0.9496 | 5000 | 4 | 0.02 | (0.9,0.8) | -0.3 |
| 0.9412* | 0.9462 | 0.9462 | 0.9462 | 0.9462 | 0.9462 | 0.9462 | 5000 | 4 | 0.02 | (0.98,0.9) | -0.3 |
| 0.9478 | 0.9484 | 0.949 | 0.9486 | 0.9492 | 0.9486 | 0.9492 | 10000 | 2 | 0.02 | (0.9,0.8) | -0.3 |
| 0.9478 | 0.9522 | 0.9524 | 0.9522 | 0.9522 | 0.9522 | 0.9522 | 10000 | 2 | 0.02 | (0.98,0.9) | -0.3 |
| 0.9448 | 0.9514 | 0.9514 | 0.951 | 0.951 | 0.951 | 0.951 | 10000 | 4 | 0.02 | (0.9,0.8) | -0.3 |
| 0.9448 | 0.949 | 0.949 | 0.949 | 0.949 | 0.949 | 0.949 | 10000 | 4 | 0.02 | (0.98,0.9) | -0.3 |
| 0.945 | 0.949 | 0.949 | 0.9488 | 0.949 | 0.9488 | 0.949 | 5000 | 2 | 0.05 | (0.9,0.8) | -0.3 |
| 0.945 | 0.944 | 0.9442 | 0.944 | 0.9442 | 0.944 | 0.9442 | 5000 | 2 | 0.05 | (0.98,0.9) | -0.3 |
| 0.952 | 0.9556 | 0.9556 | 0.956 | 0.956 | 0.956 | 0.956 | 5000 | 4 | 0.05 | (0.9,0.8) | -0.3 |
| 0.952 | 0.9558 | 0.9558 | 0.9558 | 0.9558 | 0.9558 | 0.9558 | 5000 | 4 | 0.05 | (0.98,0.9) | -0.3 |
| 0.9462 | 0.952 | 0.9518 | 0.9524 | 0.9524 | 0.9524 | 0.9524 | 10000 | 2 | 0.05 | (0.9,0.8) | -0.3 |
| 0.9462 | 0.9488 | 0.949 | 0.9488 | 0.9488 | 0.9488 | 0.9488 | 10000 | 2 | 0.05 | (0.98,0.9) | -0.3 |
| 0.9532 | 0.9514 | 0.9514 | 0.9514 | 0.9514 | 0.9514 | 0.9514 | 10000 | 4 | 0.05 | (0.9,0.8) | -0.3 |
| 0.9532 | 0.949 | 0.949 | 0.9486 | 0.9486 | 0.9486 | 0.9486 | 10000 | 4 | 0.05 | (0.98,0.9) | -0.3 |
| 0.9512 | 0.9494 | 0.9496 | 0.9494 | 0.9496 | 0.9496 | 0.9496 | 5000 | 2 | 0.1 | (0.9,0.8) | -0.3 |
| 0.9512 | 0.9476 | 0.9476 | 0.9476 | 0.9476 | 0.9476 | 0.9476 | 5000 | 2 | 0.1 | (0.98,0.9) | -0.3 |
| 0.9518 | 0.9506 | 0.9506 | 0.9502 | 0.9502 | 0.9502 | 0.9502 | 5000 | 4 | 0.1 | (0.9,0.8) | -0.3 |
| 0.9518 | 0.9512 | 0.9512 | 0.9514 | 0.9514 | 0.9514 | 0.9514 | 5000 | 4 | 0.1 | (0.98,0.9) | -0.3 |

**WEB TABLE 35-** Coverage of 95% CIs for log-relative hazard parameter $\beta_{3}$ from stratified sampling using different methods of analysis and variance estimation, for various probabilities of missing covariate data, in 5,000 simulated cohorts. * indicates coverage outside the expected interval [0.9440; 0.9560]

| Cohort | USCC.True | | USCC.Est | | USCC.Naive | | $n$ | $K$ | $p_{Y}$ | $\boldsymbol{\pi}^{(3)}$ | $\beta_{3}$ |
| --- | --- | --- | --- | --- | --- | --- | --- | --- | --- | --- | --- |
|  | $\hat{V}_{\mathrm{Robust}}$ | $\hat{V}$ | $\hat{V}_{\mathrm{Robust}}$ | $\hat{V}$ | $\hat{V}_{\mathrm{Robust}}$ | $\hat{V}$ |  |  |  |  |  |
| 0.9456 | 0.951 | 0.9512 | 0.951 | 0.9512 | 0.951 | 0.9512 | 5000 | 2 | 0.02 | (0.9,0.8) | -0.3 |
| 0.9456 | 0.9522 | 0.9522 | 0.9522 | 0.9522 | 0.9522 | 0.9522 | 5000 | 2 | 0.02 | (0.98,0.9) | -0.3 |
| 0.9412 | 0.9456 | 0.9458 | 0.9456 | 0.9456 | 0.9456 | 0.9456 | 5000 | 4 | 0.02 | (0.9,0.8) | -0.3 |
| 0.9412 | 0.9468 | 0.947 | 0.9468 | 0.947 | 0.9468 | 0.947 | 5000 | 4 | 0.02 | (0.98,0.9) | -0.3 |
| 0.9478 | 0.9516 | 0.9516 | 0.9514 | 0.9518 | 0.9514 | 0.9518 | 10000 | 2 | 0.02 | (0.9,0.8) | -0.3 |
| 0.9478 | 0.9496 | 0.9496 | 0.9496 | 0.9496 | 0.9496 | 0.9496 | 10000 | 2 | 0.02 | (0.98,0.9) | -0.3 |
| 0.9448 | 0.9488 | 0.9488 | 0.9488 | 0.9488 | 0.9488 | 0.9488 | 10000 | 4 | 0.02 | (0.9,0.8) | -0.3 |
| 0.9448 | 0.947 | 0.947 | 0.947 | 0.947 | 0.947 | 0.947 | 10000 | 4 | 0.02 | (0.98,0.9) | -0.3 |
| 0.945 | 0.947 | 0.947 | 0.9468 | 0.9468 | 0.9468 | 0.9468 | 5000 | 2 | 0.05 | (0.9,0.8) | -0.3 |
| 0.945 | 0.9508 | 0.9508 | 0.9508 | 0.9508 | 0.9508 | 0.9508 | 5000 | 2 | 0.05 | (0.98,0.9) | -0.3 |
| 0.952 | 0.949 | 0.949 | 0.9488 | 0.9488 | 0.9488 | 0.9488 | 5000 | 4 | 0.05 | (0.9,0.8) | -0.3 |
| 0.952 | 0.954 | 0.954 | 0.954 | 0.954 | 0.954 | 0.954 | 5000 | 4 | 0.05 | (0.98,0.9) | -0.3 |
| 0.9462 | 0.9482 | 0.9482 | 0.9482 | 0.9482 | 0.9482 | 0.9482 | 10000 | 2 | 0.05 | (0.9,0.8) | -0.3 |
| 0.9462 | 0.9474 | 0.9476 | 0.9472 | 0.9472 | 0.9472 | 0.9472 | 10000 | 2 | 0.05 | (0.98,0.9) | -0.3 |
| 0.9532 | 0.9546 | 0.9546 | 0.9544 | 0.9544 | 0.9544 | 0.9544 | 10000 | 4 | 0.05 | (0.9,0.8) | -0.3 |
| 0.9532 | 0.9574* | 0.9574* | 0.9574* | 0.9574* | 0.9574* | 0.9574* | 10000 | 4 | 0.05 | (0.98,0.9) | -0.3 |
| 0.9512 | 0.9558 | 0.9558 | 0.956 | 0.956 | 0.956 | 0.956 | 5000 | 2 | 0.1 | (0.9,0.8) | -0.3 |
| 0.9512 | 0.9554 | 0.9554 | 0.9556 | 0.9556 | 0.9556 | 0.9556 | 5000 | 2 | 0.1 | (0.98,0.9) | -0.3 |
| 0.9518 | 0.95 | 0.95 | 0.9496 | 0.9496 | 0.9496 | 0.9496 | 5000 | 4 | 0.1 | (0.9,0.8) | -0.3 |
| 0.9518 | 0.9498 | 0.9498 | 0.9496 | 0.9496 | 0.9496 | 0.9496 | 5000 | 4 | 0.1 | (0.98,0.9) | -0.3 |

**WEB TABLE 36-** Coverage of 95% CIs for log-relative hazard parameter $\beta_{3}$ from unstratified sampling using different methods of analysis and variance estimation, for various probabilities of missing covariate data, in 5,000 simulated cohorts. * indicates coverage outside the expected interval [0.9440; 0.9560]

| Cohort | SCC.True | | SCC.Est | | SCC.Naive | | $n$ | $K$ | $p_{Y}$ | $\boldsymbol{\pi}^{(3)}$ | $\log\left\{ \pi\left( \tau_{1},\tau_{2};\boldsymbol{x} \right) \right\}$ |
| --- | --- | --- | --- | --- | --- | --- | --- | --- | --- | --- | --- |
|  | $\hat{V}_{\mathrm{Robust}}$ | $\hat{V}$ | $\hat{V}_{\mathrm{Robust}}$ | $\hat{V}$ | $\hat{V}_{\mathrm{Robust}}$ | $\hat{V}$ |  |  |  |  |  |
| 0.949 | 0.9724* | 0.9548 | 0.9716* | 0.9554 | 0.973* | 0.9566 | 5000 | 2 | 0.02 | (0.9,0.8) | -3.948 |
| 0.949 | 0.9714* | 0.9524 | 0.9706* | 0.9522 | 0.9708* | 0.9528 | 5000 | 2 | 0.02 | (0.98,0.9) | -3.948 |
| 0.9494 | 0.9594* | 0.9486 | 0.96* | 0.9498 | 0.9608* | 0.9512 | 5000 | 4 | 0.02 | (0.9,0.8) | -3.948 |
| 0.9494 | 0.9598* | 0.9492 | 0.9596* | 0.949 | 0.9596* | 0.949 | 5000 | 4 | 0.02 | (0.98,0.9) | -3.948 |
| 0.949 | 0.9688* | 0.9508 | 0.9692* | 0.9508 | 0.9698* | 0.953 | 10000 | 2 | 0.02 | (0.9,0.8) | -3.948 |
| 0.949 | 0.969* | 0.9498 | 0.9684* | 0.9498 | 0.9688* | 0.9504 | 10000 | 2 | 0.02 | (0.98,0.9) | -3.948 |
| 0.9476 | 0.9572* | 0.9446 | 0.9564* | 0.9444 | 0.9582* | 0.946 | 10000 | 4 | 0.02 | (0.9,0.8) | -3.948 |
| 0.9476 | 0.9606* | 0.9474 | 0.962* | 0.9476 | 0.9622* | 0.9482 | 10000 | 4 | 0.02 | (0.98,0.9) | -3.948 |
| 0.9532 | 0.9688* | 0.9528 | 0.9696* | 0.9552 | 0.9716* | 0.9564* | 5000 | 2 | 0.05 | (0.9,0.8) | -3.046 |
| 0.9532 | 0.9712* | 0.955 | 0.9708* | 0.9556 | 0.9712* | 0.9558 | 5000 | 2 | 0.05 | (0.98,0.9) | -3.046 |
| 0.9438* | 0.9586* | 0.95 | 0.9578* | 0.9498 | 0.959* | 0.9508 | 5000 | 4 | 0.05 | (0.9,0.8) | -3.046 |
| 0.9438* | 0.9566* | 0.9472 | 0.9554 | 0.947 | 0.9558 | 0.947 | 5000 | 4 | 0.05 | (0.98,0.9) | -3.046 |
| 0.9496 | 0.9702* | 0.9544 | 0.9712* | 0.954 | 0.9722* | 0.955 | 10000 | 2 | 0.05 | (0.9,0.8) | -3.046 |
| 0.9496 | 0.9708* | 0.954 | 0.9704* | 0.955 | 0.9706* | 0.955 | 10000 | 2 | 0.05 | (0.98,0.9) | -3.046 |
| 0.9474 | 0.9578* | 0.9486 | 0.958* | 0.9488 | 0.9592* | 0.95 | 10000 | 4 | 0.05 | (0.9,0.8) | -3.046 |
| 0.9474 | 0.9562* | 0.9464 | 0.9566* | 0.947 | 0.957* | 0.948 | 10000 | 4 | 0.05 | (0.98,0.9) | -3.046 |
| 0.9482 | 0.9622* | 0.9484 | 0.9634* | 0.949 | 0.9642* | 0.9524 | 5000 | 2 | 0.1 | (0.9,0.8) | -2.377 |
| 0.9482 | 0.963* | 0.9494 | 0.964* | 0.9484 | 0.9642* | 0.9492 | 5000 | 2 | 0.1 | (0.98,0.9) | -2.377 |
| 0.9488 | 0.9522 | 0.9478 | 0.9522 | 0.9472 | 0.9536 | 0.9484 | 5000 | 4 | 0.1 | (0.9,0.8) | -2.377 |
| 0.9488 | 0.9526 | 0.9492 | 0.9542 | 0.9512 | 0.9542 | 0.9514 | 5000 | 4 | 0.1 | (0.98,0.9) | -2.377 |

**WEB TABLE 37-** Coverage of 95% CIs for pure risk parameter $\log\left\{ \pi\left( \tau_{1},\tau_{2};\boldsymbol{x} \right) \right\}$ with $\boldsymbol{x}=\left( -1, 1, -0.6 \right)'$, from stratified sampling using different methods of analysis and variance estimation, for various probabilities of missing covariate data, in 5,000 simulated cohorts. * indicates coverage outside the expected interval [0.9440; 0.9560]

| Cohort | USCC.True | | USCC.Est | | USCC.Naive | | $n$ | $K$ | $p_{Y}$ | $\boldsymbol{\pi}^{(3)}$ | $\log\left\{ \pi\left( \tau_{1},\tau_{2};\boldsymbol{x} \right) \right\}$ |
| --- | --- | --- | --- | --- | --- | --- | --- | --- | --- | --- | --- |
|  | $\hat{V}_{\mathrm{Robust}}$ | $\hat{V}$ | $\hat{V}_{\mathrm{Robust}}$ | $\hat{V}$ | $\hat{V}_{\mathrm{Robust}}$ | $\hat{V}$ |  |  |  |  |  |
| 0.949 | 0.9638* | 0.952 | 0.966* | 0.9532 | 0.9668* | 0.9546 | 5000 | 2 | 0.02 | (0.9,0.8) | -3.948 |
| 0.949 | 0.9658* | 0.951 | 0.9652* | 0.953 | 0.9656* | 0.9536 | 5000 | 2 | 0.02 | (0.98,0.9) | -3.948 |
| 0.9494 | 0.959* | 0.951 | 0.9584* | 0.9522 | 0.9596* | 0.9534 | 5000 | 4 | 0.02 | (0.9,0.8) | -3.948 |
| 0.9494 | 0.959* | 0.9516 | 0.9592* | 0.952 | 0.9592* | 0.9522 | 5000 | 4 | 0.02 | (0.98,0.9) | -3.948 |
| 0.949 | 0.9624* | 0.9518 | 0.962* | 0.951 | 0.9634* | 0.9532 | 10000 | 2 | 0.02 | (0.9,0.8) | -3.948 |
| 0.949 | 0.9628* | 0.9506 | 0.9628* | 0.9524 | 0.9632* | 0.9528 | 10000 | 2 | 0.02 | (0.98,0.9) | -3.948 |
| 0.9476 | 0.9556 | 0.9486 | 0.9548 | 0.9492 | 0.9556 | 0.9504 | 10000 | 4 | 0.02 | (0.9,0.8) | -3.948 |
| 0.9476 | 0.956 | 0.9474 | 0.9554 | 0.9476 | 0.9556 | 0.9478 | 10000 | 4 | 0.02 | (0.98,0.9) | -3.948 |
| 0.9532 | 0.9668* | 0.9576* | 0.9672* | 0.9568* | 0.9688* | 0.9586* | 5000 | 2 | 0.05 | (0.9,0.8) | -3.046 |
| 0.9532 | 0.9688* | 0.9566* | 0.9682* | 0.957* | 0.9682* | 0.9574* | 5000 | 2 | 0.05 | (0.98,0.9) | -3.046 |
| 0.9438* | 0.947 | 0.9384* | 0.946 | 0.9394* | 0.9466 | 0.9404* | 5000 | 4 | 0.05 | (0.9,0.8) | -3.046 |
| 0.9438* | 0.947 | 0.9402* | 0.9462 | 0.9392* | 0.9462 | 0.9394* | 5000 | 4 | 0.05 | (0.98,0.9) | -3.046 |
| 0.9496 | 0.9634* | 0.9526 | 0.9628* | 0.9506 | 0.9638* | 0.9522 | 10000 | 2 | 0.05 | (0.9,0.8) | -3.046 |
| 0.9496 | 0.9636* | 0.9522 | 0.9644* | 0.9524 | 0.9644* | 0.9526 | 10000 | 2 | 0.05 | (0.98,0.9) | -3.046 |
| 0.9474 | 0.9568* | 0.9508 | 0.958* | 0.9504 | 0.9584* | 0.9514 | 10000 | 4 | 0.05 | (0.9,0.8) | -3.046 |
| 0.9474 | 0.956 | 0.952 | 0.9558 | 0.9516 | 0.9564* | 0.952 | 10000 | 4 | 0.05 | (0.98,0.9) | -3.046 |
| 0.9482 | 0.9572* | 0.9454 | 0.9584* | 0.9494 | 0.9598* | 0.9506 | 5000 | 2 | 0.1 | (0.9,0.8) | -2.377 |
| 0.9482 | 0.9582* | 0.9446 | 0.9588* | 0.9448 | 0.9588* | 0.9452 | 5000 | 2 | 0.1 | (0.98,0.9) | -2.377 |
| 0.9488 | 0.9484 | 0.9426 | 0.9498 | 0.9446 | 0.9498 | 0.9452 | 5000 | 4 | 0.1 | (0.9,0.8) | -2.377 |
| 0.9488 | 0.9528 | 0.9492 | 0.9532 | 0.9492 | 0.9532 | 0.9494 | 5000 | 4 | 0.1 | (0.98,0.9) | -2.377 |

**WEB TABLE 38-** Coverage of 95% CIs for pure risk parameter $\log\left\{ \pi\left( \tau_{1},\tau_{2};\boldsymbol{x} \right) \right\}$ with $\boldsymbol{x}=\left( -1, 1, -0.6 \right)'$, from unstratified sampling using different methods of analysis and variance estimation, for various probabilities of missing covariate data, in 5,000 simulated cohorts. * indicates coverage outside the expected interval [0.9440; 0.9560]

| Cohort | SCC.True | | SCC.Est | | SCC.Naive | | $n$ | $K$ | $p_{Y}$ | $\boldsymbol{\pi}^{(3)}$ | $\log\left\{ \pi\left( \tau_{1},\tau_{2};\boldsymbol{x} \right) \right\}$ |
| --- | --- | --- | --- | --- | --- | --- | --- | --- | --- | --- | --- |
|  | $\hat{V}_{\mathrm{Robust}}$ | $\hat{V}$ | $\hat{V}_{\mathrm{Robust}}$ | $\hat{V}$ | $\hat{V}_{\mathrm{Robust}}$ | $\hat{V}$ |  |  |  |  |  |
| 0.9484 | 0.967* | 0.9488 | 0.9664* | 0.9488 | 0.9664* | 0.949 | 5000 | 2 | 0.02 | (0.9,0.8) | -5.201 |
| 0.9484 | 0.971* | 0.9498 | 0.9716* | 0.9514 | 0.9716* | 0.9514 | 5000 | 2 | 0.02 | (0.98,0.9) | -5.201 |
| 0.9448 | 0.9606* | 0.9522 | 0.96* | 0.9518 | 0.96* | 0.9522 | 5000 | 4 | 0.02 | (0.9,0.8) | -5.201 |
| 0.9448 | 0.9608* | 0.95 | 0.9606* | 0.9506 | 0.9606* | 0.9506 | 5000 | 4 | 0.02 | (0.98,0.9) | -5.201 |
| 0.95 | 0.9662* | 0.9472 | 0.9664* | 0.9474 | 0.9668* | 0.9482 | 10000 | 2 | 0.02 | (0.9,0.8) | -5.201 |
| 0.95 | 0.9698* | 0.9492 | 0.9694* | 0.9492 | 0.9694* | 0.9494 | 10000 | 2 | 0.02 | (0.98,0.9) | -5.201 |
| 0.9494 | 0.9608* | 0.9502 | 0.9618* | 0.9508 | 0.9618* | 0.951 | 10000 | 4 | 0.02 | (0.9,0.8) | -5.201 |
| 0.9494 | 0.965* | 0.9538 | 0.965* | 0.9542 | 0.965* | 0.9542 | 10000 | 4 | 0.02 | (0.98,0.9) | -5.201 |
| 0.949 | 0.9694* | 0.9498 | 0.969* | 0.9496 | 0.969* | 0.9502 | 5000 | 2 | 0.05 | (0.9,0.8) | -4.289 |
| 0.949 | 0.9692* | 0.9502 | 0.969* | 0.9502 | 0.969* | 0.9504 | 5000 | 2 | 0.05 | (0.98,0.9) | -4.289 |
| 0.9432* | 0.9614* | 0.9516 | 0.9626* | 0.9514 | 0.9626* | 0.9514 | 5000 | 4 | 0.05 | (0.9,0.8) | -4.289 |
| 0.9432* | 0.957* | 0.9478 | 0.957* | 0.9476 | 0.957* | 0.9476 | 5000 | 4 | 0.05 | (0.98,0.9) | -4.289 |
| 0.944 | 0.9656* | 0.949 | 0.9662* | 0.9474 | 0.9662* | 0.9478 | 10000 | 2 | 0.05 | (0.9,0.8) | -4.289 |
| 0.944 | 0.97* | 0.9492 | 0.9708* | 0.9508 | 0.971* | 0.9508 | 10000 | 2 | 0.05 | (0.98,0.9) | -4.289 |
| 0.9486 | 0.9556 | 0.9498 | 0.9564* | 0.95 | 0.9568* | 0.9502 | 10000 | 4 | 0.05 | (0.9,0.8) | -4.289 |
| 0.9486 | 0.9588* | 0.9486 | 0.9596* | 0.949 | 0.9596* | 0.949 | 10000 | 4 | 0.05 | (0.98,0.9) | -4.289 |
| 0.9526 | 0.9652* | 0.9474 | 0.9646* | 0.9494 | 0.9652* | 0.9498 | 5000 | 2 | 0.1 | (0.9,0.8) | -3.602 |
| 0.9526 | 0.9664* | 0.9494 | 0.9666* | 0.95 | 0.9668* | 0.95 | 5000 | 2 | 0.1 | (0.98,0.9) | -3.602 |
| 0.9462 | 0.9562* | 0.9498 | 0.9572* | 0.9484 | 0.9574* | 0.9486 | 5000 | 4 | 0.1 | (0.9,0.8) | -3.602 |
| 0.9462 | 0.9538 | 0.9474 | 0.9538 | 0.948 | 0.9538 | 0.948 | 5000 | 4 | 0.1 | (0.98,0.9) | -3.602 |

**WEB TABLE 39-** Coverage of 95% CIs for pure risk parameter $\log\left\{ \pi\left( \tau_{1},\tau_{2};\boldsymbol{x} \right) \right\}$ with $\boldsymbol{x}=\left( 1, -1, 0.6 \right)'$, from stratified sampling using different methods of analysis and variance estimation, for various probabilities of missing covariate data, in 5,000 simulated cohorts. * indicates coverage outside the expected interval [0.9440; 0.9560]

| Cohort | USCC.True | | USCC.Est | | USCC.Naive | | $n$ | $K$ | $p_{Y}$ | $\boldsymbol{\pi}^{(3)}$ | $\log\left\{ \pi\left( \tau_{1},\tau_{2};\boldsymbol{x} \right) \right\}$ |
| --- | --- | --- | --- | --- | --- | --- | --- | --- | --- | --- | --- |
|  | $\hat{V}_{\mathrm{Robust}}$ | $\hat{V}$ | $\hat{V}_{\mathrm{Robust}}$ | $\hat{V}$ | $\hat{V}_{\mathrm{Robust}}$ | $\hat{V}$ |  |  |  |  |  |
| 0.9484 | 0.9578* | 0.9552 | 0.9584* | 0.9552 | 0.9584* | 0.9556 | 5000 | 2 | 0.02 | (0.9,0.8) | -5.201 |
| 0.9484 | 0.9554 | 0.9528 | 0.9556 | 0.9536 | 0.9556 | 0.9536 | 5000 | 2 | 0.02 | (0.98,0.9) | -5.201 |
| 0.9448 | 0.9478 | 0.9462 | 0.946 | 0.9452 | 0.9466 | 0.9454 | 5000 | 4 | 0.02 | (0.9,0.8) | -5.201 |
| 0.9448 | 0.948 | 0.9466 | 0.9482 | 0.9466 | 0.9482 | 0.9466 | 5000 | 4 | 0.02 | (0.98,0.9) | -5.201 |
| 0.95 | 0.956 | 0.951 | 0.956 | 0.952 | 0.9562* | 0.9522 | 10000 | 2 | 0.02 | (0.9,0.8) | -5.201 |
| 0.95 | 0.9564* | 0.953 | 0.9552 | 0.953 | 0.9554 | 0.953 | 10000 | 2 | 0.02 | (0.98,0.9) | -5.201 |
| 0.9494 | 0.9534 | 0.9518 | 0.9526 | 0.951 | 0.9532 | 0.9512 | 10000 | 4 | 0.02 | (0.9,0.8) | -5.201 |
| 0.9494 | 0.953 | 0.9516 | 0.953 | 0.9516 | 0.953 | 0.9516 | 10000 | 4 | 0.02 | (0.98,0.9) | -5.201 |
| 0.949 | 0.9524 | 0.9508 | 0.9518 | 0.9508 | 0.9518 | 0.951 | 5000 | 2 | 0.05 | (0.9,0.8) | -4.289 |
| 0.949 | 0.9544 | 0.952 | 0.9546 | 0.9518 | 0.9546 | 0.9518 | 5000 | 2 | 0.05 | (0.98,0.9) | -4.289 |
| 0.9432* | 0.9476 | 0.9462 | 0.9486 | 0.9474 | 0.9488 | 0.9476 | 5000 | 4 | 0.05 | (0.9,0.8) | -4.289 |
| 0.9432* | 0.9512 | 0.9498 | 0.9502 | 0.9488 | 0.9502 | 0.9488 | 5000 | 4 | 0.05 | (0.98,0.9) | -4.289 |
| 0.944 | 0.952 | 0.9504 | 0.9526 | 0.9504 | 0.9528 | 0.9508 | 10000 | 2 | 0.05 | (0.9,0.8) | -4.289 |
| 0.944 | 0.9534 | 0.95 | 0.954 | 0.951 | 0.954 | 0.951 | 10000 | 2 | 0.05 | (0.98,0.9) | -4.289 |
| 0.9486 | 0.9508 | 0.9496 | 0.9522 | 0.951 | 0.9524 | 0.9514 | 10000 | 4 | 0.05 | (0.9,0.8) | -4.289 |
| 0.9486 | 0.952 | 0.9504 | 0.9522 | 0.9504 | 0.9522 | 0.9504 | 10000 | 4 | 0.05 | (0.98,0.9) | -4.289 |
| 0.9526 | 0.9546 | 0.952 | 0.9546 | 0.9522 | 0.9548 | 0.9526 | 5000 | 2 | 0.1 | (0.9,0.8) | -3.602 |
| 0.9526 | 0.9586* | 0.9566* | 0.9596* | 0.9562* | 0.9596* | 0.9564* | 5000 | 2 | 0.1 | (0.98,0.9) | -3.602 |
| 0.9462 | 0.9504 | 0.9492 | 0.9514 | 0.9508 | 0.9518 | 0.9512 | 5000 | 4 | 0.1 | (0.9,0.8) | -3.602 |
| 0.9462 | 0.9512 | 0.9504 | 0.9514 | 0.95 | 0.9514 | 0.95 | 5000 | 4 | 0.1 | (0.98,0.9) | -3.602 |

**WEB TABLE 40-** Coverage of 95% CIs for pure risk parameter $\log\left\{ \pi\left( \tau_{1},\tau_{2};\boldsymbol{x} \right) \right\}$ with $\boldsymbol{x}=\left( 1, -1, 0.6 \right)'$, from unstratified sampling using different methods of analysis and variance estimation, for various probabilities of missing covariate data, in 5,000 simulated cohorts. * indicates coverage outside the expected interval [0.9440; 0.9560]

| Cohort | SCC.True | | SCC.Est | | SCC.Naive | | $n$ | $K$ | $p_{Y}$ | $\boldsymbol{\pi}^{(3)}$ | $\log\left\{ \pi\left( \tau_{1},\tau_{2};\boldsymbol{x} \right) \right\}$ |
| --- | --- | --- | --- | --- | --- | --- | --- | --- | --- | --- | --- |
|  | $\hat{V}_{\mathrm{Robust}}$ | $\hat{V}$ | $\hat{V}_{\mathrm{Robust}}$ | $\hat{V}$ | $\hat{V}_{\mathrm{Robust}}$ | $\hat{V}$ |  |  |  |  |  |
| 0.944 | 0.962 | 0.952 | 0.964 | 0.952 | 0.9644 | 0.9522 | 5000 | 2 | 0.02 | (0.9,0.8) | -4.702 |
| 0.944 | 0.9652 | 0.949 | 0.9652 | 0.9484 | 0.9658 | 0.949 | 5000 | 2 | 0.02 | (0.98,0.9) | -4.702 |
| 0.9426 | 0.955 | 0.9498 | 0.9546 | 0.9496 | 0.9548 | 0.9504 | 5000 | 4 | 0.02 | (0.9,0.8) | -4.702 |
| 0.9426 | 0.9542 | 0.9476 | 0.954 | 0.9468 | 0.954 | 0.947 | 5000 | 4 | 0.02 | (0.98,0.9) | -4.702 |
| 0.9458 | 0.9598 | 0.9498 | 0.9614 | 0.9486 | 0.9618 | 0.949 | 10000 | 2 | 0.02 | (0.9,0.8) | -4.702 |
| 0.9458 | 0.9678 | 0.9524 | 0.9666 | 0.951 | 0.9666 | 0.9514 | 10000 | 2 | 0.02 | (0.98,0.9) | -4.702 |
| 0.9514 | 0.9602 | 0.9528 | 0.96 | 0.953 | 0.9604 | 0.9534 | 10000 | 4 | 0.02 | (0.9,0.8) | -4.702 |
| 0.9514 | 0.9594 | 0.952 | 0.9602 | 0.9522 | 0.9602 | 0.9526 | 10000 | 4 | 0.02 | (0.98,0.9) | -4.702 |
| 0.9468 | 0.96 | 0.9456 | 0.9598 | 0.9462 | 0.9604 | 0.9476 | 5000 | 2 | 0.05 | (0.9,0.8) | -3.793 |
| 0.9468 | 0.9558 | 0.9452 | 0.9564 | 0.944 | 0.9564 | 0.944 | 5000 | 2 | 0.05 | (0.98,0.9) | -3.793 |
| 0.9526 | 0.9554 | 0.9512 | 0.9572 | 0.9522 | 0.9576 | 0.9528 | 5000 | 4 | 0.05 | (0.9,0.8) | -3.793 |
| 0.9526 | 0.9594 | 0.9536 | 0.9602 | 0.9534 | 0.9602 | 0.9534 | 5000 | 4 | 0.05 | (0.98,0.9) | -3.793 |
| 0.9464 | 0.9658 | 0.9546 | 0.9654 | 0.9538 | 0.9662 | 0.9546 | 10000 | 2 | 0.05 | (0.9,0.8) | -3.793 |
| 0.9464 | 0.9654 | 0.952 | 0.9648 | 0.9522 | 0.9648 | 0.9526 | 10000 | 2 | 0.05 | (0.98,0.9) | -3.793 |
| 0.9532 | 0.9602 | 0.9562 | 0.9592 | 0.9548 | 0.9594 | 0.955 | 10000 | 4 | 0.05 | (0.9,0.8) | -3.793 |
| 0.9532 | 0.9616 | 0.956 | 0.9616 | 0.954 | 0.9618 | 0.9542 | 10000 | 4 | 0.05 | (0.98,0.9) | -3.793 |
| 0.9532 | 0.9616 | 0.9542 | 0.9614 | 0.9532 | 0.9632 | 0.9542 | 5000 | 2 | 0.1 | (0.9,0.8) | -3.111 |
| 0.9532 | 0.9638 | 0.951 | 0.9638 | 0.9518 | 0.964 | 0.9524 | 5000 | 2 | 0.1 | (0.98,0.9) | -3.111 |
| 0.9532 | 0.9556 | 0.951 | 0.956 | 0.952 | 0.9564 | 0.9522 | 5000 | 4 | 0.1 | (0.9,0.8) | -3.111 |
| 0.9532 | 0.9556 | 0.9512 | 0.9546 | 0.9518 | 0.9546 | 0.9518 | 5000 | 4 | 0.1 | (0.98,0.9) | -3.111 |

**WEB TABLE 41-** Coverage of 95% CIs for pure risk parameter $\log\left\{ \pi\left( \tau_{1},\tau_{2};\boldsymbol{x} \right) \right\}$ with $\boldsymbol{x}=\left( 1, 1, 0.6 \right)'$, from stratified sampling using different methods of analysis and variance estimation, for various probabilities of missing covariate data, in 5,000 simulated cohorts. * indicates coverage outside the expected interval [0.9440; 0.9560]

| Cohort | USCC.True | | USCC.Est | | USCC.Naive | | $n$ | $K$ | $p_{Y}$ | $\boldsymbol{\pi}^{(3)}$ | $\log\left\{ \pi\left( \tau_{1},\tau_{2};\boldsymbol{x} \right) \right\}$ |
| --- | --- | --- | --- | --- | --- | --- | --- | --- | --- | --- | --- |
|  | $\hat{V}_{\mathrm{Robust}}$ | $\hat{V}$ | $\hat{V}_{\mathrm{Robust}}$ | $\hat{V}$ | $\hat{V}_{\mathrm{Robust}}$ | $\hat{V}$ |  |  |  |  |  |
| 0.944 | 0.9604* | 0.9524 | 0.9578* | 0.9534 | 0.9584* | 0.9538 | 5000 | 2 | 0.02 | (0.9,0.8) | -4.702 |
| 0.944 | 0.959* | 0.954 | 0.9588* | 0.9536 | 0.9588* | 0.9536 | 5000 | 2 | 0.02 | (0.98,0.9) | -4.702 |
| 0.9426* | 0.95 | 0.947 | 0.9488 | 0.9466 | 0.9492 | 0.9468 | 5000 | 4 | 0.02 | (0.9,0.8) | -4.702 |
| 0.9426* | 0.9518 | 0.9474 | 0.9512 | 0.947 | 0.9512 | 0.947 | 5000 | 4 | 0.02 | (0.98,0.9) | -4.702 |
| 0.9458 | 0.9608* | 0.9554 | 0.9608* | 0.957* | 0.9616* | 0.958* | 10000 | 2 | 0.02 | (0.9,0.8) | -4.702 |
| 0.9458 | 0.9608* | 0.9548 | 0.9614* | 0.9544 | 0.9614* | 0.9546 | 10000 | 2 | 0.02 | (0.98,0.9) | -4.702 |
| 0.9514 | 0.956 | 0.9516 | 0.9542 | 0.9504 | 0.9542 | 0.951 | 10000 | 4 | 0.02 | (0.9,0.8) | -4.702 |
| 0.9514 | 0.9572* | 0.9528 | 0.9558 | 0.952 | 0.956 | 0.952 | 10000 | 4 | 0.02 | (0.98,0.9) | -4.702 |
| 0.9468 | 0.9526 | 0.948 | 0.9556 | 0.9494 | 0.9562* | 0.95 | 5000 | 2 | 0.05 | (0.9,0.8) | -3.793 |
| 0.9468 | 0.9536 | 0.9478 | 0.953 | 0.948 | 0.953 | 0.948 | 5000 | 2 | 0.05 | (0.98,0.9) | -3.793 |
| 0.9526 | 0.9538 | 0.9512 | 0.9554 | 0.9532 | 0.9554 | 0.954 | 5000 | 4 | 0.05 | (0.9,0.8) | -3.793 |
| 0.9526 | 0.9604* | 0.9586* | 0.9604* | 0.958 | 0.9604* | 0.958 | 5000 | 4 | 0.05 | (0.98,0.9) | -3.793 |
| 0.9464 | 0.9586* | 0.9522 | 0.9584* | 0.9532 | 0.9594* | 0.9536 | 10000 | 2 | 0.05 | (0.9,0.8) | -3.793 |
| 0.9464 | 0.9596* | 0.9538 | 0.9602* | 0.9542 | 0.9602* | 0.9542 | 10000 | 2 | 0.05 | (0.98,0.9) | -3.793 |
| 0.9532 | 0.9564* | 0.9546 | 0.958* | 0.9566* | 0.9586* | 0.9566* | 10000 | 4 | 0.05 | (0.9,0.8) | -3.793 |
| 0.9532 | 0.9564* | 0.9536 | 0.9572* | 0.953 | 0.9574* | 0.953 | 10000 | 4 | 0.05 | (0.98,0.9) | -3.793 |
| 0.9532 | 0.9566* | 0.9522 | 0.9578* | 0.9536 | 0.9586* | 0.9544 | 5000 | 2 | 0.1 | (0.9,0.8) | -3.111 |
| 0.9532 | 0.9562* | 0.9518 | 0.9558 | 0.9518 | 0.956 | 0.9518 | 5000 | 2 | 0.1 | (0.98,0.9) | -3.111 |
| 0.9532 | 0.9474 | 0.9454 | 0.9484 | 0.947 | 0.9486 | 0.9476 | 5000 | 4 | 0.1 | (0.9,0.8) | -3.111 |
| 0.9532 | 0.954 | 0.952 | 0.9532 | 0.9522 | 0.9532 | 0.9522 | 5000 | 4 | 0.1 | (0.98,0.9) | -3.111 |

**WEB TABLE 42-** Coverage of 95% CIs for pure risk parameter $\log\left\{ \pi\left( \tau_{1},\tau_{2};\boldsymbol{x} \right) \right\}$ with $\boldsymbol{x}=\left( 1, 1, 0.6 \right)'$, from unstratified sampling using different methods of analysis and variance estimation, for various probabilities of missing covariate data, in 5,000 simulated cohorts. * indicates coverage outside the expected interval [0.9440; 0.9560]

| Cohort | | SCC.True | | | SCC.Est | | | SCC.Naive | | | $n$ | $K$ | $p_{Y}$ | $\boldsymbol{\pi}^{(3)}$ | $\beta_{1}$ |
| --- | --- | --- | --- | --- | --- | --- | --- | --- | --- | --- | --- | --- | --- | --- | --- |
| Empir var | $\hat{V}_{\mathrm{Robust}}$ | Empir var | $\hat{V}_{\mathrm{Robust}}$ | $\hat{V}$ | Empir var | $\hat{V}_{\mathrm{Robust}}$ | $\hat{V}$ | Empir var | $\hat{V}_{\mathrm{Robust}}$ | $\hat{V}$ |  |  |  |  |  |
| 0.0135 | 0.014 | 0.0224 | 0.0261 | 0.0232 | 0.0224 | 0.0261 | 0.0232 | 0.0224 | 0.0261 | 0.0232 | 5000 | 2 | 0.02 | (0.9,0.8) | -0.2 |
| 0.0135 | 0.014 | 0.0195 | 0.0232 | 0.0202 | 0.0195 | 0.0232 | 0.0202 | 0.0195 | 0.0232 | 0.0202 | 5000 | 2 | 0.02 | (0.98,0.9) | -0.2 |
| 0.0141 | 0.0139 | 0.0207 | 0.0214 | 0.02 | 0.0207 | 0.0214 | 0.02 | 0.0207 | 0.0214 | 0.02 | 5000 | 4 | 0.02 | (0.9,0.8) | -0.2 |
| 0.0141 | 0.0139 | 0.0181 | 0.019 | 0.0176 | 0.0181 | 0.019 | 0.0176 | 0.0181 | 0.019 | 0.0176 | 5000 | 4 | 0.02 | (0.98,0.9) | -0.2 |
| 0.007 | 0.007 | 0.0111 | 0.0124 | 0.0109 | 0.0111 | 0.0124 | 0.0109 | 0.0111 | 0.0124 | 0.0109 | 10000 | 2 | 0.02 | (0.9,0.8) | -0.2 |
| 0.007 | 0.007 | 0.0096 | 0.0111 | 0.0096 | 0.0096 | 0.0111 | 0.0096 | 0.0096 | 0.0111 | 0.0096 | 10000 | 2 | 0.02 | (0.98,0.9) | -0.2 |
| 0.0071 | 0.0069 | 0.01 | 0.0104 | 0.0097 | 0.01 | 0.0104 | 0.0097 | 0.01 | 0.0104 | 0.0097 | 10000 | 4 | 0.02 | (0.9,0.8) | -0.2 |
| 0.0071 | 0.0069 | 0.0086 | 0.0093 | 0.0086 | 0.0086 | 0.0093 | 0.0086 | 0.0086 | 0.0093 | 0.0086 | 10000 | 4 | 0.02 | (0.98,0.9) | -0.2 |
| 0.0056 | 0.0056 | 0.0084 | 0.0095 | 0.0085 | 0.0084 | 0.0095 | 0.0085 | 0.0084 | 0.0095 | 0.0085 | 5000 | 2 | 0.05 | (0.9,0.8) | -0.2 |
| 0.0056 | 0.0056 | 0.0075 | 0.0085 | 0.0075 | 0.0075 | 0.0085 | 0.0075 | 0.0075 | 0.0085 | 0.0075 | 5000 | 2 | 0.05 | (0.98,0.9) | -0.2 |
| 0.0058 | 0.0056 | 0.0078 | 0.0081 | 0.0077 | 0.0078 | 0.0081 | 0.0077 | 0.0078 | 0.0081 | 0.0077 | 5000 | 4 | 0.05 | (0.9,0.8) | -0.2 |
| 0.0058 | 0.0056 | 0.0069 | 0.0072 | 0.0068 | 0.0069 | 0.0072 | 0.0068 | 0.0069 | 0.0072 | 0.0068 | 5000 | 4 | 0.05 | (0.98,0.9) | -0.2 |
| 0.0028 | 0.0028 | 0.0041 | 0.0047 | 0.0042 | 0.0041 | 0.0047 | 0.0042 | 0.0041 | 0.0047 | 0.0042 | 10000 | 2 | 0.05 | (0.9,0.8) | -0.2 |
| 0.0028 | 0.0028 | 0.0036 | 0.0042 | 0.0037 | 0.0036 | 0.0042 | 0.0037 | 0.0036 | 0.0042 | 0.0037 | 10000 | 2 | 0.05 | (0.98,0.9) | -0.2 |
| 0.0028 | 0.0028 | 0.0038 | 0.004 | 0.0038 | 0.0038 | 0.004 | 0.0038 | 0.0038 | 0.004 | 0.0038 | 10000 | 4 | 0.05 | (0.9,0.8) | -0.2 |
| 0.0028 | 0.0028 | 0.0034 | 0.0036 | 0.0034 | 0.0034 | 0.0036 | 0.0034 | 0.0034 | 0.0036 | 0.0034 | 10000 | 4 | 0.05 | (0.98,0.9) | -0.2 |
| 0.0029 | 0.0029 | 0.0042 | 0.0045 | 0.0041 | 0.0042 | 0.0045 | 0.0041 | 0.0042 | 0.0045 | 0.0041 | 5000 | 2 | 0.1 | (0.9,0.8) | -0.2 |
| 0.0029 | 0.0029 | 0.0037 | 0.0041 | 0.0037 | 0.0037 | 0.0041 | 0.0037 | 0.0037 | 0.0041 | 0.0037 | 5000 | 2 | 0.1 | (0.98,0.9) | -0.2 |
| 0.0029 | 0.0029 | 0.0038 | 0.0039 | 0.0038 | 0.0038 | 0.0039 | 0.0038 | 0.0038 | 0.0039 | 0.0038 | 5000 | 4 | 0.1 | (0.9,0.8) | -0.2 |
| 0.0029 | 0.0029 | 0.0033 | 0.0035 | 0.0033 | 0.0033 | 0.0035 | 0.0033 | 0.0033 | 0.0035 | 0.0033 | 5000 | 4 | 0.1 | (0.98,0.9) | -0.2 |

**WEB TABLE 43-** Empirical variance and mean of estimated variances of log-relative hazard parameter $\beta_{1}$ from stratified sampling using different methods of analysis and variance estimation, for various probabilities of missing covariate data, in 5,000 simulated cohorts

| Cohort | | USCC.True | | | USCC.Est | | | USCC.Naive | | | $n$ | $K$ | $p_{Y}$ | $\boldsymbol{\pi}^{(3)}$ | $\beta_{1}$ |
| --- | --- | --- | --- | --- | --- | --- | --- | --- | --- | --- | --- | --- | --- | --- | --- |
| Empir var | $\hat{V}_{\mathrm{Robust}}$ | Empir var | $\hat{V}_{\mathrm{Robust}}$ | $\hat{V}$ | Empir var | $\hat{V}_{\mathrm{Robust}}$ | $\hat{V}$ | Empir var | $\hat{V}_{\mathrm{Robust}}$ | $\hat{V}$ |  |  |  |  |  |
| 0.0135 | 0.014 | 0.0264 | 0.0273 | 0.0273 | 0.0264 | 0.0273 | 0.0273 | 0.0264 | 0.0273 | 0.0273 | 5000 | 2 | 0.02 | (0.9,0.8) | -0.2 |
| 0.0135 | 0.014 | 0.0235 | 0.0242 | 0.0243 | 0.0235 | 0.0242 | 0.0243 | 0.0235 | 0.0242 | 0.0243 | 5000 | 2 | 0.02 | (0.98,0.9) | -0.2 |
| 0.0141 | 0.0139 | 0.0223 | 0.0221 | 0.0221 | 0.0223 | 0.0221 | 0.0221 | 0.0223 | 0.0221 | 0.0221 | 5000 | 4 | 0.02 | (0.9,0.8) | -0.2 |
| 0.0141 | 0.0139 | 0.0197 | 0.0196 | 0.0196 | 0.0197 | 0.0196 | 0.0196 | 0.0197 | 0.0196 | 0.0196 | 5000 | 4 | 0.02 | (0.98,0.9) | -0.2 |
| 0.007 | 0.007 | 0.0129 | 0.0128 | 0.0128 | 0.0129 | 0.0128 | 0.0128 | 0.0129 | 0.0128 | 0.0128 | 10000 | 2 | 0.02 | (0.9,0.8) | -0.2 |
| 0.007 | 0.007 | 0.0115 | 0.0115 | 0.0115 | 0.0115 | 0.0115 | 0.0115 | 0.0115 | 0.0115 | 0.0115 | 10000 | 2 | 0.02 | (0.98,0.9) | -0.2 |
| 0.0071 | 0.0069 | 0.0111 | 0.0106 | 0.0106 | 0.0111 | 0.0106 | 0.0106 | 0.0111 | 0.0106 | 0.0106 | 10000 | 4 | 0.02 | (0.9,0.8) | -0.2 |
| 0.0071 | 0.0069 | 0.0099 | 0.0095 | 0.0095 | 0.0099 | 0.0095 | 0.0095 | 0.0099 | 0.0095 | 0.0095 | 10000 | 4 | 0.02 | (0.98,0.9) | -0.2 |
| 0.0056 | 0.0056 | 0.0097 | 0.0098 | 0.0098 | 0.0097 | 0.0098 | 0.0098 | 0.0097 | 0.0098 | 0.0098 | 5000 | 2 | 0.05 | (0.9,0.8) | -0.2 |
| 0.0056 | 0.0056 | 0.0087 | 0.0088 | 0.0088 | 0.0087 | 0.0088 | 0.0088 | 0.0087 | 0.0088 | 0.0088 | 5000 | 2 | 0.05 | (0.98,0.9) | -0.2 |
| 0.0058 | 0.0056 | 0.0084 | 0.0083 | 0.0083 | 0.0084 | 0.0083 | 0.0083 | 0.0084 | 0.0083 | 0.0083 | 5000 | 4 | 0.05 | (0.9,0.8) | -0.2 |
| 0.0058 | 0.0056 | 0.0075 | 0.0074 | 0.0074 | 0.0075 | 0.0074 | 0.0074 | 0.0075 | 0.0074 | 0.0074 | 5000 | 4 | 0.05 | (0.98,0.9) | -0.2 |
| 0.0028 | 0.0028 | 0.0049 | 0.0049 | 0.0049 | 0.0049 | 0.0049 | 0.0049 | 0.0049 | 0.0049 | 0.0049 | 10000 | 2 | 0.05 | (0.9,0.8) | -0.2 |
| 0.0028 | 0.0028 | 0.0044 | 0.0044 | 0.0044 | 0.0044 | 0.0044 | 0.0044 | 0.0044 | 0.0044 | 0.0044 | 10000 | 2 | 0.05 | (0.98,0.9) | -0.2 |
| 0.0028 | 0.0028 | 0.004 | 0.0041 | 0.0041 | 0.004 | 0.0041 | 0.0041 | 0.004 | 0.0041 | 0.0041 | 10000 | 4 | 0.05 | (0.9,0.8) | -0.2 |
| 0.0028 | 0.0028 | 0.0036 | 0.0037 | 0.0037 | 0.0036 | 0.0037 | 0.0037 | 0.0036 | 0.0037 | 0.0037 | 10000 | 4 | 0.05 | (0.98,0.9) | -0.2 |
| 0.0029 | 0.0029 | 0.0047 | 0.0047 | 0.0047 | 0.0047 | 0.0047 | 0.0047 | 0.0047 | 0.0047 | 0.0047 | 5000 | 2 | 0.1 | (0.9,0.8) | -0.2 |
| 0.0029 | 0.0029 | 0.0041 | 0.0042 | 0.0042 | 0.0041 | 0.0042 | 0.0042 | 0.0041 | 0.0042 | 0.0042 | 5000 | 2 | 0.1 | (0.98,0.9) | -0.2 |
| 0.0029 | 0.0029 | 0.004 | 0.004 | 0.004 | 0.004 | 0.004 | 0.004 | 0.004 | 0.004 | 0.004 | 5000 | 4 | 0.1 | (0.9,0.8) | -0.2 |
| 0.0029 | 0.0029 | 0.0036 | 0.0035 | 0.0035 | 0.0036 | 0.0035 | 0.0035 | 0.0036 | 0.0035 | 0.0035 | 5000 | 4 | 0.1 | (0.98,0.9) | -0.2 |

**WEB TABLE 44-** Empirical variance and mean of estimated variances of log-relative hazard parameter $\beta_{1}$ from unstratified sampling using different methods of analysis and variance estimation, for various probabilities of missing covariate data, in 5,000 simulated cohorts

| Cohort | | SCC.True | | | SCC.Est | | | SCC.Naive | | | $n$ | $K$ | $p_{Y}$ | $\boldsymbol{\pi}^{(3)}$ | $\beta_{2}$ |
| --- | --- | --- | --- | --- | --- | --- | --- | --- | --- | --- | --- | --- | --- | --- | --- |
| Empir var | $\hat{V}_{\mathrm{Robust}}$ | Empir var | $\hat{V}_{\mathrm{Robust}}$ | $\hat{V}$ | Empir var | $\hat{V}_{\mathrm{Robust}}$ | $\hat{V}$ | Empir var | $\hat{V}_{\mathrm{Robust}}$ | $\hat{V}$ |  |  |  |  |  |
| 0.021 | 0.0197 | 0.0321 | 0.0353 | 0.0303 | 0.0321 | 0.0353 | 0.0303 | 0.0321 | 0.0353 | 0.0303 | 5000 | 2 | 0.02 | (0.9,0.8) | 0.25 |
| 0.021 | 0.0197 | 0.0281 | 0.0314 | 0.0264 | 0.0281 | 0.0314 | 0.0264 | 0.0281 | 0.0314 | 0.0264 | 5000 | 2 | 0.02 | (0.98,0.9) | 0.25 |
| 0.02 | 0.0198 | 0.0274 | 0.0298 | 0.0275 | 0.0274 | 0.0298 | 0.0275 | 0.0274 | 0.0298 | 0.0275 | 5000 | 4 | 0.02 | (0.9,0.8) | 0.25 |
| 0.02 | 0.0198 | 0.0243 | 0.0265 | 0.0241 | 0.0243 | 0.0265 | 0.0241 | 0.0243 | 0.0265 | 0.0241 | 5000 | 4 | 0.02 | (0.98,0.9) | 0.25 |
| 0.0097 | 0.0098 | 0.0145 | 0.017 | 0.0145 | 0.0145 | 0.017 | 0.0145 | 0.0145 | 0.017 | 0.0145 | 10000 | 2 | 0.02 | (0.9,0.8) | 0.25 |
| 0.0097 | 0.0098 | 0.0126 | 0.0151 | 0.0127 | 0.0126 | 0.0151 | 0.0127 | 0.0126 | 0.0151 | 0.0127 | 10000 | 2 | 0.02 | (0.98,0.9) | 0.25 |
| 0.0099 | 0.0097 | 0.0135 | 0.0144 | 0.0133 | 0.0135 | 0.0144 | 0.0133 | 0.0135 | 0.0144 | 0.0133 | 10000 | 4 | 0.02 | (0.9,0.8) | 0.25 |
| 0.0099 | 0.0097 | 0.0119 | 0.0129 | 0.0117 | 0.0119 | 0.0129 | 0.0117 | 0.0119 | 0.0129 | 0.0117 | 10000 | 4 | 0.02 | (0.98,0.9) | 0.25 |
| 0.0079 | 0.0079 | 0.0116 | 0.0132 | 0.0114 | 0.0116 | 0.0132 | 0.0114 | 0.0116 | 0.0132 | 0.0114 | 5000 | 2 | 0.05 | (0.9,0.8) | 0.25 |
| 0.0079 | 0.0079 | 0.01 | 0.0118 | 0.01 | 0.01 | 0.0118 | 0.01 | 0.01 | 0.0118 | 0.01 | 5000 | 2 | 0.05 | (0.98,0.9) | 0.25 |
| 0.0083 | 0.0079 | 0.0109 | 0.0113 | 0.0106 | 0.0109 | 0.0113 | 0.0106 | 0.0109 | 0.0113 | 0.0106 | 5000 | 4 | 0.05 | (0.9,0.8) | 0.25 |
| 0.0083 | 0.0079 | 0.0096 | 0.0101 | 0.0093 | 0.0096 | 0.0101 | 0.0093 | 0.0096 | 0.0101 | 0.0093 | 5000 | 4 | 0.05 | (0.98,0.9) | 0.25 |
| 0.004 | 0.0039 | 0.0058 | 0.0065 | 0.0056 | 0.0058 | 0.0065 | 0.0056 | 0.0058 | 0.0065 | 0.0056 | 10000 | 2 | 0.05 | (0.9,0.8) | 0.25 |
| 0.004 | 0.0039 | 0.0051 | 0.0058 | 0.005 | 0.0051 | 0.0058 | 0.005 | 0.0051 | 0.0058 | 0.005 | 10000 | 2 | 0.05 | (0.98,0.9) | 0.25 |
| 0.004 | 0.0039 | 0.0053 | 0.0056 | 0.0052 | 0.0053 | 0.0056 | 0.0052 | 0.0053 | 0.0056 | 0.0052 | 10000 | 4 | 0.05 | (0.9,0.8) | 0.25 |
| 0.004 | 0.0039 | 0.0046 | 0.005 | 0.0046 | 0.0046 | 0.005 | 0.0046 | 0.0046 | 0.005 | 0.0046 | 10000 | 4 | 0.05 | (0.98,0.9) | 0.25 |
| 0.004 | 0.004 | 0.0056 | 0.0063 | 0.0056 | 0.0056 | 0.0063 | 0.0056 | 0.0056 | 0.0063 | 0.0056 | 5000 | 2 | 0.1 | (0.9,0.8) | 0.25 |
| 0.004 | 0.004 | 0.0049 | 0.0056 | 0.005 | 0.0049 | 0.0056 | 0.005 | 0.0049 | 0.0056 | 0.005 | 5000 | 2 | 0.1 | (0.98,0.9) | 0.25 |
| 0.004 | 0.004 | 0.0052 | 0.0054 | 0.0052 | 0.0052 | 0.0054 | 0.0052 | 0.0052 | 0.0054 | 0.0052 | 5000 | 4 | 0.1 | (0.9,0.8) | 0.25 |
| 0.004 | 0.004 | 0.0046 | 0.0049 | 0.0046 | 0.0046 | 0.0049 | 0.0046 | 0.0046 | 0.0049 | 0.0046 | 5000 | 4 | 0.1 | (0.98,0.9) | 0.25 |

**WEB TABLE 45-** Empirical variance and mean of estimated variances of log-relative hazard parameter $\beta_{2}$ from stratified sampling using different methods of analysis and variance estimation, for various probabilities of missing covariate data, in 5,000 simulated cohorts

| Cohort | | USCC.True | | | USCC.Est | | | USCC.Naive | | | $n$ | $K$ | $p_{Y}$ | $\boldsymbol{\pi}^{(3)}$ | $\beta_{2}$ |
| --- | --- | --- | --- | --- | --- | --- | --- | --- | --- | --- | --- | --- | --- | --- | --- |
| Empir var | $\hat{V}_{\mathrm{Robust}}$ | Empir var | $\hat{V}_{\mathrm{Robust}}$ | $\hat{V}$ | Empir var | $\hat{V}_{\mathrm{Robust}}$ | $\hat{V}$ | Empir var | $\hat{V}_{\mathrm{Robust}}$ | $\hat{V}$ |  |  |  |  |  |
| 0.021 | 0.0197 | 0.0368 | 0.0357 | 0.0357 | 0.0368 | 0.0357 | 0.0357 | 0.0368 | 0.0357 | 0.0357 | 5000 | 2 | 0.02 | (0.9,0.8) | 0.25 |
| 0.021 | 0.0197 | 0.0332 | 0.0318 | 0.0318 | 0.0332 | 0.0318 | 0.0318 | 0.0332 | 0.0318 | 0.0318 | 5000 | 2 | 0.02 | (0.98,0.9) | 0.25 |
| 0.02 | 0.0198 | 0.0301 | 0.03 | 0.03 | 0.0301 | 0.03 | 0.03 | 0.0301 | 0.03 | 0.03 | 5000 | 4 | 0.02 | (0.9,0.8) | 0.25 |
| 0.02 | 0.0198 | 0.0268 | 0.0267 | 0.0267 | 0.0268 | 0.0267 | 0.0267 | 0.0268 | 0.0267 | 0.0267 | 5000 | 4 | 0.02 | (0.98,0.9) | 0.25 |
| 0.0097 | 0.0098 | 0.017 | 0.0171 | 0.0171 | 0.017 | 0.0171 | 0.0171 | 0.017 | 0.0171 | 0.0171 | 10000 | 2 | 0.02 | (0.9,0.8) | 0.25 |
| 0.0097 | 0.0098 | 0.0152 | 0.0153 | 0.0153 | 0.0152 | 0.0153 | 0.0153 | 0.0152 | 0.0153 | 0.0153 | 10000 | 2 | 0.02 | (0.98,0.9) | 0.25 |
| 0.0099 | 0.0097 | 0.0144 | 0.0145 | 0.0145 | 0.0144 | 0.0145 | 0.0145 | 0.0144 | 0.0145 | 0.0145 | 10000 | 4 | 0.02 | (0.9,0.8) | 0.25 |
| 0.0099 | 0.0097 | 0.0129 | 0.0129 | 0.0129 | 0.0129 | 0.0129 | 0.0129 | 0.0129 | 0.0129 | 0.0129 | 10000 | 4 | 0.02 | (0.98,0.9) | 0.25 |
| 0.0079 | 0.0079 | 0.0131 | 0.0132 | 0.0133 | 0.0131 | 0.0132 | 0.0133 | 0.0131 | 0.0132 | 0.0133 | 5000 | 2 | 0.05 | (0.9,0.8) | 0.25 |
| 0.0079 | 0.0079 | 0.0119 | 0.0118 | 0.0119 | 0.0119 | 0.0118 | 0.0119 | 0.0119 | 0.0118 | 0.0119 | 5000 | 2 | 0.05 | (0.98,0.9) | 0.25 |
| 0.0083 | 0.0079 | 0.0117 | 0.0113 | 0.0113 | 0.0117 | 0.0113 | 0.0113 | 0.0117 | 0.0113 | 0.0113 | 5000 | 4 | 0.05 | (0.9,0.8) | 0.25 |
| 0.0083 | 0.0079 | 0.0104 | 0.0101 | 0.0101 | 0.0104 | 0.0101 | 0.0101 | 0.0104 | 0.0101 | 0.0101 | 5000 | 4 | 0.05 | (0.98,0.9) | 0.25 |
| 0.004 | 0.0039 | 0.0066 | 0.0065 | 0.0065 | 0.0066 | 0.0065 | 0.0065 | 0.0066 | 0.0065 | 0.0065 | 10000 | 2 | 0.05 | (0.9,0.8) | 0.25 |
| 0.004 | 0.0039 | 0.0059 | 0.0058 | 0.0058 | 0.0059 | 0.0058 | 0.0058 | 0.0059 | 0.0058 | 0.0058 | 10000 | 2 | 0.05 | (0.98,0.9) | 0.25 |
| 0.004 | 0.0039 | 0.0055 | 0.0056 | 0.0056 | 0.0055 | 0.0056 | 0.0056 | 0.0055 | 0.0056 | 0.0056 | 10000 | 4 | 0.05 | (0.9,0.8) | 0.25 |
| 0.004 | 0.0039 | 0.005 | 0.005 | 0.005 | 0.005 | 0.005 | 0.005 | 0.005 | 0.005 | 0.005 | 10000 | 4 | 0.05 | (0.98,0.9) | 0.25 |
| 0.004 | 0.004 | 0.0063 | 0.0063 | 0.0063 | 0.0063 | 0.0063 | 0.0063 | 0.0063 | 0.0063 | 0.0063 | 5000 | 2 | 0.1 | (0.9,0.8) | 0.25 |
| 0.004 | 0.004 | 0.0055 | 0.0057 | 0.0057 | 0.0055 | 0.0057 | 0.0057 | 0.0055 | 0.0057 | 0.0057 | 5000 | 2 | 0.1 | (0.98,0.9) | 0.25 |
| 0.004 | 0.004 | 0.0054 | 0.0055 | 0.0055 | 0.0054 | 0.0055 | 0.0055 | 0.0054 | 0.0055 | 0.0055 | 5000 | 4 | 0.1 | (0.9,0.8) | 0.25 |
| 0.004 | 0.004 | 0.0049 | 0.0049 | 0.0049 | 0.0049 | 0.0049 | 0.0049 | 0.0049 | 0.0049 | 0.0049 | 5000 | 4 | 0.1 | (0.98,0.9) | 0.25 |

**WEB TABLE 46-** Empirical variance and mean of estimated variances of log-relative hazard parameter $\beta_{2}$ from unstratified sampling using different methods of analysis and variance estimation, for various probabilities of missing covariate data, in 5,000 simulated cohorts

| Cohort | | SCC.True | | | SCC.Est | | | SCC.Naive | | | $n$ | $K$ | $p_{Y}$ | $\boldsymbol{\pi}^{(3)}$ | $\beta_{3}$ |
| --- | --- | --- | --- | --- | --- | --- | --- | --- | --- | --- | --- | --- | --- | --- | --- |
| Empir var | $\hat{V}_{\mathrm{Robust}}$ | Empir var | $\hat{V}_{\mathrm{Robust}}$ | $\hat{V}$ | Empir var | $\hat{V}_{\mathrm{Robust}}$ | $\hat{V}$ | Empir var | $\hat{V}_{\mathrm{Robust}}$ | $\hat{V}$ |  |  |  |  |  |
| 0.0137 | 0.0136 | 0.027 | 0.0262 | 0.0263 | 0.027 | 0.0262 | 0.0263 | 0.027 | 0.0262 | 0.0263 | 5000 | 2 | 0.02 | (0.9,0.8) | -0.3 |
| 0.0137 | 0.0136 | 0.0236 | 0.0233 | 0.0234 | 0.0236 | 0.0233 | 0.0234 | 0.0236 | 0.0233 | 0.0234 | 5000 | 2 | 0.02 | (0.98,0.9) | -0.3 |
| 0.0141 | 0.0136 | 0.0218 | 0.0214 | 0.0214 | 0.0218 | 0.0214 | 0.0214 | 0.0218 | 0.0214 | 0.0214 | 5000 | 4 | 0.02 | (0.9,0.8) | -0.3 |
| 0.0141 | 0.0136 | 0.0194 | 0.019 | 0.019 | 0.0194 | 0.019 | 0.019 | 0.0194 | 0.019 | 0.019 | 5000 | 4 | 0.02 | (0.98,0.9) | -0.3 |
| 0.0069 | 0.0068 | 0.0126 | 0.0124 | 0.0125 | 0.0126 | 0.0124 | 0.0125 | 0.0126 | 0.0124 | 0.0125 | 10000 | 2 | 0.02 | (0.9,0.8) | -0.3 |
| 0.0069 | 0.0068 | 0.011 | 0.0111 | 0.0111 | 0.011 | 0.0111 | 0.0111 | 0.011 | 0.0111 | 0.0111 | 10000 | 2 | 0.02 | (0.98,0.9) | -0.3 |
| 0.0068 | 0.0068 | 0.0104 | 0.0103 | 0.0103 | 0.0104 | 0.0103 | 0.0103 | 0.0104 | 0.0103 | 0.0103 | 10000 | 4 | 0.02 | (0.9,0.8) | -0.3 |
| 0.0068 | 0.0068 | 0.0094 | 0.0092 | 0.0092 | 0.0094 | 0.0092 | 0.0092 | 0.0094 | 0.0092 | 0.0092 | 10000 | 4 | 0.02 | (0.98,0.9) | -0.3 |
| 0.0057 | 0.0055 | 0.01 | 0.0096 | 0.0096 | 0.01 | 0.0096 | 0.0096 | 0.01 | 0.0096 | 0.0096 | 5000 | 2 | 0.05 | (0.9,0.8) | -0.3 |
| 0.0057 | 0.0055 | 0.009 | 0.0086 | 0.0086 | 0.009 | 0.0086 | 0.0086 | 0.009 | 0.0086 | 0.0086 | 5000 | 2 | 0.05 | (0.98,0.9) | -0.3 |
| 0.0054 | 0.0056 | 0.0078 | 0.0081 | 0.0081 | 0.0078 | 0.0081 | 0.0081 | 0.0078 | 0.0081 | 0.0081 | 5000 | 4 | 0.05 | (0.9,0.8) | -0.3 |
| 0.0054 | 0.0056 | 0.007 | 0.0072 | 0.0072 | 0.007 | 0.0072 | 0.0072 | 0.007 | 0.0072 | 0.0072 | 5000 | 4 | 0.05 | (0.98,0.9) | -0.3 |
| 0.0028 | 0.0028 | 0.0046 | 0.0047 | 0.0047 | 0.0046 | 0.0047 | 0.0047 | 0.0046 | 0.0047 | 0.0047 | 10000 | 2 | 0.05 | (0.9,0.8) | -0.3 |
| 0.0028 | 0.0042 | 0.0042 | 0.0042 | 0.0042 | 0.0042 | 0.0042 | 0.0042 | 0.0042 | 0.0042 | 0.0042 | 10000 | 2 | 0.05 | (0.98,0.9) | -0.3 |
| 0.0028 | 0.0039 | 0.0039 | 0.004 | 0.004 | 0.0039 | 0.004 | 0.004 | 0.0039 | 0.004 | 0.004 | 10000 | 4 | 0.05 | (0.9,0.8) | -0.3 |
| 0.0028 | 0.0035 | 0.0035 | 0.0036 | 0.0036 | 0.0035 | 0.0036 | 0.0036 | 0.0035 | 0.0036 | 0.0036 | 10000 | 4 | 0.05 | (0.98,0.9) | -0.3 |
| 0.0028 | 0.0044 | 0.0044 | 0.0045 | 0.0045 | 0.0044 | 0.0045 | 0.0045 | 0.0044 | 0.0045 | 0.0045 | 5000 | 2 | 0.1 | (0.9,0.8) | -0.3 |
| 0.0028 | 0.004 | 0.004 | 0.004 | 0.004 | 0.004 | 0.004 | 0.004 | 0.004 | 0.004 | 0.004 | 5000 | 2 | 0.1 | (0.98,0.9) | -0.3 |
| 0.0028 | 0.0038 | 0.0038 | 0.0038 | 0.0038 | 0.0038 | 0.0038 | 0.0038 | 0.0038 | 0.0038 | 0.0038 | 5000 | 4 | 0.1 | (0.9,0.8) | -0.3 |
| 0.0028 | 0.0034 | 0.0034 | 0.0034 | 0.0034 | 0.0034 | 0.0034 | 0.0034 | 0.0034 | 0.0034 | 0.0034 | 5000 | 4 | 0.1 | (0.98,0.9) | -0.3 |

**WEB TABLE 47-** Empirical variance and mean of estimated variances of log-relative hazard parameter $\beta_{3}$ from stratified sampling using different methods of analysis and variance estimation, for various probabilities of missing covariate data, in 5,000 simulated cohorts

| Cohort | | USCC.True | | | USCC.Est | | | USCC.Naive | | | $n$ | $K$ | $p_{Y}$ | $\boldsymbol{\pi}^{(3)}$ | $\beta_{3}$ |
| --- | --- | --- | --- | --- | --- | --- | --- | --- | --- | --- | --- | --- | --- | --- | --- |
| Empir var | $\hat{V}_{\mathrm{Robust}}$ | Empir var | $\hat{V}_{\mathrm{Robust}}$ | $\hat{V}$ | Empir var | $\hat{V}_{\mathrm{Robust}}$ | $\hat{V}$ | Empir var | $\hat{V}_{\mathrm{Robust}}$ | $\hat{V}$ |  |  |  |  |  |
| 0.0137 | 0.0136 | 0.028 | 0.0274 | 0.0274 | 0.028 | 0.0274 | 0.0274 | 0.028 | 0.0274 | 0.0274 | 5000 | 2 | 0.02 | (0.9,0.8) | -0.3 |
| 0.0137 | 0.0136 | 0.0249 | 0.0244 | 0.0244 | 0.0249 | 0.0244 | 0.0244 | 0.0249 | 0.0244 | 0.0244 | 5000 | 2 | 0.02 | (0.98,0.9) | -0.3 |
| 0.0141 | 0.0136 | 0.0232 | 0.0219 | 0.0219 | 0.0232 | 0.0219 | 0.0219 | 0.0232 | 0.0219 | 0.0219 | 5000 | 4 | 0.02 | (0.9,0.8) | -0.3 |
| 0.0141 | 0.0136 | 0.0205 | 0.0195 | 0.0195 | 0.0205 | 0.0195 | 0.0195 | 0.0205 | 0.0195 | 0.0195 | 5000 | 4 | 0.02 | (0.98,0.9) | -0.3 |
| 0.0069 | 0.0068 | 0.0131 | 0.0129 | 0.0129 | 0.0131 | 0.0129 | 0.0129 | 0.0131 | 0.0129 | 0.0129 | 10000 | 2 | 0.02 | (0.9,0.8) | -0.3 |
| 0.0069 | 0.0068 | 0.0119 | 0.0115 | 0.0115 | 0.0119 | 0.0115 | 0.0115 | 0.0119 | 0.0115 | 0.0115 | 10000 | 2 | 0.02 | (0.98,0.9) | -0.3 |
| 0.0068 | 0.0068 | 0.0108 | 0.0105 | 0.0105 | 0.0108 | 0.0105 | 0.0105 | 0.0108 | 0.0105 | 0.0105 | 10000 | 4 | 0.02 | (0.9,0.8) | -0.3 |
| 0.0068 | 0.0068 | 0.0096 | 0.0094 | 0.0094 | 0.0096 | 0.0094 | 0.0094 | 0.0096 | 0.0094 | 0.0094 | 10000 | 4 | 0.02 | (0.98,0.9) | -0.3 |
| 0.0057 | 0.0055 | 0.0101 | 0.0099 | 0.0099 | 0.0101 | 0.0099 | 0.0099 | 0.0101 | 0.0099 | 0.0099 | 5000 | 2 | 0.05 | (0.9,0.8) | -0.3 |
| 0.0057 | 0.0055 | 0.0089 | 0.0088 | 0.0089 | 0.0089 | 0.0088 | 0.0089 | 0.0089 | 0.0088 | 0.0089 | 5000 | 2 | 0.05 | (0.98,0.9) | -0.3 |
| 0.0054 | 0.0056 | 0.008 | 0.0082 | 0.0082 | 0.008 | 0.0082 | 0.0082 | 0.008 | 0.0082 | 0.0082 | 5000 | 4 | 0.05 | (0.9,0.8) | -0.3 |
| 0.0054 | 0.0056 | 0.0071 | 0.0073 | 0.0074 | 0.0071 | 0.0073 | 0.0074 | 0.0071 | 0.0073 | 0.0074 | 5000 | 4 | 0.05 | (0.98,0.9) | -0.3 |
| 0.0028 | 0.0028 | 0.0049 | 0.0049 | 0.0049 | 0.0049 | 0.0049 | 0.0049 | 0.0049 | 0.0049 | 0.0049 | 10000 | 2 | 0.05 | (0.9,0.8) | -0.3 |
| 0.0028 | 0.0042 | 0.0045 | 0.0044 | 0.0044 | 0.0045 | 0.0044 | 0.0044 | 0.0045 | 0.0044 | 0.0044 | 10000 | 2 | 0.05 | (0.98,0.9) | -0.3 |
| 0.0028 | 0.0039 | 0.004 | 0.0041 | 0.0041 | 0.004 | 0.0041 | 0.0041 | 0.004 | 0.0041 | 0.0041 | 10000 | 4 | 0.05 | (0.9,0.8) | -0.3 |
| 0.0028 | 0.0035 | 0.0035 | 0.0036 | 0.0036 | 0.0035 | 0.0036 | 0.0036 | 0.0035 | 0.0036 | 0.0036 | 10000 | 4 | 0.05 | (0.98,0.9) | -0.3 |
| 0.0028 | 0.0044 | 0.0044 | 0.0046 | 0.0046 | 0.0044 | 0.0046 | 0.0046 | 0.0044 | 0.0046 | 0.0046 | 5000 | 2 | 0.1 | (0.9,0.8) | -0.3 |
| 0.0028 | 0.004 | 0.0039 | 0.0042 | 0.0042 | 0.0039 | 0.0042 | 0.0042 | 0.0039 | 0.0042 | 0.0042 | 5000 | 2 | 0.1 | (0.98,0.9) | -0.3 |
| 0.0028 | 0.0038 | 0.0039 | 0.0039 | 0.0039 | 0.0039 | 0.0039 | 0.0039 | 0.0039 | 0.0039 | 0.0039 | 5000 | 4 | 0.1 | (0.9,0.8) | -0.3 |
| 0.0028 | 0.0034 | 0.0034 | 0.0035 | 0.0035 | 0.0034 | 0.0035 | 0.0035 | 0.0034 | 0.0035 | 0.0035 | 5000 | 4 | 0.1 | (0.98,0.9) | -0.3 |

**WEB TABLE 48-** Empirical variance and mean of estimated variances of log-relative hazard parameter $\beta_{3}$ from unstratified sampling using different methods of analysis and variance estimation, for various probabilities of missing covariate data, in 5,000 simulated cohorts

| Cohort | | SCC.True | | | SCC.Est | | | SCC.Naive | | | $n$ | $K$ | $p_{Y}$ | $\boldsymbol{\pi}^{(3)}$ | | $\log\left\{ \pi\left( \tau_{1},\tau_{2};\boldsymbol{x} \right) \right\}$ |
| --- | --- | --- | --- | --- | --- | --- | --- | --- | --- | --- | --- | --- | --- | --- | --- | --- |
| Empir var | $\hat{V}_{\mathrm{Robust}}$ | Empir var | $\hat{V}_{\mathrm{Robust}}$ | $\hat{V}$ | Empir var | $\hat{V}_{\mathrm{Robust}}$ | $\hat{V}$ | Empir var | $\hat{V}_{\mathrm{Robust}}$ | $\hat{V}$ |  |  |  |  |  |  |
| 0.0256 | 0.025 | 0.0337 | 0.0398 | 0.0334 | 0.0332 | 0.0393 | 0.0329 | 0.0332 | 0.0398 | 0.0334 | 5000 | 2 | 0.02 | (0.9,0.8) | -3.948 | |
| 0.0256 | 0.025 | 0.0312 | 0.0371 | 0.0307 | 0.0311 | 0.037 | 0.0306 | 0.0311 | 0.0371 | 0.0307 | 5000 | 2 | 0.02 | (0.98,0.9) | -3.948 | |
| 0.0255 | 0.0248 | 0.031 | 0.0332 | 0.0301 | 0.0307 | 0.0329 | 0.0299 | 0.0307 | 0.0332 | 0.0301 | 5000 | 4 | 0.02 | (0.9,0.8) | -3.948 | |
| 0.0255 | 0.0248 | 0.0289 | 0.0311 | 0.0281 | 0.0288 | 0.0311 | 0.028 | 0.0288 | 0.0311 | 0.0281 | 5000 | 4 | 0.02 | (0.98,0.9) | -3.948 | |
| 0.0123 | 0.0123 | 0.0162 | 0.0191 | 0.0159 | 0.0159 | 0.0188 | 0.0156 | 0.0159 | 0.0191 | 0.0159 | 10000 | 2 | 0.02 | (0.9,0.8) | -3.948 | |
| 0.0123 | 0.0123 | 0.015 | 0.0179 | 0.0147 | 0.0149 | 0.0179 | 0.0147 | 0.0149 | 0.0179 | 0.0147 | 10000 | 2 | 0.02 | (0.98,0.9) | -3.948 | |
| 0.0127 | 0.0122 | 0.0154 | 0.0161 | 0.0146 | 0.0152 | 0.016 | 0.0145 | 0.0152 | 0.0161 | 0.0146 | 10000 | 4 | 0.02 | (0.9,0.8) | -3.948 | |
| 0.0127 | 0.0122 | 0.0142 | 0.0152 | 0.0137 | 0.0141 | 0.0152 | 0.0137 | 0.0141 | 0.0152 | 0.0137 | 10000 | 4 | 0.02 | (0.98,0.9) | -3.948 | |
| 0.0095 | 0.0096 | 0.012 | 0.0144 | 0.0122 | 0.0118 | 0.0142 | 0.012 | 0.0118 | 0.0144 | 0.0122 | 5000 | 2 | 0.05 | (0.9,0.8) | -3.046 | |
| 0.0095 | 0.0096 | 0.0111 | 0.0135 | 0.0114 | 0.0111 | 0.0134 | 0.0113 | 0.0111 | 0.0135 | 0.0114 | 5000 | 2 | 0.05 | (0.98,0.9) | -3.046 | |
| 0.0103 | 0.0097 | 0.0117 | 0.0122 | 0.0113 | 0.0116 | 0.0121 | 0.0112 | 0.0116 | 0.0122 | 0.0113 | 5000 | 4 | 0.05 | (0.9,0.8) | -3.046 | |
| 0.0103 | 0.0097 | 0.0111 | 0.0115 | 0.0106 | 0.0111 | 0.0115 | 0.0106 | 0.0111 | 0.0115 | 0.0106 | 5000 | 4 | 0.05 | (0.98,0.9) | -3.046 | |
| 0.0047 | 0.0048 | 0.0059 | 0.0071 | 0.006 | 0.0058 | 0.007 | 0.0059 | 0.0058 | 0.0071 | 0.006 | 10000 | 2 | 0.05 | (0.9,0.8) | -3.046 | |
| 0.0047 | 0.0048 | 0.0055 | 0.0067 | 0.0056 | 0.0055 | 0.0067 | 0.0056 | 0.0055 | 0.0067 | 0.0056 | 10000 | 2 | 0.05 | (0.98,0.9) | -3.046 | |
| 0.0049 | 0.0048 | 0.0056 | 0.006 | 0.0056 | 0.0056 | 0.006 | 0.0055 | 0.0056 | 0.006 | 0.0056 | 10000 | 4 | 0.05 | (0.9,0.8) | -3.046 | |
| 0.0049 | 0.0048 | 0.0053 | 0.0057 | 0.0053 | 0.0053 | 0.0057 | 0.0052 | 0.0053 | 0.0057 | 0.0053 | 10000 | 4 | 0.05 | (0.98,0.9) | -3.046 | |
| 0.0048 | 0.0047 | 0.0059 | 0.0065 | 0.0058 | 0.0058 | 0.0065 | 0.0057 | 0.0058 | 0.0065 | 0.0058 | 5000 | 2 | 0.1 | (0.9,0.8) | -2.377 | |
| 0.0048 | 0.0047 | 0.0055 | 0.0062 | 0.0054 | 0.0055 | 0.0061 | 0.0054 | 0.0055 | 0.0062 | 0.0054 | 5000 | 2 | 0.1 | (0.98,0.9) | -2.377 | |
| 0.0048 | 0.0047 | 0.0055 | 0.0056 | 0.0054 | 0.0055 | 0.0056 | 0.0053 | 0.0055 | 0.0056 | 0.0054 | 5000 | 4 | 0.1 | (0.9,0.8) | -2.377 | |
| 0.0048 | 0.0047 | 0.0051 | 0.0053 | 0.0051 | 0.0051 | 0.0053 | 0.0051 | 0.0051 | 0.0053 | 0.0051 | 5000 | 4 | 0.1 | (0.98,0.9) | -2.377 | |

**WEB TABLE 49-** Empirical variance and mean of estimated variances of pure risk parameter $\log\left\{ \pi\left( \tau_{1},\tau_{2};\boldsymbol{x} \right) \right\}$ with $\boldsymbol{x}=\left( -1, 1, -0.6 \right)'$, from stratified sampling using different methods of analysis and variance estimation, for various probabilities of missing covariate data, in 5,000 simulated cohorts

| Cohort | | USCC.True | | | USCC.Est | | | USCC.Naive | | | $n$ | $K$ | $p_{Y}$ | $\boldsymbol{\pi}^{(3)}$ | | $\log\left\{ \pi\left( \tau_{1},\tau_{2};\boldsymbol{x} \right) \right\}$ |
| --- | --- | --- | --- | --- | --- | --- | --- | --- | --- | --- | --- | --- | --- | --- | --- | --- |
| Empir var | $\hat{V}_{\mathrm{Robust}}$ | Empir var | $\hat{V}_{\mathrm{Robust}}$ | $\hat{V}$ | Empir var | $\hat{V}_{\mathrm{Robust}}$ | $\hat{V}$ | Empir var | $\hat{V}_{\mathrm{Robust}}$ | $\hat{V}$ |  |  |  |  |  |  |
| 0.0256 | 0.025 | 0.038 | 0.042 | 0.0375 | 0.0374 | 0.0415 | 0.037 | 0.0374 | 0.042 | 0.0375 | 5000 | 2 | 0.02 | (0.9,0.8) | -3.948 | |
| 0.0256 | 0.025 | 0.0352 | 0.0391 | 0.0347 | 0.0351 | 0.039 | 0.0346 | 0.0351 | 0.0391 | 0.0347 | 5000 | 2 | 0.02 | (0.98,0.9) | -3.948 | |
| 0.0255 | 0.0248 | 0.0325 | 0.0343 | 0.0322 | 0.0321 | 0.0341 | 0.0319 | 0.0321 | 0.0343 | 0.0322 | 5000 | 4 | 0.02 | (0.9,0.8) | -3.948 | |
| 0.0255 | 0.0248 | 0.0304 | 0.0322 | 0.03 | 0.0303 | 0.0321 | 0.03 | 0.0303 | 0.0322 | 0.03 | 5000 | 4 | 0.02 | (0.98,0.9) | -3.948 | |
| 0.0123 | 0.0123 | 0.018 | 0.0201 | 0.0179 | 0.0178 | 0.0199 | 0.0176 | 0.0178 | 0.0201 | 0.0179 | 10000 | 2 | 0.02 | (0.9,0.8) | -3.948 | |
| 0.0123 | 0.0123 | 0.0167 | 0.0188 | 0.0166 | 0.0167 | 0.0188 | 0.0165 | 0.0167 | 0.0188 | 0.0166 | 10000 | 2 | 0.02 | (0.98,0.9) | -3.948 | |
| 0.0127 | 0.0122 | 0.0162 | 0.0166 | 0.0155 | 0.0161 | 0.0165 | 0.0154 | 0.0161 | 0.0166 | 0.0155 | 10000 | 4 | 0.02 | (0.9,0.8) | -3.948 | |
| 0.0127 | 0.0122 | 0.0153 | 0.0156 | 0.0146 | 0.0153 | 0.0156 | 0.0145 | 0.0153 | 0.0156 | 0.0146 | 10000 | 4 | 0.02 | (0.98,0.9) | -3.948 | |
| 0.0095 | 0.0096 | 0.0132 | 0.0151 | 0.0136 | 0.0131 | 0.0149 | 0.0134 | 0.0131 | 0.0151 | 0.0136 | 5000 | 2 | 0.05 | (0.9,0.8) | -3.046 | |
| 0.0095 | 0.0096 | 0.0125 | 0.0142 | 0.0126 | 0.0125 | 0.0141 | 0.0126 | 0.0125 | 0.0142 | 0.0126 | 5000 | 2 | 0.05 | (0.98,0.9) | -3.046 | |
| 0.0103 | 0.0097 | 0.0127 | 0.0126 | 0.0119 | 0.0126 | 0.0125 | 0.0118 | 0.0126 | 0.0126 | 0.0119 | 5000 | 4 | 0.05 | (0.9,0.8) | -3.046 | |
| 0.0103 | 0.0097 | 0.0119 | 0.0119 | 0.0112 | 0.0119 | 0.0119 | 0.0112 | 0.0119 | 0.0119 | 0.0112 | 5000 | 4 | 0.05 | (0.98,0.9) | -3.046 | |
| 0.0047 | 0.0048 | 0.0066 | 0.0075 | 0.0067 | 0.0065 | 0.0074 | 0.0066 | 0.0065 | 0.0075 | 0.0067 | 10000 | 2 | 0.05 | (0.9,0.8) | -3.046 | |
| 0.0047 | 0.0048 | 0.0061 | 0.007 | 0.0062 | 0.0061 | 0.007 | 0.0062 | 0.0061 | 0.007 | 0.0062 | 10000 | 2 | 0.05 | (0.98,0.9) | -3.046 | |
| 0.0049 | 0.0048 | 0.0058 | 0.0062 | 0.0059 | 0.0058 | 0.0062 | 0.0058 | 0.0058 | 0.0062 | 0.0059 | 10000 | 4 | 0.05 | (0.9,0.8) | -3.046 | |
| 0.0049 | 0.0048 | 0.0056 | 0.0059 | 0.0055 | 0.0056 | 0.0059 | 0.0055 | 0.0056 | 0.0059 | 0.0055 | 10000 | 4 | 0.05 | (0.98,0.9) | -3.046 | |
| 0.0048 | 0.0047 | 0.0066 | 0.0069 | 0.0063 | 0.0065 | 0.0068 | 0.0062 | 0.0065 | 0.0069 | 0.0063 | 5000 | 2 | 0.1 | (0.9,0.8) | -2.377 | |
| 0.0048 | 0.0047 | 0.0061 | 0.0065 | 0.0059 | 0.0061 | 0.0065 | 0.0059 | 0.0061 | 0.0065 | 0.0059 | 5000 | 2 | 0.1 | (0.98,0.9) | -2.377 | |
| 0.0048 | 0.0047 | 0.0057 | 0.0058 | 0.0056 | 0.0057 | 0.0057 | 0.0055 | 0.0057 | 0.0058 | 0.0056 | 5000 | 4 | 0.1 | (0.9,0.8) | -2.377 | |
| 0.0048 | 0.0047 | 0.0054 | 0.0055 | 0.0053 | 0.0054 | 0.0055 | 0.0052 | 0.0054 | 0.0055 | 0.0053 | 5000 | 4 | 0.1 | (0.98,0.9) | -2.377 | |

**WEB TABLE 50-** Empirical variance and mean of estimated variances of pure risk parameter $\log\left\{ \pi\left( \tau_{1},\tau_{2};\boldsymbol{x} \right) \right\}$ with $\boldsymbol{x}=\left( -1, 1, -0.6 \right)'$, from unstratified sampling using different methods of analysis and variance estimation, for various probabilities of missing covariate data, in 5,000 simulated cohorts

| Cohort | | SCC.True | | | SCC.Est | | | SCC.Naive | | | $n$ | $K$ | $p_{Y}$ | $\boldsymbol{\pi}^{(3)}$ | | $\log\left\{ \pi\left( \tau_{1},\tau_{2};\boldsymbol{x} \right) \right\}$ |
| --- | --- | --- | --- | --- | --- | --- | --- | --- | --- | --- | --- | --- | --- | --- | --- | --- |
| Empir var | $\hat{V}_{\mathrm{Robust}}$ | Empir var | $\hat{V}_{\mathrm{Robust}}$ | $\hat{V}$ | Empir var | $\hat{V}_{\mathrm{Robust}}$ | $\hat{V}$ | Empir var | $\hat{V}_{\mathrm{Robust}}$ | $\hat{V}$ |  |  |  |  |  |  |
| 0.1304 | 0.126 | 0.1913 | 0.216 | 0.1829 | 0.1904 | 0.2154 | 0.1823 | 0.1904 | 0.216 | 0.1829 | 5000 | 2 | 0.02 | (0.9,0.8) | -5.201 | |
| 0.1304 | 0.126 | 0.1644 | 0.1933 | 0.1602 | 0.1642 | 0.1932 | 0.16 | 0.1642 | 0.1933 | 0.1602 | 5000 | 2 | 0.02 | (0.98,0.9) | -5.201 | |
| 0.1311 | 0.126 | 0.1699 | 0.1831 | 0.1672 | 0.1697 | 0.1828 | 0.1669 | 0.1697 | 0.183 | 0.1672 | 5000 | 4 | 0.02 | (0.9,0.8) | -5.201 | |
| 0.1311 | 0.126 | 0.1528 | 0.1642 | 0.1484 | 0.1527 | 0.1642 | 0.1483 | 0.1527 | 0.1642 | 0.1484 | 5000 | 4 | 0.02 | (0.98,0.9) | -5.201 | |
| 0.0623 | 0.062 | 0.0891 | 0.1031 | 0.0868 | 0.0888 | 0.1028 | 0.0865 | 0.0888 | 0.1031 | 0.0868 | 10000 | 2 | 0.02 | (0.9,0.8) | -5.201 | |
| 0.0623 | 0.062 | 0.0779 | 0.0928 | 0.0764 | 0.0778 | 0.0928 | 0.0764 | 0.0778 | 0.0928 | 0.0764 | 10000 | 2 | 0.02 | (0.98,0.9) | -5.201 | |
| 0.0617 | 0.0618 | 0.08 | 0.0886 | 0.0807 | 0.0797 | 0.0884 | 0.0805 | 0.0797 | 0.0886 | 0.0807 | 10000 | 4 | 0.02 | (0.9,0.8) | -5.201 | |
| 0.0617 | 0.0618 | 0.0707 | 0.0797 | 0.0718 | 0.0707 | 0.0797 | 0.0718 | 0.0707 | 0.0797 | 0.0718 | 10000 | 4 | 0.02 | (0.98,0.9) | -5.201 | |
| 0.0504 | 0.0496 | 0.0691 | 0.0796 | 0.0679 | 0.0689 | 0.0794 | 0.0677 | 0.0689 | 0.0796 | 0.0679 | 5000 | 2 | 0.05 | (0.9,0.8) | -4.289 | |
| 0.0504 | 0.0496 | 0.0621 | 0.0719 | 0.0601 | 0.0621 | 0.0718 | 0.0601 | 0.0621 | 0.0719 | 0.0601 | 5000 | 2 | 0.05 | (0.98,0.9) | -4.289 | |
| 0.0519 | 0.0498 | 0.0661 | 0.0691 | 0.0638 | 0.0659 | 0.069 | 0.0637 | 0.0659 | 0.0691 | 0.0638 | 5000 | 4 | 0.05 | (0.9,0.8) | -4.289 | |
| 0.0519 | 0.0498 | 0.0589 | 0.0623 | 0.057 | 0.0588 | 0.0623 | 0.057 | 0.0588 | 0.0623 | 0.057 | 5000 | 4 | 0.05 | (0.98,0.9) | -4.289 | |
| 0.0246 | 0.0247 | 0.0335 | 0.0393 | 0.0335 | 0.0334 | 0.0392 | 0.0333 | 0.0334 | 0.0393 | 0.0335 | 10000 | 2 | 0.05 | (0.9,0.8) | -4.289 | |
| 0.0246 | 0.0247 | 0.0294 | 0.0355 | 0.0297 | 0.0294 | 0.0355 | 0.0297 | 0.0294 | 0.0355 | 0.0297 | 10000 | 2 | 0.05 | (0.98,0.9) | -4.289 | |
| 0.0248 | 0.0247 | 0.0318 | 0.0341 | 0.0315 | 0.0317 | 0.0341 | 0.0315 | 0.0317 | 0.0341 | 0.0315 | 10000 | 4 | 0.05 | (0.9,0.8) | -4.289 | |
| 0.0248 | 0.0247 | 0.0281 | 0.0308 | 0.0282 | 0.028 | 0.0308 | 0.0282 | 0.028 | 0.0308 | 0.0282 | 10000 | 4 | 0.05 | (0.98,0.9) | -4.289 | |
| 0.0246 | 0.0248 | 0.0328 | 0.0376 | 0.0329 | 0.0325 | 0.0375 | 0.0328 | 0.0325 | 0.0376 | 0.0329 | 5000 | 2 | 0.1 | (0.9,0.8) | -3.602 | |
| 0.0246 | 0.0248 | 0.029 | 0.034 | 0.0293 | 0.0289 | 0.034 | 0.0293 | 0.0289 | 0.034 | 0.0293 | 5000 | 2 | 0.1 | (0.98,0.9) | -3.602 | |
| 0.0253 | 0.0248 | 0.0313 | 0.0328 | 0.031 | 0.0312 | 0.0328 | 0.031 | 0.0312 | 0.0328 | 0.031 | 5000 | 4 | 0.1 | (0.9,0.8) | -3.602 | |
| 0.0253 | 0.0248 | 0.0283 | 0.0296 | 0.0278 | 0.0283 | 0.0296 | 0.0278 | 0.0283 | 0.0296 | 0.0278 | 5000 | 4 | 0.1 | (0.98,0.9) | -3.602 | |

**WEB TABLE 51-** Empirical variance and mean of estimated variances of pure risk parameter $\log\left\{ \pi\left( \tau_{1},\tau_{2};\boldsymbol{x} \right) \right\}$ with $\boldsymbol{x}=\left( 1, -1, 0.6 \right)'$, from stratified sampling using different methods of analysis and variance estimation, for various probabilities of missing covariate data, in 5,000 simulated cohorts

| Cohort | | USCC.True | | | USCC.Est | | | USCC.Naive | | | $n$ | $K$ | $p_{Y}$ | $\boldsymbol{\pi}^{(3)}$ | | $\log\left\{ \pi\left( \tau_{1},\tau_{2};\boldsymbol{x} \right) \right\}$ |
| --- | --- | --- | --- | --- | --- | --- | --- | --- | --- | --- | --- | --- | --- | --- | --- | --- |
| Empir var | $\hat{V}_{\mathrm{Robust}}$ | Empir var | $\hat{V}_{\mathrm{Robust}}$ | $\hat{V}$ | Empir var | $\hat{V}_{\mathrm{Robust}}$ | $\hat{V}$ | Empir var | $\hat{V}_{\mathrm{Robust}}$ | $\hat{V}$ |  |  |  |  |  |  |
| 0.1304 | 0.126 | 0.2136 | 0.2179 | 0.2135 | 0.2133 | 0.2174 | 0.2129 | 0.2133 | 0.2179 | 0.2135 | 5000 | 2 | 0.02 | (0.9,0.8) | -5.201 | |
| 0.1304 | 0.126 | 0.1925 | 0.1951 | 0.1906 | 0.1924 | 0.195 | 0.1905 | 0.1924 | 0.1951 | 0.1906 | 5000 | 2 | 0.02 | (0.98,0.9) | -5.201 | |
| 0.1311 | 0.126 | 0.1909 | 0.1855 | 0.1833 | 0.1904 | 0.1852 | 0.183 | 0.1904 | 0.1854 | 0.1833 | 5000 | 4 | 0.02 | (0.9,0.8) | -5.201 | |
| 0.1311 | 0.126 | 0.1718 | 0.166 | 0.1639 | 0.1716 | 0.166 | 0.1638 | 0.1716 | 0.166 | 0.1638 | 5000 | 4 | 0.02 | (0.98,0.9) | -5.201 | |
| 0.0623 | 0.062 | 0.0988 | 0.1027 | 0.1004 | 0.0986 | 0.1025 | 0.1002 | 0.0986 | 0.1027 | 0.1004 | 10000 | 2 | 0.02 | (0.9,0.8) | -5.201 | |
| 0.0623 | 0.062 | 0.0893 | 0.0925 | 0.0902 | 0.0893 | 0.0924 | 0.0902 | 0.0893 | 0.0925 | 0.0902 | 10000 | 2 | 0.02 | (0.98,0.9) | -5.201 | |
| 0.0617 | 0.0618 | 0.0874 | 0.0886 | 0.0875 | 0.0873 | 0.0884 | 0.0873 | 0.0873 | 0.0886 | 0.0875 | 10000 | 4 | 0.02 | (0.9,0.8) | -5.201 | |
| 0.0617 | 0.0618 | 0.0784 | 0.0796 | 0.0784 | 0.0784 | 0.0795 | 0.0784 | 0.0784 | 0.0796 | 0.0784 | 10000 | 4 | 0.02 | (0.98,0.9) | -5.201 | |
| 0.0504 | 0.0496 | 0.0773 | 0.0788 | 0.0772 | 0.0769 | 0.0786 | 0.077 | 0.0769 | 0.0788 | 0.0772 | 5000 | 2 | 0.05 | (0.9,0.8) | -4.289 | |
| 0.0504 | 0.0496 | 0.0698 | 0.0712 | 0.0695 | 0.0697 | 0.0711 | 0.0695 | 0.0697 | 0.0712 | 0.0695 | 5000 | 2 | 0.05 | (0.98,0.9) | -4.289 | |
| 0.0519 | 0.0498 | 0.0703 | 0.0689 | 0.0681 | 0.0702 | 0.0687 | 0.068 | 0.0702 | 0.0688 | 0.0681 | 5000 | 4 | 0.05 | (0.9,0.8) | -4.289 | |
| 0.0519 | 0.0498 | 0.0624 | 0.062 | 0.0613 | 0.0623 | 0.062 | 0.0613 | 0.0623 | 0.062 | 0.0613 | 5000 | 4 | 0.05 | (0.98,0.9) | -4.289 | |
| 0.0246 | 0.0247 | 0.0379 | 0.039 | 0.0381 | 0.0377 | 0.0389 | 0.038 | 0.0377 | 0.039 | 0.0381 | 10000 | 2 | 0.05 | (0.9,0.8) | -4.289 | |
| 0.0246 | 0.0247 | 0.0341 | 0.0352 | 0.0344 | 0.034 | 0.0352 | 0.0344 | 0.034 | 0.0352 | 0.0344 | 10000 | 2 | 0.05 | (0.98,0.9) | -4.289 | |
| 0.0248 | 0.0247 | 0.0333 | 0.034 | 0.0336 | 0.0333 | 0.0339 | 0.0335 | 0.0333 | 0.034 | 0.0336 | 10000 | 4 | 0.05 | (0.9,0.8) | -4.289 | |
| 0.0248 | 0.0247 | 0.0302 | 0.0307 | 0.0303 | 0.0302 | 0.0306 | 0.0303 | 0.0302 | 0.0307 | 0.0303 | 10000 | 4 | 0.05 | (0.98,0.9) | -4.289 | |
| 0.0246 | 0.0248 | 0.0365 | 0.0371 | 0.0365 | 0.0364 | 0.037 | 0.0363 | 0.0364 | 0.0371 | 0.0365 | 5000 | 2 | 0.1 | (0.9,0.8) | -3.602 | |
| 0.0246 | 0.0248 | 0.0324 | 0.0336 | 0.0329 | 0.0324 | 0.0335 | 0.0329 | 0.0324 | 0.0336 | 0.0329 | 5000 | 2 | 0.1 | (0.98,0.9) | -3.602 | |
| 0.0253 | 0.0248 | 0.0326 | 0.0326 | 0.0323 | 0.0325 | 0.0325 | 0.0323 | 0.0325 | 0.0326 | 0.0323 | 5000 | 4 | 0.1 | (0.9,0.8) | -3.602 | |
| 0.0253 | 0.0248 | 0.0296 | 0.0294 | 0.0292 | 0.0296 | 0.0294 | 0.0292 | 0.0296 | 0.0294 | 0.0292 | 5000 | 4 | 0.1 | (0.98,0.9) | -3.602 | |

**WEB TABLE 52-** Empirical variance and mean of estimated variances of pure risk parameter $\log\left\{ \pi\left( \tau_{1},\tau_{2};\boldsymbol{x} \right) \right\}$ with $\boldsymbol{x}=\left( 1, -1, 0.6 \right)'$, from unstratified sampling using different methods of analysis and variance estimation, for various probabilities of missing covariate data, in 5,000 simulated cohorts

| Cohort | | SCC.True | | | SCC.Est | | | SCC.Naive | | | $n$ | $K$ | $p_{Y}$ | $\boldsymbol{\pi}^{(3)}$ | | $\log\left\{ \pi\left( \tau_{1},\tau_{2};\boldsymbol{x} \right) \right\}$ |
| --- | --- | --- | --- | --- | --- | --- | --- | --- | --- | --- | --- | --- | --- | --- | --- | --- |
| Empir var | $\hat{V}_{\mathrm{Robust}}$ | Empir var | $\hat{V}_{\mathrm{Robust}}$ | $\hat{V}$ | Empir var | $\hat{V}_{\mathrm{Robust}}$ | $\hat{V}$ | Empir var | $\hat{V}_{\mathrm{Robust}}$ | $\hat{V}$ |  |  |  |  |  |  |
| 0.0569 | 0.0559 | 0.0876 | 0.0938 | 0.0847 | 0.0867 | 0.0932 | 0.0841 | 0.0867 | 0.0938 | 0.0847 | 5000 | 2 | 0.02 | (0.9,0.8) | -4.702 | |
| 0.0569 | 0.0559 | 0.0771 | 0.0849 | 0.0758 | 0.0768 | 0.0848 | 0.0757 | 0.0768 | 0.0849 | 0.0758 | 5000 | 2 | 0.02 | (0.98,0.9) | -4.702 | |
| 0.0577 | 0.0559 | 0.0762 | 0.0793 | 0.075 | 0.0763 | 0.0791 | 0.0747 | 0.0763 | 0.0793 | 0.075 | 5000 | 4 | 0.02 | (0.9,0.8) | -4.702 | |
| 0.0577 | 0.0559 | 0.07 | 0.0721 | 0.0678 | 0.0699 | 0.0721 | 0.0677 | 0.0699 | 0.0721 | 0.0678 | 5000 | 4 | 0.02 | (0.98,0.9) | -4.702 | |
| 0.0274 | 0.0277 | 0.0401 | 0.0444 | 0.0398 | 0.0399 | 0.0441 | 0.0396 | 0.0399 | 0.0444 | 0.0398 | 10000 | 2 | 0.02 | (0.9,0.8) | -4.702 | |
| 0.0274 | 0.0277 | 0.0357 | 0.0404 | 0.0358 | 0.0355 | 0.0403 | 0.0358 | 0.0355 | 0.0404 | 0.0358 | 10000 | 2 | 0.02 | (0.98,0.9) | -4.702 | |
| 0.0267 | 0.0276 | 0.0354 | 0.0382 | 0.036 | 0.0353 | 0.0381 | 0.0359 | 0.0353 | 0.0382 | 0.036 | 10000 | 4 | 0.02 | (0.9,0.8) | -4.702 | |
| 0.0267 | 0.0276 | 0.0319 | 0.0349 | 0.0327 | 0.0319 | 0.0348 | 0.0326 | 0.0319 | 0.0349 | 0.0327 | 10000 | 4 | 0.02 | (0.98,0.9) | -4.702 | |
| 0.0228 | 0.0221 | 0.0317 | 0.034 | 0.0308 | 0.0314 | 0.0338 | 0.0306 | 0.0314 | 0.034 | 0.0308 | 5000 | 2 | 0.05 | (0.9,0.8) | -3.793 | |
| 0.0228 | 0.0221 | 0.0291 | 0.0311 | 0.0278 | 0.0291 | 0.031 | 0.0278 | 0.0291 | 0.0311 | 0.0278 | 5000 | 2 | 0.05 | (0.98,0.9) | -3.793 | |
| 0.0221 | 0.0222 | 0.0285 | 0.0296 | 0.0282 | 0.0283 | 0.0295 | 0.0281 | 0.0283 | 0.0296 | 0.0282 | 5000 | 4 | 0.05 | (0.9,0.8) | -3.793 | |
| 0.0221 | 0.0222 | 0.0257 | 0.0271 | 0.0256 | 0.0256 | 0.0271 | 0.0256 | 0.0256 | 0.0271 | 0.0256 | 5000 | 4 | 0.05 | (0.98,0.9) | -3.793 | |
| 0.011 | 0.011 | 0.0147 | 0.0167 | 0.0151 | 0.0147 | 0.0166 | 0.015 | 0.0147 | 0.0167 | 0.0151 | 10000 | 2 | 0.05 | (0.9,0.8) | -3.793 | |
| 0.011 | 0.011 | 0.0133 | 0.0153 | 0.0137 | 0.0133 | 0.0153 | 0.0136 | 0.0133 | 0.0153 | 0.0137 | 10000 | 2 | 0.05 | (0.98,0.9) | -3.793 | |
| 0.0108 | 0.011 | 0.0136 | 0.0146 | 0.0139 | 0.0136 | 0.0146 | 0.0138 | 0.0136 | 0.0146 | 0.0139 | 10000 | 4 | 0.05 | (0.9,0.8) | -3.793 | |
| 0.0108 | 0.011 | 0.0123 | 0.0134 | 0.0126 | 0.0123 | 0.0134 | 0.0126 | 0.0123 | 0.0134 | 0.0126 | 10000 | 4 | 0.05 | (0.98,0.9) | -3.793 | |
| 0.0108 | 0.011 | 0.0142 | 0.0158 | 0.0145 | 0.0141 | 0.0157 | 0.0144 | 0.0141 | 0.0158 | 0.0145 | 5000 | 2 | 0.1 | (0.9,0.8) | -3.111 | |
| 0.0108 | 0.011 | 0.013 | 0.0145 | 0.0132 | 0.013 | 0.0145 | 0.0132 | 0.013 | 0.0145 | 0.0132 | 5000 | 2 | 0.1 | (0.98,0.9) | -3.111 | |
| 0.0108 | 0.011 | 0.0133 | 0.0139 | 0.0134 | 0.0133 | 0.0139 | 0.0134 | 0.0133 | 0.0139 | 0.0134 | 5000 | 4 | 0.1 | (0.9,0.8) | -3.111 | |
| 0.0108 | 0.011 | 0.0122 | 0.0127 | 0.0122 | 0.0121 | 0.0127 | 0.0122 | 0.0121 | 0.0127 | 0.0122 | 5000 | 4 | 0.1 | (0.98,0.9) | -3.111 | |

**WEB TABLE 53-** Empirical variance and mean of estimated variances of pure risk parameter $\log\left\{ \pi\left( \tau_{1},\tau_{2};\boldsymbol{x} \right) \right\}$ with $\boldsymbol{x}=\left( 1, 1, 0.6 \right)'$, from stratified sampling using different methods of analysis and variance estimation, for various probabilities of missing covariate data, in 5,000 simulated cohorts

| Cohort | | USCC.True | | | USCC.Est | | | USCC.Naive | | | $n$ | $K$ | $p_{Y}$ | $\boldsymbol{\pi}^{(3)}$ | | $\log\left\{ \pi\left( \tau_{1},\tau_{2};\boldsymbol{x} \right) \right\}$ |
| --- | --- | --- | --- | --- | --- | --- | --- | --- | --- | --- | --- | --- | --- | --- | --- | --- |
| Empir var | $\hat{V}_{\mathrm{Robust}}$ | Empir var | $\hat{V}_{\mathrm{Robust}}$ | $\hat{V}$ | Empir var | $\hat{V}_{\mathrm{Robust}}$ | $\hat{V}$ | Empir var | $\hat{V}_{\mathrm{Robust}}$ | $\hat{V}$ |  |  |  |  |  |  |
| 0.0569 | 0.0559 | 0.0922 | 0.0962 | 0.0917 | 0.0916 | 0.0957 | 0.0911 | 0.0916 | 0.0962 | 0.0917 | 5000 | 2 | 0.02 | (0.9,0.8) | -4.702 | |
| 0.0569 | 0.0559 | 0.083 | 0.087 | 0.0825 | 0.0828 | 0.0869 | 0.0824 | 0.0828 | 0.087 | 0.0825 | 5000 | 2 | 0.02 | (0.98,0.9) | -4.702 | |
| 0.0577 | 0.0559 | 0.0822 | 0.0808 | 0.0786 | 0.082 | 0.0805 | 0.0783 | 0.082 | 0.0808 | 0.0786 | 5000 | 4 | 0.02 | (0.9,0.8) | -4.702 | |
| 0.0577 | 0.0559 | 0.074 | 0.0732 | 0.071 | 0.0738 | 0.0731 | 0.0709 | 0.0738 | 0.0732 | 0.071 | 5000 | 4 | 0.02 | (0.98,0.9) | -4.702 | |
| 0.0274 | 0.0277 | 0.0418 | 0.0449 | 0.0426 | 0.0415 | 0.0446 | 0.0423 | 0.0415 | 0.0449 | 0.0426 | 10000 | 2 | 0.02 | (0.9,0.8) | -4.702 | |
| 0.0274 | 0.0277 | 0.038 | 0.041 | 0.0387 | 0.038 | 0.0409 | 0.0386 | 0.038 | 0.041 | 0.0387 | 10000 | 2 | 0.02 | (0.98,0.9) | -4.702 | |
| 0.0267 | 0.0276 | 0.0371 | 0.0385 | 0.0374 | 0.037 | 0.0384 | 0.0373 | 0.037 | 0.0385 | 0.0374 | 10000 | 4 | 0.02 | (0.9,0.8) | -4.702 | |
| 0.0267 | 0.0276 | 0.0334 | 0.0351 | 0.034 | 0.0333 | 0.0351 | 0.034 | 0.0333 | 0.0351 | 0.034 | 10000 | 4 | 0.02 | (0.98,0.9) | -4.702 | |
| 0.0228 | 0.0221 | 0.0332 | 0.0343 | 0.0327 | 0.033 | 0.0341 | 0.0325 | 0.033 | 0.0343 | 0.0327 | 5000 | 2 | 0.05 | (0.9,0.8) | -3.793 | |
| 0.0228 | 0.0221 | 0.0301 | 0.0313 | 0.0297 | 0.0301 | 0.0313 | 0.0297 | 0.0301 | 0.0313 | 0.0297 | 5000 | 2 | 0.05 | (0.98,0.9) | -3.793 | |
| 0.0221 | 0.0222 | 0.0285 | 0.0299 | 0.0292 | 0.0284 | 0.0298 | 0.0291 | 0.0284 | 0.0299 | 0.0292 | 5000 | 4 | 0.05 | (0.9,0.8) | -3.793 | |
| 0.0221 | 0.0222 | 0.0261 | 0.0273 | 0.0266 | 0.0261 | 0.0272 | 0.0265 | 0.0261 | 0.0273 | 0.0266 | 5000 | 4 | 0.05 | (0.98,0.9) | -3.793 | |
| 0.011 | 0.011 | 0.016 | 0.0169 | 0.016 | 0.0159 | 0.0168 | 0.0159 | 0.0159 | 0.0169 | 0.016 | 10000 | 2 | 0.05 | (0.9,0.8) | -3.793 | |
| 0.011 | 0.011 | 0.0145 | 0.0154 | 0.0146 | 0.0145 | 0.0154 | 0.0146 | 0.0145 | 0.0154 | 0.0146 | 10000 | 2 | 0.05 | (0.98,0.9) | -3.793 | |
| 0.0108 | 0.011 | 0.0139 | 0.0147 | 0.0144 | 0.0138 | 0.0147 | 0.0143 | 0.0138 | 0.0147 | 0.0144 | 10000 | 4 | 0.05 | (0.9,0.8) | -3.793 | |
| 0.0108 | 0.011 | 0.0127 | 0.0135 | 0.0131 | 0.0127 | 0.0134 | 0.0131 | 0.0127 | 0.0135 | 0.0131 | 10000 | 4 | 0.05 | (0.98,0.9) | -3.793 | |
| 0.0108 | 0.011 | 0.0148 | 0.016 | 0.0153 | 0.0147 | 0.0159 | 0.0152 | 0.0147 | 0.016 | 0.0153 | 5000 | 2 | 0.1 | (0.9,0.8) | -3.111 | |
| 0.0108 | 0.011 | 0.0135 | 0.0146 | 0.014 | 0.0135 | 0.0146 | 0.0139 | 0.0135 | 0.0146 | 0.014 | 5000 | 2 | 0.1 | (0.98,0.9) | -3.111 | |
| 0.0108 | 0.011 | 0.0137 | 0.014 | 0.0137 | 0.0137 | 0.0139 | 0.0137 | 0.0137 | 0.014 | 0.0137 | 5000 | 4 | 0.1 | (0.9,0.8) | -3.111 | |
| 0.0108 | 0.011 | 0.0123 | 0.0128 | 0.0126 | 0.0123 | 0.0128 | 0.0125 | 0.0123 | 0.0128 | 0.0126 | 5000 | 4 | 0.1 | (0.98,0.9) | -3.111 | |

**WEB TABLE 54-** Empirical variance and mean of estimated variances of pure risk parameter $\log\left\{ \pi\left( \tau_{1},\tau_{2};\boldsymbol{x} \right) \right\}$ with $\boldsymbol{x}=\left( 1, 1, 0.6 \right)'$, from unstratified sampling using different methods of analysis and variance estimation, for various probabilities of missing covariate data, in 5,000 simulated cohorts

# DATA ANALYSIS

# Parameter estimation

Recall that estimation was performed using the stratified case-cohort with design weights (SCC); the stratified case-cohort with calibrated weights (SCC.Calib); the unstratified case-cohort with design weights (USCC); and the unstratified case-cohort with calibrated weights (USCC.Calib). We also estimated the parameters using the whole cohort ($n =$30,000).

Estimation results for all 11 parameters are display in **WEB TABLE 55**. Corresponding variance estimates are displayed in Table 3 in the Main Document. Interpretation of the results are given in Section 8 in the Main Document.

| Parameter | | Cohort | SCC | SCC.Calib | USCC | USCC.Calib |
| --- | --- | --- | --- | --- | --- | --- |
| $\beta_{1}$ | | 0.3845 | 0.3519 | 0.3843 | 0.3765 | 0.3863 |
| $\beta_{2}$ | | -0.8461 | -0.8418 | -0.868 | -0.9687 | -0.8327 |
| $\beta_{3}$ | | 0.2723 | 0.3017 | 0.2772 | 0.2745 | 0.2865 |
| $\beta_{4}$ | | 0.6836 | 0.6786 | 0.6803 | 0.7073 | 0.6863 |
| $\beta_{5}$ | | 0.7009 | 0.6862 | 0.7042 | 0.7087 | 0.7005 |
| $\beta_{6}$ | | -0.1208 | -0.1645 | -0.1276 | -0.1225 | -0.1423 |
| $\beta_{7}$ | | -0.2306 | -0.1799 | -0.2269 | -0.2309 | -0.2354 |
| $\pi\left( \tau_{1},\tau_{2};\boldsymbol{x} \right)$ | $\boldsymbol{x}=\left( 0,-0.4,0,1,\boldsymbol{0}_{3} \right)'$ | 0.2185 | 0.2219 | 0.2195 | 0.2318 | 0.2182 |
|  | $\boldsymbol{x}=\left( 0,0.4,0,1,\boldsymbol{0}_{3} \right)'$ | 0.1178 | 0.1201 | 0.1164 | 0.1144 | 0.1188 |
|  | $\boldsymbol{x}=\left( \boldsymbol{0}_{4},1,\boldsymbol{0}_{2} \right)'$ | 0.1638 | 0.1652 | 0.1642 | 0.1641 | 0.1639 |
|  | $\boldsymbol{x}=\boldsymbol{0}_{7}'$ | 0.0849 | 0.0869 | 0.0849 | 0.0845 | 0.085 |

**WEB TABLE 55-** Estimates of log-relative hazard and pure risk parameters from using different sampling designs, methods of analysis and variance estimation, in the Golestan Cohort ($n=$ 30,000). $\beta_{p}$ denotes the log-relative hazard parameter of covariate $x_{p}$, $p\in\{1,\ldots,7\}$

# STEP BY STEP PSEUDO CODE TO OBTAIN $\hat{\boldsymbol{V}}$ FOR SCC AND SCC.Calib IN TABLE 3 IN THE MAIN DOCUMENT

A. Estimation using the stratified case cohort with design weights -------------------

1. Run the coxph model to estimate the log-relative hazard from the case-cohort data with design weights

2. Estimate the baseline hazard non parametrically from the case-cohort data with design weights and log-relative hazard estimate obtained in step A.1

3. Estimate the pure risk from the relative hazard and baseline hazard estimates obtained in steps A.1 and A.2

4. Compute the influences of the case-cohort individuals on the parameter estimates

5. Estimate the variances of the parameter estimates from the influences obtained in step A.4

--------------------------------------------------------------------------------------

B. Estimation using the stratified case cohort with calibrated weights ---------------

1. Build a regression model to predict the phase-two covariates

2. Impute the phase-two covariates on the whole cohort using the model built in step B.1

3. Run the coxph model to estimate the log-relative hazard from the imputed cohort data obtained in step B.2

4. Compute the influences of the cohort individuals on the log-relative hazard estimate obtained in step B.3 to construct the auxiliary variables proposed by Breslow et al. (2009)

5. Calibrate the design weights against the influences obtained in step B.4

6. Run the coxph model to estimate the log-relative hazard from the case-cohort data with the calibrated weights obtained in step 5

1. Compute the total follow-up time on the pure-risk time interval on the whole cohort
2. Compute the relative hazard on the imputed cohort data, using the log-relative hazard estimate obtained in step 6.

9. Multiply the estimated relative hazard obtained in step B.8 to the total follow-up time obtained in step 7, to get construct an additional auxiliary variable, as proposed by Shin et al. (2020)

10. Calibrate the design weights against the influences obtained in steps B.4 and B.9

11. Run the coxph model to estimate the log-relative hazard from the case-cohort data with calibrated weights obtained in step B.10

12. Estimate the baseline hazard non parametrically from the case-cohort data with calibrated weights obtained in step B.10 and log-relative hazard estimate obtained in step B.11

13. Estimate the pure risk from the relative hazard and baseline hazard estimates obtained in steps B.11 and B.12

14. Compute the influences of the cohort individuals on the parameter estimates, taking calibration into account

15. Estimate the variances of the parameter estimates from the influences obtained in step B.14

#

# ACKNOWLEDGMENTS

We thank Dr. Barry Graubard and Dr. Edmund Jones for insightful comments and Dr. Arash Etemadi and GEMShare for documenting and sharing data from the Golestan Cohort. We are grateful to the Reviewers and Editors of Lifetime Data Analysis for valuable suggestions.

This work was supported by the Intramural Research Program of the Division of Cancer Epidemiology and Genetics, National Cancer Institute, National Institutes of Health. This work utilized the computational resources of the [NIH HPC Biowulf cluster](http://hpc.nih.gov/).

# DATA AVAILABILITY STATEMENT

R code and functions used for the simulations in Web Appendix D and Web Appendix G are available in the Supporting Information of this article and on GitHub at <https://github.com/Etievant/CaseCohort>. We are not authorized to release the clinical data used in Web Appendix H.

# REFERENCES

Barlow, W. E. (1994). Robust Variance Estimation for the Case-Cohort Design. *Biometrics*, *50*(4), 1064–1072. https://doi.org/10.2307/2533444

Borgan, Ø., Langholz, B., Samuelsen, S. O., Goldstein, L., & Pogoda, J. (2000). Exposure stratified case-cohort designs. *Lifetime Data Analysis*, *6*(1), 39–58. https://doi.org/10.1023/a:1009661900674

Breslow, N. E., & Lumley, T. (2013). Semiparametric models and two-phase samples: Applications to Cox regression. *From Probability to Statistics and Back: High-Dimensional Models and Processes -- A Festschrift in Honor of Jon A. Wellner*, *9*, 65–78. https://doi.org/10.1214/12-IMSCOLL906

Breslow, N. E., Lumley, T., Ballantyne, C. M., Chambless, L. E., & Kulich, M. (2009). Improved Horvitz–Thompson Estimation of Model Parameters from Two-phase Stratified Samples: Applications in Epidemiology. *Statistics in Biosciences*, *1*(1), 32–49. https://doi.org/10.1007/s12561-009-9001-6

Deville, J.-C., & Sarndal, C.-E. (1992). Calibration Estimators in Survey Sampling. *Journal of the American Statistical Association*, *87*(418), 376–382. https://doi.org/10.2307/2290268

Graubard, B. I., & Fears, T. R. (2005). Standard errors for attributable risk for simple and complex sample designs. *Biometrics*, *61*(3), 847–855. https://doi.org/10.1111/j.1541-0420.2005.00355.x

Gray, R. J. (2009). Weighted analyses for cohort sampling designs. *Lifetime Data Analysis*, *15*(1), 24–40. https://doi.org/10.1007/s10985-008-9095-z

Jiao, J. (2002). Comparison of variance estimators in case-cohort studies. *PhD Dissertation, University of Southern California.*

Lin, D. (2000). On fitting Cox’s proportional hazards models to survey data. *Biometrika*, *87*(1), 37–47. https://doi.org/10.1093/biomet/87.1.37

Pfeiffer, R. M., & Gail, M. H. (2017). *Absolute Risk: Methods and Applications in Clinical Management and Public Health*. Chapman and Hall/CRC. https://doi.org/10.1201/9781315117539

Reid, N., & Crépeau, H. (1985). Influence functions for proportional hazards regression. *Biometrika*, *72*(1), 1–9. https://doi.org/10.1093/biomet/72.1.1

Samuelsen, S. O., Ånestad, H., & Skrondal, A. (2007). Stratified Case-Cohort Analysis of General Cohort Sampling Designs. *Scandinavian Journal of Statistics*, *34*(1), 103–119. https://doi.org/10.1111/j.1467-9469.2006.00552.x

Shin, Y. E., Pfeiffer, R. M., Graubard, B. I., & Gail, M. H. (2020). Weight calibration to improve the efficiency of pure risk estimates from case-control samples nested in a cohort. *Biometrics*, *76*(4), 1087–1097. https://doi.org/10.1111/biom.13209
